# CATALISE: A Multinational and Multidisciplinary Delphi Consensus Study. Identifying Language Impairments in Children

**DOI:** 10.1371/journal.pone.0158753

**Published:** 2016-07-08

**Authors:** D. V. M. Bishop, Margaret J. Snowling, Paul A. Thompson, Trisha Greenhalgh, CATALISE consortium

**Affiliations:** 1 Department of Experimental Psychology, University of Oxford, Oxford, Oxon, United Kingdom; 2 Nuffield Department of Primary Care Health Sciences, University of Oxford, Oxford, Oxon, United Kingdom; Leiden University, NETHERLANDS

## Abstract

Delayed or impaired language development is a common developmental concern, yet there is little agreement about the criteria used to identify and classify language impairments in children. Children's language difficulties are at the interface between education, medicine and the allied professions, who may all adopt different approaches to conceptualising them. Our goal in this study was to use an online Delphi technique to see whether it was possible to achieve consensus among professionals on appropriate criteria for identifying children who might benefit from specialist services. We recruited a panel of 59 experts representing ten disciplines (including education, psychology, speech-language therapy/pathology, paediatrics and child psychiatry) from English-speaking countries (Australia, Canada, Ireland, New Zealand, United Kingdom and USA). The starting point for round 1 was a set of 46 statements based on articles and commentaries in a special issue of a journal focusing on this topic. Panel members rated each statement for both relevance and validity on a seven-point scale, and added free text comments. These responses were synthesised by the first two authors, who then removed, combined or modified items with a view to improving consensus. The resulting set of statements was returned to the panel for a second evaluation (round 2). Consensus (percentage reporting 'agree' or 'strongly agree') was at least 80 percent for 24 of 27 round 2 statements, though many respondents qualified their response with written comments. These were again synthesised by the first two authors. The resulting consensus statement is reported here, with additional summary of relevant evidence, and a concluding commentary on residual disagreements and gaps in the evidence base.

## Introduction

Unexplained language impairments in children are common, but there is little agreement about the criteria used to identify and classify such problems. This acts as a barrier to identifying children for prevention and intervention services. Furthermore, there is wide variation in the terminology used to refer to these children. Terms such as specific language impairment (SLI), language delay, developmental language disorder and developmental dysphasia are all used, sometimes with precise and sometimes with rather general meaning [1]. Confusion regarding criteria and terminology has been detrimental to clinical practice and to research. In part, this lack of consensus may have arisen because there are many professional groups involved, ranging from those with backgrounds in education, psychology, speech-language therapy (SLT)/pathology (SLP), paediatrics and child psychiatry. Even within the SLT/SLP profession, there is no consistency of terminology and criteria [2]. The current project was stimulated by discussions between a group of experts who initiated a campaign: Raising Awareness of Language Learning Impairments [3], which identified tackling these issues of criteria and terminology as a high priority.

The complex and multifaceted nature of language adds to the difficulties of identifying and categorising language impairments. In common usage, the terms speech, language and communication are often treated interchangeably, but they have distinct meanings. **Language** involves the comprehension and use of words and sentences to convey ideas and information. Language can occur in different modalities: spoken, written or signed. **Speech** refers to the production of vocal sounds, a process that involves both motor (articulatory) and linguistic skills. It is possible to have impaired speech but intact language, as in the case of someone with a physical impairment of the articulators who can express themselves through written language. Language and speech are both facets of **communication**, which encompasses the broader set of nonverbal and verbal means of conveying information and emotions.

In 2014, the International Journal of Language and Communication Disorders (IJLCD) included a special issue focussing on the topic of Specific Language Impairment [4]. This contained two position papers with commentaries representing a range of constituencies [1,5], and an overview paper [6]. It was apparent that not only was there widespread disagreement about terminology; there were also diverse viewpoints about which children should be regarded as requiring expert help for language difficulties.

One may wonder why there should be so much disagreement, when children's language disorders are included in two major diagnostic manuals, the International Classification of Diseases (ICD-10) [7] and the Diagnostic and Statistical Manual of the American Psychiatric Association [8]. However, the commentaries on the IJLCD position papers suggested that these biomedically-derived diagnostic systems are widely ignored or regarded as irrelevant, inadequate, or inappropriate: few commentators mentioned them, and those who did were either involved in drawing up the guidelines, and/or were critical of the resulting categories [9,10,11,12,13]. Children's language difficulties are at the interface between education, medicine and the allied professions. The professional group with primary responsibility for intervention with these children is speech and language therapists—SLTs (known as speech-language pathologists—SLPs—in North America and Australia; henceforth SLT/SLPs), but children with language difficulties are also seen by, and may be identified by, educational or clinical psychologists, paediatricians, psychiatrists, general practitioners and teachers. Day-to-day management of their difficulties is typically the responsibility of teachers, who may reject a 'medical model' of disability [14,15]. Disagreements cannot, therefore, necessarily be resolved by gathering further evidence: there are radical differences in how children's difficulties are conceptualised and classified. A further complication is that while genetic and neurobiological factors contribute to children's language problems [16], there are no biomedical tests for language impairment, and language development is also influenced by the child's social environment [17]. Nevertheless, one point of agreement between all those contributing to the special issue was that some children have language difficulties that are significant, i.e., severe and persistent enough to have serious negative consequences for their educational and social outcomes [6].

A final reason for disagreement is that definition of language impairment will depend on the purposes of those identifying the problems. The term "impairment" has been defined to mean "any loss or abnormality of psychological, physiological, or anatomical structure or function" [18]. In the context of language development, it could, for instance, refer to a limitation of short-term memory, poor auditory perception, or a failure to acquire mastery of grammatical inflections of the language [19]. These skills can be assessed using appropriate standardized tests, with cutoffs specified to correspond to mild, moderate or severe impairments. However, this may not be the most appropriate way to proceed if the goal is to identify children in need of extra help. Then the question becomes to what extent the child experiences difficulties with language function in everyday life; this may depend not only on the nature, number and severity of impairments in language and other systems, but also on the environment and any adjustments made to counteract the impairment. The World Health Organization's International Classification of Functioning, Disability and Health—Children and Youth (ICF-CY) framework [20] is consistent with such an approach.

In an effort to improve consensus in this area we adopted the Delphi technique [21], taking as our initial model the approach used by Greenhalgh and colleagues [22]. We decided to undertake two Delphi exercises; the first to consider the criteria that would be used to identify children in need of extra specialist help, and the second to address terminological issues. This paper reports on the first of these.

The Delphi is a consensus-building method that has key features that distinguish it from the other main approach that has been used, which is to gather experts together to discuss issues, either at a conference, or in a series of meetings:

1. The process goes through a series of cycles. In each cycle, a panel of experts is presented with a set of statements to rate, and feedback is then given that shows how each individual’s ratings compare with whole distribution. Items can then be dropped or modified in relation to the feedback, before the next cycle. This process is repeated until either consensus is obtained, or it is clear no consensus is possible.
2. As well a quantitative ratings, open ended comments can be included at the rating stage and fed back to all panel members. This way, panel members can attempt to influence the consensus by giving justification for their ratings.
3. The process is anonymised. This means everyone gets a chance to have their views taken into account, without senior individuals or forceful personalities dominating.
4. The Delphi can be run online. It does not require that everyone is in the same place at the same time; this facilitates international collaboration and gives people time to respond as they find convenient.

Note that, although quantitative ratings are used in the Delphi process, it is not equivalent to a simple voting system, because it incorporates interaction and engagement between panel members. It necessarily involves judgement, particularly at the stage between cycles when decisions are made to modify or drop items. The basis for doing this is to improve the likelihood of agreement in the subsequent round.

## Materials and Methods

### Identification of panel members

Selection of an expert panel is a key part of any Delphi exercise, and has been a topic of some debate in the literature [23]. It is important to have panel members who are committed to the project, have credibility, and are heterogeneous enough to represent the range of stakeholders who have an interest in results. In determining panel membership, key questions arose as to the scope of the exercise: in terms of what we aimed to achieve, and whether our focus would be national, multinational or multilingual.

In terms of the goal of the exercise, our focus was on those children who would traditionally be regarded as having specific language impairment (SLI), i.e. those with severe and persistent language difficulties who are at risk of educational and social problems even if taught by teachers who are skilled at supporting children’s communication. These are the children who need additional help beyond targeted help in the classroom and who should be referred to a SLT/SLP for more detailed evaluation and intervention tailored to their specific needs.

Given this focus, we deemed it appropriate to have predominant representation of SLT/SLPs, as this is the professional group that has particular expertise in children's speech and language difficulties. However, we thought it was important also to include representation of different professions involved with these children, including those from education, psychology, audiology and medicine, so that a variety of views could be aired and discussed. Our goal was to have a mix of individuals who had strong research credentials in this field and those who had extensive clinical experience, with some panel members combining both of these. In addition, we included representatives from charities whose primary focus is on supporting families affected by language impairments in children.

We restricted consideration to English-speaking countries. The issues we focused on are equally challenging in other countries, but manifestations of language difficulty, and terminology used to describe these, differ across languages [21]. Given that there are an estimated 6500 languages in the world, it would be unfeasible to cover all of them. Rather than encompassing all languages, we aimed to produce a study that might form a model for future studies in other languages. Nevertheless, we note that in many of the countries we included, significant proportions of the population speak more than one language, and we included items relevant to multilingual contexts.

Our focus was predominantly on the United Kingdom, but we aimed to include on the panel representatives of the other large English-speaking countries, i.e., Australia, Canada, Ireland, New Zealand and USA. Our rationale was that we were aware that experts in other countries planned to conduct their own consensus exercises; these might reach different conclusions related to different structures for education, insurance, health care and intervention. Our goal was to ensure our process kept us aware of any major discrepancies in approach from one discipline or country to another, so that we could, as far as possible, increase the likelihood that our guidelines would be acceptable beyond the narrow confines of one profession or nationality.

There is no agreement about the optimal size of a Delphi panel, with many including under 20 people [24],[25],[26]. The advantage of a larger panel is more representative coverage of experts, but a corresponding disadvantage is that the group discussion that is an inherent part of the process gets harder to achieve when more than 40 or 50 participants are involved [27]. We aimed to strike a compromise between coverage of a range of disciplines and geographical regions, and encouraging development of a cohesive group for discussion.

To form the Delphi panel we started with the individuals who had been asked to write commentaries for the IJLCD special issue, and all co-authors of the target articles, excluding Bishop and Snowling, who acted as moderators. These had been identified by the editor, Susan Ebbels, as representing a balance of academics and practitioners who could offer different perspectives on the topic of SLI. At this point, three individuals declined to take part—two of these judged they did not have sufficient expertise in children's language disorders, and one did not think the Delphi approach would be helpful.

The moderators then scrutinised the preliminary panel and recruited further members to ensure diversity in terms of gender and ethnicity, and to include representatives of the main English-speaking countries, i.e., Australia, Canada, Ireland, New Zealand, United Kingdom and USA. All those invited were deemed to be experts in children's language impairments. Summary characteristics of panel members are shown in Table 1. We also gathered information to establish the expertise of panel members: 54 members provided information on request and it was obtained from public sources for the remaining five. The mean number of years’ experience that panel members had working with children with language impairments was 24 (SD = 10.7). 51 panel members had provided training to others in their profession; 43 were involved in clinical or research advisory committees and 49 had published peer-reviewed papers or books relating to children’s language impairments. In addition, 10 had a child or close relative with a language impairment.

**Table 1:** Professional group and nationality of panel members.

Profession	N and Nationality
Speech-Language Therapist/Pathologist	32 (15 UK, 6 USA, 3 NZ, 3 Ire, 1 Can, 4 Aus)
Joint SLT/SLP and Psychologist	7 (3 Can, 2 Aus, 2 UK)
Psychologist/Educational Psychologist	8 (3 UK, 1 US, 3 Can, 1 Aus)
Paediatrician	3 (3 UK)
Psychiatrist	2 (1 UK, 1 Can)
Audiologist	1 (1 NZ)
Specialist teacher	2 (2 UK)
Charity representative	4 (4 UK)
Total	59

### Ethics approval

This research was approved by The Medical Sciences Interdisciplinary Research Ethics Committee, University of Oxford (approval number: MS-IDREC-C1-2015-061). The committee approved for panel members to give written consent for their ratings to be used to derive a consensus statement.

### Delphi consensus process

**Fig 1:**
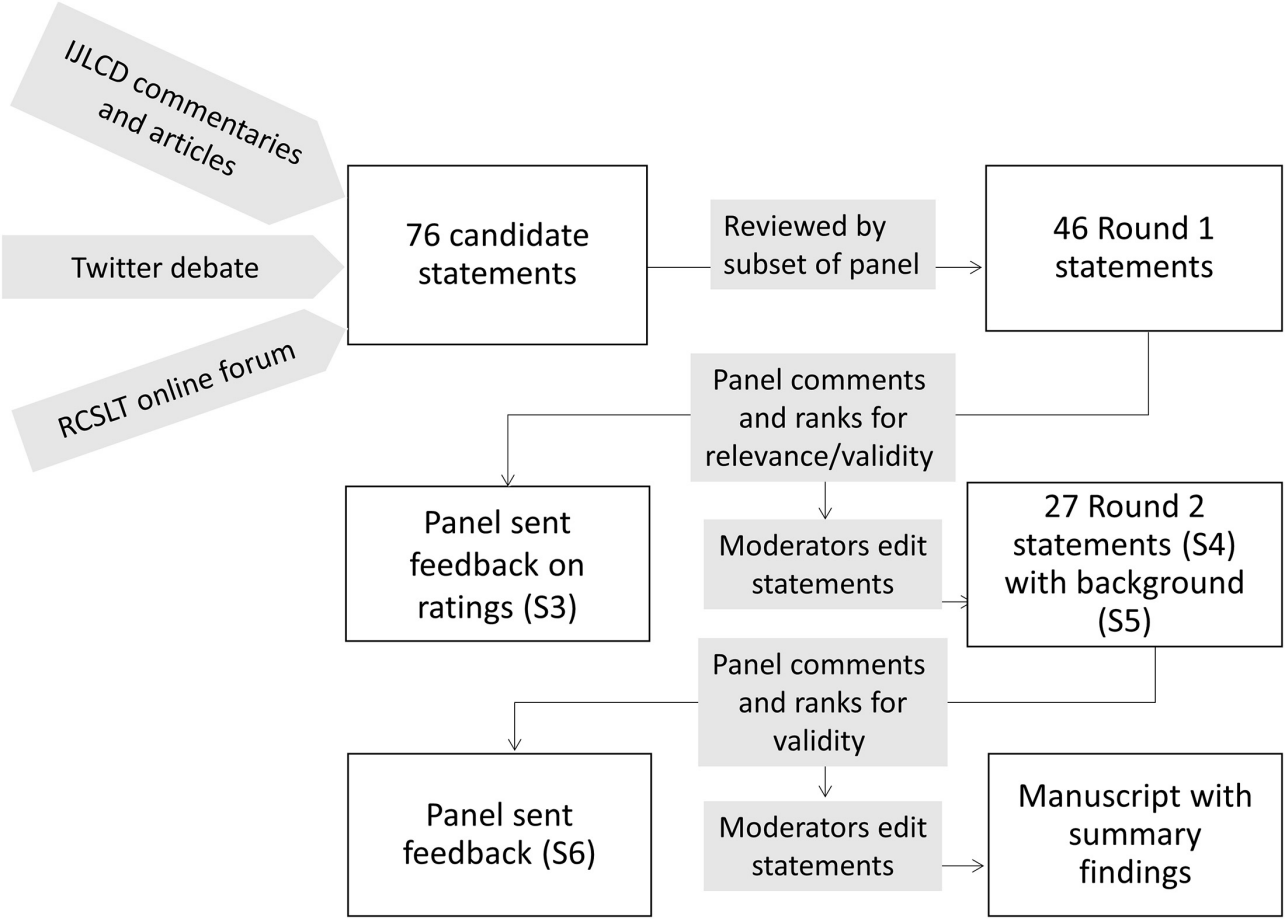
Flowchart showing stages in the Delphi consensus process. S3, S4, S5, S6 refer to Supporting Information documents.

The flowchart for the Delphi process is shown in Fig 1.

A Delphi process typically starts with an open-ended brainstorming session to identify topics that can form the basis for an initial pool of statements to be rated. This step had in effect already been undertaken with the publication of target articles and commentaries in IJLCD. We also engaged in internet-based activities designed to encourage further debate and to involve more extensive networks of professionals, including a discussion forum set up by the Royal College of Speech and Language Therapists [28], and a Twitter debate moderated by the first author using the @WeSpeechies curated meeting point [29]. Statements for evaluation were taken from these articles, commentaries and internet sources. These were then circulated to a subset of panel members (shown with * in the Consortium list in Acknowledgements) who advised on wording and added further suggested statements. On the advice of TG, who acted as adjudicator, we aimed for a pool of less than 50 items, and after further discussion between the moderators a potential pool of 76 items was pruned to 46 items for Round 1. A briefing document (see S1 Doc) was provided to give panel members the context of the exercise and to clarify its purpose. The statements were presented in survey form using the Qualtrics platform (www.qualtrics.com). These were prefixed by the text in S2 Doc to reinforce the background to the survey, and to emphasise that the goal was to seek consensus on how to identify children in need of extra, specialist help with language, beyond what is usually available in the classroom.

After some initial items that asked about professional background, the 46 Round 1 statements were presented, and panel members were asked to rank each one twice on a seven-point Likert scale ranging from "strongly against" to "strongly in favour", once for relevance (i.e., should a statement on this theme/topic be included in our guidelines?) and once for validity (i.e., to what extent do you agree with the statement as currently worded?).

Participant responses to Round 1 were collated, the distribution of responses and associated anonymised comments were fed back to all panel members by PT. Responses and comments were scrutinised by the moderators, who remained blind to the identity of those commenting. The adjudicator gave general advice on procedure, e.g. it became clear that the 'relevance' dimension was not needed, as all but two items were deemed relevant, and so this dimension was dropped in Round 2.

Between Round 1 and Round 2 we departed from our original protocol in one respect: we discovered that a group in the Netherlands had conducted a Delphi exercise [30] attempting to specify criteria (which they termed 'red flags') for identifying major speech or language problems in different age bands. The importance of taking age into account was one issue flagged in Round 1 free text comments, and so we decided to incorporate some of these items in Round 2. We also moved to a five-point rating scale for Round 2.

On the basis of ratings and comments, and advice from the adjudicator, the two moderators agreed on rewording of some items, amalgamation of others, and removal of yet others. The set of items used in Round 2 consisted of the 27 statements shown in S4 Doc, grouped into three broad categories; referral for specialist assessment/intervention, assessment, and accompanying conditions. The S4 Doc materials also show the relationship between Round 1 and Round 2 items, and are colour-mapped to show extent of agreement with each statement in both rounds. Since it was evident that a short statement was not always sufficient without explanatory context, for Round 2, the panel was asked to read a background document giving a more detailed rationale for each statement before rating it (S5 Doc).

In addition, we used hierarchical cluster analysis with Round 1 scores to see whether there were obvious patterns in responding related to either the country of the respondent or the professional discipline. This technique begins with a point as a cluster and then repeatedly merges nearest neighbour clusters until a single cluster remains. The pattern of clusters can then be displayed graphically as a dendrogram. The analysis was performed using the R statistical software [31] using the pvclust package [32], which performs hypothesis tests, via bootstrapping, to determine whether a cluster truly exists.

## Results and Discussion

### Round 1

S3 Doc is a report showing quantitative and qualitative responses to the Round 1 statements; a personalised copy of this report was sent to all panel members, showing how their own responses related to those of other panel members. As noted above, the moderators examined the anonymised quantitative scores and qualitative comments for each item, and generated new Round 2 items designed to improve consensus.

Hierarchical cluster analysis was used to test the hypothesis that structure was present in Round 1 data that correlated with the predefined groupings of country or discipline. After trying some different fitting procedures, no coherent groupings emerged; very few clusters reached statistical significance, and none was stable across different analytic approaches. We cannot draw strong conclusions from this analysis, given the limited number of items and the small number of panel members, but the analysis was consistent with our impression from the original IJLCD articles and commentaries that, although there was a wide variety of views, these did not align with either national or professional boundaries.

### Round 2

For Round 2 we received responses from 57 of our 59 original respondents (96.7%). One did not respond, and the other sent in responses too late for inclusion. Again, the panel provided rich qualitative data in the form of comments as well as ratings, and some also provided references giving supportive evidence for their ratings.

S6 Doc is the report that was sent to all panel members indicating anonymised quantitative and qualitative responses to the Round 2 statements. There was a high level of agreement for most statements, with all items achieving at least 72% agreement (slightly favour, favour or strongly favour), and 24 of the 27 statements achieving 80% or more agreement. (For summary, see also colour-coded indication of agreement in S3 Doc).

### Final version of consensus statements

**Fig 2:**
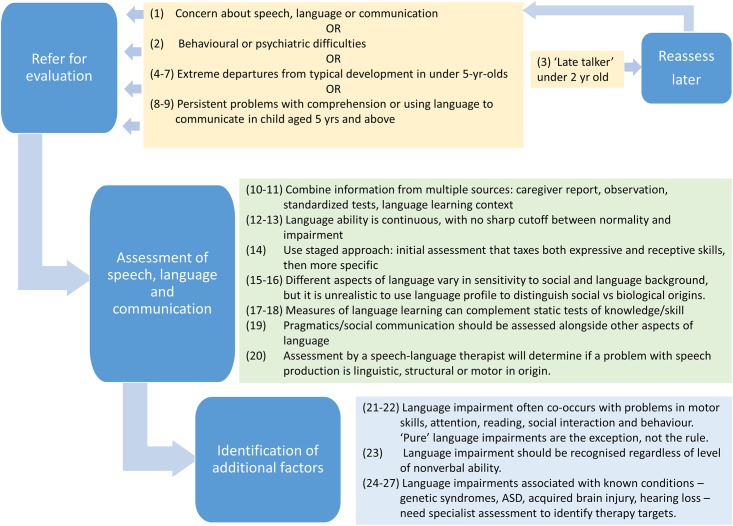
Final set of statements in precis form.

Even though there was a high level of agreement for Round 2 statements, we made some further modifications to the statements and to the background document to take into account the comments and additional sources of evidence provided by the panel. Some items were re-ordered (see S4 Doc). The revised set of modified statements and background explanation was circulated to the panel for further comment, and the current paper represents the final agreed version. Fig 2 provides a precis of the main messages embodied in the final set of statements.

Please note that any supporting references are provided in the supplementary comments, rather than in the statements themselves. These do not constitute a detailed literature review, but are based on references that were contributed by the moderators and panel members to support specific statements.

**When should a child be referred for specialist assessment/intervention**?

*1*. *Reasons for referral for specialist assessment/intervention include concern about speech, language or communication expressed by caregivers (which includes parents and guardians), teachers or healthcare professionals, or a lack of progress in language or scholastic attainment despite targeted classroom assistance*.

**Supplementary comment:** We recommend reliance on concerns expressed by those who know the child rather than universal screening. Screening of a whole population has the potential advantage that it can identify a child whose difficulties might otherwise go undetected. However, as emphasised in the classic text by Wilson and Jungner [33], any screening program must beware of over-identification of problems, which can lead to resources being diverted to cases that do not need them. Even where an instrument has good sensitivity and specificity, it may have weak positive predictive value (percentage of those identified who have problems) in the general population if the condition that is screened for is relatively uncommon [34]. Although screening has been introduced in some places, there is concern that universal screening for language impairment is not advisable in toddlers, because early language delay often resolves and currently available tests lack adequate sensitivity and specificity for predicting longer-term problems [35] (see also statement 3). The most recent US Preventive Services Task Force recommendation on this topic stated that there was insufficient evidence to assess the benefits and harms of screening for speech and language delay and disorders in children aged 5 years and under [36], though they qualified this recommendation by stating it applied to asymptomatic children where there was no parental or clinical concern.

*2*. *Language impairments may go undetected. Referral for language assessment is recommended for children who present with behavioural or psychiatric difficulties, and for children with poor reading comprehension or listening difficulties*.

**Supplementary comment:** The high prevalence of unsuspected language impairments in these populations motivates this recommendation [37,38,39,40].

*3*. *Many late talkers (children with limited expressive vocabulary at 18–24 months) catch up without any special help. Research to date has shown it is difficult to predict which children will go on to have longer-term problems. Children at greatest risk of persisting problems are late-talkers with poor language comprehension, poor use of gesture, and/or a family history of language impairment. Nevertheless, even with these indicators, prediction of outcomes for individual children is unreliable, and except where problems are severe (as in item 4). Therefore reassessment after six months is recommended in our current state of knowledge*.

**Supplementary comment:** The desirability of early intervention is often taken as a given, but many late-talking toddlers catch up without special help [41], and there are disadvantages of intervening with children who would outgrow their problems [42,43]. In addition, some children who have language difficulties at 4 to 5 years of age were not late talkers [41,44,45].

*4*. *Between 1 and 2 years of age, the following features are indicative of atypical development in speech, language or communication: (a) No babbling (b) Not responding to speech and/or sounds; (c) Minimal or no attempts to communicate*

*Children showing any of these features should be referred for expert assessment to determine whether there is evidence of hearing loss, autism spectrum disorder or intellectual disability*.

**Supplementary comment:** This statement and statements 5–7 are based on Visser-Bochane et al [30], who described these as 'red flag' behaviours that their Delphi panel regarded as **definitely atypical** at this age. Note that these items describe a consensus view of clinicians, rather than empirically validated criteria. It is also important to stress that **many children who exceed these minimum levels of language and communication nevertheless have language problems**. As shown in Fig 2, items 4 to 7 indicate definite abnormality giving cause for concern, but in deciding when to refer for evaluation the more general aspects in items 1–2 should also be taken into account.

In very young children, it can be difficult to draw clear distinctions between speech, language and communication disorders; for instance, a child may fail to babble because of some lack of communicative intent, or because of a problem with speech perception or production. We therefore include here fairly nonspecific early indicators of communicative problems, which may be indicative of autism spectrum disorder (ASD), hearing loss and/or intellectual disability. Note, too that some children who subsequently are identified with language impairment may not have had such evident communicative problems at this age [46].

*5*. *Between 2 and 3 years of age, any of the following features is indicative of atypical development in speech, language or communication: (a) Minimal interaction; (b) Does not display intention to communicate; (c) No words; (d) Minimal reaction to spoken language; (e) Regression or stalling of language development*.

**Supplementary comment:** As with item 3, these are criteria for detecting severe difficulties that may indicate a range of underlying concerns, including autism spectrum disorder, intellectual disability or hearing loss. Children with these features should definitely be referred for evaluation, but other children in this age range with milder difficulties would also be referred on the basis of statements 1 or 2.

*6*. *Between 3 and 4 years of age, any of the following features is indicative of atypical development in speech, language or communication: (a) At most two-word utterances; (b) Child does not understand simple commands; (c) Close relatives cannot understand much of child's speech*

**Supplementary comment:** These criteria encompass a broad range of speech, language and communication skills. It does not follow that all children meeting these criteria will prove to have significant language problems, but they should be referred so that this can be evaluated and the nature of the underlying problem established. As with items 4–5, other children in this age range who do not show these features can also be referred on the basis of statements 1 or 2.

*7*. *Between 4 and 5 years of age, the following features are indicators of atypical language development*:

(a) Inconsistent or abnormal interaction (b) At most three word utterances (c) Poor understanding of spoken language; (d) Strangers cannot understand much of child's speech; (e) Close relatives cannot understand more than half of what child says

**Supplementary comment:** As with items 4–6, these are broad guidelines that can be understood by non-experts that may be helpful for flagging up children who need specialist evaluation to establish the nature and severity of any problems. Other children in this age range who do not show these features can also be referred on the basis of statements 1 or 2.

*8*. *Children's language can change dramatically, especially in the preschool/early school years (aged 4 to 5 years), even if there is no intervention. However, severe language impairment involving both comprehension and expression is more likely to be persistent*.

**Supplementary comment:** Rapid changes are sometimes seen in this age range, even in the absence of intervention [47]. The evidence supports the idea that, except where problems are severe, a staged approach to intervention is appropriate for many children of this age, with specialised provision focused on children who do not respond to good classroom practice and targeted intervention provided by teachers [48]. A severe problem would be one where the child had very limited comprehension, with impact on everyday interactions at home and school.

*9*. *From 5 years of age upwards, the following features are indicators of atypical language development: (a) Difficulty in telling or re-telling a coherent story (producing narrative) (b) Difficulty in understanding what is read or listened to (c) Marked difficulty in following or remembering spoken instructions (d) Talking a lot but very poor at engaging in reciprocal conversation (e) Many instances of over-literal interpretation, missing the point of what was meant*.

**Supplementary comment:** These flags are intended to alert those working with school-aged children to the range of ways in which language difficulties can manifest, but they represent a synthesis of clinical opinion and are not formally validated. In England, profiling based on a statutory framework, the Early Years Foundation Stage Profile (EYFSP), is devised to evaluate progress in various academic domains, including language and communication, for children at the end of their reception year, aged 4 yr 10 to 5 yr 9 mo. The EYFSP provides concrete examples of what kinds of language expression and comprehension abilities should be achieved, but, unfortunately, it fails to take age into account. There is now evidence that the EYFSP over-identifies younger children as not reaching the expected level [42,49], and so it is not recommended for identifying children in need of additional help.

**Aspects of language assessment**.

*10*. *Multiple sources of information should be combined in assessment, including interview/questionnaires with parents or caregivers, direct observation of the child, and standardized age-normed tests or criterion-based assessments*.

**Supplementary comment:** All these sources of information can play a role, depending on the purpose of assessment [50]. An interview with a caregiver and/or questionnaires completed by adults who know the child well can pick up functional impairments that may be missed by other methods [51,52,53]. Clinical observation gives an indication of how the child functions in a more naturalistic setting, but reliability of observation can be hard to establish, and interpretation depends heavily on the experience of the clinician. Methods have been developed for standardized collection and computer-aided analysis of naturalistic language samples, which can then be evaluated against normative data, to give estimates of both grammatical and vocabulary development [54]. A well-standardized test that has good reliability, validity and sensitivity can provide a less labour-intensive way of quantifying severity of language impairment relative to a peer group in a relatively objective manner, but needs to be interpreted cautiously if the child's background is not comparable to the standardization population. Also, many standardized tests are relatively insensitive to change over time. A criterion-referenced test can help pinpoint targets for intervention, but the significance of impairments will be age-dependent. Finally, the child's own perspectives on day-to-day challenges should be solicited where possible.

*11*. *A low score on a language test should be interpreted in relation to information from observation and interview; functional impact as well as test performance needs to be taken into account when identifying the child's needs*.

**Supplementary comment:** Standardised tests can indicate problems with specific components of language and communication—especially those that may otherwise go undetected, such as problems with comprehension. Establishing level of functional impairment is important [20] and methods are being developed for evaluating this more systematically [52,53]. Results from a language test should be considered in relation to information from caregivers, teachers and other professionals to help select targets for intervention.

*12*. *There is no clear cut-off that distinguishes between language impairment (regardless of its cause) from the lower end of normal variation of language ability*.

**Supplementary comment:** Language impairment can be a secondary consequence of known conditions, such as hearing loss, genetic syndromes, or epilepsy, but in many cases there is no known cause, and no clear cutoff between impairment and normal variation [55,56]. Obesity and high blood pressure provide useful analogies: both are conditions that can arise for a range of reasons, but there is often no obvious cause, and the cut-off between normal and abnormal is arbitrary; nevertheless, those falling in the more extreme range merit intervention. Regardless of the cause, where a person's language abilities fall at the low end of the normal range, it can be appropriate to recommend intervention, ranging from environmental adjustments to specialised help, depending on the severity and nature of the problems and accompanying risk factors. However, it should be noted that many children who are judged clinically to have language impairments score within one SD of the mean on many commonly used language tests [57,58]. This suggests that many instruments used to assess child language are insensitive to impairments that affect day-to-day language functioning, possibly because items can be answered using nonlinguistic compensatory strategies.

*13*. *For research comparing rates of language impairment over time, or in different places, it would be useful to have a standard set of criteria based on a test battery that covers a range of aspects of expressive and receptive language*.

**Supplementary comment:** Clearly, prevalence will depend on the cutoff used. In ICD-10 [7] there is a requirement for a score on an individually-administered standardized language test to be two standard deviations below the mean. However, this begs the question of which test to use, and how to combine information from different language components, especially when there is an uneven language profile. Tomblin et al. [59] investigated a range of possible criteria in an epidemiological study. They settled on the EpiSLI criterion, which is based on five composite scores from norm-referenced tests of receptive and expressive language in three domains of language. Children with two or more composite scores below the 10^th^ centile (i.e. 1.25 standard deviations or more below the mean) were considered to have a language disorder. Many children identified by this criterion had not been identified by caregivers or professionals as having language difficulties. Nevertheless, a follow-up in adolescence confirmed that children identified this way often had persistent problems [60].

*14*. *When using standardized tests, a staged approach to language assessment is efficient, with an initial age-appropriate instrument that taxes a range of receptive and expressive skills (e.g. tests involving narrative retelling and/or sentence repetition), to give an indication of the nature and severity of impairment, followed by more specific assessments as necessary*.

**Supplementary comment:** There are many components to language, but it is seldom feasible to evaluate all of them in an initial assessment, even if suitable instruments are available. An initial assessment should suggest hypotheses about factors that lead to language impairment, which can then be assessed with more specific measures. Evaluation of component skills can be conducted alongside intervention and be informed by response to intervention. N.B. Pragmatic difficulties, which are difficult to assess using traditional assessments, are specifically considered in item 19, below.

*15*. *There is no distinctive language profile associated with social disadvantage*.

**Supplementary comment:** Language development will be influenced by social and linguistic environment as well as by biological differences between children (e.g. due to genetic and prenatal influences). Attempts have been made to distinguish between social and other causes of language difficulties using the child's profile of language skills. Some panel members noted that an uneven, 'spiky' profile of skills is sometimes equated with language disorder, whereas a more even pattern corresponds to language delay, which is assumed to be due to inadequate language experience. However, there is no supporting evidence for this approach. A second view is that social and non-social factors are associated with different types of language difficulty. There is some research showing that measures of learned knowledge, e.g., vocabulary, are more sensitive to social disadvantage than measures that reflect language processing, such as nonword repetition [61,62]. However, these trends do not provide an adequate basis for categorising individual children as having social vs. non-social causes of language difficulties. In practice, it is over-simplistic to treat these as alternative explanations for language difficulties, as both social and non-social risk factors often co-occur and may interact [63].

*16*. *Aspects of language impairment that are relatively uninfluenced by social and cultural background are nonword repetition, sentence repetition, and production of grammatical inflections marking verb tense. Some studies have found these to give good agreement with clinical diagnosis of language impairment, at least for children whose main difficulties are with language form, rather than content or use*.

**Supplementary comments**: A body of work on 'markers' for language impairment has identified these particular aspects of language as promising for identifying children with language difficulties [64,65,66,67]. Both nonword repetition and aspects of grammatical inflection production have been shown to have strong genetic influence, and also to be relatively independent of social background [68,69]. Nevertheless, this work is a long way from clinical application; we need further research to establish how these aspects of language align with functional impairments, to improve their sensitivity and specificity in a clinical context [70], and to consider how they change with age. Finally, it is important to note that some children with significant language difficulties are unimpaired on these aspects of language.

*17*. *Assessment approaches that explore how children learn language provide a promising approach. They can be integrated with intervention to give an indication of responsiveness to specific approaches. However, although there has been much interest in this approach in the field of reading disabilities, there has been relatively little research on its application to children's language learning difficulties*.

**Supplementary comments:** An assessment that uses a test, teach, and retest approach can be helpful for indicating whether the child is ready for this level of language modification and for identifying intervention targets [71,72,73]. Dynamic assessment embodies such ideas as well as exposing the child to different kinds of prompt and support to identify how the child responds. In principle this kind of method might help distinguish children whose difficulties are due to lack of exposure from those whose learning is impaired (see item 18). However, more work is needed to translate research in this area into clinical practice. We might learn from the field of reading, where measures of Response to Intervention have been used as part of the criteria to identify children with reading disabilities [74,75,76]

*18*. *Children with English as an Additional Language (EAL) pose challenges, because it can be difficult to determine if poor mastery of English reflects a genuine language problem or a lack of exposure to English. Where there is a language problem, this will be evident in the home language(s), but direct assessment of this may not be feasible. Report from a family member, by interview or checklist, may be able to clarify whether or not the child's skills in the home language are giving concern. Dynamic assessment (item 17) also has promise in this area*.

**Supplementary comment**: There is a wide body of evidence showing that growing up with more than one language is unproblematic, and can be advantageous, for many children [77]. At 30 months of age, children who have at least 60% exposure to English will usually have similar language competence to a native English speaker [78]. Nevertheless, we should be alert to the possibility of children with EAL who have language learning difficulties in the home language [79,80]. Furthermore, in some contexts, having a different language at home and school is a risk factor for poor academic achievement, and some children with EAL will benefit from additional language support [81]. Even where translated assessments are not feasible, parental report can be used to indicate mastery of the home language [82]. There has also been an active research focus on the use of dynamic assessment to identify children with language-learning impairments as opposed to those with lack of learning opportunity [83,84,85].

*19*. *Training of speech and language therapists/pathologists should encompass assessment and planning of intervention for children who have pragmatic difficulties (including those diagnosed with DSM-5 social communication disorder). Other professional groups, including educators and psychologists, may also play a major role in identifying and planning for the needs of these children*.

**Supplementary comment**: This item relates to the construct of pragmatic language impairment, a term used to refer to cases of non-autistic children with poor pragmatic skills [86]. Some of these children also have structural language problems, but others do not. The term 'social communication disorder' (SCD) is very close in meaning to the term 'pragmatic language impairment', which has been adopted in the UK, but does not have any formal status. There has been concern that social communication disorder has been introduced in DSM-5 without any validation studies, and without clear diagnostic guidelines [87]. There is also concern that these children could 'fall through the cracks' because they do not meet criteria for autism services, and may also not appear to have a classical language impairment. Research on assessment and intervention for pragmatic problems is still in its infancy [88]. Checklists completed by caregivers or others who know the child well may be the most useful approach for identifying pragmatic difficulties of functional significance [89,90].

*20*. *Speech and language therapists/pathologists have specialist expertise in the assessment of problems with production of speech sounds, many of which are linguistic rather than motor/structural in origin. Speech difficulties can occur separately from or together with other language difficulties, and have different prognosis and intervention needs*.

**Supplementary comments:** Problems with expressive phonology are identified when the child collapses or substitutes phonological categories despite there being no structural or motor reason for this. Such problems have not been treated consistently in systems of terminology and classification [91]. Because phonology is part of language, one can make a logical case that they should be categorised as part of language impairment. In practice, however, difficulties restricted to production of speech sounds often (but not invariably) occur in the absence of other language difficulties [92,93], and have different prognosis and intervention needs. Therefore, if these are included under the umbrella of language impairment, they need to be recognised as a distinct subgroup. Nevertheless, a speech problem can be the most obvious problem in a child with more pervasive language difficulties, so it is important that a child presenting with speech difficulties has both speech and language assessed by a SLT/SLP. Speech problems persisting into school age are associated with a risk of literacy problems, particularly when the child also has other language difficulties [94].

**Relation of language impairment to other developmental difficulties**.

*21*. *Language impairment frequently co-occurs with other neurodevelopmental difficulties, including attentional problems, motor impairments, reading difficulties, social impairment and behaviour problems*.

**Supplementary comments:** Co-occurring problems are common in clinically referred children and should not be a reason for ignoring a language impairment [95]; they should be documented, the presence of these additional difficulties may affect prognosis and intervention strategies. A multidisciplinary approach to assessment and intervention can be useful to give a full picture of the child's needs.

*22*. *Much research has adopted narrow exclusionary criteria, with a focus on identifying and studying children with 'pure' language impairments. However, in clinical contexts, restricting attention to 'pure' cases is not appropriate as most language impaired children have additional problems*.

**Supplementary comments:** Criteria for language impairment will depend to some extent on the question being asked, and there will be occasions when researchers need to adopt narrow, exclusionary criteria to minimise confounding and define a homogeneous group; however, there is now ample evidence that many, perhaps most, children receiving clinical services for language difficulties have additional problems [96].

*23*. *In general, language impairment should be identified regardless of whether there is a mismatch with nonverbal ability. Where a child has a language impairment in the context of markedly poor nonverbal functioning and/or significant limitations of adaptive behaviour, the primary diagnosis should be intellectual disability, with a secondary diagnosis of language impairment*.

**Supplementary comments:** This topic was the most controversial of those we covered, and some panel members did not agree with this final statement. Nevertheless, on the basis of majority opinion, supported by research evidence, we do not endorse the traditional view, still used in some diagnostic systems, e.g., ICD-10 [7], of recognising language impairment only when there is a significant mismatch with nonverbal IQ. This kind of definition has come under attack from four directions. First, there has been a move away from sole reliance on IQ tests for diagnosing intellectual disability, to take into account ability to function adaptively in everyday life in terms of reasoning and judgement [97]. Second, it has been shown that, in children with language impairments, level of nonverbal skills is not a reliable indicator of potential, does not determine response to language intervention [98,99,100,101] and is not associated with a unique linguistic profile [102,103,104]. Third, discrepancy scores are so unstable that they cannot provide a reliable basis for classification or diagnosis [105]. Fourth, adequate language functioning is found in many children with low nonverbal IQ, contradicting the notion that nonverbal ability sets some kind of limit on rate of language development [106]. In sum, where low nonverbal ability accompanies poor language skills, it should be seen as a correlate rather than an explanation. The key consideration in identifying language impairment is whether the child is likely to benefit from intervention and that is not determined by IQ. Indeed, restricting intervention to those with a large IQ discrepancy risks denying services to the children with the most severe and extensive needs [37].

*24*. *The language difficulties of children with autism spectrum disorder (ASD) require an approach to intervention that addresses social and behavioural as well as language difficulties. Nevertheless, many children with autism have problems with structural aspects of language similar to those seen in some non-autistic children*.

**Supplementary comments:** For many years autism was regarded as quite distinct from other developmental language difficulties, and diagnosis of ASD would lead to a different educational/intervention pathway. However, it is now recognised that the distinction between ASD and other conditions is not as clearly delineated as some textbooks might suggest. On the one hand, there are children with Social Communication Disorder/ Pragmatic Language Impairment, who have pragmatic impairments without all the features necessary for a diagnosis of ASD (see item 19). On the other hand, a high proportion of verbal children with ASD have language difficulties similar to those seen in non-autistic children, especially with grammar or phonology [107], though there is debate as to whether the similarities are merely superficial [108]. Where structural language impairment co-occurs with ASD there are more severe problems with receptive language and functional communication [109]. There is as yet no research evidence on whether intervention approaches used with language-impaired children are effective for analogous difficulties in ASD.

*25*. *Children with known syndromes (e.g. Down syndrome, Klinefelter syndrome) often have accompanying language problems that resemble those seen in children with no known aetiology*.

**Supplementary comments**: It is important to recognise the need to assess language skills in children with genetic syndromes and not assume they will be unresponsive to treatment. In both Down syndrome and Klinefelter syndrome, the profile of language impairment is similar to that seen in classical specific language impairment [110,111]. Where children have language impairments, this should be identified as a co-occurring feature. There is little research on interventions for these groups; it seems plausible they would respond to the types of intervention used with children whose language difficulties have no known cause.

*26*. *Children with acquired language impairment (e.g. caused by stroke, tumour, or traumatic brain injury) are likely to have a different prognosis from those with developmental problems with no acquired aetiology*.

**Supplementary comments:** Language difficulties after acquired brain injury in children are rare. It is difficult to generalise about outcomes because these will depend on the age of the child and the nature and location of the lesion [112]. There can be good recovery even after severe focal damage in young children [113,114]. Nevertheless, formal assessment is important because it may reveal persisting problems associated with poor academic outcomes [115].

*27*. *Moderate-severe-profound hearing loss is typically associated with difficulties in learning oral language, but most hearing-impaired children demonstrate normal sign language skills if exposed to signing early in life. However, some children have language abilities—in spoken and/or signed language—that are well below those of their hearing-impaired peer group, and may be regarded as having a disproportionate language impairment that is not secondary to hearing loss*.

**Supplementary comments:** A child with a sensorineural hearing loss learns oral language via speech-reading plus a degraded auditory signal which is only partly compensated for by hearing aids or a cochlear implant. Even with mild-moderate hearing loss, there is typically some delay in acquiring both spoken and written language [116]. Most children with hearing impairment demonstrate normal language skills in the visual modality if exposed to a sign language early in life. Nonetheless, it is possible to have an impairment in acquiring sign language, just as in spoken language [117]. In a similar vein, some children make unexpectedly poor progress with spoken language after a cochlear implant [118]. It used to be thought that a fluctuating, conductive hearing loss associated with otitis media could lead to persistent language impairment, but a meta-analysis of prospective studies indicated this was not the case [119]. Language assessment and intervention with hearing-impaired children requires specialist skills.

### Study limitations

Although we have noted the advantages of the Delphi technique over in-person consensus meetings, it is important also to recognise its limitations [120]. Two issues that are particularly pertinent to the current study concern how composition of the panel could affect results, and how far there is potential for manipulation by those administering the study. As explained in the Introduction, our panel included representatives from a range of disciplines, but with a predominance of SLT/SLPs, because our aim was to produce recommendations relevant to referral to this professional group. It is possible that somewhat different conclusions might be reached if there had been a higher proportion of representatives of other disciplines, such as education or medicine, or if we had included more parent representatives on the panel. We also had a predominance of panel members from the UK, where SLT is largely funded by the National Health Service, whereas for those from other health systems, the implications of referral may be different. The implications for other systems could be fruitfully worked through by running additional Delphi exercises on a country-specific basis, but we hope that the consensus statements we have produced will provide a useful starting point for further work.

We aimed to avoid bias in the conduct of the process by ensuring that data-processing and feedback were handled by PT, whose expertise is in biostatistics rather than language impairment, and guidance about the overall process was provided by an adjudicator, TG, who was from a different research area. In contrast, the two moderators were both experts in children’s language disorders. They remained blind to the identity of those making ratings and were not themselves involved in contributing ratings, though they did select the initial pool of items (albeit from material representing a wide range of views), selected a subset of panel members, and were also responsible for deleting, rewording or combining items between rounds 1 and 2, and for rewording items between round 2 and the final paper. Having said that, we note that it would not be feasible for someone to select and revise items intelligently if they did not have expertise in the area. Furthermore, at every stage there was scope for panel members to disagree, and it is clear that in the initial round there was substantial disagreement on some items, indicating that we had not just selected a group of like-minded individuals. Further, the final manuscript was a collaborative effort with substantial input from the panel.

We conclude this section by arguing that there is no one true solution to the question of how to identify children for special help: the wording of the statements and the degree of consensus around each one may have differed with a different panel. Furthermore, it is impossible to be completely neutral about controversial issues: we all bring our own prejudices to bear. Nevertheless, we take some reassurance from the fact that the final set of statements was not obviously aligned with any one profession, and the statements are, in general, supported by research literature. A Delphi exercise is a relatively inexpensive method for consensus-building, and we hope that further research will be done applying this approach to children’s language disorders and related conditions, so that the robustness of the outcome can be further evaluated. Our approach is very different from that adopted by those developing guidelines such as DSM5, where a panel of experts recommends changes to existing criteria on the basis of a quality review of the extant research evidence. That approach has much to recommend it, but it may fail to be accepted if those with everyday clinical experience do not accept the resulting criteria. In the current Delphi, views that were supported with evidence were given most weight, but there were topics that were clearly deemed important but where there was little research to guide decisions. Where this was the case, the extent of consensus determined the outcome. In this way, the Delphi process not only helped achieve consensus statements, but also identified priorities for future research.

## An Agenda for Future Research, Education and Training

In some instances, disagreement between panel members reflected differences in opinion about how language difficulties should be conceptualised. Such disagreement is unlikely to be resolved by further research. There were also some disagreements that reflected concern about resource implications: would a modification of criteria for identifying children in need of extra help lead to that extra help being spread too thinly? However, there was strong agreement that decisions about identifying problems should not be influenced by what we know about available resources. Other disagreements reflected the inadequacy of the evidence base in this field. We note here specific instances of this kind, in the hope that this might stimulate research on these issues, so we can come to evidence-based conclusions in the future. Similarly, CATALISE revealed a number of issues about which the panel felt there was a lack of understanding amongst professionals and practitioners (and to some extent caregivers); for instance, the lack of clear boundaries between language impairment and typical development, and the difficulty of attributing a language impairment to a single cause in most cases. Here we discuss the emerging agenda for research, practice and continuing education.

### Research

A number of general points can be made about limitations in the evidence base. First, much of the research has been on small samples, often highly selected ones, and there is a need for prospective longitudinal studies, ideally of whole populations, to confirm the risk factors which contribute to language difficulties and to elucidate the nature and progression of these (including their relationship with other problems). Second, there is an urgent need for intervention studies using robust methodologies to identify and explain individual differences in response to intervention. In addition, most research to date has focused on school-age, preadolescent samples and needs extending both to younger groups focusing on ‘at-risk’ signs and to older children and young adults in order to ascertain longer term outcomes. A dearth of research on acquired language disorders in children was also noted.

A related set of issues surround co-occurring conditions (or comorbidities). It was widely held that language difficulties are often associated with behavioural problems but we are only starting to understand the causal relationships [121]. The relationship between language difficulties and autism spectrum disorder on the one hand and intellectual disability on the other hand generated many free text comments from our panellists and it is clear that more and better research is needed. Arguably the same can be said for co-occurring motor disorders and executive impairments (notably working memory problems).

Our panel also made clear that progress would be limited until more reliable and valid assessment methods are developed. The current consensus is that we lack suitable tools for early identification of children at risk of longer-term language impairment: development of methods for distinguishing transient from persistent language problems should be a priority. In addition, further work needs to be done to develop methods for evaluating functional impact of language impairment—that is assessments which go beyond specifying severity in statistical terms. Methods for reliable assessment of pragmatic difficulties are urgently required. There was also interest in identifying developmental trajectories or profiles that would aid differential diagnosis. In this regard, it is timely to make comparisons between different neurodevelopmental disorders [97].

Aside from psychometric concerns, there were two important issues which particularly affect professionals working in education. First, how to conceptualise the relationship between poor socioeconomic circumstances and language impairment and second, how to identify a language impairment in a child whose first (and often home) language is not English. Both of these issues underline the need for multicultural research perspectives in future research and the importance of developing assessments which mirror classroom demands. Moreover, there were cogent arguments for research into the use of dynamic assessment methods which are culture-fair, in particular to inform decisions regarding intervention.

Together these research gaps comprise a significant research agenda. They also underline the fact that language difficulties are heterogeneous and much that has been learnt from studying children with relatively ‘pure’ disorders will need to be modified in the light of recent developments in theory and practice. There is, it was argued, a duty on editorial boards of journals to ensure that samples of children with language difficulties are described fully; we need to know not only what the inclusion and exclusion criteria are, and whether there was any assessment for co-occurring conditions such as speech, attentional or motor problems. We should not restrict research to those with no co-occurring problems, but we do need to know whether they are present and affect language profiles. Development of a standard checklist for reporting participant characteristics could make it easier to compare and combine information across studies.

### Education

Given the complexity of language impairment and co-occurring difficulties such as speech disorders, social impairments and reading disorders, there is an urgent need for better information and training [122]. The panel recognized the need for much greater understanding of typical language development and the extent of normal variation as a framework for identifying children with spoken language needs. Such information needs to reach caregivers, health care practitioners and educators as well as speech and language professionals.

Building on this knowledge, practitioners need to be well informed of the expected levels of performance of children of the age with which they work and to receive support in using tools to identify language impairment and track developmental change. The ultimate aim is to provide classrooms that support good communication for all [123], enhanced provision for those ‘at risk’ and to know when to refer for specialist assessment. A strong clinical interview including history-taking and an assessment of functional impact is important for supplementing language tests.

An important message is that one indication of the severity of language difficulties is poor response to intervention, whether this be direct one-to-one work with a SLT/SLP, attendance in a language enriching educational setting, or indirect intervention via caregivers. While this begs the question of what the intervention should be and how intense, practitioners should be alert to signs of poor progress. Such a strategy would help in ascertaining the nature of the language difficulties of a child with EAL or related disadvantages.

It is clear that there is inadequate knowledge concerning pragmatic language skills and how to assess them. Arguably this can lead to misdiagnosis and confusion with other conditions including psychiatric disorders. There is an urgent need for more skilled practitioners to tackle this under-researched aspect of language impairment from the perspective of assessment and intervention.

Lastly, panel members’ comments (S3 and S6 Docs), revealed concerns about prevailing practice, and issues regarding service delivery. First, the issue of delay versus disorder in language development: although the difference is not supported by research, there appears to be a widely held belief that children with uneven profiles of language impairment are being prioritised for SLT/SLP services over those with 'flat' profiles of impairment. Second, there is a persisting tendency in some circles to think that intervention is not required when language impairments are associated with social disadvantage. Where these misconceptions persist, they need challenging.

Regarding the resources for service delivery, there was concern that increased awareness of language difficulties and better identification might ‘open floodgates’ and that present services could not cope. We would argue that this concern is misplaced. Rather, it is important for greater recognition that language impairment is a public health and education concern, and one that will lead to greater social, medical and educational problems if not addressed. We are at a time when models of service delivery are under scrutiny, with recognition of the importance of prevention as well as treatment [124]. For those with persisting problems it is clearly important to delineate treatment pathways, to ensure correct referrals are made and response to intervention is monitored. Success in this endeavour will require better collaboration between speech and language professionals, those in education, and in mental health services, as well as a commitment to evidence-based policy and practice.

## Supporting Information

S1 Doc Briefing document for Round 1. (PDF)

S2 Doc Preamble to Round 1 survey. (PDF)

S3 Doc Round 1 report. Personalised version of this sent to all respondents, showing overall distribution of responses and qualitative comments.
(PDF)

S4 Doc Round 2 statements, showing relationship with Round 1. Final item number shown in square brackets.
(PDF)

S5 Doc Briefing document sent to panel members with Round 2 items. (PDF)

S6 Doc Round 2 report. Personalised version of this sent to all respondents, showing overall distribution of responses and qualitative comments.
(PDF)
