# Correction: CATALISE: A Multinational and Multidisciplinary Delphi Consensus Study. Identifying Language Impairments in Children

**DOI:** 10.1371/journal.pone.0168066

**Published:** 2016-12-09

**Authors:** The PLOS ONE Staff

## Notice of Republication

This article was republished on September 6, 2016, to correct errors in formatting that were introduced during the typesetting process, as well as correct an error in Table 1. The publisher apologizes for the errors. Please download this article again to view the correct version. The originally published, uncorrected article and the republished, corrected article are provided here for reference.

## Supporting Information

S1 File Originally published, uncorrected article. (PDF)

S2 File Republished, corrected article. (PDF)
