# Supplementary material for: CATALISE: A Multinational and Multidisciplinary Delphi Consensus Study. Identifying Language Impairments in Children
Source: PLoS One. 2016 Jul 8;11(7):e0158753. doi: 10.1371/journal.pone.0158753 (PMC4938414; doi:10.1371/journal.pone.0158753)
Supplement: S1 Doc — (PDF) [file pone.0158753.s001.pdf]

Delphi Panel Briefing Document

CATALISE

Criteria and Terminology Applied to Language Impairments:

Synthesising the Evidence

Dorothy Bishop, Maggie Snowling, Trish Greenhalgh, Paul Thompson

CONTENTS

INTRODUCTION 2

WHAT WE WOULD LIKE YOU TO DO, HOW AND WHEN 3

AUTHORSHIP POLICY 4

BACKGROUND INFORMATION 5

Introduction

This project developed out of discussions by members of the RALLI group for Raising Awareness of Language Learning Impairments (Professor Dorothy Bishop, Professor Maggie Snowling, Professor Courtenay Norbury, Professor Gina Conti-Ramsden and Ms Becky Clark) [1] who, together with Dr Susan Ebbels, argued for the need for an international project to address the confusion in diagnostic terminology around children's language disorders.

Lack of agreement about terminology and diagnostic criteria is recognised as a major problem in the field of children's language disorders . It has held back research and led to wide variations in practice in terms of which children get support. Across the English-speaking world, there is lack of agreement both within and between countries. We plan to run a Delphi consensus process over email to devise an acceptable set of criteria for identifying children with significant language difficulties, and a second exercise to consider appropriate terminology. The Delphi method, in which Prof Greenhalgh has experience, is a process in which a panel of experts is asked to rate a set of statements in an iterative process. The process is conducted over email.

Participants in phase 1 (establishing diagnostic criteria) will consist of researchers and practitioners, starting with those who were involved in writing articles or commentaries for the August 2014 special issue of the International Journal of Language and Communication Disorders, which was devoted to this topic [2]. In addition, we have invited others who have expressed views on an internet forum on this topic hosted by the Royal College of Speech and Language Therapists [3], and asked panel members to suggest additional people to join the panel. For phase 2 (agreeing terminology) we will conduct a second exercise with an extended pool of respondents that will include support groups, family members and adults with language impairments.

In each phase, ratings are collated and results from each iteration fed back anonymously to participants; outliers are invited to offer further comment. The feedback is managed by a facilitator who does not have background in the area (Prof Greenhalgh) to avoid any one interested party controlling the process.

The Delphi method has been used in many areas of social science [4] and there is precedent for it being used to help arrive at agreed diagnostic criteria in medicine [5].

It is anticipated that if reasonable agreement can be obtained by this process, then representative bodies such as the RCSLT, American Speech and Hearing Association and Australian Speech and Hearing Association will adopt the criteria and terminology that are arrived at. We also plan to write a paper explaining the Delphi process and documenting the results.

References

[1] Bishop, D. V. M., Clark, B., Conti-Ramsden, G., Norbury, C. F., & Snowling, M. J. (2012). RALLI: An internet campaign for raising awareness of language learning impairments. Child Language Teaching & Therapy, 28(3), 259-262, doi:10.1177/0265659012459467.

[2] Reilly, S., Bishop, D. V. M., & Tomblin, B. (2014). Terminological debate over language impairment in children: Forward movement and sticking points. International Journal of Language and Communication Disorders, 49(4), 452-462, doi:10.1111/1460-6984.12111.
[3] http://www.rcslt.org/news/news/2014_news_archive/ijlcd_discussion_forum
[4] Hasson, F., Keeney, S., & McKenna, H. (2000). Research guidelines for the Delphi survey technique. Journal of Advanced Nursing, 32(4), 1008-1015, doi:10.1046/j.1365-2648.2000.01567.x.
[5] Lux, A. L., & Osborne, J. P. (2004). A Proposal for Case Definitions and Outcome Measures in Studies of Infantile Spasms and West Syndrome: Consensus Statement of the West Delphi Group. Epilepsia, 45(11), 1416-1428, doi:10.1111/j.0013-9580.2004.02404.x.

What we would like you to do, how and when

The task is to produce consensus statements on criteria for identifying children with significant language difficulties, and terminology to refer to those children. We are inviting you to be a member of our Delphi panel; if you agree, please return the attached form stating your agreement.

A Delphi panel is a way of working towards consensus on a topic or question. It consists of a number of rounds (usually two more), in which you will be asked to do a task which involves *scoring* a draft set of statements. There will be a deadline for this, because we can't analyse the responses until everyone has replied. We anticipate it will take between 30 and 60 minutes to complete the scoring for each round. This is done in your own time and we will give you about 4 weeks to do it.

After each scoring round, you will be sent your own scores *and* the distribution of scores for everyone in the group. If you find you are an 'outlier', you have two choices: amend your score (after reflecting on the statement and why you scored it as you did) – or stand your ground and argue your case to the group (they won't know how you scored the statement). Even if you scored a statement similarly to the group average, you may be swayed to change your score by arguments put subsequently.

Statements are scored on two dimensions: [a] relevance (should we include this topic / theme at all?) and [b] content (should we word it like this?). High scores for relevance *and* content mean the statement will be included 'as is'. High scores for relevance but low scores for content means we need to word the statement differently (we'll ask for suggestions). Low scores for relevance mean the statement gets dropped. But when some panel members score a statement high and others score it low, we need a discussion. For references on the validity and methodology of the Delphi process, please see attached documents.

Here's what we'd like you to do now:

- Read this background paper
- Either complete the form giving your consent, or let us know if you do not wish to take part.

If you would like to take part, you will receive a ROUND 1 email within around 4 weeks. You should then

- Respond to the ROUND 1 email within one month by looking at the statements and entering your scores for each (we'll give you a link to an online questionnaire)
- Wait while we analyse the data and send you back your scores
- Join in an email discussion on how we might amend the statements
- Repeat the last three steps for ROUND 2 (expected in around 4 months time)

Authorship policy

We want to acknowledge the input of everyone who contributes to CATALISE. We propose two levels of authorship:

a. People who contribute materially and significantly to conceptualising the study, undertaking the research, analysing the data or writing up will be named as co-authors on a publication describing the results. The format of the author list will be "Smith A, Jones B, Bloggs D on behalf of the CATALISE group".
b. Members of the Delphi panel who do not fulfil the above criteria will be listed directly below the authors in the following format: "The CATALISE group comprised: Aardvark H, Bloggs D …etc to Zindel B".

Please let us know if you are looking for a formal authorship role or if at any stage you believe you deserve to join the author list. We will also be alert to input from Delphi panel members above and beyond what is expected of an ordinary participant. It is quite possible that a publication based on the CATALISE statement will have a large number of authors and we are comfortable with that.

We regret we cannot pay those who contribute, but please be assured that your input is greatly valued.

Background Information

There is considerable disagreement around the topic of how to identify children who have significant language difficulties that will require specialist input. Furthermore, there is a wide range of terminology used to refer to such children. There is growing recognition that this lack of agreement is damaging; it causes confusion among families with an affected child and among those who work with them, and it hampers research and communication. The problem is international.

In 2012, a small group of UK researchers – Prof Dorothy Bishop, Prof Gina Conti-Ramsden, Prof Courtenay Norbury and Prof Maggie Snowling, together with a speech and language therapist, Ms Becky Clark – formed a group for Raising Awareness of Language Learning Impairments (RALLI). Initially, activities were focused on creating short films for widespread dissemination on the internet. However, another goal of the RALLI group was to tackle the confusion around definitions and terminology for children with language difficulties, and late in 2013, Dorothy Bishop suggested to the editors of the International Journal of Language and Communication Disorders (IJLCD) that there should be a special issue of the journal dedicated to exploring the range of viewpoints on this topic. As it happened, this suggestion coincided with two related developments. First, Professor Sheena Reilly from Melbourne University was giving the IJLCD Winter lecture on 'Childhood Language Disorders'. In her lecture she argued for the abandonment of the current term 'Specific Language Impairment', with its associated narrowly defined diagnostic criteria. In addition, the Royal College of Speech and Language Therapists (RCSLT) had been discussing the possibility of having webinars or other web-based activities around this topic.

It was agreed that the IJLCD would have a special issue of their journal which would feature target papers by Dorothy Bishop and by Sheena Reilly and colleagues, with invited commentaries from a wide range of people covering a range of disciplines and nationalities. In a final paper, Sheena Reilly, Dorothy Bishop and Bruce Tomblin attempted to synthesise the views expressed in commentaries and identify points of agreement. The special issue was edited by Dr Susan Ebbels, and it appeared in August 2014, attracting a very wide readership. All the papers are Open Access and can be downloaded from: http://onlinelibrary.wiley.com/doi/10.1111/jlcd.2014.49.issue-4/issuetoc. Subsequently an internet discussion forum was set up by the RCSLT http://www.rcslt.org/news/news/2014_news_archive/ijlcd_discussion_forum. Soon thereafter, Professor Rhea Paul set up a wiki for interested parties in the USA to share their views: www.wikispaces.com /user/my/ChildLanguageDisorders.

While these activities were useful for scoping the range of viewpoints on this topic, it was difficult to see how to move forward to reach a consensus. We had various discussions about the possibility of having a consensus meeting, but were concerned that it would be difficult to involve all relevant constituencies from all countries. The Delphi process provides a more tractable solution, because it does not require that everyone is in the same place at the same time, it gives people time to respond as they find convenient, and the

anonymisation means that everyone gets a chance to have their views taken into account, without a few forceful personalities dominating. We plan to have a meeting in person in 2016 of at least some of those taking part, plus other interested parties, but this is more likely to be effective if preceded by a Delphi exercise.

The pool of items to be scored in the Delphi process was devised to represent as wide as possible a set of viewpoints. It was developed by reading all the IJLCD articles and commentaries, and the contributions to the RCSLT forum, and writing a new item for each key point that was made. This draft pool of items was then circulated to a small group of interested parties, including RALLI members and Sheena Reilly and Rhea Paul, and some amendments made on the basis of their comments.

Participants in the Delphi include those who wrote articles or commentaries for the IJLCD special issue, plus additional persons who have been invited to ensure comprehensive coverage of different interest groups, professions, national and ethnic groups. It was decided to restrict coverage to English-speaking participants in the first instance, as otherwise the pool of participants would get unmanageably large.
