# Supplementary material for: CATALISE: A Multinational and Multidisciplinary Delphi Consensus Study. Identifying Language Impairments in Children
Source: PLoS One. 2016 Jul 8;11(7):e0158753. doi: 10.1371/journal.pone.0158753 (PMC4938414; doi:10.1371/journal.pone.0158753)
Supplement: S2 Doc — (PDF) [file pone.0158753.s002.pdf]

Supporting Information

Preamble to CATALISE survey items in Round 1

Lack of agreement about terminology and diagnostic criteria is recognised as a major problem in the field of children's language disorders. It has held back research and led to wide variations in practice in terms of which children get support. Across the English-speaking world, there is lack of agreement both within and between countries.

As explained in the briefing document, the aim of the exercise is to seek consensus on how to identify children in need of extra, specialist help with language beyond what is usually available in the classroom, as shown in Tier 3 in Figure 1.

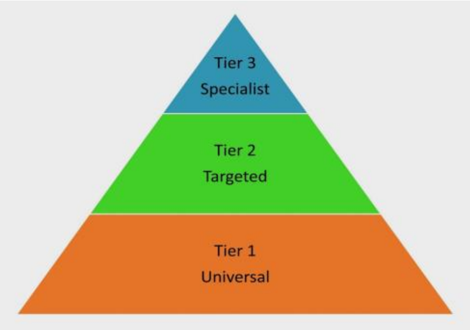

Figure 1. Universal services are designed to optimize communication for all children; targeted services give additional support to those who are vulnerable; specialist services are for those who continue to have significant difficulties despite targeted support. Based on Dockrell, J. et al (2012). Understanding speech, language and communication needs: profiles of need and provision, Better Communication Research Programme. London, UK: Department for Education.

There is ample evidence that children with significant language difficulties persisting beyond preschool are at increased risk of educational failure and social exclusion. There was general agreement among those involved in the IJLCD target articles and commentaries that we need criteria for identifying which children require specialist support.

The focus in this exercise is on how best to identify children with significant language difficulties. In this document we have used the term 'language impairment'. This does not commit us to this term. We will have another Delphi exercise on terminology.

The survey is completed anonymously and takes around 30 minutes to complete. The survey contains 3 general information questions, 46 statements relating to language impairment to rate, and 1 open ended question.
