# Supplementary material for: CATALISE: A Multinational and Multidisciplinary Delphi Consensus Study. Identifying Language Impairments in Children
Source: PLoS One. 2016 Jul 8;11(7):e0158753. doi: 10.1371/journal.pone.0158753 (PMC4938414; doi:10.1371/journal.pone.0158753)
Supplement: S3 Doc — Personalised version of this sent to all respondents, showing overall distribution of responses and qualitative comments. (PDF) [file pone.0158753.s003.pdf]

CATALISE

Criteria and Terminology Applied to Language Impairments:

Synthesising the Evidence

Dorothy Bishop*[1], Maggie Snowling[1], Trish Greenhalgh[2] and Paul Thompson[1]

[1]Department of Experimental Psychology, Tinbergen Building, South Parks Road, Oxford, OX1 3UD, UK.

[2]Nuffield Department of Primary Care Health Sciences, New Radcliffe House, 2nd floor, Walton Street, Jericho, OX2 6NW, UK.

June 2015

Individual Report:

ANONYMOUS

*oscci@psy.ox.ac.uk

Contents

1 Summary **1**

1.1 Participants . . . 1

1.2 Overview of responses . . . 2

2 Delphi analysis results:Your responses relative to rest of panel **3**

2.1 Language impairment as a category . . . 3

2.2 Use of cognitive referencing and delay/disorder distinction . . . 13

2.3 Use of exclusionary criteria . . . 24

2.4 Preschoolers/transient problems . . . 49

2.5 Assessing language difficulties . . . 53

2.6 Breadth of inclusion . . . 79

2.7 Co-occurring problems . . . 87

2.8 Final comment . . . 97

1 Summary

1.1 Participants

A multidisciplinary group of 60 experts from English-speaking countries in Europe, North America and Australasia were recruited to the study. The group comprised eight different diciplines and some combinations of disciplines (Audiology, N=1; Charity, N=4; Educational Psychologist, N=6; Paediatrician, N=3; Psychiatrist, N=; Psychology, Speech and Language Therapist/pathologist (SLP), Specialist teacher, SLP/Ed Psych, SLP/Psych). One member opted out from the panel at the start of round one. Figure 1 shows the breakdown of the group by discipline and country.

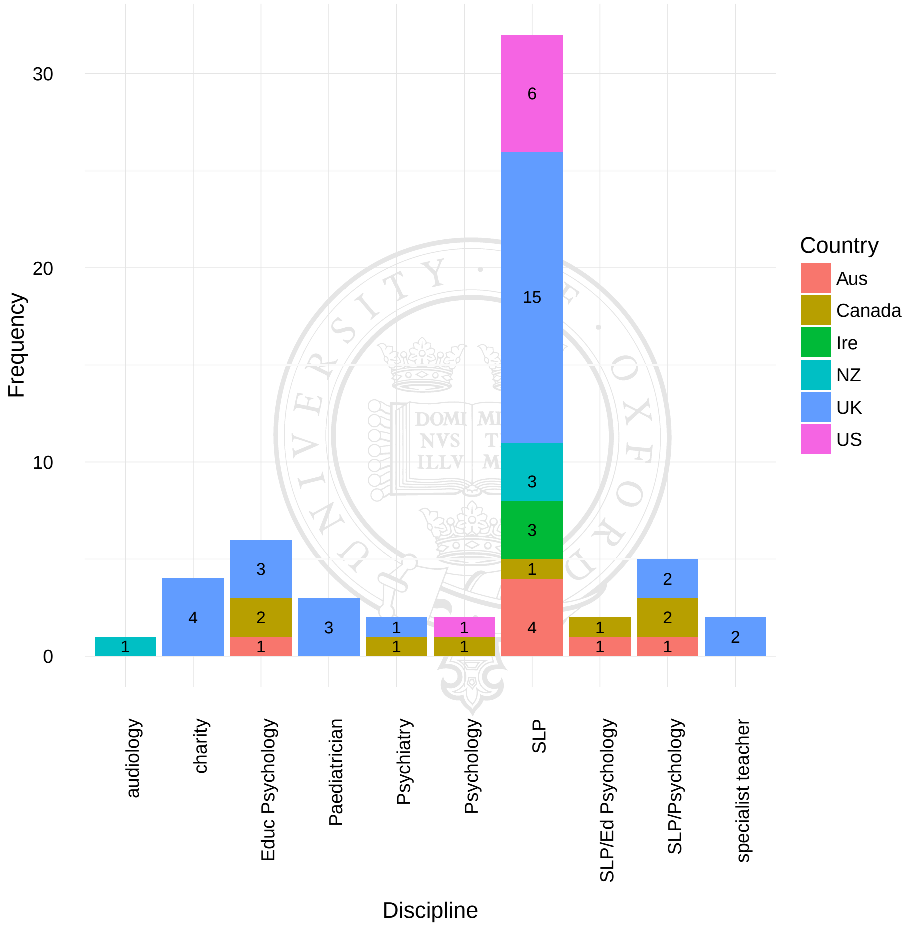

Figure 1: Number of participants summary by Discipline and Country

1.2 Overview of responses

Figure 2 shows an overview of the responses to all 46 statements according to Relevance ('Should we include this topic?') and Validity ('Do you agree with the statement?'). Each bar in the polar histogram represents a specific statements on either Relevance or Valdity and assigns a different colour for each response category in the Likert scale ('Strongly disagree' to 'Strongly Agree'). Within each bar, the percentage responded in each category is represented proportionally as the size of each coloured chunk.

The following section provides a more detailed investigation on an item-by-item basis. Furthermore, we include all the feedback commentary for each item from the panel.

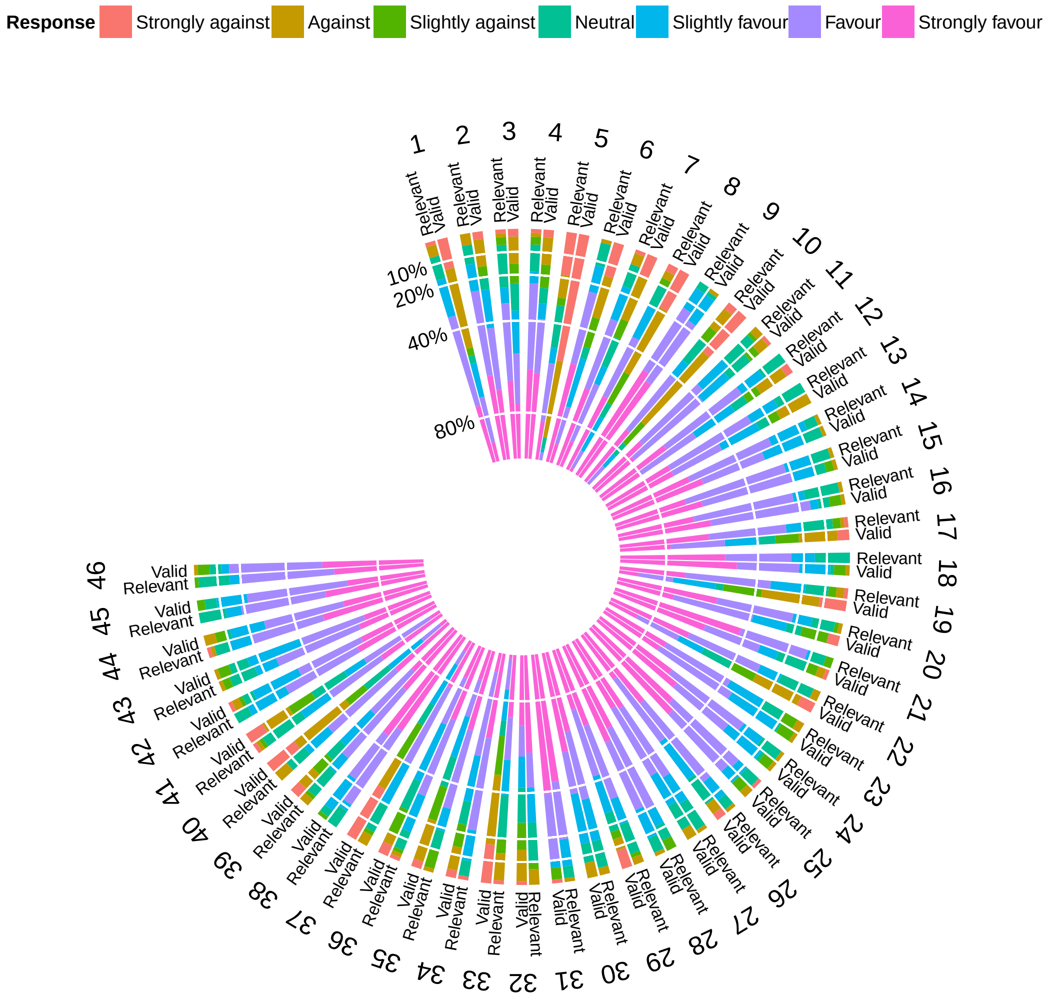

Figure 2: Overview of consensus by statement

2 Delphi analysis results:Your responses relative to rest of panel

2.1 Language impairment as a category

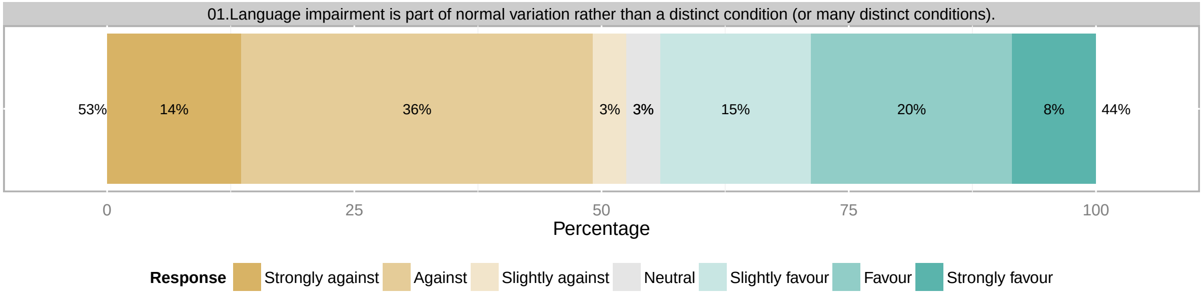

Figure 3: Percentage of panel members in each response category to statement 1. The percentages shown at each end of the scale are the cumulative percentages for the top and bottom three categories respectively.

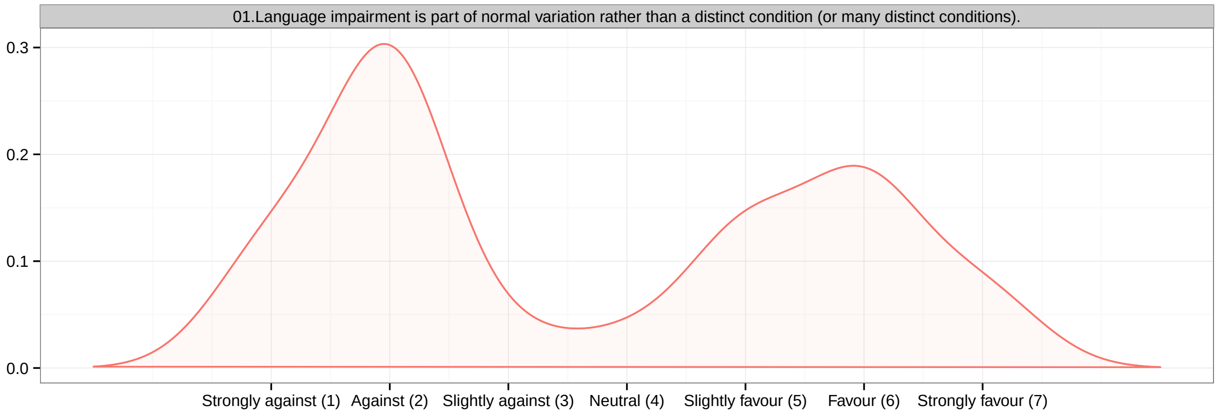

Figure 4: Distribution of responses to statement 1.

Table 1: Comments for each statement.

ResponseID	Q1B
$R_6RlkuyWJYcIIsmN$	The considerable international evidence on SES-influences on language skills in childhood and adolescence poses difficulties in this respect. Though the skills of low-SES children might best be described as reflecting "difference" rather than "disorder", these children are still disadvantaged with respect to meeting the increasingly complex and highly verbal demands of the school curriculum. Also, some children from low-SES backgrounds will experience language impairment on top of their low starting point (and/or some will have identifiable neurodevelopmental disorders). How do we identify such children and meet their needs?
$R_5cKMfR48zQytYc5$	not sure i totally understand the statement if you mean language impairment is one end of the 'language skills' bell curve then yes
$R_bOrkJKVQ6T8FeGp$	This is difficult. I feel that SLI is part of normal variation but that there other syndromes - specific genotype-language phenotype relationships (where the genotype involves multiple gene interactions typically rather than being monogenic. My views on this are relevant throughout this document (e.g., glancing down this is pertitent to question 3 for example) so I shall not continue to repeat this comment throughout.

$R_6LIAgEx6sspizpX$	Questions about the effects of early lanaguage deprivation/disadvantage vs. children who have good early language input but still have significant difficulties. Qualitatively 'feels' different but does the evidence support this?
$R_3sXNbQYRlZaMb3L$	I understand this question in terms of whether language difficulties reflect delayed development or different development, but I'm not sure whether that question is even relevant anymore. I think that many of the traits which make up distinct conditions (eg autism spectrum conditions) exist as normally distributed traits, but that doesn't mean there aren't benefits of identifying a 'condition' beyond a cut-off point.
$R_0Gj2hZlxlaPtHbT$	Normal variation suggests difference without difficulty. / Need to include as topic to discuss thresholds for acceptable variance versus criteria for pathologic condition.
$R_e9cPjWuFpcer4B7$	Strange first question. Should we include it in what? The survey?
$R_9uJ5LinD5e8X5Yh$	Depends what area you are looking at. For speech production easier to evidence a distinct condition applies. For language formulation and comprehension can we defend the use of part of a normal variation? Helpful if we could! Usually the amount of delay (eg %-ile scores on SLT) is the driver.
$R_1TXxdyLg1UFCx4V$	This is a difficult statement since it is likely that language competence is a continuum but there needs to be a clear distinction between a variation in skills and a condition that is significantly impairing
$R_3pDedyU4fM1kOXj$	From both the research available and from my clinical experience I believe that language impairment is a distinct condition. I believe it to be a diagnosis for life. It is a diagnosis which is not part of normal variation.
$R_bwwc7dPFEcp1azH$	If we say that it is part of normal variation that could lead to removal of services for these children
$R_cLU7KRGW2XvEql7$	I don't agree with this statement but discussion my be useful to rule it in/out with reference to evidence
$R_dguQPTfUoDzSKB7$	well this argument has been going on for a very long time, so I agree we should include it
$R_6Dvhy7Alhw5wqIR$	include in what?
$R_2o7JoTNgC3lqSIR$	Sorry, include this topic in what? I'm not quite clear what precisely is being asked here.
$R_7WXquZJy8WlgXAx$	For me this is one of the distinguishing features of language impairment - that it is NOT part of normal variation; it is something different to normal
$R_3rrKtkb2VvC3uG9$	I object to being required to answer a question that is poorly framed for an informed response. This is a case where "language impairment" is too broad a term.
$R_8bIXFrv4VBlvVyZ$	With tests that are sufficiently sensitive, and intensive sampling of children over time (both non LI and LI children) many language behaviours found in children with LI are found in children without. The genetic underpinnings of LI, which won't be shared by all children, preclude it from being entirely part of normal variation; Additionally, at a particular point in time in the developmental trajectory of a child with LI, certain patterns and representations may have become entrenched and unchanging-these in turn may preclude further change/development

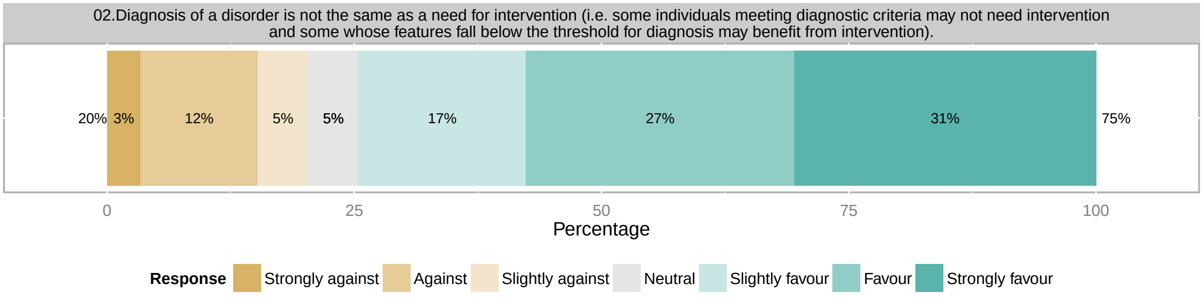

Figure 5: Percentage of panel members in each response category to statement 2. The percentages shown at each end of the scale are the cumulative percentages for the top and bottom three categories respectively.

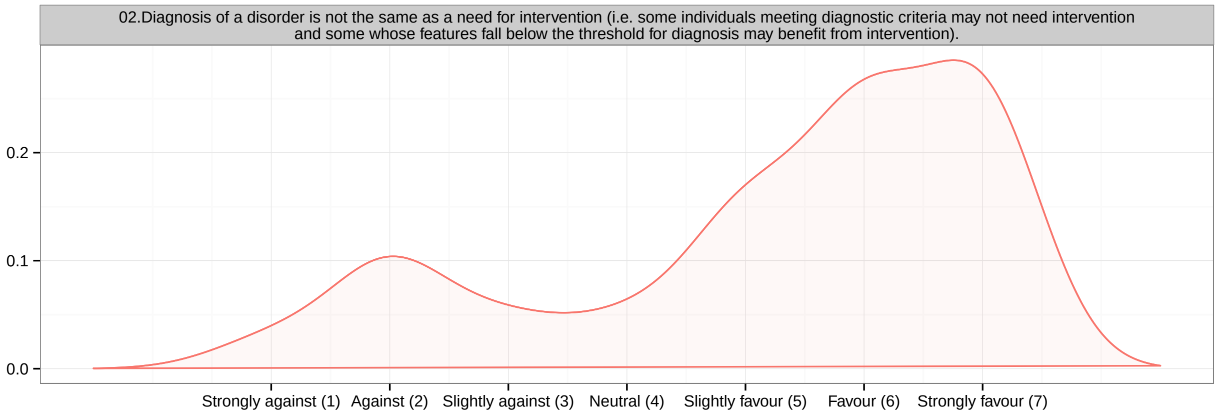

Figure 6: Distribution of responses to statement 2.

Table 2: Comments for each statement.

ResponseID	Q2B
$R_ba8iHG84IJ8cW7X$	It seems to me that 'diagnostic criteria' arise from consensus / professional judgements not objective scientific discontinuities. Thus any definition of when to intervene is disputable.
$R_6RlkuyWJYcIIsmN$	If the aim is diagnostic clarity and having a tighter classification system, I don't think that process should be influenced by a subsequent issue, which is how to determine who needs intervention and when.
$R_5cKMfR48zQytYc5$	assume this is akin to something like blood pressure where you can be on the cusp of having it but not have it? that is in an at risk category????
$R_6JOosydU46ZndMF$	THis is such an important topic & one that is generally not well-understood in the field of medicine and education
$R_bOrkJKVQ6T8FeGp$	This is true. We need to consider the functional impact and environment. Many children in large cohort studies are reported to 'meet diagnostic criterion' at a certain timepoint but a number of such individuals (or specifically their parents) have not sought help and are not concerned about the child's language abilities. These large cohort studies (Community studies) are likely to reflect what is happening out there in the real world.
$R_5cd8BDkYcGfGLKl$	The problem with accepting this is how people decide what children need. I have heard people say that those with the most severe needs do not require intervention because they won't benefit (from what is offered, which isn't much)
$R_e9cPjWuFpcer4B7$	interesting example because the reverse is especially true. Most children who receive services do not receive "diagnoses", in the medical sense of the term, at all.
$R_9uJ5LinD5e8X5Yh$	I have never known a situation where there was no need for intervention if a disorder was described. Not needing is different to not having the resources for!

$R_1TXxdyLg1UFCx4V$	I think the definition of a disorder is that it requires some intervention (see comment above) however in neurodevelopmental disorders there are changes with age and maturity that mean that intervention may be needed at some times and not others e.g. increased need for support at times when demand for skills exceeds ability.
$R_3pDedyU4fM1kOXj$	The diagnosis of a disorder does not indicate or determine the impact- if the disorder has a huge impact on the child then intervention is required. It can be possible for a child to meet the criteria for a diagnosis however their difficulties are supported without needing additional specialist support.
$R_eOEFfbvY55KRtRP$	Need for intervention should be based on broader clinical judgement than diagnostics alone and should take into account holistic considerations such as available support through school/family
$R_cLU7KRGW2XvEql7$	Considering children holistically, some children have environmental or risk factors that ameliorate or exacerbate their difficulties
$R_6Dvhy7Alhw5wqIR$	criteria for a diagnosis of a disorder includes impairment of function across contexts and functions which by implication means intervention–even modification of environment so the parentheses are not really helpful in completing what is a black/white sattement
$R_dmR80BQCC0tAFuZ$	agree with: some whose features fall below the threshold for diagnosis may benefit from intervention but those with disorder should benefit from intervention....
$R_2o7JoTNgC3lqSIR$	The children we deal with certainly need intervention, and often struggle to get it, though admittedly they would generally meet the criteria by anybody's standards. This may not apply to milder cases. What precisely is meant by intervention anyway?
$R_7WXquZJy8WlgXAx$	the need for intervention takes into account a range of factors, one of which is a diagnosis. this doesn't, however, depend on the dsefintion of 'intervention'. if 'intervention' is something above what is available in regular classrooms that have a focus on supporting communication then absolutely it does not imply intervention. this assumes that the child is in a communication supportive classroom
$R_3rrKtkb2VvC3uG9$	I object to being required to answer a questions that is poorly framed for an informed response. An answer requires knowledge of the intervention services to be provided. The opening assumptions for the survey presume a tri-level of services that is not universal.
$R_8AhxnQPe8mJkUoR$	In practice there will be a need to provide guidance as to key features of those individuals who fall below the threshold which warrant providing intervention (and documenting benefit from intervention), as well as features of individuals that meet diagnostic criteria who are not likely to benefit from intervention.

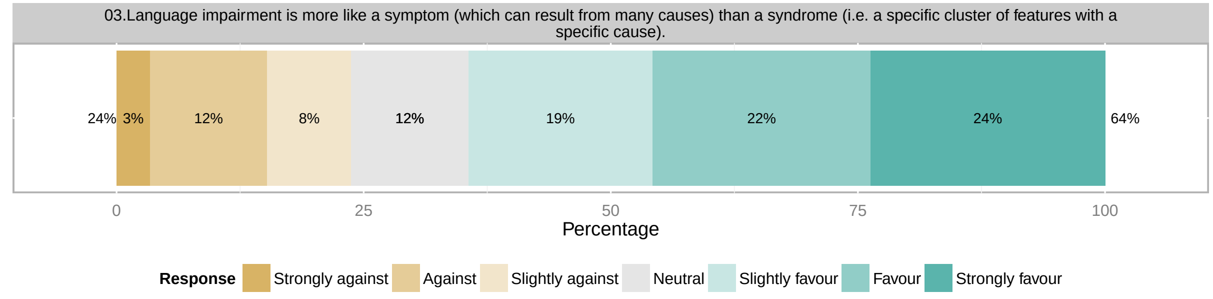

Figure 7: Percentage of panel members in each response category to statement 3. The percentages shown at each end of the scale are the cumulative percentages for the top and bottom three categories respectively.

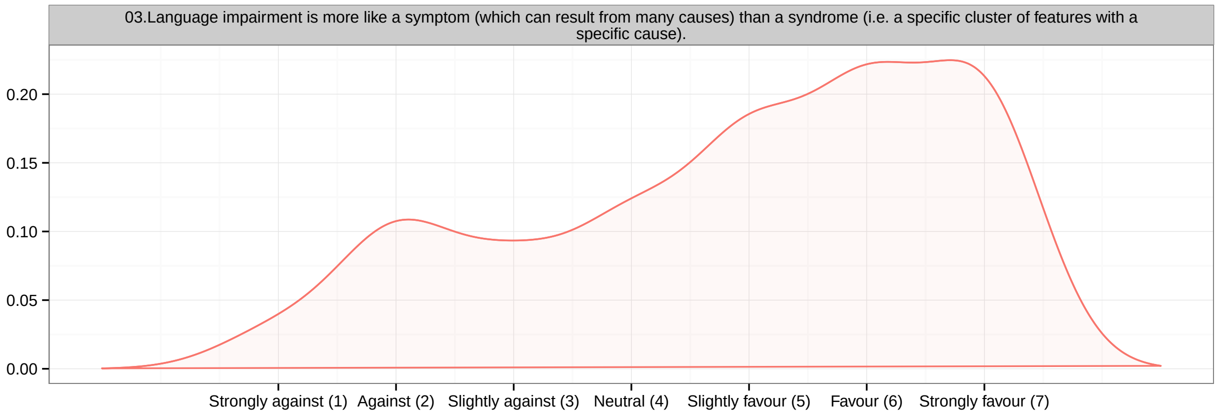

Figure 8: Distribution of responses to statement 3.

Table 3: Comments for each statement.

ResponseID	Q3B
$R_6RlkuyWJYcIIsmN$	I think the term “specific” language impairment has been unhelpful in creating a sense that there are children with “pure” LIs and then all the others. In reality, I think “all the others” are a much larger group.
$R_5cKMfR48zQytYc5$	I think language impairment is a bit like saying you have cancer. Behind that word sits a multitude of different types and individual treatments
$R_5cd8BDkYcGfGLKl$	It is also a feature of many other developmental conditions and this gets lost in service planning at times.
$R_3sXNbQYRlZaMb3L$	The reality in EP practice is that we aren’t preoccupied with causation, other than what it might tell us about effective support and response to intervention. If a child has, for example, social interaction difficulties, they need social skills teaching and modelling, and facilitation of their peer relationships, whether their social interaction difficulties are part of SLI or ASD or anything else. / / The big issue in EP practice is children who have language difficulties in the context of all kinds of other difficulties. They have the same need for intervention and support, but often cannot access speech and language therapy or speech and language specialist education bases etc because their language difficulties may not be specific.
$R_9U2zxMLVAPcvQUd$	I suspect the answer to this question could be both. For some, LI is a symptom resulting from many causes, and for others it is a primary syndrome.
$R_9uJ5LinD5e8X5Yh$	There is a specific cluster of features but not sure we are clever enough to know causes in total yet
$R_6Dvhy7Alhw5wqIR$	a language delay/problem is a symptom–I am afraid the impairment terminology is presenting me with problems! as I interpret it as more specific –ie other causes ruled out

$R_{d}mR80BQCC0tAFuZ$	or can be both......
$R_{7}WXquZJy8WlgXAx$	I am scoring this neutral as I prefer use of the term ‘risk factors’ instead of causes. I do think it should be included but with different terminology
$R_{1}QTm7VrpDX1OAi9$	It may be that there are syndromes within this broad terms and children with an impairment arising from a particular cause are more similar to each other than to other children with impairments arising from other causes
$R_{3}rrKtkb2VvC3uG9$	This is another wonky question that is hard to answer with the overly broad category “language impairment”
$R_{e}G1jl51DiHRqXKB$	I don’t think that this is an either/or issue. I think that both can be true, i.e., a symptom or a specific cluster of features
$R_{8}bIXFrv4VBlvVyZ$	Yes, assessment of components of language impairment across diagnostic groups indicates that there are shared characteristics in features-the differences might be in degree of difficulty with particular aspects; and that underpinning those features of language impairment in the different groups are differences in relative strength and weakness in general v linguistic processing v environmental inputs
$R_{8}AhxnQPe8mJkUoR$	This knowledge may be helpful to both practitioners and parents conceptualization of children’s difficulties.

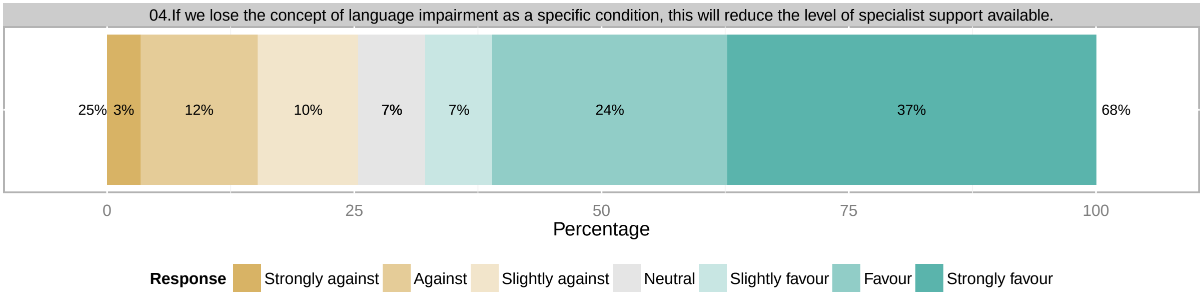

Figure 9: Percentage of panel members in each response category to statement 4. The percentages shown at each end of the scale are the cumulative percentages for the top and bottom three categories respectively.

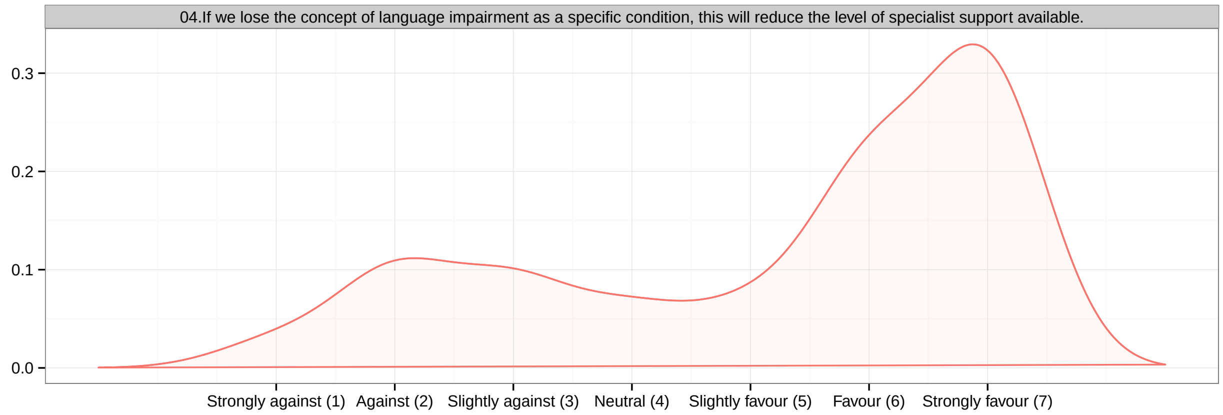

Figure 10: Distribution of responses to statement 4.

Table 4: Comments for each statement.

ResponseID	Q4B
$R_6RlkuyWJYcIIsmN$	Language impairment needs to be positioned as something that affects some children - a bit like hearing or vision impairment. sometimes it occurs on its own, but in many cases it occurs in the context of other disorders. Either way, it needs to be appropriately managed.
$R_6JOosydU46ZndMF$	This would necessitate inservice education to help professionals involved in the support of youngsters with LI to understand the issue
$R_5cd8BDkYcGfGLKl$	As long as we agree that treating language symptoms, that arise for whatever reason, is important for children's academic and social well-being.
$R_3sXNbQYRlZaMb3L$	The key question is whether children identified as having SLI do need support which is different from or additional to that needed by other children with language difficulties. From my understanding working in a local authority, the concept of SLI is used to prioritise children for resources and intervention (and thus exclude other children) but I don't think research supports the idea that these are the only children who would benefit from support, or that the support is particularly different. / / Another major concern as an EP is that the diagnostic label which channels support to children with SLI in their primary years is then used to exclude them from receiving support by the time of secondary transition; I understand that research shows that even children who begin with a 'pure' language impairment (I.e. fitting a discrepancy model - verbal vs. non-verbal skills) will cease to meet the model over time. This is certainly my experience as an EP; by secondary transfer, children are no longer eligible for intensive therapy or for places at speech and language specialists units. NOT because their verbal skills have improved but because their non-verbal skills have declined. Surely the non-responders need more help, not less?!

$R_eLIdYhExxkQtUZn$	Country / region specific issue - level of support given currently varies. Where I live currently, having a diagnosis of 'language impairment' does not impact positively or negatively on service provision. However, in other places I have worked, diagnostic labels have had a strong impact on service access.
$R_e9cPjWuFpcer4B7$	This is a bit of a multi-headed hydra of a question. "Lose" sounds rather careless. If we choose to stop using the term, children will still have speech, language and communication needs. The majority of these children are in schools and educationalists do not tend to use the term "impairment" anyway. It is the need that is paramount not the impairment.
$R_9uJ5LinD5e8X5Yh$	In the current era of SEN even when there is a specific impairment allocated to a child there is a severe difficulty with support. We need to be mindful of political context to ensure children have the access to learning that they need.
$R_1TXxdyLg1UFCx4V$	There should not be a reduction in specialist support if adequate assessment, diagnosis and description of the language impairment leads to well specified needs and plans for intervention
$R_3pDedyU4fM1kOXj$	I fear that children will not be able to access the support they need if we lose the concepts of language impairment as a specific condition- very often to access services a child requires a recognised diagnosis. I am worried that children with language impairments will not be prioritised or provided with the specialist support they require if we lose the concept of language impairment as a specific condition.
$R_es7hPPlfD7bdd65$	I don't feel there is very much support in the first place, so that's why I don't agree more strongly.
$R_4HGIGYFIvMxLWcJ$	This really depends on how children qualify for specialist support. The question is not quite clear - does it mean lose the concept of specific language impairment?
$R_2o7JoTNgC3lqSIR$	We cannot ignore the real world implications. Accessing help for children with SLI can be desperately difficult.
$R_e5KJQmN6txthTRX$	Of course. It's usually impossible to get into a language unit without a 'specific' label etc.
$R_7WXquZJy8WlgXAx$	I think this has been shown through the Better Communication Research Programme - that even WITH a lable, childrenw ith language difficulties get less support than those with other types of need such as ASD
$R_1QTm7VrpDX1OAi9$	I think we need to be very mindful of the possible consequences and anything which will further reduce support should be discouraged
$R_eG1jl51DiHRqXKB$	If anything waters down the need for specialist support that would be tragic. However, there are children who may not meet a standard criterion who, nevertheless, could benefit from specialised support.
$R_8bIXFrv4VBlvVyZ$	Awareness of LI has increased but we are not at a point where the level of awareness is such that the necessary funding and support for intervention and research are as they need to be. There are failures to recognise the nature of and the implications of language impairment at a societal, service and legislative level.

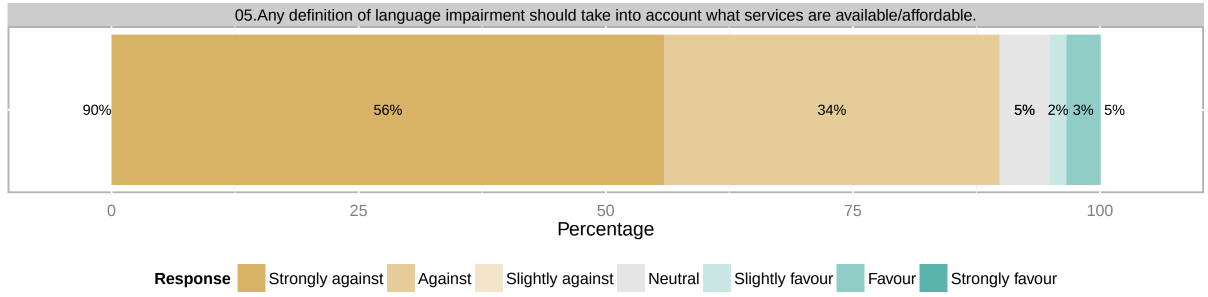

Figure 11: Percentage of panel members in each response category to statement 5. The percentages shown at each end of the scale are the cumulative percentages for the top and bottom three categories respectively.

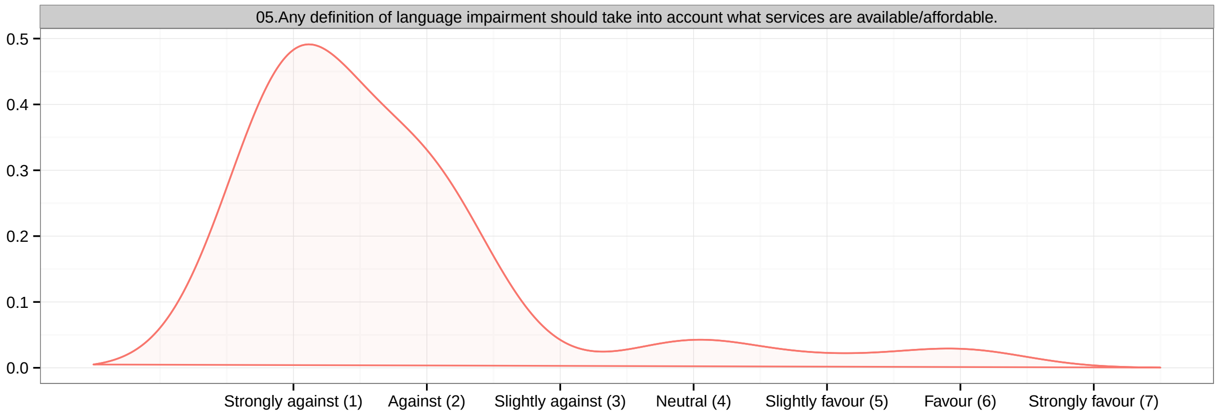

Figure 12: Distribution of responses to statement 5.

Table 5: Comments for each statement.

ResponseID	Q5B
$R_6RlkuyWJYcIIsmN$	Definitions are definitions. Policy makers need to be lobbied to provide adequate services based on identified need.
$R_6JZKVRyNZK6U0zX$	No, as this is bound to vary markedly in different geographical and political contexts.
$R_5cd8BDkYcGfGLKl$	However, we do need to be reasonable. 5% of children is a tractable problem, 50% is one in which people decide that specialist services are not required.
$R_6LIAgEx6sspizpX$	BUT would advocate for sensible use of limited resources we have; we need to get better at properly joined up working across e.g. schools and SLT teams (who does what with whom)
$R_3sXNbQYRlZaMb3L$	Children's difficulties exist whether there are services to meet their needs or not.
$R_e9cPjWuFpcer4B7$	This will inevitably be the case. Prevalence is almost always sensitive to those available to do something about it.
$R_9uJ5LinD5e8X5Yh$	This would be morally wrong and completely lacking in professional integrity.
$R_cLU7KRGW2XvEql7$	this is the road to nowhere (or even hell!)
$R_6mrinfsu6CeSmBn$	If we want our definition to be internationally accepted, then we can't link it to services avaialable as these will vary hugely from place to place.
$R_2o7JoTNgC3lqSIR$	Absolutely not. If children need help, they need help even if it is a struggle to access/provide it.
$R_e5KJQmN6txthTRX$	Children who need support should get the support
$R_7WXquZJy8WlgXAx$	any defintion should not take this into account - we should aim for needs-led provision rather than resource led. the fact that often services aren't available in some areas does not mean they are not needed....and having a specific label for an idenitfied condiftion will provide more room for influecning the providers of those services or policy makers

$R_3rrKtkb2VvC3uG9$	It seems to rule out the need to advocate for broadening services to children not currently served if we define their problems as only valid if services are available/affordable.
$R_eG1jl51DiHRqXKB$	I only agree in the sense that at a practical level some priority may need to be set regarding who actually gets service. In an ideal world services would be broadly available.

2.2 Use of cognitive referencing and delay/disorder distinction

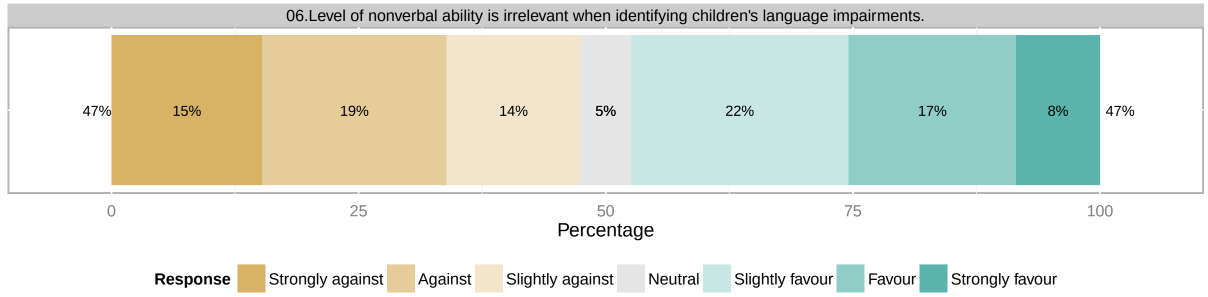

Figure 13: Percentage of panel members in each response category to statement 6. The percentages shown at each end of the scale are the cumulative percentages for the top and bottom three categories respectively.

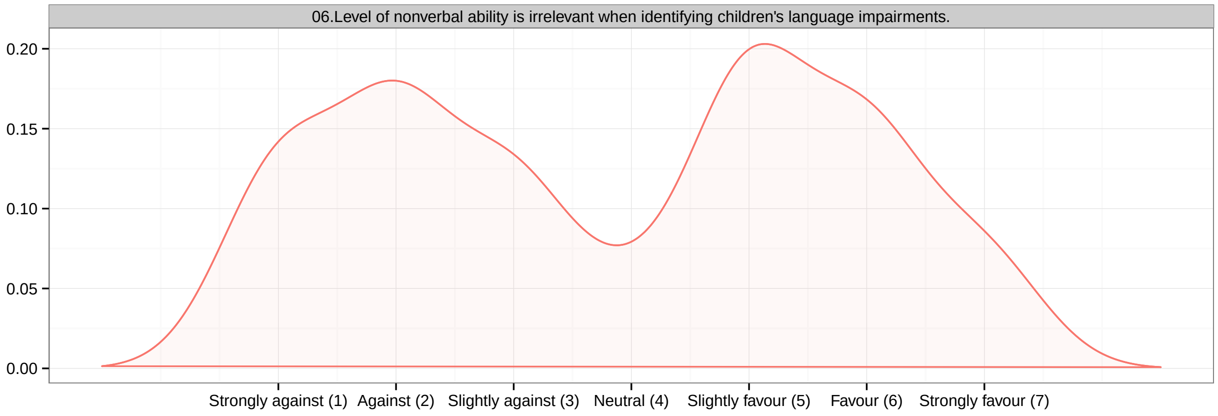

Figure 14: Distribution of responses to statement 6.

Table 6: Comments for each statement.

ResponseID	Q6B
$R_6RlkuyWJYcIIsmN$	It's not completely "irrelevant" (particularly for treatment planning) but I don't think it's useful in a diagnostic sense.
$R_5cKMfR48zQytYc5$	i find it impossible to rate this in isolation
$R_6JOosydU46ZndMF$	A very important topic that necessitates in-depth discussion
$R_6LIAgEx6sspizpX$	This is partly about managing expectations and intervention for children who have severe learning difficulties/cogntive impairments i.e. some children may acquire very limited language because of cognitive levels

$R_3sXNbQYRlZaMb3L$	This is what I would like greater clarity on as an EP. I understand research to be clear that a discrepancy does not differentiate the intervention needed, or the response to intervention, and in any case the discrepancy disappears over time even in children who initially had specific language difficulties. / / The challenge as an EP is that speech and language therapy services and specialist educational provision are both working to this model. My professional judgement is that cognitive assessments are often irrelevant and I feel ethically should not be used in determining eligibility for support and provision, yet everyone is relying on EPs to carry out the cognitive assessments. / / Also, it seems extraordinary that speech and language services are reliant on another professional group (EPs) in order to be able to make any diagnoses or make decisions about whether to offer therapy. As well as being very difficult for SALT services, it puts immense pressure on EPs when they are asked via letters to parents and schools to do cognitive assessments for children who do not meet our own service priorities (e.g. children at high risk of exclusion; children in care). I suspect that we unwittingly deprive children of SALT services because we do not have the capacity to do all these cognitive assessments. Yet I'm equally reluctant to do the assessments, because they are used to deny services (oh, the reason he hasn't responded to therapy is he also has memory/attention/non-verbal difficulties.... I'll withdraw intensive therapy).
$R_e9cPjWuFpcer4B7$	All aspects of the child's development and indeed behaviour need to be taken into consideration. The problem is the arbitrary, and shifting, nature of the thresholds used. These are effectively social constructs which are reified in a pretty arbitrary fashion.
$R_9uJ5LinD5e8X5Yh$	Level of non verbal ability is crucial for consideration of cause, and therefore intervention programmes/pathways. Cognitive and language being at the same level would imply global developmental delay and the type of educational interventions would be differently planned, as would the emotional and social support mechanisms. I could write a book here - but won't!
$R_1TXxdyLg1UFCx4V$	Not irrelevant but not key in defining the disorder. NV ability may be relevant in profiling the individual's strengths and needs and may assist in considering expected outcome of intervention
$R_3pDedyU4fM1kOXj$	I do not believe that there should be a IQ cut-off however I think it is helpful to identify two things; (1) the child's language is following a disordered pattern of development (2) there is a discrepancy between their non-verbals abilities compared to their language levels.... stronger non-verbal skills (however their non-verbal skills do not need to reach a certain number of cut-off)
$R_bwwc7dPFEcp1azH$	the feature of verbal ability should be more of a focus than non-verbal
$R_eOEFfbvY55KRtRP$	In my view is is always relevant. Presence of low non-verbal ability however should not rule out language impairment or the potential to benefit from language therapy.
$R_cLU7KRGW2XvEql7$	how non verbal ability is measured is crucial though, as many test used by educational psychologists are not actually tests of non verbal ability, they just don't require the child to talk! eg, sorting pictures into a sequence to tell a story totally relies on verbal skills, even if you do it silently (yet some EPs don't understand that!)
$R_2o7JoTNgC3lqSIR$	This does depend a bit what this question is asking. Do you mean recognising that there is some sort of language deficit, diagnosing that it is a specific impairment or not, or deciding what support to provide? These are all slightly different questions.
$R_e5KJQmN6txthTRX$	In terms of identifying a specific impairment, it is highly relevant
$R_71b9fvukXBUQ5dr$	Language develops alongside non-verbal ability. Intervention requires understanding of non-verbal ability and other abilities to determine activities and plan episodes of care. It is not an irrelevant factor when identifying LI, although it may not be a relevant influence on LI.
$R_7WXquZJy8WlgXAx$	I think this should be included as it is a contentious and variable issue - particularly in some areas. I think this does provide additional infromation about the specifcity of the impairment, but it is possible to have language impairment in the context of other difficulties. there is also limited evidence that this distinction makes a difference for effective intervention.
$R_1QTm7VrpDX1OAi9$	I think level of nonverbal ability may be relevant for planning the details of intervention, but not whether a child should receive intervention or a diagnosis of language impairment. Therefore, I do not think it should be included in the definition. Some children with low nonverbal IQ have relatively good language abilities, therefore low nonverbal IQ does not mean language must be impaired. Those children with low nonverbal IQ and impaired language should receive services from an SLT
$R_3rrKtkb2VvC3uG9$	another question not answerable in an informed way

$R_3DfMsLnqK54HqcZ$	It is clear that a discrepancy criteria is NOT helpful and SLI versus non-specific LI is not a useful distinction. / For children with severe learning disability (i.e. those with IQs below 70) it would be important for the diagnosis of Learning Disability to take precedence as this is likely to have the greatest impact on the child's prognosis and the educational/intervention approaches adopted. However acknowledging poor language abilities within this group would be helpful in terms of compensatory strategies being adopted in educational provision.
$R_eG1jl51DiHRqXKB$	This is an important topic. For instance, we have found that youth with higher order language problems do more poorly on nonverbal tasks.
$R_8bIXFrv4VBlvVyZ$	Non-verbal ability may provide useful information regarding a child's relative strengths and weaknesses in areas of cognition which support engaging with the environmental inputs that will be essential to their ongoing development (e.g. poor visuospatial abilities might affect event processing and in turn language learning); but non-verbal ability does not determine ability to benefit from intervention; children with low non-verbal ability (e.g. in the case of Down Syndrome) may present with many characteristic features of language impairment in commmon with a child with average non-verbal IQ. The first task in identification is to identify a language impairment, regardless of non verbal ability

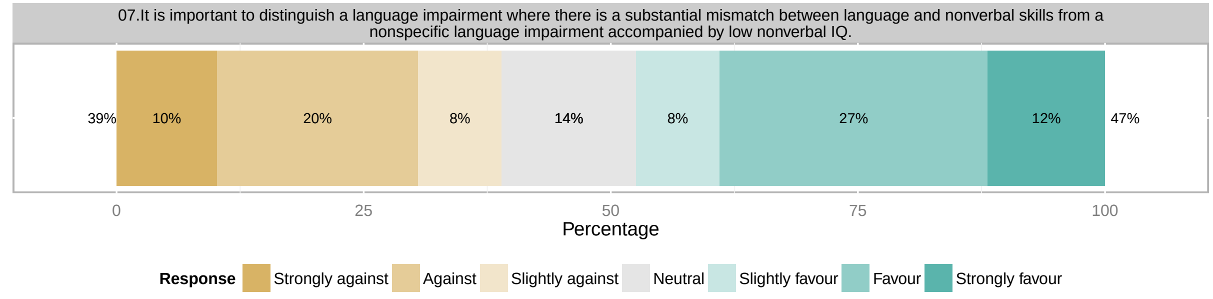

Figure 15: Percentage of panel members in each response category to statement 7. The percentages shown at each end of the scale are the cumulative percentages for the top and bottom three categories respectively.

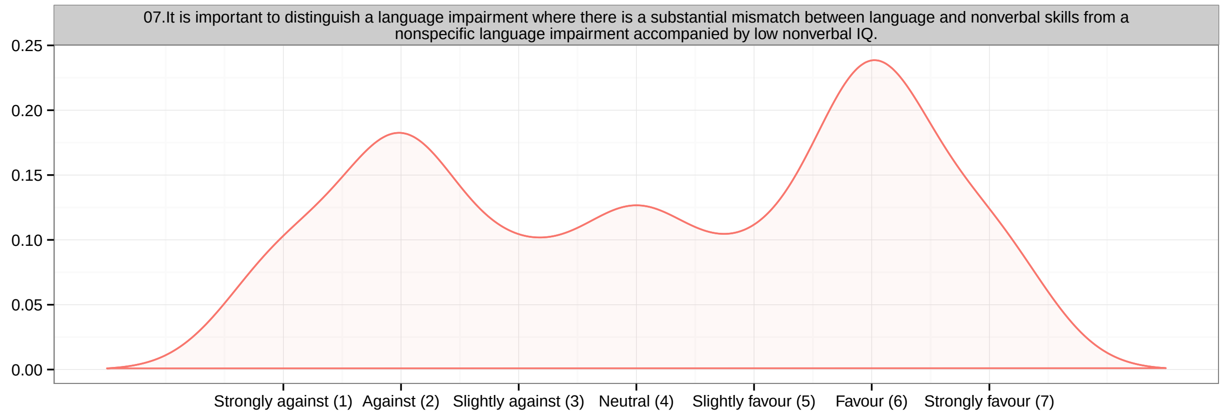

Figure 16: Distribution of responses to statement 7.

Table 7: Comments for each statement.

ResponseID	Q7B
$R_6RlkuyWJYcIIsmN$	It's useful to distinguish these phenomena in order to better understand aetiological pathways and design interventions, but it's equally important to not "favour" the former over the latter (either for intervention services or as the target of research grant applications).
$R_5cKMfR48zQytYc5$	if this is the case then there is an assumption that nonspecific LI does not need attention - again it would depend on what lies behind the language impairment
$R_6JOosydU46ZndMF$	a highly controversial issue that requires in depth discussion and analysis of the research literature - similar to the issue of IQ in the definition of Specific Learning Disoarders
$R_6LIAgEx6sspizpX$	Again , about expectations but also we need to talk about what it means if there is a mismatch. Some clinicians and teachers assume that a mismatch implies that given the right/enough intervention a child will be able to close the gap i.e. non-verbal skills are a measure of verbal potential- in my experience this is not the case.
$R_3sXNbQYRlZaMb3L$	Please see above rant!
$R_e9cPjWuFpcer4B7$	This makes relatively little difference to the child's response to intervention.
$R_9uJ5LinD5e8X5Yh$	See above comments
$R_1TXxdyLg1UFCx4V$	I don't think there is any justification in making a distinction other than to describe an individual's profile when planning and delivering an appropriate intervention. Also it is important to describe profiles fully when comparing research across different individuals with LI
$R_dmR80BQCC0tAFuZ$	with older children, this seems much less relevant (given reduction in NVIQ, possibly caused by lang imp itself, and / or shared cognitive impairment) This can also lead to children being ineligible for interventions which they would benefit from.

$R_4HGIGYFIvMxLWcJ$	I think the issue is that there needs to be a substantial mismatch. As you would not expect average language skills in a child who has a significant cognitive impairment.
$R_6mrinfsu6CeSmBn$	I think there need to be some way of separating the children for whom their langauge impairment is the primary area of difficulty, from those for whom other difficulties are primary, but who also have impaired language development, as I believe care pathways for these groups should be different in order to meet their individual needs.
$R_2o7JoTNgC3lqSIR$	Otherwise, the existence of language impairments per se basically disappears, and all of our families would struggle to access the SLT and other support they can access now - though often only after a lengthy struggle
$R_71b9fvukXBUQ5dr$	As above - may affect setting and education and intervention.
$R_7WXquZJy8WlgXAx$	as previous answer - there is limited evidence that children with specific LI make more progress than those with non specific LI. There are a range of factors that need to be taken into account when planning intervention, and this is one of them. it provides a useful profile for the child's strengths and difficulties, and helps in particular to idenitfy strengths....
$R_1QTm7VrpDX1OAi9$	I feel cognitive referencing should not be used - however, I could be persuaded that this could be useful for those with very low non-verbal IQs where this could enable them to receive a diagnosis of language impairment on top of M/SLD and hence receive services. I do not feel this should be used to exclude children from services
$R_3rrKtkb2VvC3uG9$	Another question with dubious presuppostions; this is a forced contrast
$R_3DfMsLnqK54HqcZ$	As above It is clear that a discrepancy criteria is NOT helpful and SLI versus non-specific LI is not a useful distinction. / For children with severe learning disability (i.e. those with IQs below 70) it would be important for the diagnosis of Learning Disability to take precedence as this is likely to have the greatest impact on the child's prognosis and the educational/intervention approaches adopted. However acknowledging poor language abilities within this group would be helpful in terms of compensatory strategies being adopted in educational provision.
$R_23qAFVuJCo6YHOd$	However, it is important that wider measures of ability/ profiling are needed rather than specific non verbal IQ tests. /
$R_eG1jl51DiHRqXKB$	While the distinction can be described I think that it is worthwhile to discuss nonspecific language impairment especially in light of performance on nonverbal tasks.
$R_8bIXFrv4VBlvVyZ$	I'm not sure that we have sufficient research comparing groups to definitively answer this-for the reasons outlined above, a child may bring different sets of skills to the task of language learning based on their non-verbal ability, which in turn might inform intervention. So knowing there are relative strengths in specific skills may be useful to planning to meet a child's needs

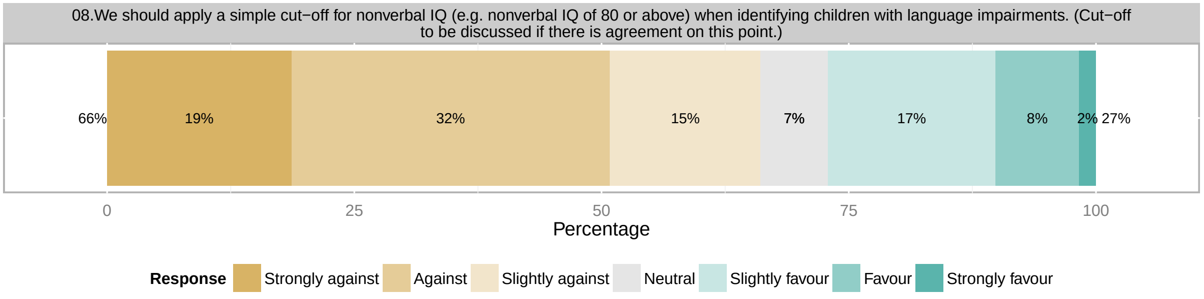

Figure 17: Percentage of panel members in each response category to statement 8. The percentages shown at each end of the scale are the cumulative percentages for the top and bottom three categories respectively.

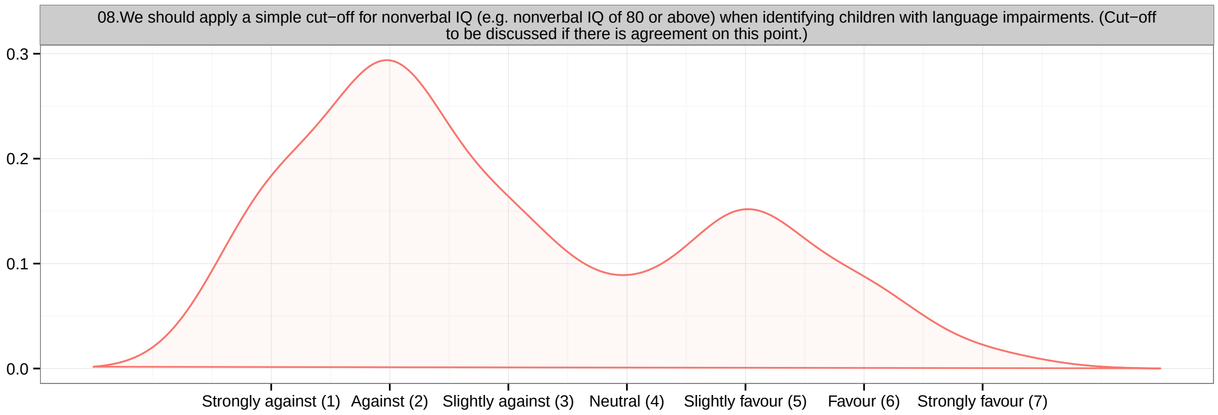

Figure 18: Distribution of responses to statement 8.

Table 8: Comments for each statement.

ResponseID	Q8B
$R_5cKMfR48zQytYc5$	there is no empirical evidence to support this approach
$R_6JOosydU46ZndMF$	The cut-off is often the most controversial issue - needs to take into account the current definition & cut-off for Intellectual Disability & also include measurement error (plus or minus 5 points)
$R_bOrkJKVQ6T8FeGp$	This only applies to SPECIFIC language impairment. I cannot see how this is relevant to all language impairment(s). If we are not talking about children with poor IQ (and genetic syndromes, autism, etc) here then we need to be explicit about this. Apologies if I missed this clarification in the preamble... Otherwise we would be suggesting that children with low IQ cannot have language impairment. Indeed the complexity is in teasing out whether the language impairment is due to deficits in cognitive mechanisms or specific to language only in children where IQ is poor. .... we need to get all of this clear and agreed upon before we can get consensus on anything else.
$R_5cd8BDkYcGfGLKl$	different tests will give different cut-offs and performance is likely to change over time.
$R_3sXNbQYRlZaMb3L$	Please see above
$R_e9cPjWuFpcer4B7$	this is a researchers convenience being extrapolated to services and is inevitably open to threshold bias - ie what is the real difference between two children two points apart either side of the threshold based on assessment by a stranger on a single occasion.
$R_9uJ5LinD5e8X5Yh$	too blunt a criterion. A look at the peaks and troughs of the non verbal scoring is more informative...........where have visual perceptual difficulties, motor coordination problems affected the scoring. Crucially important to know which testing has been used - many EPs a) don't use standardised assts b) use those where language is a component (if not in the batteries then in the delivery)

$R_3pDedyU4fM1kOXj$	I am against using a IQ non-verbal cut off
$R_dguQPTfUoDzSKB7$	I think we meed to describe and continue to examine what the role is in nonverbal IQ and whether the characteristics do differ depending on IQ and syndromes.
$R_6mrinfsu6CeSmBn$	I don't think there will be any evidence to support where the cutoff should be... I would favour a more functional approach (see comment above) although i realise that it woudl be hard to define in objective terms.
$R_2o7JoTNgC3lqSIR$	There probably does need to be an absolute cut-off - and 80 seems reasonable - but really we think the emphasis should be more on the differential between non-verbal and verbal scales.
$R_e5KJQmN6txthTRX$	The level of the cut-off might need to be discussed and the question of borderline children considered
$R_71b9fvukXBUQ5dr$	WHO-ICD 10/11 uses below 70, which gives a useful international classification and identifies children who require intervention across many areas.
$R_7WXquZJy8WlgXAx$	in practice, this has not proved useful. it risks some children who have language impairment not having a useful diagnostic label
$R_1QTm7VrpDX1OAi9$	I disagree with the need for a cut-off. There could be an upper cut-off for those with learning difficulties (e.g., 70) and those children below this cut-off would have a dual diagnosis of learning difficulties and language impairment
$R_3rrKtkb2VvC3uG9$	Again, this question becomes ridiculous under the term "language impairments"
$R_3DfMsLnqK54HqcZ$	As above identifying children with severe learning disability would be important. It is clear that a discrepancy criteria is NOT helpful and SLI versus non-specific LI is not a useful distinction. / For children with severe learning disability (i.e. those with IQs below 70) it would be important for the diagnosis of Learning Disability to take precedence as this is likely to have the greatest impact on the child's prognosis and the educational/intervention approaches adopted. However acknowledging poor language abilities within this group would be helpful in terms of compensatory strategies being adopted in educational provision.
$R_8bIXFrv4VBlvVyZ$	To provide effective services and allow for some degree of specialism to meet specific needs, there is some merit in dimensions of impairment, children with substantially lower non verbal abilities may have needs for a wider set of services than might be just be warranted by virtue of their language impairment alone. But I don't know of any research supporting a particular cut-off, or that we have a consensus on how low the non-verbal IQ can go

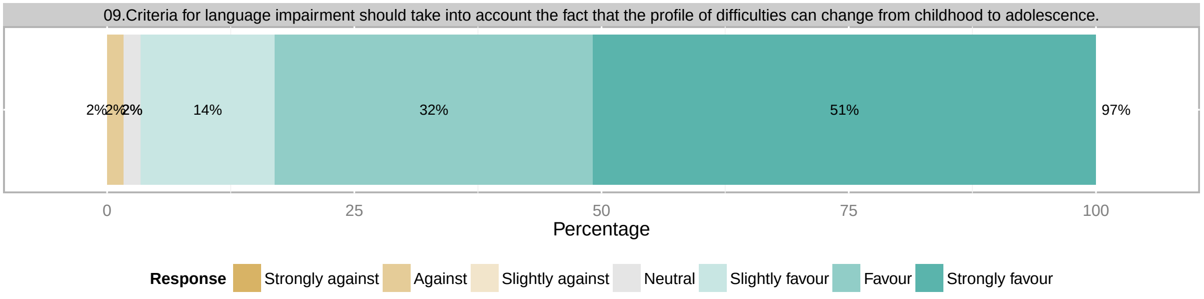

Figure 19: Percentage of panel members in each response category to statement 9. The percentages shown at each end of the scale are the cumulative percentages for the top and bottom three categories respectively.

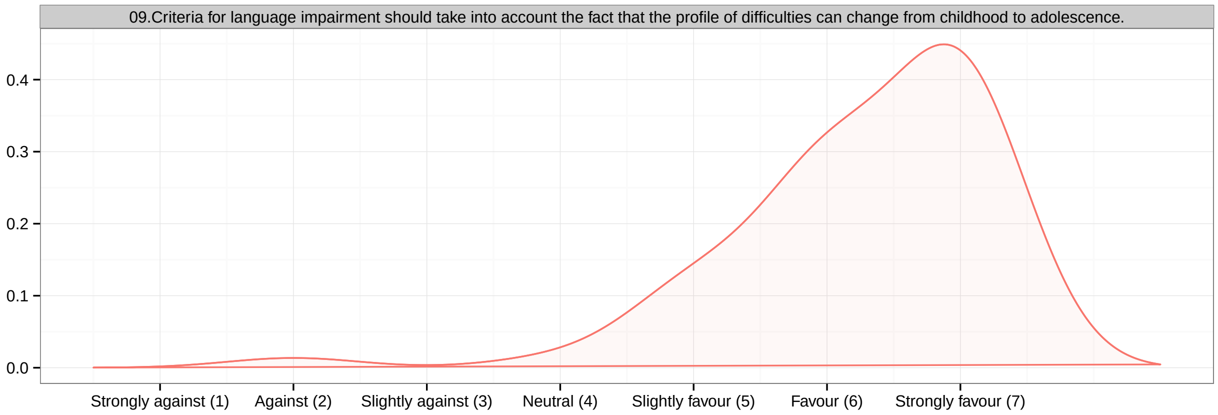

Figure 20: Distribution of responses to statement 9.

Table 9: Comments for each statement.

ResponseID	Q9B
$R_3sXNbQYRlZaMb3L$	Please see previous comments about children becoming ineligible for services because their non-verbal skills profiles change over time. However, I don't think this is an argument for changing the criteria for language impairment, but rather for accepting that non-verbal IQ is not relevant or useful.
$R_e9cPjWuFpcer4B7$	The temporal nature of the construct is critical. the problem is that our capacity to predict in the population as a whole is rather limited.
$R_3pDedyU4fM1kOXj$	research would support this statement
$R_cLU7KRGW2XvEql7$	Also, recognise that some clever children can compensate when they are young so it appears their language is OK, but as they get older this becomes too difficult and they appear to 'suddenly' develop a language difficulty or it manifests as behaviour difficulty and they don't get the help they need
$R_2o7JoTNgC3lqSIR$	I would probably go with this but the question raises a whole number of other issues that could be relevant: / - Does the profile of difficulties always change in the same way? We think probably not, but then can one set of criteria for adolescents cover everyone, or do we say that those meeting certain criteria only would be given the diagnosis? / - If having a diagnosis implies the need for support, should we be restricting the criteria to those likely to need support? / - If the criteria were too broad, would we risk 'medicalising' people who do not need (much) support? / - With no real support available beyond around 16 (and very little between 11- 16) would this raise expectations that could not be delivered, and make people feel hard done-by? / - Perhaps any criteria for older children and adults would need to focus on those in need of substantial support, in order to ensure they get it.

$R_e5KJQmN6txthTRX$	Probably yes, though this then raises a whole raft of other issues, around the support that might be needed, who might deliver it, building up their resilience etc
$R_71b9fvukXBUQ5dr$	Criteria have to be set clearly, in order for such changes in profile to be identified.
$R_7WXquZJy8WlgXAx$	There is good evidence for this and it is extremely important to take into account when planning appropriate support and/or intervention
$R_3rrKtkb2VvC3uG9$	The tricky part here is the term "profile of difficulties can change." "profile" is a strong term; "manifestations" or "symptoms" is more neutral
$R_3DfMsLnqK54HqcZ$	Tackling this issue once a child has received a diagnosis at age (say) 5 and then describing possible pathways after that age with potential co-morbidiites which may emerge, with relative risks would be a very helpful for service planning and potentially feasible to derive from population cohorts. More problematic would be from say 3 years to 5 years where spontaneous resolution is still very likely. A staged approach encompassing "Risk" as well as "caseness" is required here where children who are "pre diagnosis" could access intervention surveillance prior to a persistent impairment being definitively diagnosed. Allowing LI to include children with other associated diagnoses would also be necessary to allow for children presenting late to services with other difficulties and where language difficulties are identified (e.g. reading comprehension problems).
$R_23qAFVuJCo6YHOd$	Experience suggests that most children with language impairment show some pragmatic language difficulties (not secondary to their structural language problems). These appear to become more significant as they get older and can become the primary focus of intervention due to the importance of pragmatic skills.
$R_eG1jl51DiHRqXKB$	Although higher order language skills begin developing from early on they gain increased prominence during adolescence.
$R_8bIXFrv4VBlvVyZ$	Yes, some children may not be identified until later on, for various reasons; the profile of impairment will change and for example present with a more written than verbal impairment as they become older

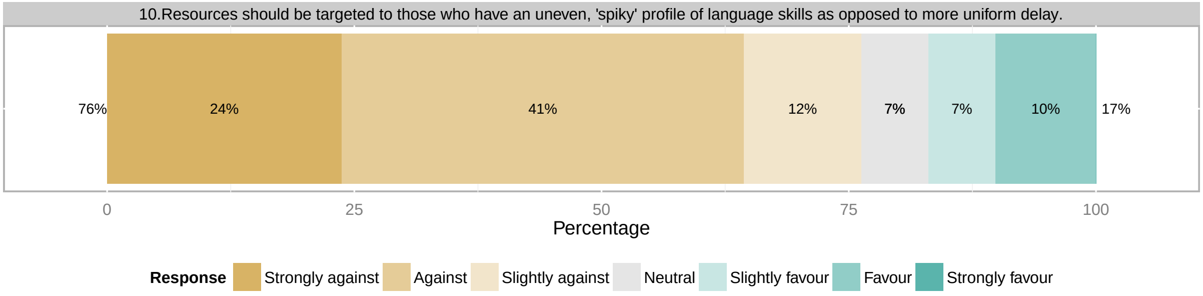

Figure 21: Percentage of panel members in each response category to statement 10. The percentages shown at each end of the scale are the cumulative percentages for the top and bottom three categories respectively.

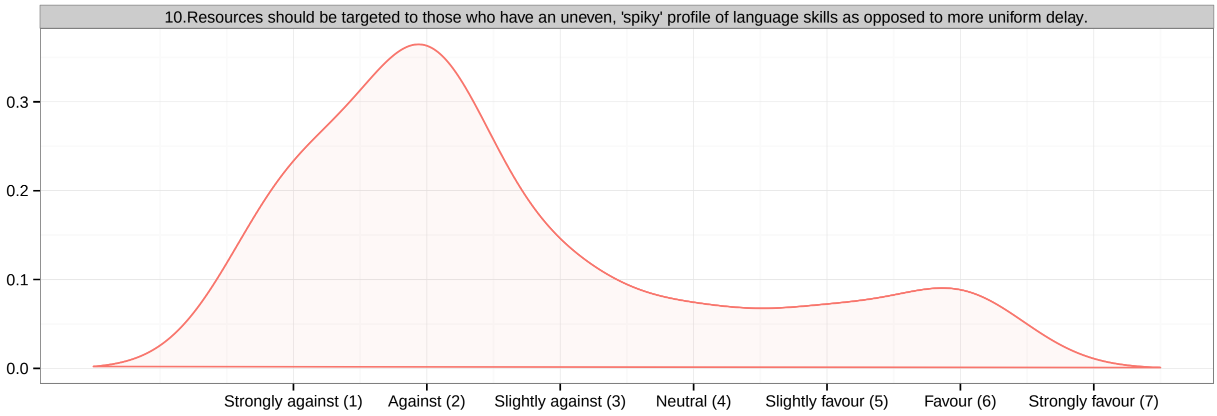

Figure 22: Distribution of responses to statement 10.

Table 10: Comments for each statement.

ResponseID	Q10B
$R_6RlkuyWJYcIIsmN$	Resources should be targeted in such a way as to reach as many children as possible whose language skills (regardless of profile) are judged as "deficient".
$R_5cKMfR48zQytYc5$	can't agree with such a blanket statement - implies we might ration to one group over another - have to consider the impact on the individual despite the profile
$R_6JOosydU46ZndMF$	Why?
$R_5cd8BDkYcGfGLKl$	evidence suggests that those with a flat profile of impairment have greater need over time
$R_6LIAgEx6sspizpX$	Tricky one! Are we assuming that we can close the gaps and bring all skills up to the 'best' level. My experience is that in children's spiky profiles tend to persist after the age of 7/8.
$R_3sXNbQYRlZaMb3L$	Why? This seems to be widely accepted, but I don't understand it. / / Is it because we believe that these children will respond to intervention? As far as I understand, the evidence doesn't support this idea. Even if were true, I don't see how it is ethically tenable. We don't 'target' educational psychology services at those who will conveniently respond to our help. We target them at the most vulnerable children (who by definition, will probably be the hardest to help). / / Is it because we believe that other services will pick up those with 'uniform delay'? These other services don't have specific knowledge and skills in speech and language. / / I fear that this 'targeting' of help reflects something more troubling about who is more and less 'worthy', about the erroneous value we place on 'IQ' in telling us the extent of children's potential, and the value of their lives (and the idea that these two are somehow connected).
$R_0Gj2hZlxlaPtHbT$	to both categories
$R_e9cPjWuFpcer4B7$	"Spikyness" is a truly arbitrary criteria. Everyone is spiky on certain tasks.

$R_9uJ5LinD5e8X5Yh$	All SEN children should have the necessary resources - it is the way these are used that makes the difference eg as a wrap around in the class all day, as a discrete 1:1.
$R_1TXxdyLg1UFCx4V$	Individuals with spiky profiles should not be prioritized over those with a more uniform pattern of strengths and difficulties. The use of the word 'delay' here is misleading. A uniform pattern of strengths and difficulties does not imply that development is delayed rather than disordered. Resources should be targeted at individuals whose progress is impaired by their language difficulties
$R_3pDedyU4fM1kOXj$	I think that children with both types of profiles should have access to resources
$R_bwwc7dPFEcp1azH$	the profile of skills is not relevant, although the fact that very different profiles can present should be acknowledged in the definition
$R_eOEFfbvY55KRtRP$	In my experience some very needy children can have very uniformly depressed profiles and may require as much if not more intervention and support than spikier profiles. The latter group are better placed to find a way to compensate for specific skills lacking.
$R_6mrinfsu6CeSmBn$	We need to be able to distinguish between a 'uniform delay' and a 'uniform disorder'. Chidlren who are uniformly delayed need less resources, but children who are 'uniformly diosrdered' need more than those with spikey profiles.
$R_2o7JoTNgC3lqSIR$	Decisions should be made on the basis of the level of need. If a single area of difficulty is causing very significant problems, this should perhaps be prioritised over a child who is perhaps 'getting by' - even if the overall score profile looks lower. / If however, we are including scores on a psychology assessment, we would tend to favour the child with a spiky profile - which we would interpret to indicate a specific difficulty - over a child with a general learning difficulty.
$R_e5KJQmN6txthTRX$	It does however depend on the individual need
$R_71b9fvukXBUQ5dr$	Not sure what is being profiled, but any aspect of language skill may require resources.
$R_7WXquZJy8WlgXAx$	Intervention should be needs-led, i.e. what that individual child needs.the decision is based on a range of factors, one of which is the profile of needs. this range of factors will chnage as the child gets older
$R_1QTm7VrpDX1OAi9$	Those with a uniform delay are most in need of services as they make the least progress without them. Those with a 'spikey' profile have some strengths they can draw on
$R_1z8h1XMT676UOwd$	Both groups need resources, but they have different need.
$R_3rrKtkb2VvC3uG9$	this is another forced choice. resources should be available to all with language impairments in need of intervention
$R_3DfMsLnqK54HqcZ$	There is no evidence for this but it is still a widely held belief in practice and so needs to be explicitly challenged.
$R_23qAFVuJCo6YHOd$	There are two problems with this statement: / 1) Children with specific language difficulties (without other developmental difficulties) can score low on all subtests of a language test battery. This does not mean they are generally delayed or that they are less deserving of support. If they are scoring low on all subtests, it could be argued that they have more severe and pervasive language difficulties and are likely to be more needing of support. / 2) Whether a child is given support should not depend on their level of general ability, or whether they have more specific difficulties, but on the impact of the difficulties and whether they NEED support. What may differ is the style of delivery depending on the child's profile and the child's response to types of intervention.
$R_eG1jl51DiHRqXKB$	In the best of possible worlds some work would be done with all children with a delay to see whether an impact on development is possible. In the real world the 'spiky' profile group is more likely to receive services.
$R_8bIXFrv4VBlvVyZ$	No there are children with severe language impairment affecting all components of language - phonological, semantic, morpho-syntactic and who need & will benefit from resources. I'm not sure that the notion of "spiky" profiles prevails in people's thinking as it might have done a number of years back, when our then understanding of language impairment was at least in part based on research that had looked at mid-school age/older children with developed systems-(some of whom had entrenched difficulties in particular areas based on inherited grammatical v non-word impairments) or based on sub-typing exercises.

2.3 Use of exclusionary criteria

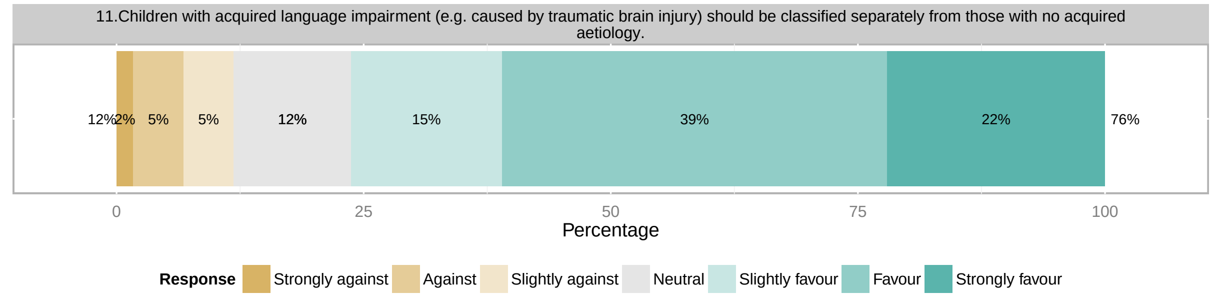

Figure 23: Percentage of panel members in each response category to statement 11. The percentages shown at each end of the scale are the cumulative percentages for the top and bottom three categories respectively.

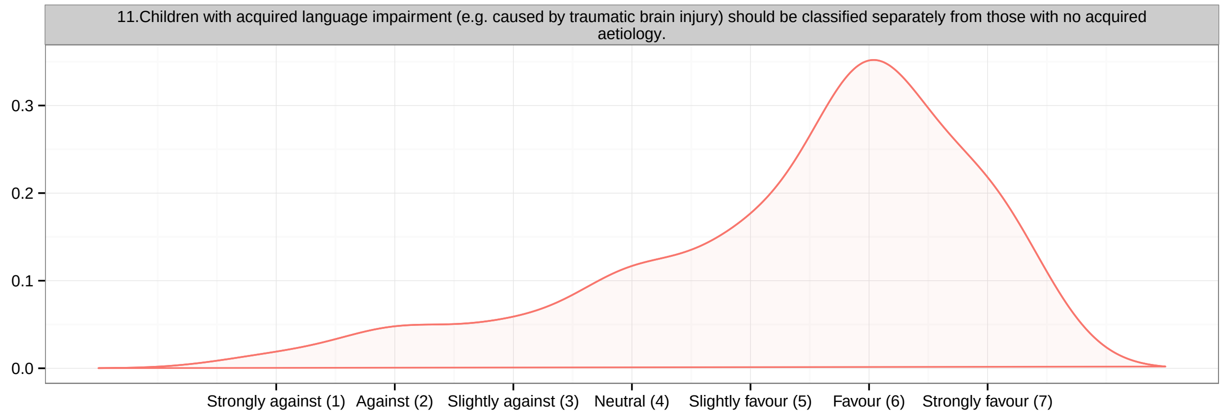

Figure 24: Distribution of responses to statement 11.

Table 11: Comments for each statement.

ResponseID	Q11B
$R_2f9ctxaHBJuJdLD$	Important to recognise organic aetiology
$R_6RlkuyWJYcIIsmN$	Traumatic brain injury can have serious implications for ongoing language development, and social and academic success. However because it reflects an identifiable event and aetiology, I don't think it's useful to include it here.
$R_5cKMfR48zQytYc5$	so if you have a clearly identified aetiology you can't have language impairment? / I think a classification is required that takes into account what is know about aetiology etc
$R_6JOosydU46ZndMF$	I am insufficiently familiar with the literature on this specific topic to give an opinion
$R_6LIAgEx6sspizpX$	I don't feel I know enough about ABI to say.
$R_3sXNbQYRlZaMb3L$	Again, as a pragmatist practitioner, I would find this helpful if it was because different support is needed by each group.
$R_9U2zxMLVAPcvQUd$	with the qualifier that we are talking about "those with no known acquired aetiology"
$R_e9cPjWuFpcer4B7$	Again an odd question. Classified by whom? Their access to services is likely to be completely different from those with developmental problems - certainly in the younger age groups. So they are unlikely to be classified by the same people. By contrast their needs can readily be placed on a similar scale.
$R_9uJ5LinD5e8X5Yh$	Profile matching should reveal clusters of need and input rather than working from causes. The latter are interesting and add to the knowledge/package, but presentation of need is the critical information to inform intervention.

$R_1TXxdyLg1UFCx4V$	This depends on the purpose of the classification. Aetiology is important for research but it should not be used to 'cordon off' clinical services since each individual needs a full assessment and intervention targeted at their current needs. Aetiology is also important in influencing expectations of outcomes following intervention.
$R_bwwc7dPFEcp1azH$	as the causation/prognosis is different for this group, the classification should be separate
$R_6mrinfsu6CeSmBn$	They may however need the same care pathway....
$R_2o7JoTNgC3lqSIR$	It is likely that if the acquired impairment happens very early in life, while the child is a baby or toddler, it might make no difference to the help the child needs, but we don't know enough to comment about older children.
$R_e5KJQmN6txthTRX$	Not enough knowledge about older children with acquired impairment though in the early years there may be no difference between them and children with a developmental difficulty. If children need help, they should get it
$R_71b9fvukXBUQ5dr$	WHO again - although interventions may be similar, other concomitant factors could affect intervention - e.g. recovery rates.
$R_7WXquZJy8WlgXAx$	the language impairment should refer to the profile of needs, not the cause. there may be language impairment associated with the acquired condition
$R_1z8h1XMT676UOwd$	Acquired versus developmental language impairment should be identified separately, but it should be recognised that the result is the same, i.e., specific language impairment (deficit in language compared to nonverbal cognitive abilities) can be later onset and associated with a specific event such as TBI. The current common definition of SLI is problematic in this respect I think.
$R_3rrKtkb2VvC3uG9$	again, the term "language impairment" is too broad for an informed answer
$R_3DfMsLnqK54HqcZ$	This developmental vs acquired distinction is important in terms of prognosis and nature of impairments and should be retained
$R_eG1jl51DiHRqXKB$	I don't know the comparative literature on this topic so don't have an educated opinion.
$R_8bIXFrv4VBlvVyZ$	At least based on my current knowledge, these children have needs children whose impairment is developmental rather than acquired would not have

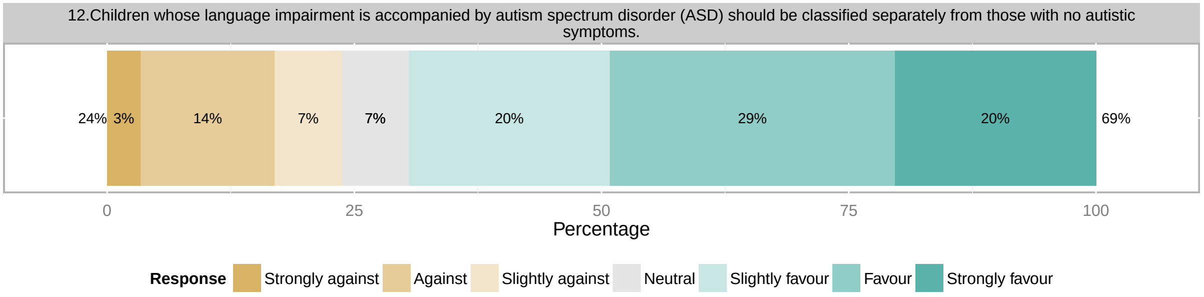

Figure 25: Percentage of panel members in each response category to statement 12. The percentages shown at each end of the scale are the cumulative percentages for the top and bottom three categories respectively.

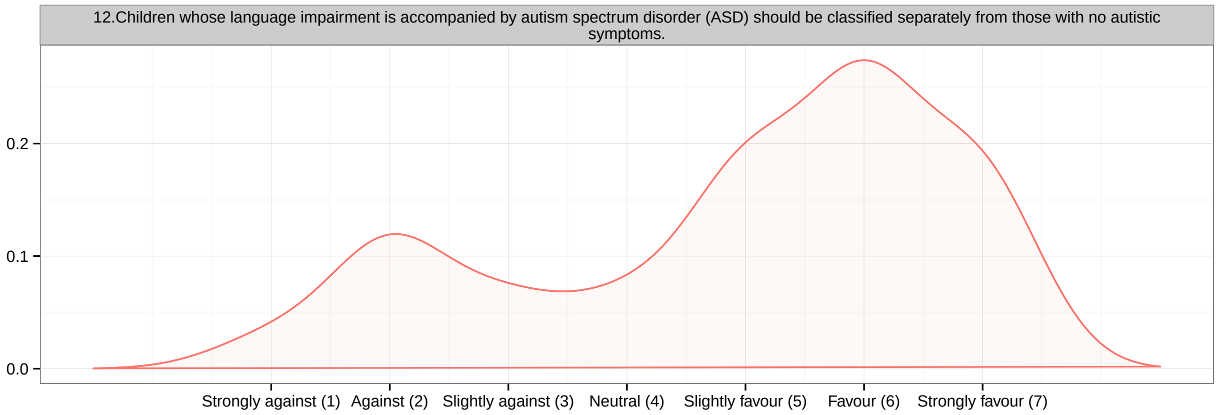

Figure 26: Distribution of responses to statement 12.

Table 12: Comments for each statement.

ResponseID	Q12B
$R_2f9ctxaHBJuJdLD$	Both can cooexist and the relative weighting may change with time
$R_6RlkuyWJYcIIsmN$	The diagnostic boundaries between ASD and LI are permeable and changeable.
$R_5cKMfR48zQytYc5$	slightly agree because of different needs
$R_6JOosydU46ZndMF$	I think this is a criticL TOPIC FOR DISCUSSION. TO ME THE KEY ISSUE IS TO ENSURE THAT THE COMORBID CONDITION IS SPECIFIED BECAUSE OF IMPLICATIONS FOR INTERVENTION
$R_5cd8BDkYcGfGLKl$	I do think children with ASD may have additional learning, language and therapy needs. However, it is important to stress that many children with ASD have language impairments (not just social skill deficits) that require attention and remediation.
$R_3sXNbQYRlZaMb3L$	It is helpful to know about the child's other difficulties because those other needs must also be met, but beyond this I'm not sure.
$R_e9cPjWuFpcer4B7$	Diagnostically professionals an psychologists in particularly often prefer to split the population up into ever smaller groups. From an intervention perspective these children have much in common.
$R_9uJ5LinD5e8X5Yh$	There is a huge need for the professionals to understand the root of the language impairment in this situation. ASD gives rise to pragmatic language difficulties and an ASD learning style - both need to be addressed. Some children have this profile plus a specific language impairment - this is complex and needs skilled interventions to address all 3 areas. My 2nd book will be about this! Please please can professionals look at ASD learning (outside that of speech language and communication) - it is not just about the language of the ASD learner, it is as much about the style with which they learn everything. SLTs step away and let the educationalists have a say!

$R_1TXxdyLg1UFCx4V$	See comments relating to the previous statement. Over categorization of types of language impairment leads clinicians to feel they do not have the expertise to work with a range of language impairments and allows services to limit the demands on their service by excluding groups
$R_bwwc7dPFEcp1azH$	Although they are separate, they may have overlapping features which should be acknowledged
$R_6Dvhy7Alhw5wqIR$	waht matters is skilled assessment of what is due to autism and what to language impairment
$R_2o7JoTNgC3lqSIR$	This is however easier said than done. Many of the cases Afasic deals with, are children in the 'grey area' between the two.
$R_71b9fvukXBUQ5dr$	Classification is of disease - descriptions of language skills are separate.
$R_7WXquZJy8WlgXAx$	the key wording here is 'accompanied by'. if the child has language impairment as well ASD then that is how it should be described. this should be different to the language and communication difficulties associated with ASD
$R_1z8h1XMT676UOwd$	Intervention approach will be different.
$R_3rrKtkb2VvC3uG9$	I believe I may have the same response to many of these queries
$R_3DfMsLnqK54HqcZ$	For research purposes it may be important to exclude these groups e.g. in genetic or neuro-imaging research - or to make comparisons between these groups and others with LI. For clinical and educational purposes recognising LI within the context of other diagnosis would be helpful in ensuring the increased risk of LI in ASD is widely recognised and addressed through interventions. But also to recognise that some children with ASD have relative strengths in structural language. Clearly a diagnosis of ASD brings with it broader needs than those associated with LI depending on the individual's profile of strengths and needs (in a similar way to those with a learning disability). A recognition of LI and ASD as umbrella terms within which subgroups exist and where overlap occurs would be helpful. Also it would be important for there to be a clear recognition that ASD and LI are descriptive diagnosis based on surface symptoms with many potential underlying causes. Indeed a further umbrella term of Learning Disability would be needed with recognition of overlap.
$R_23qAFVuJCo6YHOd$	I think it should be possible for a child to have 'ASD' and 'SLI'.
$R_eG1jl51DiHRqXKB$	I don't feel that I have enough knowledge about the language of autistic children to rate this item.
$R_8bIXFrv4VBlvVyZ$	For the purposes of identification and diagnoses; this may serve to direct children to more specialised services based on the wider dimensions of their ASD; for service delivery and clinical practice purposes therapists/educators might also require additional skills/knowledge to work with children with ASD & be directed to relevant evidence, literature and CPD to meet those needs.

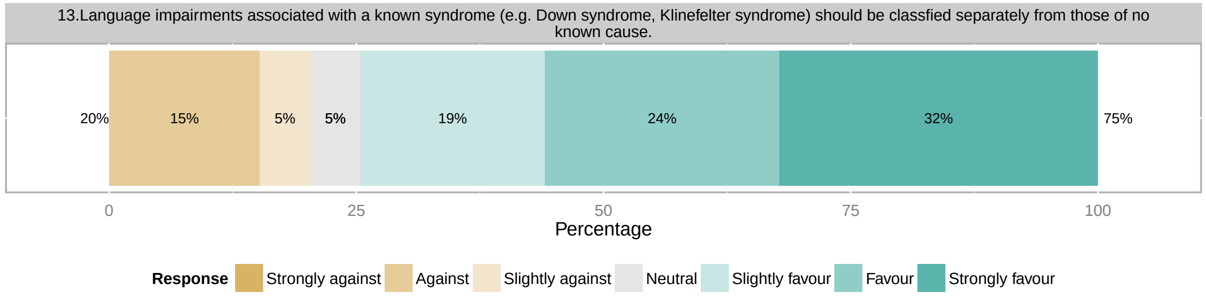

Figure 27: Percentage of panel members in each response category to statement 13. The percentages shown at each end of the scale are the cumulative percentages for the top and bottom three categories respectively.

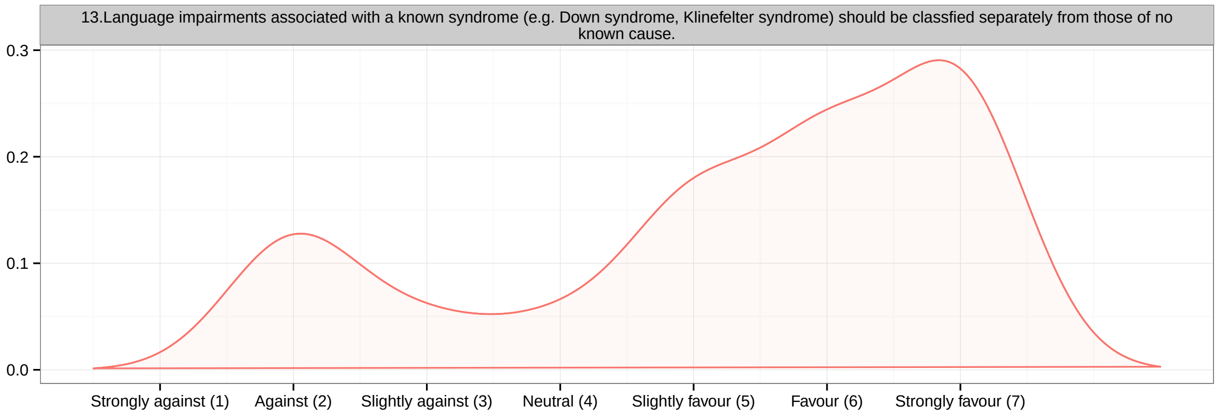

Figure 28: Distribution of responses to statement 13.

Table 13: Comments for each statement.

ResponseID	Q13B
$R_2f9ctxaHBJuJdLD$	It would depend on how unique their presentation and could it occur without the other condition
$R_6RlkuyWJYcIIsmN$	Where there is an identifiable syndrome, it might be easier to describe the typical language profile associated with that condition. However a suitable term (eg LI) will still need to be agreed upon for such populations. Also, many children have non-syndromal genetic disorders and have language problems - so once again, we need to avoid inadvertently creating a “hierarchy” of children as a function of their aetiological pathways.
$R_5cKMfR48zQytYc5$	as per comments in question 11
$R_6JOosydU46ZndMF$	the issue is whether these groups of individuals differ in clinically meaningful ways from LI in the absence of these syndromes: especially in terms of response to intervention - is there a difference?
$R_bOrkJKVQ6T8FeGp$	Terrific - questions 12 and 13 are getting at the issues I raised earlier.
$R_5cd8BDkYcGfGLKl$	similar to comments about ASD. These children should not be denied SLT services just because they have a known diagnosis.
$R_6LIAgEx6sspizpX$	Similar to discussions about cognitive impairements/LD and language.
$R_3sXNbQYRlZaMb3L$	See above.
$R_e9cPjWuFpcer4B7$	Again the needs may well be pretty similar. Of course both of these children have much clearer biological markers and can be distinguished on the basis of these but would they really be treated in a very different way?
$R_9uJ5LinD5e8X5Yh$	A label is a handy shortcut to much lengthy description of the whole child, but as said before - it is the presentation of the language difficulties which should drive interventions............... no matter what the syndrome/label.

$R_1TXxdyLg1UFCx4V$	As above
$R_bwwc7dPFEcp1azH$	The features of language impairment will overlap with language impairment, but the prognosis, causation is different as is the current evidence base for interventions - I would argue that children with known syndromes do not get as much intervention or access to specialist services as those with language impairment because of the perception that their nonverbal IQ is lower, thus their potential is lower which is wrong
$R_6mrinfsu6CeSmBn$	They may however need the same care pathway....
$R_2o7JoTNgC3lqSIR$	It is really our view that SLI is a condition of its own. The risk of saying there is no distinction is that, in the real world, people assume language impairment always results from some other condition, and if none can be identified, that there is no language impairment, or if there is, that it doesn't need to be taken seriously and is attributed simply to poor parenting etc
$R_e5KJQmN6txthTRX$	Unless they a have a specific language impairment as well
$R_71b9fvukXBUQ5dr$	My model is of language impairment co-occuring with other disabling conditions, or not, and existing clinical classification being useful.
$R_7WXquZJy8WlgXAx$	the term here again influences my rating. if the wording was 'accomapnied by' I would diagree. language difficulties asscoaited with Downs Syndrome do not necesarily mean a language impairmenrt
$R_1z8h1XMT676UOwd$	Intervention approach will be different.
$R_3rrKtkb2VvC3uG9$	Another forced choice
$R_3DfMsLnqK54HqcZ$	For research purposes it may be important to exclude these groups e.g. in genetic or neuro-imaging research - or to make comparisons between these groups and others - and/or in other studies depending on the research questions being asked. However, for clinical and educational purposes recognising LI within the context of other diagnosis would be helpful in ensuring the increased risk of LI is more widely recognised (e.g. in ADHD) and addressed through interventions. In the same way, however using LI and not acknowledging the other diagnosis would be unhelpful so both would need to be acknowledged. For educational and intervention purposes diagnoses based on the nature of an individual's strengths and weaknesses and the functional impact of the impairment over aetiological diagnoses (and so using diagnoses of LI, ASD, Learning Disability, EBD, Reading Disorders), and acknowledging overlap, may be more informative for intervention than diagnoses based on the underlying aetiology (where this is known). / / Clearly families should still be given the 'aetiological' diagnoses where these are known to learn from previous research about this group of children in terms of prognosis etc. But the more descriptive groupings based on dimensions of strengths and weaknesses of key domains (Cognition, Structural Language, Social Cognition, Pragmatics, Attention, Social and Emotional Adjustment, Literacy, Imagination/Flexibility/Interests) should drive educational and intervention choices.
$R_8bIXFrv4VBlvVyZ$	As with the statement above

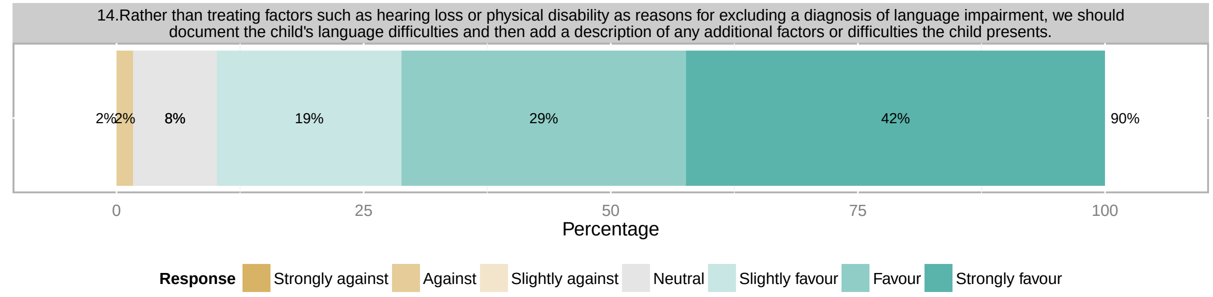

Figure 29: Percentage of panel members in each response category to statement 14. The percentages shown at each end of the scale are the cumulative percentages for the top and bottom three categories respectively.

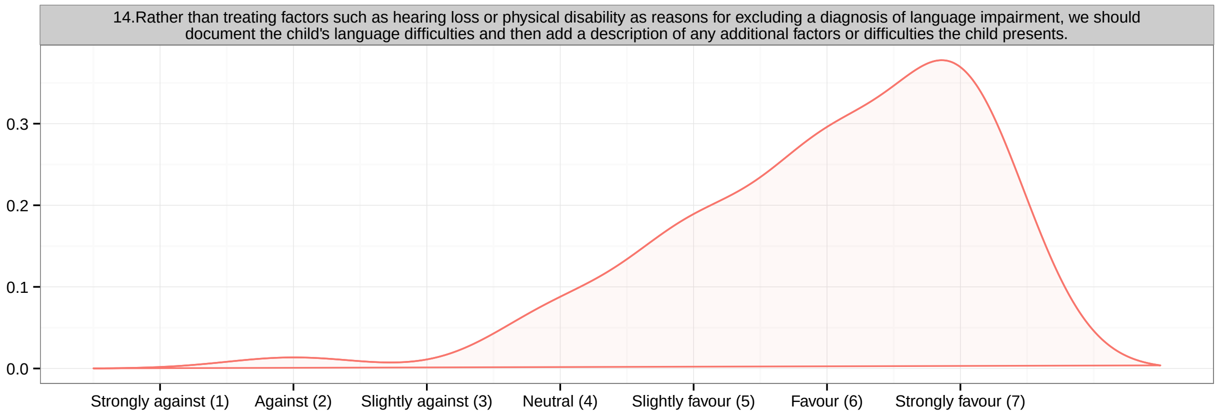

Figure 30: Distribution of responses to statement 14.

Table 14: Comments for each statement.

ResponseID	Q14B
$R_6RlkuyWJYcIIsmN$	Language impairments are common and commonly occur alongside other disorders. We need a diagnostic/classificatory system that accommodates, rather than fights this.
$R_6JOosydU46ZndMF$	To me the key issue is the implication for intervention - hence the reason to specify any other co-occurrng problems
$R_e9cPjWuFpcer4B7$	Yes I would say that this is the correct solution. there may be cases where the priorities are completely different depending on the nature of the condition. For example if a child was completely deaf the priority might be the introduction of or support for sign language, but from my perspective these are the exceptions that prove the rule
$R_9uJ5LinD5e8X5Yh$	Spot on!!
$R_bwwc7dPFEcp1azH$	Given that comorbidity with other conditions (ASD/ADHD) is widely acknowledged, comorbidity with sensory or physical disability could also be possible. however the focus and model of intervention may be different
$R_6mrinfsu6CeSmBn$	We do need to be able to distinguish between a child whose language is showing disorder due to hearign impairment and those who have an underlying langaueg disorder and a HI.
$R_2o7JoTNgC3lqSIR$	Absolutely, children with language needs of any sort, should have them recognised and be given appropriate support. However, language difficulties that arise from other conditions are not the same as SLI and should not be categorised, or probably supported, in the same way.
$R_e5KJQmN6txthTRX$	It depends whether they actually have a language impairment or their language difficulty arises from their other disabilities. Whatever the situation, their language difficulties should be recognised and addressed

$R_7 1b9fvukXBUQ5dr$	Should document both - but not 'then' (later) describe difficulties - may well be the other way round (and chronologically, hearing loss and physical disability will often be diagnosed before language emerges). Intervention (e.g. amplification) may have to be in place for language to emerge, despite normal language potential.
$R_7 WXquZJy8WlgXAx$	this is a clear and useful statement
$R_4 ORQ8jYm1JwWwND$	Not sure what this means.
$R_1 QTm7VrpDX1OAi9$	I think this would be an extremely helpful way forward.
$R_3 rrKtkb2VvC3uG9$	Why would we every exclude a child with a hearing loss and language impairment from receiving a diagnosis of language impairment?
$R_8 bIXFrv4VBlvVyZ$	In the case of physical disability, careful consideration of history, and trajectory of development with speech and language characteristics would be required before automatically precluding, for example, every child with a physical disability, from a diagnosis of language impairment. A child possessing genetic markers for grammatical impairment and poor phonological short-term memory is at risk for language impairment. Birth anoxia resulting in a physical disability in that same child could in turn give rise to severe motor speech difficulties and limited expressive output.

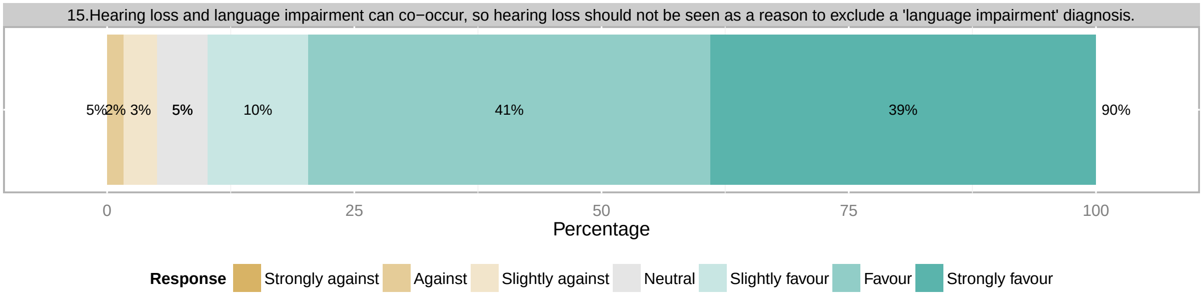

Figure 31: Percentage of panel members in each response category to statement 15. The percentages shown at each end of the scale are the cumulative percentages for the top and bottom three categories respectively.

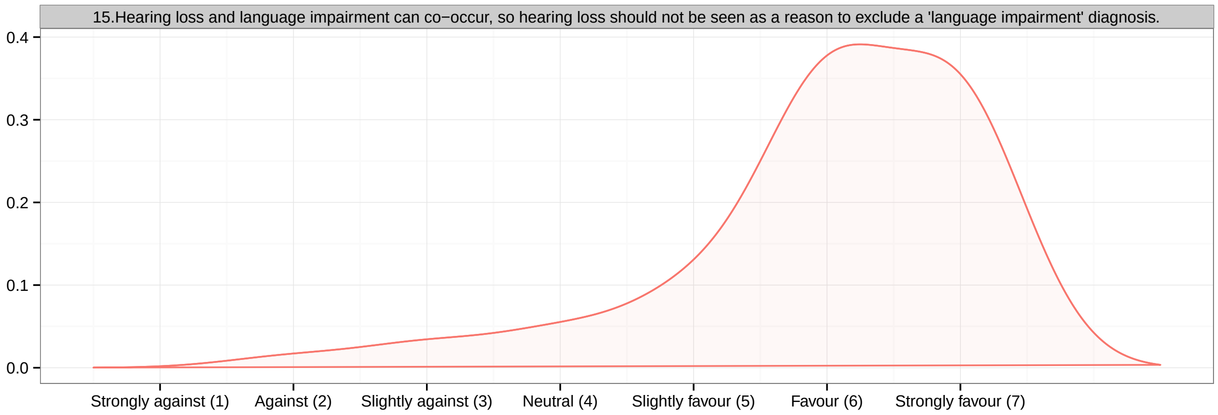

Figure 32: Distribution of responses to statement 15.

Table 15: Comments for each statement.

ResponseID	Q15B
$R_6RlkuyWJYcIIsmN$	Language impairments are one of the KEY difficulties for children with hearing impairments, so we cannot overlook this group if we're dealing with LI in childhood.
$R_6JOosydU46ZndMF$	my knowledge is insufficient to me to give strong opinion
$R_5cd8BDkYcGfGLKl$	although if child is signing, this raises issues of how one determines there is a language impairment, similar to issues of children who speak languages other than English.
$R_9uJ5LinD5e8X5Yh$	Disentangling the 2 is the problem - we need to be better at assessing. The enlightened world of hearing impairment acknowledges that HI can co occur. Why wouldn't it? Not in every case but there is strong evidence around.
$R_dguQPTfUoDzSKB7$	I agree with the statement, so LI in children with hearing loss should be treated differently in terms of diagnosis, classification and treatment. At the smae time we can examine the characteristics and compare if they overlap to cobine or not.
$R_6mrinfsu6CeSmBn$	We do need to be able to distinguish between a child whose language is showing disorder due to hearign impairment and those who have an underlying langaueg disorder and a HI.
$R_2o7JoTNgC3lqSIR$	Diagnostic criteria should acknowledge the possibility of co-morbidity. So if a child with hearing impairment has a language impairment in addition to the hearing difficulties, he should receive the dual diagnosis. If the language difficulties are simply the result of the hearing impairment this should not be seen as a language impairment in the same way.
$R_e5KJQmN6txthTRX$	If they co-occur, the language impairment should be recognised as such and be addressed
$R_71b9fvukXBUQ5dr$	Getting muddled in the double negatives here.
$R_7WXquZJy8WlgXAx$	but this does not necessarily mean that everyone with a hearing impairment has a language impairment

$R_8bIXFrv4VBlvVyZ$	Some children with cochlear implants for example, have been found to present with language impairments that are more severe than can be explained by their hearing loss so excluding automatically on the basis of hearing loss does not allow for this
$R_8AhxnQPe8mJkUoR$	There is evidence of co-occurring problems, for example LI in BSL. Problem in one area does not "protect" an individual from problems in other areas. However, as my responses above suggest, there are some cases, e.g. acquired brain injury, where the age, extent and type of the injury is likely to be a key variable which is likely to benefit from being conceptualised separately.

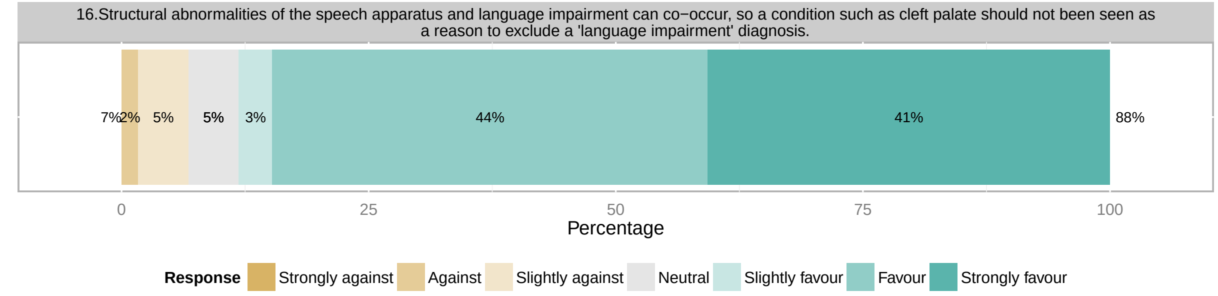

Figure 33: Percentage of panel members in each response category to statement 16. The percentages shown at each end of the scale are the cumulative percentages for the top and bottom three categories respectively.

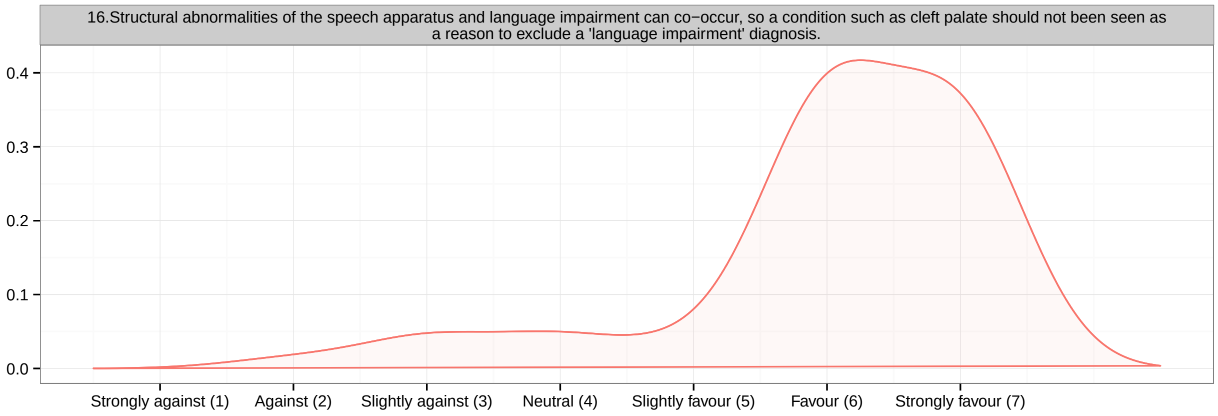

Figure 34: Distribution of responses to statement 16.

Table 16: Comments for each statement.

ResponseID	Q16B
$R_5cKMfR48zQytYc5$	as per question 11
$R_6JOosydU46ZndMF$	but needs to be specified because of implications for intervention
$R_5cd8BDkYcGfGLKl$	These children come under remit of SLT anyway, so I see this as less central to the issues where a different diagnosis might mean the child never gets to SLT.
$R_e9cPjWuFpcer4B7$	Again professionals often separate for their own convenience but the interventions may well be the same. Any interesting difference would be using prosthetics for specific conditions such as cleft palate and this may call for very specialised skills.
$R_9uJ5LinD5e8X5Yh$	May be better described as a speech output disorder rather than a language impairment. We do tend to muddy our own already murky waters I think. A quality asst will reveal if it is purely speech rather than language, but SLTs tend not to assess to this depth unless engaged privately. Is there enough knowledge in the SLT world of young therapists about speech skills?
$R_bwwc7dPFEcp1azH$	Agree where a primary/secondary classification is included
$R_dguQPTfUoDzSKB7$	again, we can have different syndroms, but they can all have language impairment. at the end we can examine how different profiles are given the variation in the syndromes, etiology, and unidentified causes
$R_6Dvhy7Alhw5wqIR$	there may well be an addtional languge impairment but is the question about speech production? that is different
$R_2o7JoTNgC3lqSIR$	No, the two are different. A child with a cleft palate might have speech difficulties as a result of the physical abnormality but a significant language impairment is a different issue altogether.
$R_e5KJQmN6txthTRX$	They're not the same thing at all. If a child with a cleft palate has a language impairment, it should be recognised and addressed

$R_7 1b9fvukXBUQ5dr$	There is no logical entailment, unlike unamplified hearing loss (although speech difficulties may directly result).
$R_7 WXquZJy8WlgXAx$	as above

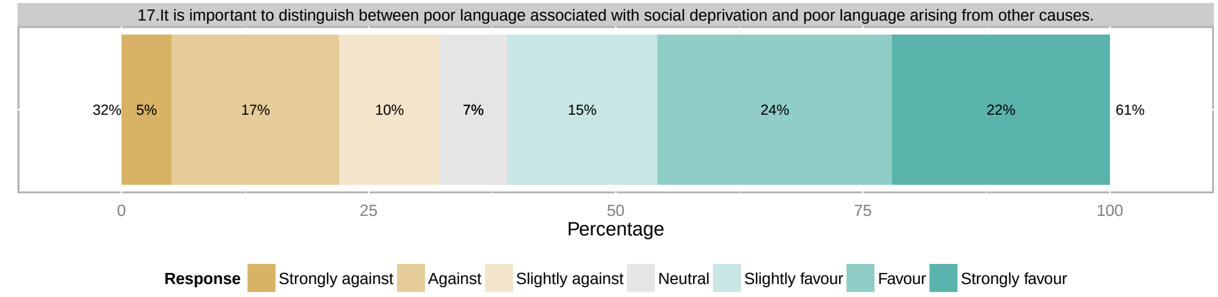

Figure 35: Percentage of panel members in each response category to statement 17. The percentages shown at each end of the scale are the cumulative percentages for the top and bottom three categories respectively.

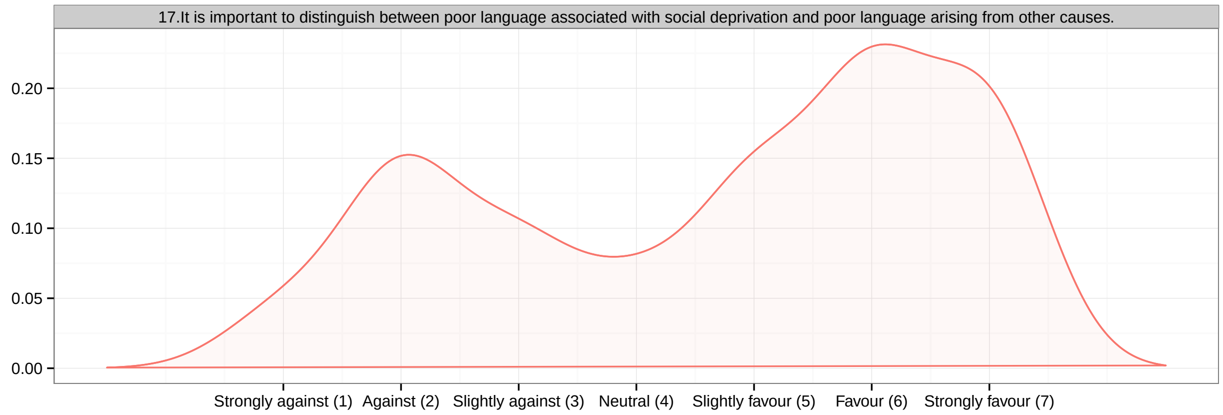

Figure 36: Distribution of responses to statement 17.

Table 17: Comments for each statement.

ResponseID	Q17B
$R_2f9ctxaHBJuJdLD$	It can be useful to consider in safeguarding or developmental cases eg Romanian orphans and ASD question
$R_6RlkuyWJYcIIsmN$	I think it's important to distinguish in the sense that we need to have a good understanding of the pathways by which children fail to meet language criteria - I don't mean that these children should be "put to one side". / Low SES has a clear and measurable effect on children's language skills. If (for arguments sake) 40% of children from a low SES background have language scores >1.5SD below a standardized mean, against say 8% from more privileged backgrounds, then we need to ask ourselves about the inverse of language impairment - language COMPETENCE. What is it, and how do we know when it is present? Low SES children may have adequate language competence to deal with everyday social and environmental demands, but they will struggle to meet the more middle-class language demands of school and the workplace. / Children perform poorly on language measures for a range of reasons, and low SES is one of those reasons. / We also need to remember that some low-SES children will have additional language-learning needs (identifiable or not) - low SES is not mutually exclusive of ASD, ADHD, etc.
$R_5cKMfR48zQytYc5$	i don't believe its about distinguishing between them. only in extreme cases does deprivation alone cause impairment. but there is a strong social gradient that impacts on language development and outcomes - some of this may be social some may be biological.
$R_6JOosydU46ZndMF$	depends on the implications for intervention

$R_bOrkJKVQ6T8FeGp$	Easier said than done however! Surely only trials of intensive intervention/markedly modified environmental change will show whether the child had poor language due to impoverished envionmental language input? Should we make distinctions that will be almost impossible to determine? How do we take into account the genetic mix in these cases where often the parents providing a poor language environment have a genetic bases predisposing them to their socio-economic situation?
$R_5cd8BDkYcGfGLKl$	It seems that many people do not appreciate that the environmental context may also reflect genetic factors that may contribute to language impairment.
$R_3sXNbQYRlZaMb3L$	Again, the question is do these two groups of children need different intervention?
$R_e9cPjWuFpcer4B7$	This is an interesting question because it is pretty difficult to do this from the child's language skills as a number of studies have shown. One can do it by making a judgement about the parent and their resources but this is quite wrong from my perspective. One of the problems here is the use fo the term "language delay" implying that children grow out of it. it is clear that there are subsets of any group of children with early difficulty for whom those difficulties persist. indeed one could argue that for many children having what used to be known as "specific" language impairment without associated risk factors such as psychopathology or environmental disadvantage was effectively a protective factor - ie their long term outcomes were pretty good.
$R_9uJ5LinD5e8X5Yh$	Delay can be caught up with, neurological damage is far more long term. Best use of resources dictates we understand the 2. Delay has to be caught when the child is young - prior to school entry; catch up can mean relatively little lack of progress in school.
$R_1TXxdyLg1UFCx4V$	Language Impairment and social deprivation are often associated and there is no clarity as yet about causality i.e. whether social and/or genetic factors are involved. A profile on individuals with LI for research or clinical purposes should incorporate this information because it is relevant to case management and for research
$R_3pDedyU4fM1kOXj$	I am not sure if it is not easy to distinguish between these two completely. How can you prove that a child who is living in social deprivation was not born with a language impairment?
$R_eOEFfbvY55KRtRP$	I think that this would be very difficult to do in practice
$R_6Dvhy7Alhw5wqIR$	and how would one do that with certainty? I DO think that speed of improvemt/change over time si diagnostically helpful as with all developmental disorders
$R_dmR80BQCC0tAFuZ$	I think this distinction risks gross inequality in service provision
$R_4HGIGYFIvMxLWcJ$	Again I think it should be described as a factor.
$R_6mrinfsu6CeSmBn$	I think we need to distinguish between chidlren who have poor language because they have not had opportunities to develop better, and those who have internal language learning difficulties. These two groups of children need very different things to improve their language.
$R_2o7JoTNgC3lqSIR$	Admittedly, this is not always easy to do, but unless we emphasise the distinction, language impairment will be viewed by policy makers and others as a social deprivation issue rather than a medical/educational disability.
$R_e5KJQmN6txthTRX$	It might not always be easy to do, but is essential. The current focus on language as a social deprivation issue means that language impairments are not always identified or taken seriously. Increasingly too, SLTs seem to be focusing on children with impoverished language rather than those who really need highly skilled help
$R_5AzMzLGZTUhhjKt$	It depends if that is clear delineation between these two. I don't think there consistently is
$R_71b9fvukXBUQ5dr$	Again, the language output may be indistinguishable, but underlying factors may be amenable to different interventions. 'Associated with' is difficult - reciprocal problem.
$R_7WXquZJy8WlgXAx$	there are qualitative differences in these two types of language difficulties, and also in their persistence and response to intervention
$R_1QTm7VrpDX1OAi9$	I don't think these can be distinguished unless Response to Intervention is included. Social deprivation and language impairment can also co-occur (and often do partly due to genetic factors and parents low qualifications), therefore these can only be distinguished where a child with a socially deprived background has responded well to general intervention and therefore probably did not have a language impairment. Once they no longer have needs, distinguishing why they had them in the first place is no longer relevant.
$R_1z8h1XMT676UOwd$	Presumably if deprivation is the cause then enriched input alone may help, provided there has not been too long a period of deprivation that has impaired the child's ability to learn once input is provided. Enrioched input alone without the use of specific therapy techniques is likely to be insufficient for a child with SLI due, for example, to a difference in brain organisation.

$R_3DfMsLnqK54HqcZ$	This is a somewhat moot point as we can't currently do this. It also strongly biases towards a genetic explanation for LI ignoring gene environment interactions. Furthermore making such a distinction could privilege LI with or without social disadvantage in terms of access to intervention. A more useful distinction might be transient versus persistent LI which could be determined through response to intervention. Language
$R_23qAFVuJCo6YHOd$	Social deprivation - may be due to parents having poor language skills.
$R_8bIXFrv4VBlvVyZ$	Language impairment can occur across SES groups with similar characteristic; determining the relative contributions of deprivation and other causes of LI would be very difficult on an individual basis in practice; additionally severe deprivation alone might over time result in the same set of characteristics as a childn with LI who was not deprived.

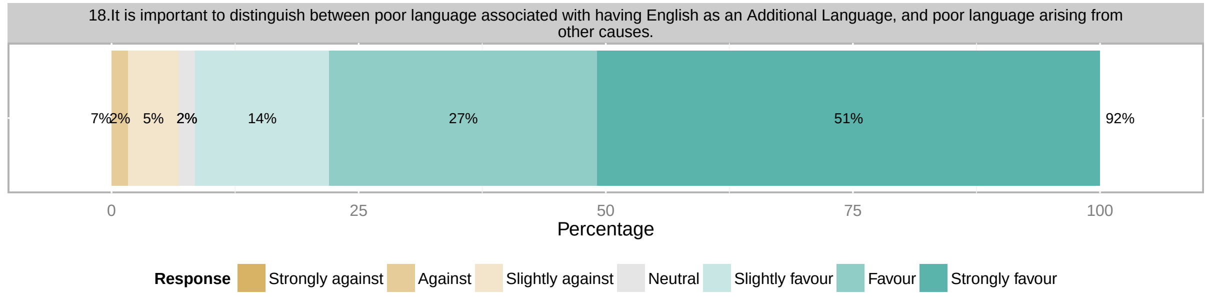

Figure 37: Percentage of panel members in each response category to statement 18. The percentages shown at each end of the scale are the cumulative percentages for the top and bottom three categories respectively.

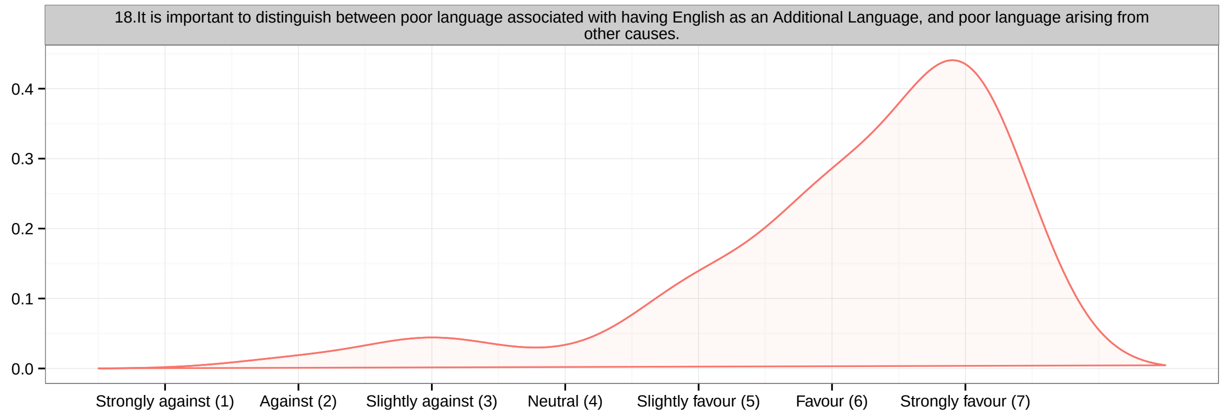

Figure 38: Distribution of responses to statement 18.

Table 18: Comments for each statement.

ResponseID	Q18B
$R_ba8iHG84IJ8cW7X$	The statement does not make it clear whether the issue is about perceived 'poor English language' or language difficulties that are not specific in any one language.
$R_2f9ctxaHBJuJdLD$	Makes sense- also there is good evidence to support enhanced brain executive function with multilingualism
$R_5cKMfR48zQytYc5$	yes kids who have impoverished language as a result of EAL needs a different approach BUT there will be some who may well have LI. these kids need careful training to ensure they don't clog up clinics
$R_6JOosydU46ZndMF$	depends on implications for intervention
$R_bOrkJKVQ6T8FeGp$	As above however, easier said than done!
$R_5cd8BDkYcGfGLKl$	It is imperative that we find a way of identifying those with EAL that likely have problems with language learning. These children really slip through the net.
$R_e9cPjWuFpcer4B7$	One would assume that the prevalence in any language would be the same. the problem of course is if people assess in English when it is quite obvious that the child does not speak it. There is concern that the SLCN category has been used by schools in the early primary school years to allocate additional resources to these children. This may be appropriate but it should not be defined as a problem per se. Indeed there are a number of studies which suggest that bilingualism on its own often confers an advantage to the child.
$R_9uJ5LinD5e8X5Yh$	EAL learners have language needs in the initial stages, but then can make rapid progress. Early intervention is critical so that there is no barrier to longterm learning. / Better asst of EAL plus SLI is needed............ the 2 can coincide, but that is rarely discovered soon enough because of professionals' assumptions

$R_1TXxdyLg1UFCx4V$	For similar reasons as above becausethey can co-exist
$R_cLU7KRGW2XvEql7$	is this about language disorder in L1 or just about difficulty learning English?
$R_2o7JoTNgC3lqSIR$	Absolutely. They are not the same thing at all. Of course children with English as a second language need language support but not SLT type support, unless they also have a language impairment in both or all their languages.
$R_71b9fvukXBUQ5dr$	Again, a normal language learning potential with limited English proficiency due to limited exposure may require different interventions.
$R_1QTm7VrpDX1OAi9$	However, for those with sufficient exposure to English, a language impairment should also be considered where the child is struggling with English (and/or their first language)
$R_3DfMsLnqK54HqcZ$	Children with EAL are at risk of over and under-identificaiton of LI. Poor majority language (in this case English) in children with EAL is not LI. But poor language in a home language may LI. So it is not the group - children who speak EAL - who should be excluded but a particular pattern of language abilities within an EAL context which should be excluded.
$R_eG1jl51DiHRqXKB$	Important but often difficult to measure
$R_8AhxnQPe8mJkUoR$	Understanding multilingual children and valuing home languages is an important part of a pluralistic society. My response is in the context of this consideration.

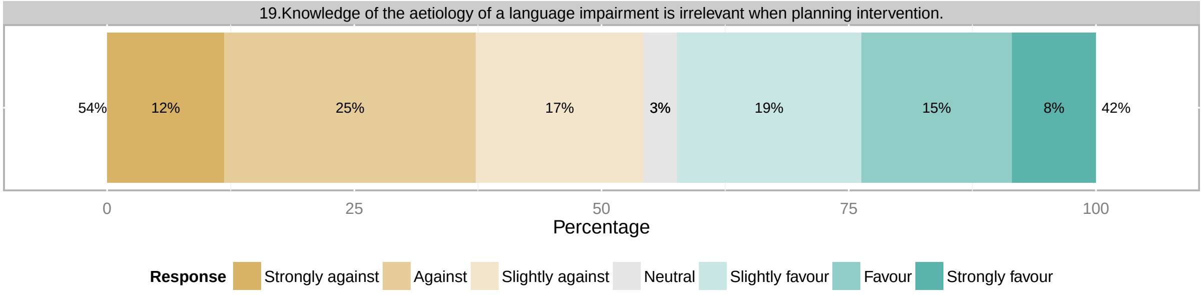

Figure 39: Percentage of panel members in each response category to statement 19. The percentages shown at each end of the scale are the cumulative percentages for the top and bottom three categories respectively.

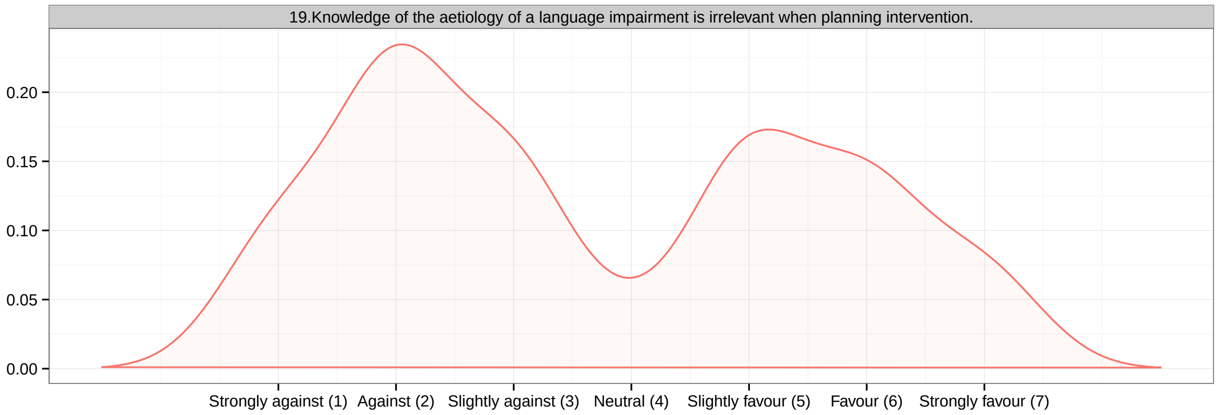

Figure 40: Distribution of responses to statement 19.

Table 19: Comments for each statement.

ResponseID	Q19B
$R_2f9ctxaHBJuJdLD$	Having an idea of causation will assist the planning of intervention
$R_5cKMfR48zQytYc5$	we know so little about what underpins LI that we can't disregard this
$R_6JOosydU46ZndMF$	hmmm. depends: some etiology is important, particularly for intervention, such as hearing impairment, acquired brian injury, autism etc
$R_5cd8BDkYcGfGLKl$	most of the time it probably won't matter, but sometimes it could (e.g. hearing loss).
$R_e9cPjWuFpcer4B7$	We have to separate out what might be termed management and treatment here. The treatment of the language is unlikely to be sensitive to the diagnosis unless that diagnosis incorporates behavioural symptoms which might affect the way the child was treated. For example the child with language difficulties associated temporal lobe epilepsy might need a particular management regime. But this is my point about the distinction. Management might differ because of the different professionals, special school requirements, age may determine parental involvement etc. but the actual language work would, I would suggest follow the child's language needs, not their diagnosis. Recall that most children with language learning difficulties are managed in schools as are children with reading difficulties. Diagnosis is a medical construct rarely shared by educational staff and entirely dependent on the proximity of and access to the medical assessment or perhaps researchers.
$R_9uJ5LinD5e8X5Yh$	Essential is knowledge of normal patterns of language acquisition so that fast asst is made when it starts to go wrong/is wrong in classrooms. I spend a lot of time teaching ITT students where lang development can go wrong - they have no idea how a child learns language, they need some detail so that they can better assess inside classrooms (instead of seeing all as poor behaviour/low cognitive potential).

$R_1TXxdyLg1UFCx4V$	The wording of this statement is difficult. Aetiology is important when planning interventions, it influences the type and possibly the delivery of an intervention and the expectations for outcomes but it should not be used to influence the resources provided to support the individual without proper evaluation of level of need.
$R_bwwc7dPFEcp1azH$	Although aetiology should not be the only factor, it does help when considering the evidence base for interventions that have been shown to work for certain groups
$R_4HGIGYFIvMxLWcJ$	It may assist in prognosis, especially if we consider children with TBI or DS. Reality is that we often do not know the aetiology anyway
$R_2o7JoTNgC3lqSIR$	It might be relevant but it might not. The more you know about the child, the more closely you can match the intervention.
$R_e5KJQmN6txthTRX$	The presentation is the important thing, but if there is other relevant information this should not be ignored
$R_71b9fvukXBUQ5dr$	See earlier comments.
$R_7WXquZJy8WlgXAx$	it is most important that the profile of strengths and needs are described. it is also important to take into account a range of other fctors when planning intervention; one of these may be aspects of aetiology - but not always, especially when this is sometimes not known. In practice, it can be unhelpful to focus on the cause of a language impairment - especially for parents
$R_1QTm7VrpDX1OAi9$	Intervention should be based on a detailed assessment of the child's background, aetiology and profile. Aetiology is one of many aspects which should be considered when planning intervention, so is relevant, but the profile of the child's strengths and weaknesses is more important.
$R_1z8h1XMT676UOwd$	It is useful to know if there is a familial pattern, for example, as other members of the family may have difficulties themselves, impacting on the ability to support therapy.A child with SLI associated with brain injury may have cognitive communication difficulties that could affect engagement with therapy. Exposure to neurotoxic elements could affect other aspects of brain function which should be considered in therapy. As genetic bases of SLI become known in the future this may improve understanding of prognosis and specific difficulties that are resistant to therapy.
$R_3DfMsLnqK54HqcZ$	It isn't irrelevant as - where this is known - it may give some indications as to prognosis and likely co-morbid difficulties etc. However they are not and should not be the primary concern when planning intervention and so I favour classification based on patterns of strengths and weaknesses in particular dimensions (as described above). Also common misconceptions about differences between groups based on these etiological distinctions need to be challenged (e.g. socially disadvantage vs. 'real' SLI having identifiably different patterns of impairment) and prognosis.

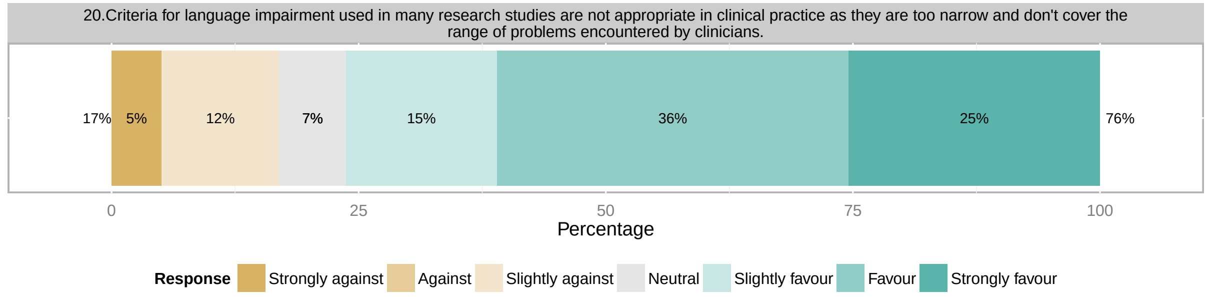

Figure 41: Percentage of panel members in each response category to statement 20. The percentages shown at each end of the scale are the cumulative percentages for the top and bottom three categories respectively.

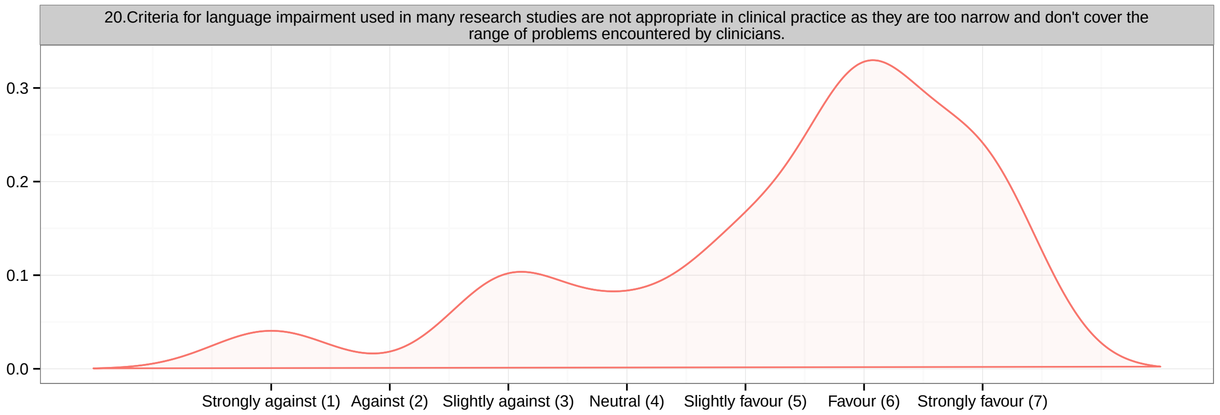

Figure 42: Distribution of responses to statement 20.

Table 20: Comments for each statement.

ResponseID	Q20B
$R_5cKMfR48zQytYc5$	theses clear form population studies
$R_e9cPjWuFpcer4B7$	This is almost certainly true.
$R_9uJ5LinD5e8X5Yh$	The exclusion criteria for most research are so tight that only the pure SLI are looked at. The world of schools has moved on in the surge of inclusion and a better fit picture (more complex) is needed
$R_1TXxdyLg1UFCx4V$	This is true now, but there has been an evolution of research knowledge that may have required tightly defined cohorts to help establish and explore the concept of language impairment
$R_eOEFfbvY55KRtRP$	Research studies - especially some of the SLI /expressive studies - often have criteria that include children who would be unlikely to receive therapy in the UK. It would be worthwhile having some kind of characterisation of research groups which would allow clinicians to know if the participants are actually similar to their own caseloads
$R_2o7JoTNgC3lqSIR$	Actually, I sometimes wonder if the criteria used in some research studies are too broad, certainly compared to the, admittedly more severe, cases we generally deal with - though even then are often struggling to get their needs taken seriously,
$R_e5KJQmN6txthTRX$	It is important that research is helpful to clinicians and parents and others
$R_71b9fvukXBUQ5dr$	Probably - but research studies should specify their participant characteristics. If clinicians need information of different groups of participants, different (additional) studies are needed.
$R_7WXquZJy8WlgXAx$	this is probably so - but this is becasue we struggle to find a set of criteria - hence this exercise!
$R_1QTm7VrpDX1OAi9$	This is a huge problem and means that we don't know for example how well children with language impairments and other co-occurring diagnoses respond to intervention.

$R_1z8h1XMT676UOwd$	It is useful to have research on narrowly defined clinical groups, but also helpful to have studies that accept heterogeneity in the language impaired population and try to have sufficient sample size to see what factors amongst the varied group are associated with certain outcomes.
$R_ebTqVBlGUNh60eN$	particularly in relation to non verbal abilities in the school-aged population and bilingualism

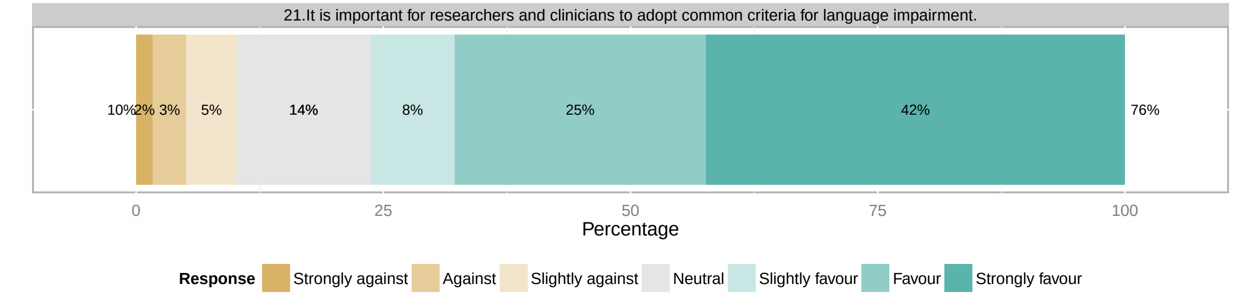

Figure 43: Percentage of panel members in each response category to statement 21. The percentages shown at each end of the scale are the cumulative percentages for the top and bottom three categories respectively.

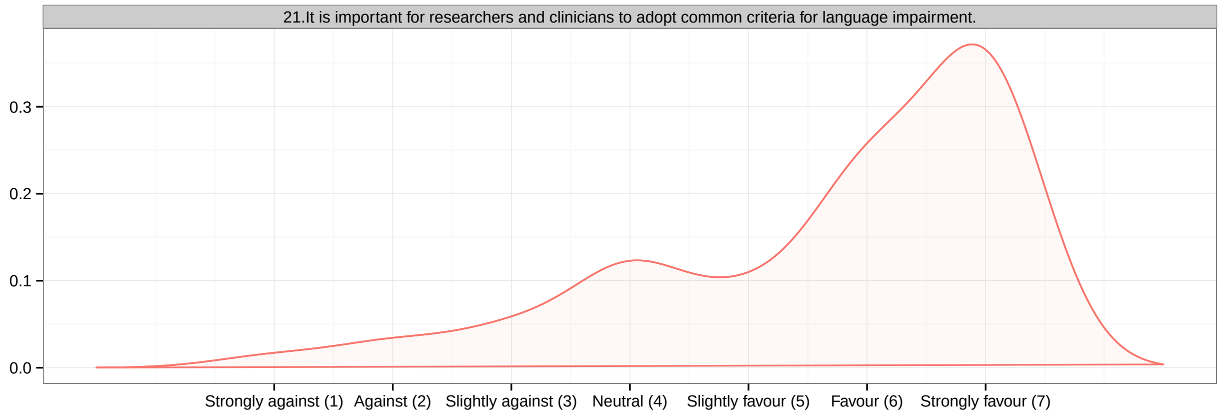

Figure 44: Distribution of responses to statement 21.

Table 21: Comments for each statement.

ResponseID	Q21B
$R_2f9ctxaHBJuJdLD$	Rather essential for putting results into practice
$R_cIxZunCo2wnTfVj$	Depends upon the situation. It's important for everyone to be able to talk to each other, so everyone needs to understand the criteria that are being used, but sometimes researchers need to have narrower sets of criteria.
$R_5cd8BDkYcGfGLKl$	It would help us generalise findings.
$R_e9cPjWuFpcer4B7$	The criteria depend on the question that is being asked and this will vary according to context. One of the problems we have is that after forty years of studying this subject and more supporting these children we do not have the metaphor for these children's needs which captures the popular imagination in a way that Autism Spectrum Disorder and Dyslexia have done. One might argue that this has got a little out of hand in the case of ASD.
$R_9uJ5LinD5e8X5Yh$	Talking one language would be such a treat! In a field of thorns why do we make life more difficult for ourselves!
$R_0ofhSCmeppIQ8kt$	But still, the clinician will be seeing a much broader population of children with language differences only some of which may meet the criteria for language impairment.
$R_cCuacCYZiqQHKgl$	In the long-term I would hope that research and clinical notions would converge. However, there are factors that constrain and influence clinical decisions regarding service that should not influence research.
$R_2o7JoTNgC3lqSIR$	Otherwise we could be talking about apples and pears - indeed sometimes I think we are anyway.
$R_e5KJQmN6txthTRX$	We all need to agree what we're talking about
$R_5AzMzLGZTUhhjKt$	I think so - although research will need to offer additional criteria/description to make it clear who the population in their study is

$R_71b9fvukXBUQ5dr$	WHO again?
$R_7WXquZJy8WlgXAx$	This would be helpful, if possible. if not, then we have to accept that this may not be possible. what is more essential is to idenitfy what the selection criteria are that have been used
$R_1QTm7VrpDX1OAi9$	I understand the need for some very theoretical research to have tighter criteria. However, other research (in particular intervention research) needs to cover the whole range of profiles
$R_3DfMsLnqK54HqcZ$	I agree but I also agree that research can also subdivide the umbrella term used clinically depending on the research aims and questions. This should also encourage researchers to much more clearly define and describe the populations in their studies and not brush under the carpet the tendency for children with SLI to have lower SES and nonverbal IQ than controls groups (even if non-significant - these trends often are present). For intervention and epidemiological studies then clinical criteria should be used so as to allow generalisation to practice and, potentially, subgroup analysis to be conducted to consider whether or not differential response to intervention exists.
$R_23qAFVuJCo6YHOd$	Narrow criteria may be needed for research studies only.
$R_8bIXFrv4VBlvVyZ$	There are good reasons for researchers to adopt a more narrow set of criteria in particular studies, so as to minimise the possibiltiy of confounding variables and have a more homogeneous group from which to interpret findings; but I would favour intervention studies being less prescriptive in applying inclusionary/exclusionary criteria-in -depth descriptions of and assessments of children included would allow for interpretation, this in turn will better match the mixed caseloads of clinicians

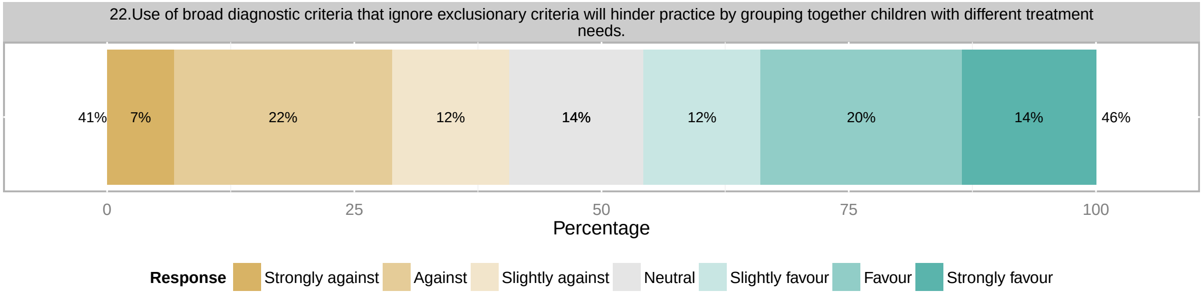

Figure 45: Percentage of panel members in each response category to statement 22. The percentages shown at each end of the scale are the cumulative percentages for the top and bottom three categories respectively.

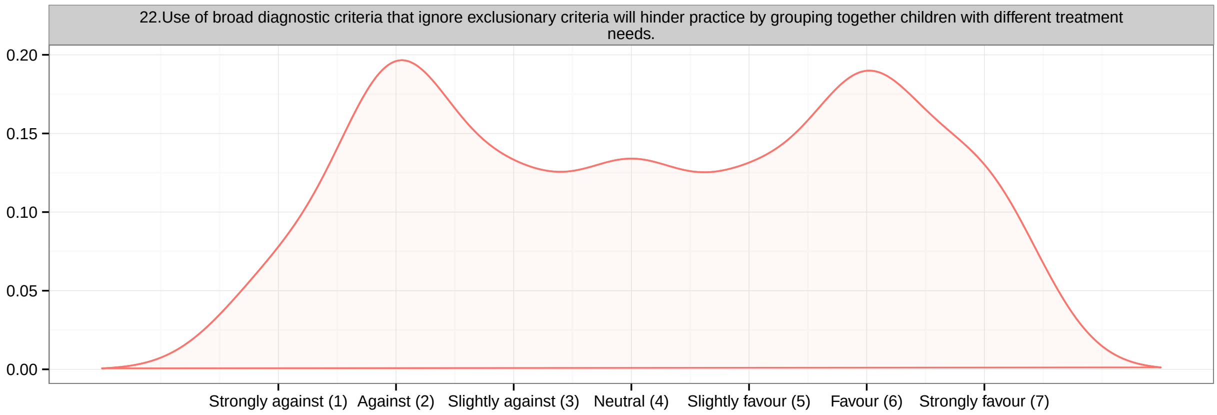

Figure 46: Distribution of responses to statement 22.

Table 22: Comments for each statement.

ResponseID	Q22B
$R_6RlkuyWJYcIIsmN$	I see the purpose of diagnostic criteria as answering the question “Does this child have a language impairment”? Having established that the answer is yes, it is the job then of a skilled clinician to determine treatment needs.
$R_5cKMfR48zQytYc5$	no we need better understanding about origins of the problems and ways of filtering kids and what they need
$R_5cd8BDkYcGfGLKl$	we don’t have any evidence that different treatment needs are required.
$R_6LIAgEx6sspizpX$	Treatment needs tend to be grouped under headings such as ‘vocab’, ‘narrative skills’, ‘syntax’ etc and these oculd apply to any child who has language difficulties. The broader grouping may hinder identification of profiles of language difficulties (if they exist) associated with particular diagnostic groups which might make it difficult to plot longitudinal profiles which might be important in identifying treatment priorities.
$R_3sXNbQYRlZaMb3L$	Not if these children all need the same thing! I’m not saying that they do, only that it’s important to know.
$R_e9cPjWuFpcer4B7$	The treatment needs are not necessarily determined by the specificity of the diagnosis. The need is within the individual not the category.
$R_9uJ5LinD5e8X5Yh$	Presentation clusters would be much more useful - and ensure that different provision is tailored to need far more accurately and successfully for the child’s progress. Peer groups are important to children.
$R_1TXxdyLg1UFCx4V$	There are almost no contexts in which individuals with LI have identical treatment needs even within one purported diagnostic group. Most children are in a context with a wide variety of treatment and learning needs

$R_eOEFfbvY55KRtRP$	I think the exception to this is that children with E2L should be included as they may have same underlying conditions and may benefit from same treatments but are often automatically excluded as messy data
$R_dguQPTfUoDzSKB7$	it does not say they have to be treated equally.
$R_2o7JoTNgC3lqSIR$	Indeed. Surely precise diagnosis is needed to identify what support is needed.
$R_e5KJQmN6txthTRX$	Intervention needs to focus on individual children's needs
$R_71b9fvukXBUQ5dr$	This is certainly a danger.
$R_7WXquZJy8WlgXAx$	It is oikportant that whatever criteria used in a study are described well. this might mean, for example, specific studies on LI in the context of Downs Syndrome
$R_1QTm7VrpDX1OAi9$	I think broad diagnostic criteria, but with detailed descriptions of the child's profile and background will improve practice
$R_1z8h1XMT676UOwd$	It may be that SLI can be defined narrowly but that concomitant conditions, known risk factors, and other areas of difficulty can be included in the diagnosis as supplementary/explanatory information.
$R_23qAFVuJCo6YHOd$	A diagnosis alone should not determine the provision, the whole child's profile and impacts will always need to be considered in informing educational placement and provision needed. / Exclusion from the group could mean that language needs are not recognised and only the other factors (e.g. hearing impairment) are addressed.
$R_eG1jl51DiHRqXKB$	I found this question hard to answer. I think that practice should focus on what children need based on assessment.

2.4 Preschoolers/transient problems

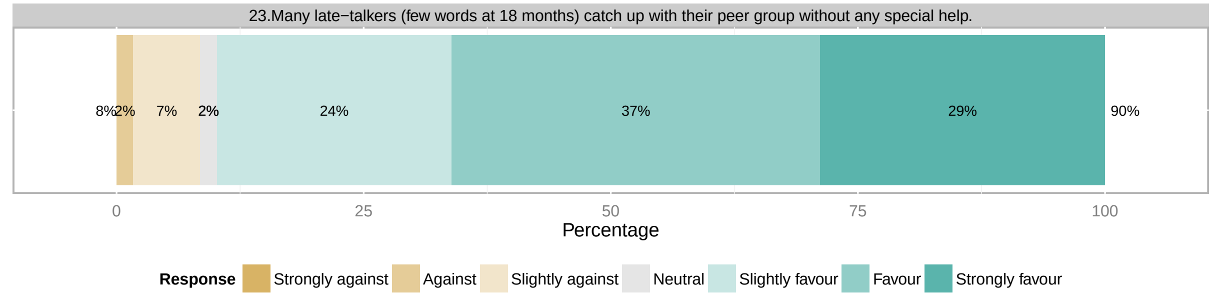

Figure 47: Percentage of panel members in each response category to statement 23. The percentages shown at each end of the scale are the cumulative percentages for the top and bottom three categories respectively.

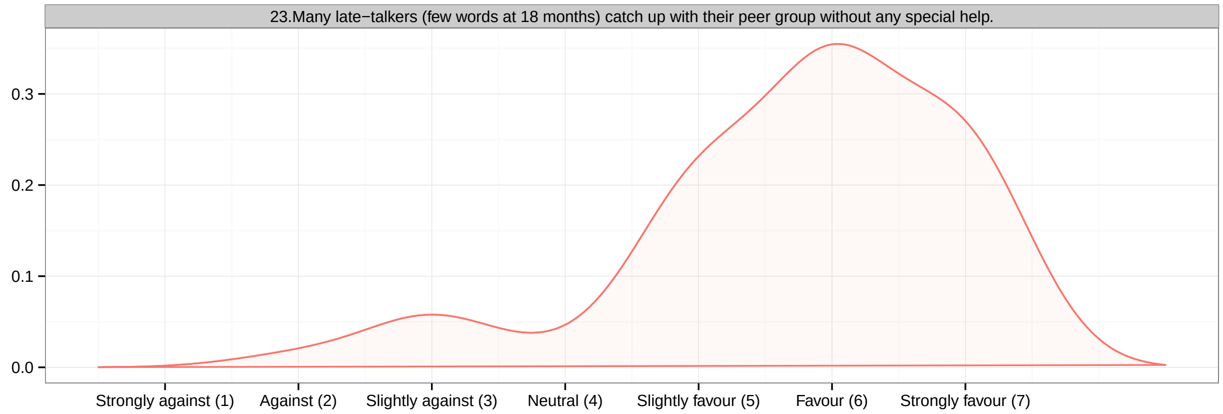

Figure 48: Distribution of responses to statement 23.

Table 23: Comments for each statement.

ResponseID	Q23B
$R_2f9ctxaHBJuJdLD$	Identifying those that are on a pernicious trajectory is the key
$R_6JOosydU46ZndMF$	depends - some research suggests longer-term implications for literacy
$R_5cd8BDkYcGfGLKl$	important given government agenda for early intervention at the exclusion of services for older children.
$R_3sXNbQYRlZaMb3L$	This is my understanding, but I would like there to be more information available for non-SALTs and for early years practitioners on this. Especially knowing a clear age cut-off for concern and referral.
$R_e9cPjWuFpcer4B7$	“Late talkers” with no other developmental and behavioural difficulties are not generally at risk of poor outcomes.
$R_9uJ5LinD5e8X5Yh$	Many but not all. Broad statement such as this on can be unhelpful/disastrous for the few. We need stats really. Many or some late talkers can catch up if etc etc
$R_3pDedyU4fM1kOXj$	I think this depends on what your definition of a late talker is? How severe is there delay (6 months, 12 months....)? Do they have receptive and expressive language difficulties?
$R_eOEFfbvY55KRtRP$	Except that appropriate advice at this stage can identify children who would benefit from further monitoring and support - especially those without a facilitatory environment.
$R_dguQPTfUoDzSKB7$	its about 50% I thought..
$R_71b9fvukXBUQ5dr$	Reasonably good research shows this.

$R_7WXquZJy8WlgXAx$	It depends on the communicative environment they are in. some do, many don't without some support.
$R_1QTm7VrpDX1OAi9$	we really need to get better at predicting who catches up and who doesn't. What are the red flags?
$R_1z8h1XMT676UOwd$	I think we still need more evidence for this, as some late talkers may catch up but then go on to have longer term difficulties that have not yet been fully explored in research.
$R_23qAFVuJCo6YHOd$	It is important however, that this is not used an as reason not to provide support where a child has obvious difficulties, obvious persistent difficulties or it is impacting on the child's well being. / Early intervention is important for those who will have persisting difficulties.
$R_bQ13TaeUPFsxVJP$	Yes, but they may need to be monitored if language comprehension is inadequate, they show a lack of any intent to communicate, or they have a family history of speech and language disorders.
$R_8bIXFrv4VBlvVyZ$	Yes, longitudinal studies support this, but we do need to be aware that even a slightly below average score can persist to the extent that ability to learn words/progress academically may be less than optimal-but where this is the case, clinical identification and targeted/specialist intervention may be unnecessary-rather, good universal supports.

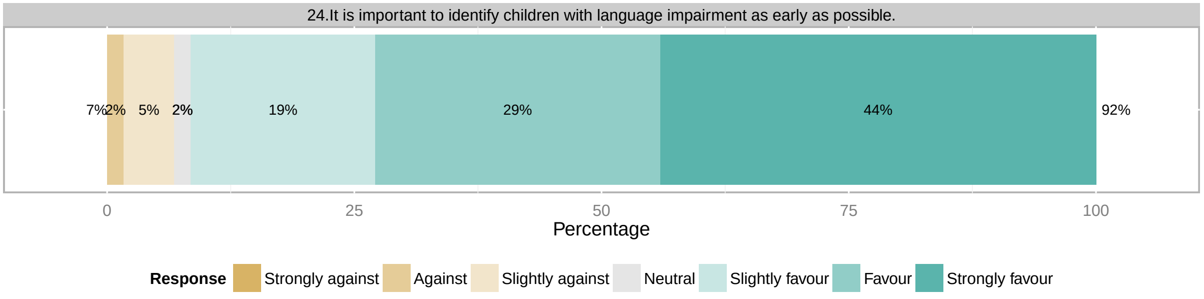

Figure 49: Percentage of panel members in each response category to statement 24. The percentages shown at each end of the scale are the cumulative percentages for the top and bottom three categories respectively.

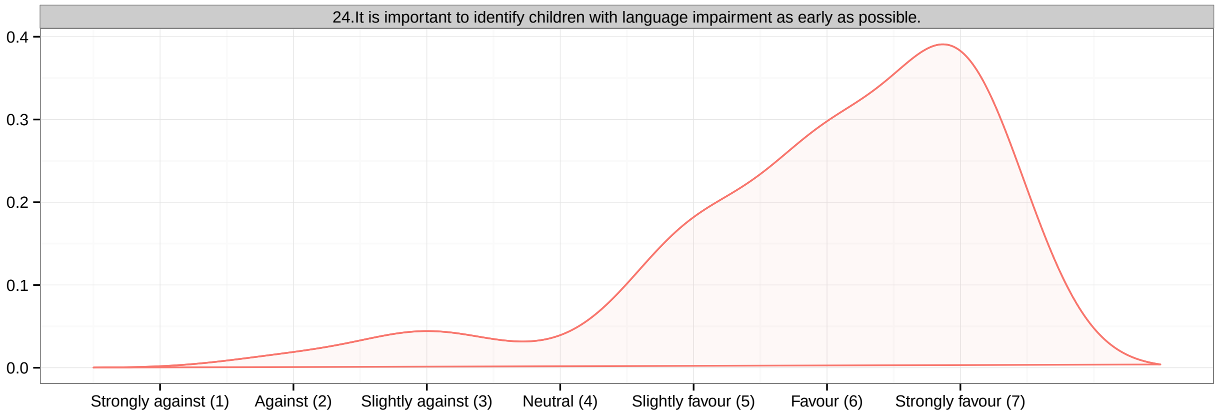

Figure 50: Distribution of responses to statement 24.

Table 24: Comments for each statement.

ResponseID	Q24B
$R_5cKMfR48zQytYc5$	My heart says yes but some of he data says not always. LI don't emerge in some kids until later. if we treat all 2 or 3 year olds then we will treat lots of kids who will get better anyway
$R_6JOosydU46ZndMF$	well - this is current conviction...i am not sure of strength of evidence or whether there is a sensitive window of opportunity after which intervention has little or no impact
$R_6JZKVRyNZK6U0zX$	Although this isn't practical at the moment, due to the large number of false positives, research needs to continue to pursue this goal.
$R_5cd8BDkYcGfGLKl$	language difficulties are often signposts to other conditions which may benefit from early intervention. may also be important to discuss with parents and document change. that doesn't necessarily mean there is a need to treat early though...
$R_6LIAgEx6sspizpX$	Sometimes we can identify issues as 'problems' too early. This means the family may be anxious when there is no need to be and services use precious resources with chidlren who are going to be fine. Maybe our norms are too prescriptive (or just wrong?)
$R_3sXNbQYRlZaMb3L$	I think the challenge is that we know that for many children language delay does resolve itself.

$R_e 9cPjWuFpcer4B7$	if we can do this accurately then we should but we are not very good at this. So we need to adopt the sort of recommendations made by Shonkoff and others about promoting parental engagement in their children's development. This an issue of universalism. But we should not go as far as to say all resources should be associated with prevention in the first couple of years of life because we do not have the evidence to support the interventions. Of course there are many interventions such as Incredible Years which do have a relatively strong underpinning evidence base albeit not using language outcomes and this could be explored further. The key thing here is to focus on early identification but not to conflate this with identification of the youngest children, a distinction being drawn by the Early Intervention Foundation. So the important thing is to provide the intervention at the point where the need is identified, not just when the children are two.
$R_9 uJ5LinD5e8X5Yh$	A no brainer really!
$R_e 5KJQmN6txthTRX$	Parents often feel they are made to wait unnecessarily for something to happen
$R_7 WXquZJy8WlgXAx$	there is evidence for early identification in relation to later outcomes
$R_1 QTm7VrpDX1OAi9$	We need to identify children at high risk of having persistent difficulties early (but how?). However, we don't need to identify all late talkers; this could do more harm than good (both to the children and their families and to services who have little time left for seeing children with more persistent difficulties).
$R_2 3qAFVuJCo6YHOd$	Yes, but bearing in mind that some will catch up. It is important to recognise those with obvious significant difficulties as some may show these at an early age. It is also important to identify risk factors for those who may turn into a significant concern, provide the right advice at this stage, and monitor closely to intervene if the child is not catching up.
$R_8 bIXFrv4VBlvVyZ$	but there is a difference between identifying risk and a possible impairment versus identifying a persistent impairment that needs and will benefit from targeted and specialist support
$R_8 AhxnQPe8mJkUoR$	This is a very difficult statement as early identification is important but the evidence does not support the "usual" thinking of early identification of LI before 3 years of age unless specific profiles are present, e.g. poor comprehension.

2.5 Assessing language difficulties

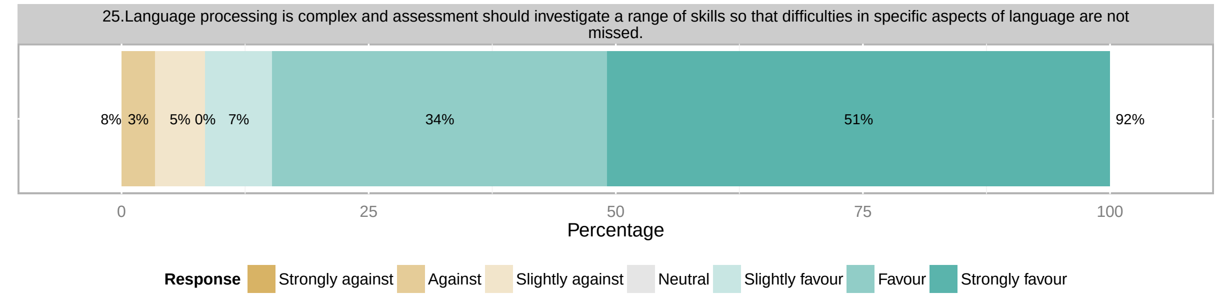

Figure 51: Percentage of panel members in each response category to statement 25. The percentages shown at each end of the scale are the cumulative percentages for the top and bottom three categories respectively.

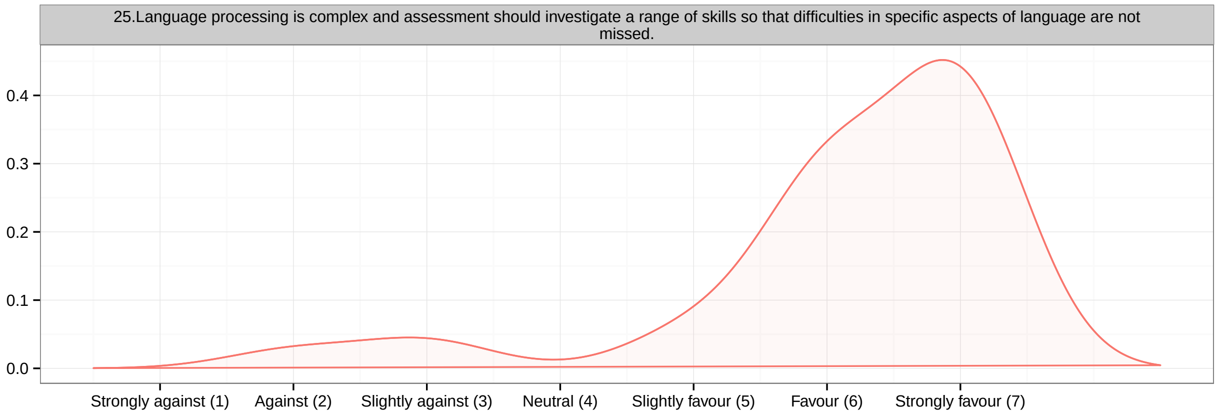

Figure 52: Distribution of responses to statement 25.

Table 25: Comments for each statement.

ResponseID	Q25B
$R_5cd8BDkYcGfGLKl$	evidence from a number of studies suggests that language tests are very highly correlated. There is probably a core battery that will cover most bases.
$R_6LIAgEx6sspizpX$	Although there might be an argument that says we should identify with the family/school what the main presenting issue is and target that in intervention and take a more incremental approach to assessment.
$R_e9cPjWuFpcer4B7$	This is a poor question and should not be included in this format. Language processing skills, like all those involved in most other behaviours such as executive function, can be complex. Whether one can miss specific details that would make a radical difference to treatment is an empirical question which has not, as far as I know ever been tested. The answer is it depends on the need identified and the reality is that exhaustive assessment is only ever going to be for the few.
$R_9uJ5LinD5e8X5Yh$	Poor initial asst is often a real problem ie BPVS above AE, but no mention of comp of running speech (eg 0.1%ile) which makes all the difference for inclusion! If SLTs could carry out a better spread of standardised assts this could only be helpful for other professionals to understand the needs of the child. My 3rd book is about comp levels being the driver for success in a mainstream school - putting a yr 5 child with a comp of a yr 2 child into a yr 5 classroom for inclusion cannot work! The gallows await me!
$R_cCuacCYZiqQHKgl$	Although language processing is indeed complex, we have very little data that suggests that individual differences in language ability reflect this complexity.

$R_5AzMzLGZTUhhjKt$	Depends who will do this. Research should but not sure feasible at all in everyday clinical/educational practice.
$R_71b9fvukXBUQ5dr$	I don't know what processing is here. Is it a skill? But range of language skills should be assessed.
$R_7WXquZJy8WlgXAx$	yes - however, this does not mean that a preliminary assessment is not possible to idenitfy some concern before an in-depth language assessment is carried out.
$R_1QTm7VrpDX1OAi9$	This is particularly important for planning intervention
$R_8bIXFrv4VBlvVyZ$	But not to the extent that requirements for in-depth assessment preclude starting a child on a path of intervention/support

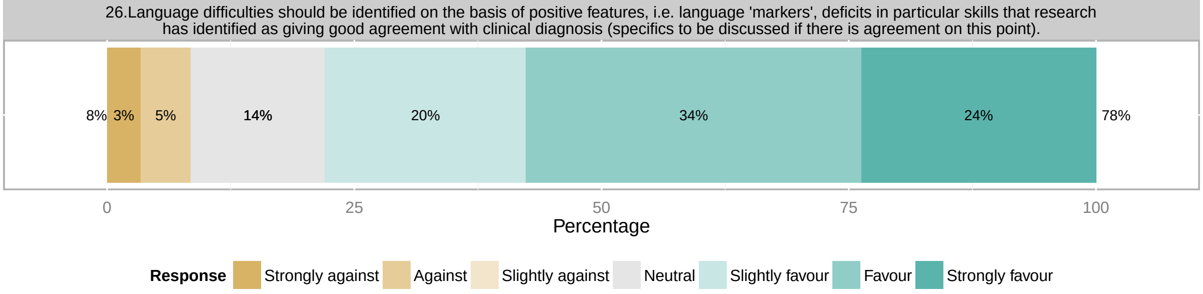

Figure 53: Percentage of panel members in each response category to statement 26. The percentages shown at each end of the scale are the cumulative percentages for the top and bottom three categories respectively.

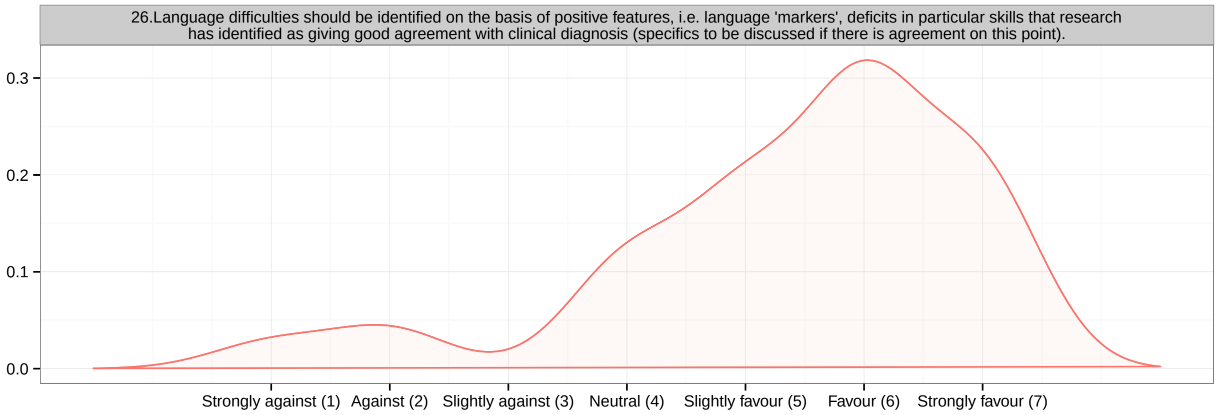

Figure 54: Distribution of responses to statement 26.

Table 26: Comments for each statement.

ResponseID	Q26B
$R_ba8iHG84IJ8cW7X$	I'm afraid I don't understand the comment
$R_2f9ctxaHBJuJdLD$	e.g. Using CCC-2 profiles
$R_6RlkuyWJYcIIsmN$	I am in agreement with this on the proviso that it does not create inadvertent disadvantage for low SES children.
$R_bOrkJKVQ6T8FeGp$	Think further examples here would have helped. Do you mean 'non word repetition' or 'Past tense' markers? If so then I am not sure this is helpful. The markers may have agreement with clinical diagnosis but not specificity for individual cases? Surely individual children just need assessment of the various language domains to look at sympotatology for intervention planning (e.g., receptive/expressive semantics, phonology, syntax—and more specific areas beyond this) and assessment of cognitive processes that are influencing these symptoms - attention, working memory, auditory processing, etc? Not sure how research markers are helpful in a clinical diagnosis?
$R_5cd8BDkYcGfGLKl$	evidence on sensitivity/specificity of markers is mixed and how marker deficits align with functional impairments is unknown.
$R_3VHaciSzwJGKIU5$	providing identification of difficulties is reliable and not a result of test artifact
$R_e9cPjWuFpcer4B7$	I agree if we can identify those that map on to clinical diagnostics but, of course, this prejudges the earlier question. If we don't need researcher and practitioner judgment to come together then this is not an issue. I don't think the record of identifying positive markers has been very successful to date unless you include the extended optional infinitive which has had its critics.
$R_9uJ5LinD5e8X5Yh$	This is a bit SLT techie for me! Not sure I understand the statement. I think I will reiterate my hope for the presentation of the profile of need equals strength areas weak areas and not just in lang areas but also in learning (literacy maths and the rest)

$R_1TXxdyLg1UFCx4V$	Analysis if deficits will also be necessary in order to identify the degree of impact/impairment
$R_eOEFfbvY55KRtRP$	At present I feel that these are rather few in number and are difficult to translate to valid identification without other measures to confirm
$R_dguQPTfUoDzSKB7$	but we should get full profiles as well so we can examine across syndromes and deficits with a variety of etiologies.
$R_cCuacCYZiqQHKgl$	Language deficits should be based on the outcomes associated with language function. We need to work away from viewing language impairment as a property of the child and toward a view of its being a relationship between ability and needed function in our society.
$R_6mrinfsu6CeSmBn$	We would need to adress how to identify chidlren who are only porducign very limited language, as positive features woudl be hard ot identify.
$R_2o7JoTNgC3lqSIR$	This might be helpful if robust markers can be identified. Certainly scores are not always accurate - children might be having an off-day or have too little language for any useful assessment, for example. Clear markers e.g. problems with forming past tenses might well be useful identification tools, and something teachers etc. could look out for.
$R_e5KJQmN6txthTRX$	Yes, if robust markers can be identified. This might be easier and preferable to measuring what children can't do
$R_71b9fvukXBUQ5dr$	If research has in fact done this?
$R_7WXquZJy8WlgXAx$	this would be useful
$R_1QTm7VrpDX1OAi9$	as long as we can find reliable markers.....
$R_3DfMsLnqK54HqcZ$	This is laudable and an important aim towards which we should all strive. However we should not overstate the ability of current 'markers' to identify SLI. If we want to identify LI without excluding any other co-morbid diagnoses then markers like non-word repetition begin to have merit. However they have not been thoroughly tested in POPULATION studies. As Reilly et al note studies of markers have used, almost exclusively, matched cohort designs which artificially inflate their sensitivity. This is a real issue for the field. Inclusion criteria relating to activity and participation limitations experienced by the child may have more merit.
$R_23qAFVuJCo6YHOd$	Markers may be of assistance to help understand underlying issues (e.g. phonological or syntactic), but should not be the basis of diagnosis.
$R_8bIXFrv4VBlvVyZ$	yes current composite standardised assessment batteries are too insensitive and insufficiently informed by assessments that better capture the phenotypic features

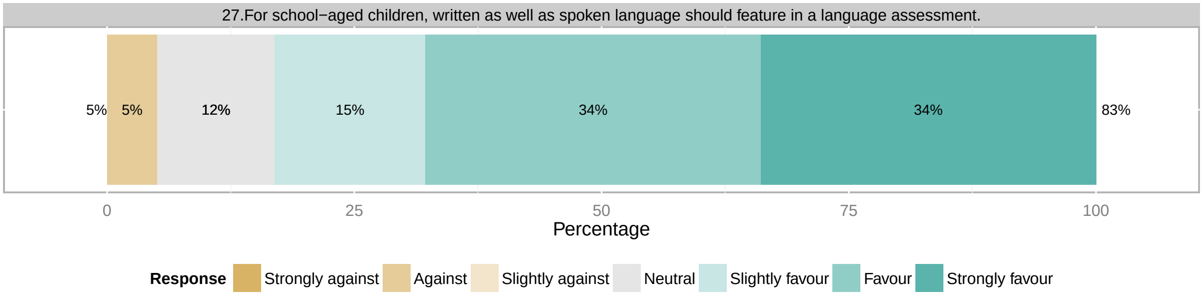

Figure 55: Percentage of panel members in each response category to statement 27. The percentages shown at each end of the scale are the cumulative percentages for the top and bottom three categories respectively.

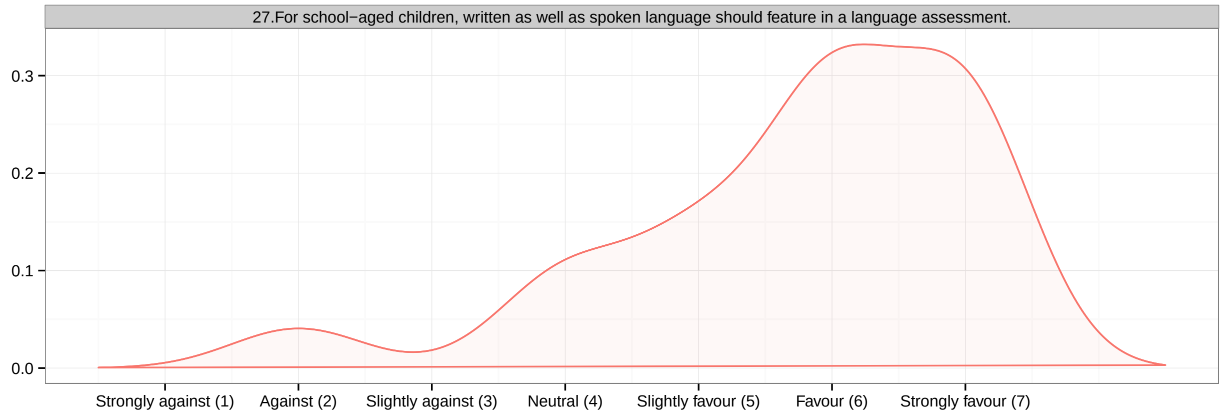

Figure 56: Distribution of responses to statement 27.

Table 27: Comments for each statement.

ResponseID	Q27B
$R_2f9ctxaHBJuJdLD$	Particularly relevant for migrant population where you would expect discrepancies for around 7 years post migration and learning new language
$R_6RlkuyWJYcIIsmN$	I agree with this in a clinical sense, but not necessarily in a diagnostic sense (though having said that most children who fail to meet diagnostic criteria on oral language measures will show evidence of difficulties on written tasks also - probably a moot point!)
$R_6JOosydU46ZndMF$	as well as reading comprehension
$R_5cd8BDkYcGfGLKl$	SLTs are not trained to assess written language and there certainly aren't resources to include reading in intervention. perhaps that should change, but my preference would be to focus on developing oral language skills that will support literacy.
$R_6LIAgEx6sspizpX$	Important for teachers and slts to work together
$R_3sXNbQYRlZaMb3L$	I don't know enough about this. Most of the children I see as an EP have significant literacy difficulties and I'm not sure how much their written work would tell us about their language (vs their fine motor skills).
$R_e9cPjWuFpcer4B7$	Given what we know about the relationship between language and literacy it would be rather bizarre not to make this recommendation.
$R_9uJ5LinD5e8X5Yh$	For older primary and secondary children this should be automatically part of the asst. Particularly where ASD learning features have been recognised (no more the 'gifted child' because s/he can bark at print and spell the dictionary!). For younger learners it is often not as useful.
$R_cYBwzqu4ivWh9qJ$	Unless dyslexia is going to be included as a language impairment

$R_6mrinfsu6CeSmBn$	Only for upper primary / secondary, as it can be used to identify a child's ability to use different 'genres' of language for different purposes, flexibiliyt of langauge use, etc. In general it will just reflect their spoken language...
$R_2o7JoTNgC3lqSIR$	Would this add anything useful?
$R_e5KJQmN6txthTRX$	How would this help?
$R_71b9fvukXBUQ5dr$	The division between teachers and SLTs on this topic is silly.
$R_7WXquZJy8WlgXAx$	I think this can be helpful, but not essential in identifying a language impairment. it is certainly helpful in idenitfying the impacts and implications of a langauge impairment
$R_23qAFVuJCo6YHOd$	The impacts on written language should feature for all. / For older children for example at secondary age, written language difficulties, resulting from language difficulties (not just decoding/encoding) may be the primary concern.
$R_eG1jl51DiHRqXKB$	Writing comes later in development and so it may be difficult to tease apart various components of written language. Some children can dictate but not write even when their motor skills are good. This always puzzles me. There is some emerging literature (I think) on written language impairment.
$R_8bIXFrv4VBlvVyZ$	Yes, assessing spoken language alone misses key aspects of a child's profile of strengths and weaknesses

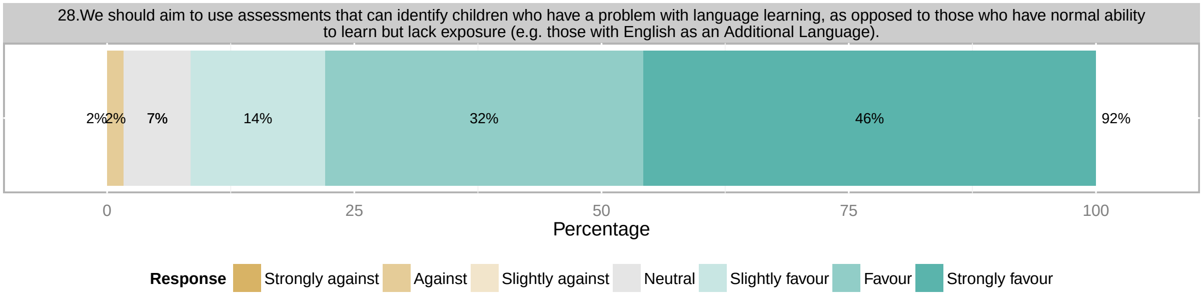

Figure 57: Percentage of panel members in each response category to statement 28. The percentages shown at each end of the scale are the cumulative percentages for the top and bottom three categories respectively.

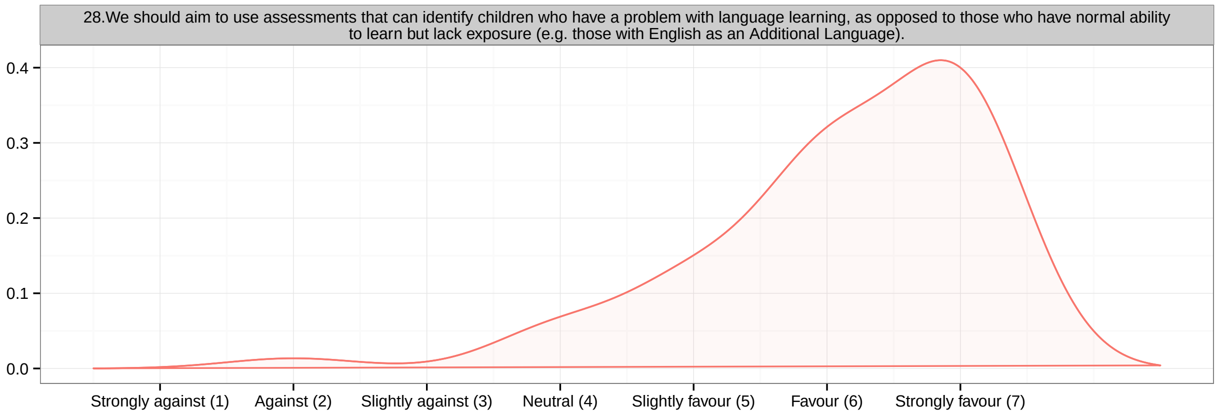

Figure 58: Distribution of responses to statement 28.

Table 28: Comments for each statement.

ResponseID	Q28B
$R_2f9ctxaHBJuJdLD$	Shouldnt we try and identify both if possible? Both may occur of course together.
$R_6RlkuyWJYcIIsmN$	Children with EAL have important needs that need to be met, but coming from a different country/home language should not in itself equate to “disorder”.
$R_bOrkJKVQ6T8FeGp$	Easier said than done.
$R_5cd8BDkYcGfGLKl$	would love to know what assessments those would be!
$R_3sXNbQYRlZaMb3L$	current practice seems to be to wait until the child can be judged to have had sufficient exposure.
$R_e9cPjWuFpcer4B7$	This is easy to say but quite difficult to achieve as Roy and Chiat have recently demonstrated. Dynamic assessment is the obvious solution and this is popular amongst many speech and language therapists and educational psychologists but it is not widely practised and not in a systematic fashion. Also the question presupposes that language is “learned” rather than “acquired” in a nativist sense. The problem with this question is that many who have what looks like an exposure problem - ie they come from very disadvantaged backgrounds, continue to exhibit marked oral language problems after years of schooling. Is this input or processing and how sensitive is this to age? of course children with EAL do not tend to present like this unless they are very disadvantaged in other ways.
$R_cLU7KRGW2XvEql7$	and children from impoverished language environments?
$R_dguQPTfUoDzSKB7$	This is my area of research and it is critical to differentiate them. but it is not easy and there is not one measure that can do this.
$R_6Dvhy7Alhw5wqIR$	age/time points and repeat assessments all need discussion
$R_2o7JoTNgC3lqSIR$	Absolutely - we need to identify those children with a real problem.

$R_7 1b9fvukXBUQ5dr$	Otherwise, interventions offered will probably be too limited.
$R_7 WXquZJy8WlgXAx$	This would be extermely helpful information in planning intervention and school placement particularly
$R_2 3qAFVuJCo6YHOd$	Assessments to identify problems with language learning, but also language processing e.g. formulation difficulties and word retrieval difficulties.

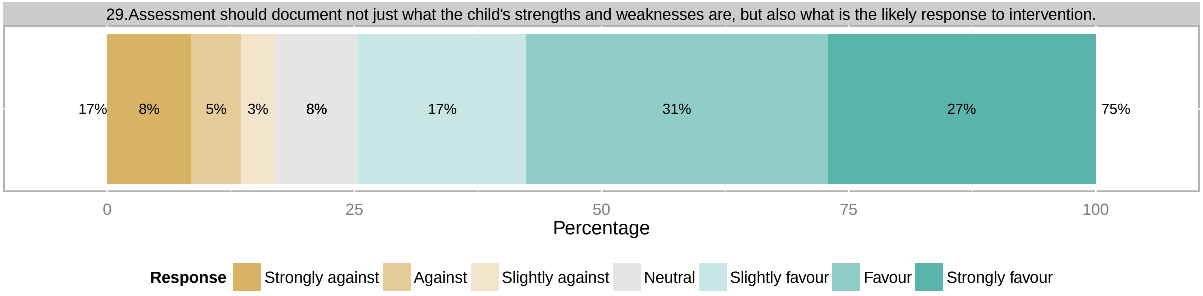

Figure 59: Percentage of panel members in each response category to statement 29. The percentages shown at each end of the scale are the cumulative percentages for the top and bottom three categories respectively.

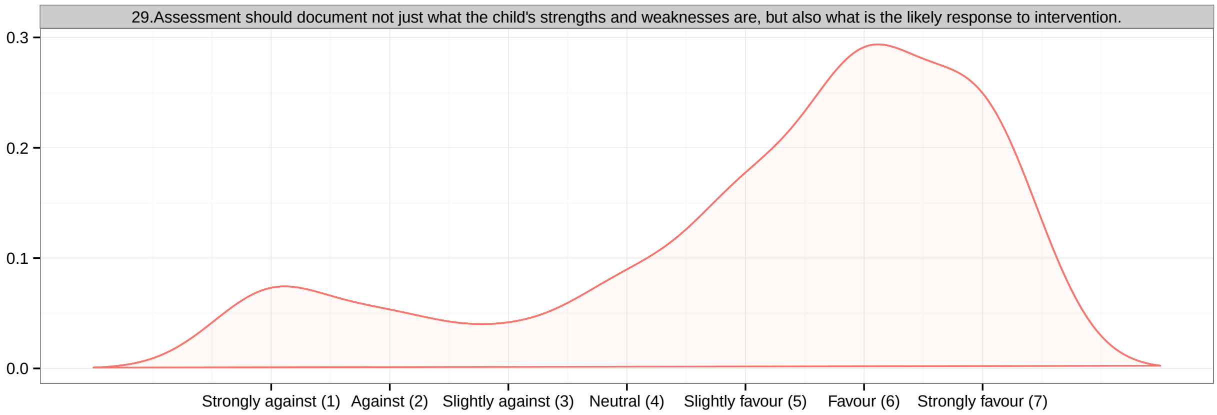

Figure 60: Distribution of responses to statement 29.

Table 29: Comments for each statement.

ResponseID	Q29B
$R_ba8iHG84IJ8cW7X$	The word 'likely' should be deleted. Assessment and further intervention should be based on actual response to intervention.
$R_2f9ctxaHBJuJdLD$	Evidence based outcome research for interventions is important- eg late evaluation of PECS?
$R_6RlkuyWJYcIIsmN$	Agree with this in a clinical sense.
$R_6JOosydU46ZndMF$	this is a 2-part statement - the second part worries me since I am not convinced that we can predict likely response to intervention
$R_bOrkJKVQ6T8FeGp$	Apologies for repetition, but this is easier said than done. How do we determine this? Different clinicians have differeing abilities and so would that mean that a child who has a dreadful therapist may not respond to the applied intervention and then goes into a 'unlikely to improve' basket... dangerous. I see you have addressed this below - indeed we don't have robust tools yet to do this that would take the clinician variable out of the equation! I would say the same for a lack of assessments for ESL vs 'real' language impairment.
$R_5cd8BDkYcGfGLKl$	too many unknowns there - what intervention? for how long? at what intensity? response to intervention is as much about the service/SLT as it is about the child's capacity.
$R_3sXNbQYRlZaMb3L$	I find the response to intervention ethically problematic, because children who are judged unlikely to respond are denied services.
$R_e9cPjWuFpcer4B7$	I agree with this but this begs the question if whether they should be treated if they look like they are not going to respond to intervention. Nonetheless the idea that different interventions should be activated at different levels and the children's responses carefully monitored and used to inform further intervention is one that should be taken seriously. RTI is technically used in the US but with rather mixed results,

$R_9uJ5LinD5e8X5Yh$	Crystals balls are difficult to find these days! If the asst has been rigorous and by skilled professionals then maybe we could hedge around and predict pace of remediation........but woe betide those who get it wrong! We would only make a hint of a suggestion as to this after working with the child for at least a year (full time in the school).The same presentation of need in 2 children can have very different rates of progress.
$R_cLU7KRGW2XvEql7$	Dynamic assessment is under used as a clinical tool
$R_es7hPPlfD7bdd65$	I don't think we know how RTI interacts with individual differences, and this may in fact differ not just measure-by-measure but interact such that some kids are less likely to RTI because they have a certain constellation of deficits. (and I see q30 basically gets at this!)
$R_0ofhSCmeppIQ8kt$	But they may not be the same measures....
$R_cCuacCYZiqQHKgl$	We should not define any form of human health on the basis of our ability to treat it; however, it seems reasonable to attempt to make prognostic statements.
$R_4HGIGYFIvMxLWcJ$	There is so little evidence, that we may not be able to do this.
$R_6mrinfsu6CeSmBn$	If only there were more dynamic assessments!!!
$R_2o7JoTNgC3lqSIR$	This would risk making assumptions about children, that might not turn out to be accurate. However, ongoing assessment should take account of how children have responded to intervention and adjust accordingly
$R_e5KJQmN6txthTRX$	Assessments should not pre-empt what might happen. Children respond to therapy in different and often unpredictable ways
$R_71b9fvukXBUQ5dr$	But hard to do - no sensitive predictors yet (beyond progress itself).
$R_7WXquZJy8WlgXAx$	a dynamic approach the assessment would provide this
$R_1QTm7VrpDX1OAi9$	I wish we could, but I don't think we have that knowledge at the moment
$R_3rrKtkb2VvC3uG9$	Intervention science or practice is not robust enough to make this judgment of likely response to intervention
$R_3DfMsLnqK54HqcZ$	Again a laudable aim but do we really have the tools to do this given current knowledge?
$R_23qAFVuJCo6YHOd$	Ideally yes, assessment should identify the type of intervention needed. We should work towards more of this being done by trialling the intervention/ carrying out dynamic tasks at the assessment stage. I am not sure if documentation of response to intervention is needed for the 'diagnosis' (and therefore this Delphi) but it is needed for identifying the provision needed for an individual.
$R_eG1jl51DiHRqXKB$	This is a good question. I am not aware of research on this topic.
$R_8bIXFrv4VBlvVyZ$	I don't believe we yet have tools and the knowledge to be definitive on this issue-we do not well understand the longitudinal trajectories of development and response to itnervention based on particular profiles/dimensions of difficulty; intervention approaches have yet to catch up with current theories of language acquisition; I am concerned that including likely response to intervention as a feature early on could limit children's access to intervention;

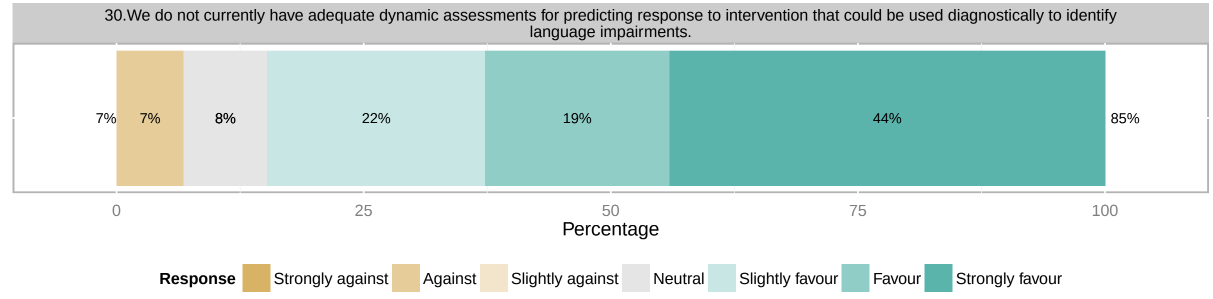

Figure 61: Percentage of panel members in each response category to statement 30. The percentages shown at each end of the scale are the cumulative percentages for the top and bottom three categories respectively.

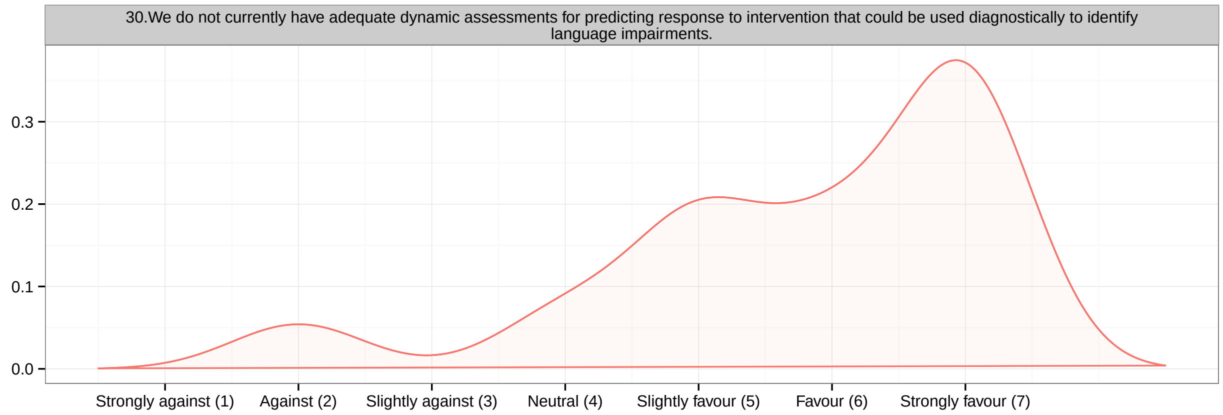

Figure 62: Distribution of responses to statement 30.

Table 30: Comments for each statement.

ResponseID	Q30B
$R_2f9ctxaHBJuJdLD$	A response to a particular treatment does not necessarily imply a particular diagnosis
$R_6RlkuyWJYcIIsmN$	Lack of good downstream tools should not preclude us from trying to get things right upstream. Once the downstream tools improve, we might use that knowledge to inform diagnostic boundaries.
$R_6JOosydU46ZndMF$	But can response to intervention be used validly & reliably to be used diagnostically - across development?
$R_e9cPjWuFpcer4B7$	DA and RTI are not in my opinion the same thing. We do have DA procedures but whether they are “adequate” is a bit of a mot point. They can be used to distinguish those who do respond to scaffolding but you are still left with a problem as to why the others did not. RTI is more about intervention at different levels rather than specific assessment procedures.
$R_9uJ5LinD5e8X5Yh$	Can we not assess for SLI without predicting response to interventions? I think we can........... (book 4?)
$R_bwwc7dPFEcp1azH$	But we do know that some form of test-treat-test format in the assessment and diagnostic process can give us valuable information about the child’s language learning potential and many clinicians/researchers do not include dynamic assessment as part of their path to diagnosis
$R_cLU7KRGW2XvEql7$	this misses the point. Dynamic assessment is a technique not a box of tricks, so if we are trained properly to do dynamic assessment and mediated learning we can do it in all circumstances
$R_cCuacCYZiqQHKgl$	Again diagnosis should not be based on the potential or the actual response to intervention. Ironically, a positive diagnosis in RTI as practiced, represents the inability to respond to intervention.
$R_6mrinfsu6CeSmBn$	see above
$R_2o7JoTNgC3lqSIR$	I believe this to be true

$R_e5KJQmN6txthTRX$	Dynamic assessment should be part of a full assessment process
$R_71b9fvukXBUQ5dr$	Nothing yet - promising developments?
$R_7WXquZJy8WlgXAx$	we need more, and more for older children and young people that can be adminstered by no specialists as well as specialists
$R_1QTm7VrpDX1OAi9$	see above
$R_8bIXFrv4VBlvVyZ$	yes those we have are not widely used, and capture only particular aspects of lnaguage impairment and development

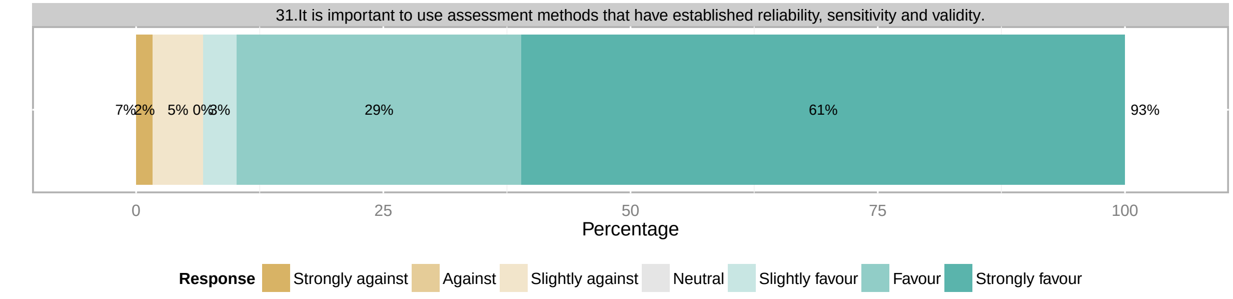

Figure 63: Percentage of panel members in each response category to statement 31. The percentages shown at each end of the scale are the cumulative percentages for the top and bottom three categories respectively.

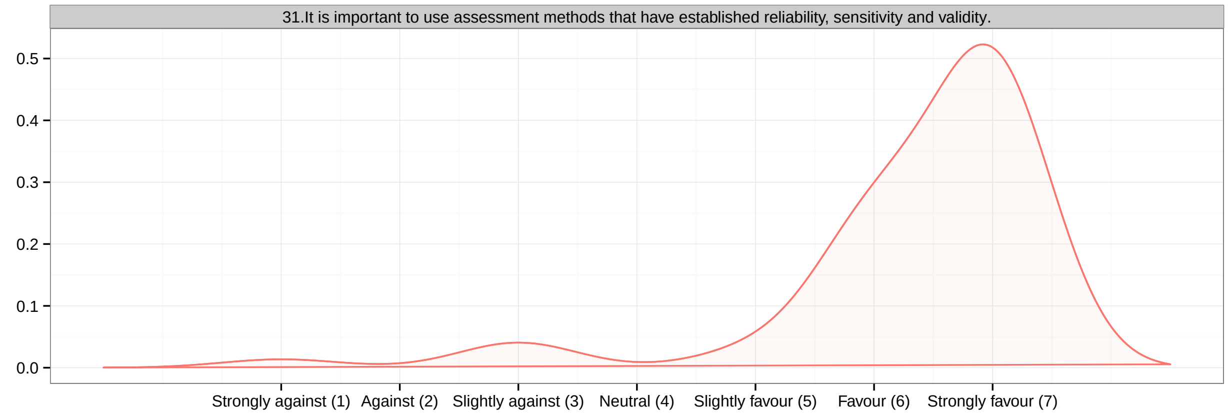

Figure 64: Distribution of responses to statement 31.

Table 31: Comments for each statement.

ResponseID	Q31B
$R_b a8iHG84IJ8cW7X$	But not necessarily only methods that have established reliability, sensitivity and validity.
$R_2 f9ctxaHBJuJdLD$	Motherhood and apple pie...
$R_5 cKMfR48zQytYc5$	yes but we keep restandardising our language assessments
$R_6 JOosydU46ZndMF$	and have cultural sensitivity, and evidence of reliability, sensitivity, validity across the developmental span
$R_b OrkJKVQ6T8FeGp$	Indeed - the range of clinical abilities needs to be taken into account somehow and this helps...
$R_e 9cPjWuFpcer4B7$	This is a poor question. It should be separated out into its component parts and it needs to be clear about what is meant by sensitivity. is this sensitivity to identification (specificity and sensitivity) or sensitivity to change? Researchers like more formal standardised tests because they allow them to make strong statements about their results. Unfortunately they also use them with very young children (eg. 2 years olds|) even though we know that children do not respond for a variety of reasons of which inability to answer the question is only one. Practitioners often do not use them in a formulaic way or at least not in a way that they were necessarily intended. They often prefer criterion referenced tests which are able to make more use of the context and may be more sensitive to change. So the answer to this question depends on who it is referring to.
$R_9 uJ5LinD5e8X5Yh$	As well as the professional judgements of staff for the bits around the edges of lang asst - eg ASD features
$R_b wwc7dPFEcp1azH$	But few exist, particularly with norms for non-UK/US children
$R_c LU7KRGW2XvEql7$	so ruling out observation, criterion referenced and dynamic assessments then? if we use standardised tests they should be fit for purposed though.

$R_es7hPPlfD7bdd65$	This should go without saying of course.
$R_6mrinfsu6CeSmBn$	This would exclude groups of chidlren from getting a diagnosis, ie EAL children for whom these assessments do not exit.
$R_5AzMzLGZTUhhjKt$	If doing standardised assessments then yes but if assessing for intervention purposes then no - depends on the purpose of the assessment.
$R_71b9fvukXBUQ5dr$	Otherwise, making it up.
$R_7WXquZJy8WlgXAx$	as well as other ways of gathering information
$R_1QTm7VrpDX1OAi9$	Yes for diagnosis, allthough informal assessments may be more useful for planning intervention.
$R_23qAFVuJCo6YHOd$	I agree these should be part of the assessment, however: / - There are gaps in measures available in pragmatics. / - Qualitative information can be the most useful - for example a sample of the child's language or difficulties experienced in the classroom. Sometimes standardised available language measures do not show the severity in real life. / - There are limited measures for considering impact of the difficulties.
$R_eG1jl51DiHRqXKB$	I agree with this statement. But at the same time clinical experience and wisdom is also a critical factor.
$R_8bIXFrv4VBlvVyZ$	Yes to the extent possible, we sould included valid, reliable and sensitive tools but we have few if any that are high on all dimensions and so I would not favour this as a recommendation in a consensus statement on the assessment, diagnosis of LI

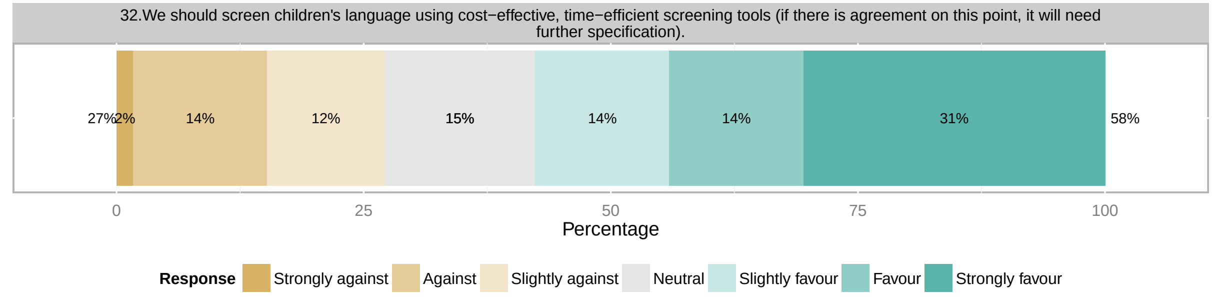

Figure 65: Percentage of panel members in each response category to statement 32. The percentages shown at each end of the scale are the cumulative percentages for the top and bottom three categories respectively.

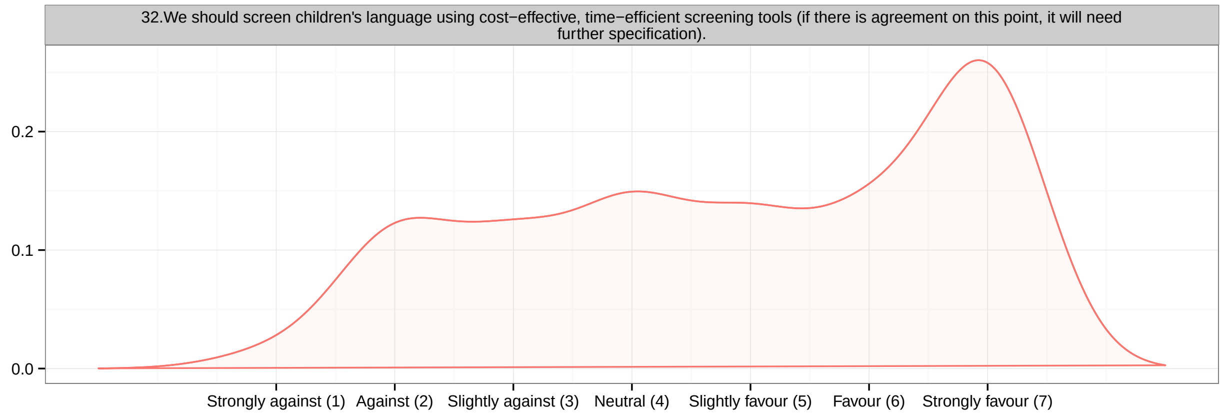

Figure 66: Distribution of responses to statement 32.

Table 32: Comments for each statement.

ResponseID	Q32B
$R_b a8iHG84IJ8cW7X$	I'm slightly wary of the use of widespread use of screening.
$R_2 f9ctxaHBJuJdLD$	Translation of tools to the front line hard pressed clinician is a real need
$R_5 cKMfR48zQytYc5$	there is no evidence that we have reliable or valid tools not that we will pick up kids who go onto to have LI. currently if we did this we would waste resources
$R_6 JOosydU46ZndMF$	Screening is a very complex issue - think of medical screening practices that are now coming under close scrutiny (mammography, PSA, health check-ups etc)
$R_6 JZKVRyNZK6U0zX$	My view here could change if better screening tools come along.
$R_5 cd8BDkYcGfGLKl$	there are no screening tools that can do the job adequately. there are many issues around this that need exploring...

$R_e9cPjWuFpcer4B7$	Again this is not a great question because it does not explain its terms and includes a variety of different issues to comment on."cost effective and time efficient" are complex ideas because they are dependent on the costs to society of not identifying children and they are also sensitive to whether intervention is effective. / / As a series of systematic reviews have indicated there are no screening tests at a population level that will do this. The ASQ is due to be rolled out across the UK later this year on the grounds that this is the best measure available with the tightest age bands. In the US "screener" is used in a rather different way and can be what the specialist professional does in school, for example and can constitute standardised language measures. So again this is a poor question because it assumes that "screening" is being used in one way when it isn't. / / One of the key problem with screening is that it assumes that children's language trajectories are consistent. This position is untenable in the early years at least because we know that they fluctuate and that natural history is difficult to predict in the population (whether representative or clinical). / / The solution to this is to consistently monitor a range of children over two time points. This has not been explored effectively and should be a focus for future research
$R_9uJ5LinD5e8X5Yh$	I would need to better understand your definitions of 'cost effective' and 'time efficient' to really make a valid comment. We don't need the reams of reporting as per the private SLT assts but we do need more than 1 asst by the SLT -eg the TROG and nothing else.
$R_1TXxdyLg1UFCx4V$	The process for screening for disorders is complex and there are health departments who investigate the efficacy and desirability of screening measures. NICE guidance has not recommended this. Screens for neurodevelopmental disorders have not been broadly adopted in the UK because of lack of specificity and sensitivity. Health Visitor checks for levels of development across a range of domains is likely to be more useful as well as rapid response to parental or professionals concerns about a child's progress
$R_3pDedyU4fM1kOXj$	We should only do this if the 'cost effective and time efficient tools' are also evidence based and have established reliability, sensitivity and validity.
$R_bwwc7dPFEcp1azH$	Although pathways for intervention need to be part of this screening as particularly at a young age many false-positives will be identified; we also need valid and reliable screening tools
$R_6Dvhy7Alhw5wqIR$	this is very much about age and I support first year at school assessment
$R_4HGIGYFIvMxLWcJ$	This does need further clarification. Does it imply universal screening?
$R_2o7JoTNgC3lqSIR$	If it is possible to accurately screen all children at, say 3 years, then we should. However, it is our understanding that there are no tools currently that are able to do this accurately enough.
$R_e5KJQmN6txthTRX$	But I'm not aware of any such tools that are sufficiently robust at the moment
$R_71b9fvukXBUQ5dr$	Criteria are sensitivity and specificity: time and cost are secondary (quick is good, but not quick and dirty).
$R_1QTm7VrpDX1OAi9$	Yes, if we can, but I don't think we are at that point at the moment.
$R_ebTqVBlGUNh60eN$	differential diagnosis requires more than a screen assessment as to which cases are likely to persist.
$R_3DfMsLnqK54HqcZ$	Screening is currently not indicated as we don't have the right tools - rather we should be looking to assessing risk and using public health approaches which allow children to receive preventative interventions when they are at risk of persisting difficulties. This is often not indicated by the child's level of language abilities but rather by other risks (e.g. family history, parenting practices)
$R_23qAFVuJCo6YHOd$	I am not sure if this means screening all children's language or using a screen when there is a concern about a child.
$R_eG1jl51DiHRqXKB$	This would good and is a practice in some kindergarten/Grade 1 classes do this. But I also agree with needing more specifications and tools.
$R_8bIXFrv4VBlvVyZ$	Waiting to identify such tools could delay the field further; when knowledge to date suggests too many parameters for inclusion in terms of risk factors/markers

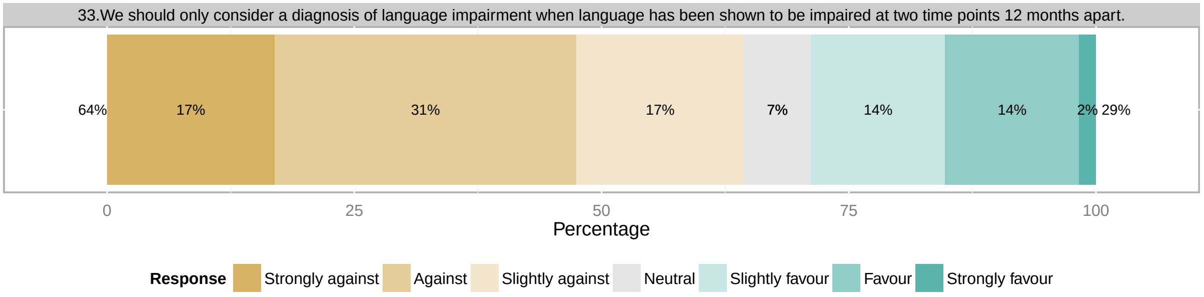

Figure 67: Percentage of panel members in each response category to statement 33. The percentages shown at each end of the scale are the cumulative percentages for the top and bottom three categories respectively.

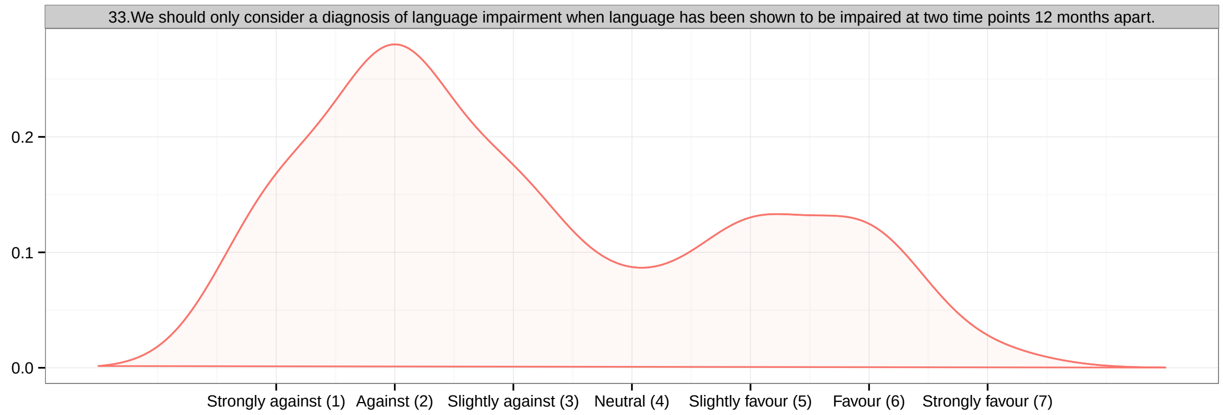

Figure 68: Distribution of responses to statement 33.

Table 33: Comments for each statement.

ResponseID	Q33B
$R_2f9ctxaHBJuJdLD$	Makes it more reliable but how practical and wouldnt 6 months be better
$R_6RlkuyWJYcIIsmN$	Twelve months is a long time in the life of a young child! missed opportunities for early intervention. Clinicians can decide if a false positive has occurred and terminate treatment if need be.
$R_5cKMfR48zQytYc5$	yes but this is only relevant to the preschool years 2-4 when expressive language only is delayed
$R_6JOosydU46ZndMF$	why 12 months - depends on developmental stage & chronological age of the child
$R_6JZKVRyNZK6U0zX$	I've not heard this one before!
$R_bOrkJKVQ6T8FeGp$	I am against here if we are talking about children with obvious issues such as those with genetic syndromes where we know that the child is likely to have a persisting impairment, being unable to overcome the neurogenetic vulnerabilities easily. I don't think such children or families should have to wait. I do think this could have a dire consequence on the child's access to services and would be a step backwards. Again, this is where I think this survey would benefit from us tackling the issue of whether we are talking about SLI only in some of these questions, or ALL children with ALL aetiologies that may have language as a symptom and/or as part of a syndrome...if you believe such things are dissociable.
$R_5cd8BDkYcGfGLKl$	why 12 months? would age of child at point of assessment matter?
$R_6LIAgEx6sspizpX$	Depends on the age of the child and what the presenting difficulties are
$R_0Gj2hZlxlaPtHbT$	discussion about assessment frequency
$R_e9cPjWuFpcer4B7$	Yes, I think that this is a solution given the variability of language across time.

$R_9uJ5LinD5e8X5Yh$	Depends when the 2 time points are: we wait to assess a child until 4 years old as before then gaps can quickly eradicate themselves. If the scoring is very low thereafter it is likely that that is what there is. For learners with only small AE gapping then maybe 2 times assts are needed. The age of the child and the degree of deficit is more important for us. Waiting for another 12 months could be a bad asst model to set up.
$R_3pDedyU4fM1kOXj$	I think that 12 months is too long of a period in a young child's life. I think that 3-6 months is a more appropriate time.
$R_bwwc7dPFEcp1azH$	Although if this was between the age of 2 and 3, there could still be a lot of issues with over and under-identification. A cut off of still displaying features at age 4 (school entry) could be applied or a period of some intervention (targeted?) applied to determine response to intervention
$R_0ofhSCmeppIQ8kt$	But Tier 2 supports could still be put in before the second assessment...and maybe even Tier 1, if it were available...?
$R_4HGIGYFIvMxLWcJ$	12 months may be too long to wait and see it that is the result of this statement
$R_6mrinfsu6CeSmBn$	I would be concerned that this may result in children starting school without appropriate support because they were identified late... and children not being able to access specialist support in the early years also because there isn't time to identify them...
$R_2o7JoTNgC3lqSIR$	This would risk delaying intervention. In our experience it is not usually that difficult to identify a child who needs help. However, if the situation is uncertain, there is no harm in talking about a language delay and intervening to see if this can be overcome, and then giving a more formal diagnosis if not.
$R_e5KJQmN6txthTRX$	This is a long gap and seems unduly prescriptive
$R_71b9fvukXBUQ5dr$	Would take ages to get a diagnosis (although could confirm this way).
$R_7WXquZJy8WlgXAx$	I think this is unrealistic in todays busy and under resourced environment
$R_1QTm7VrpDX1OAi9$	I think this depends on age. Yes, definitely for pre-schoolers, and those with borderline difficulties, but for a school-aged child with severe difficulties, one assessment point should be sufficient to trigger services even if not a definite diagnosis.
$R_ebTqVBlGUNh60eN$	I would prefer to make a reliable diagnosis through thorough assessment of clinical markers than simply wait and see. There should be no excuse for delay in treatment if that case has been identified as being likely to persist.
$R_3DfMsLnqK54HqcZ$	As language development is so variable this would seem appropriate, certain in the pre-school years. But only if this is set within clear clinical guidelines where a diagnosis is NOT required for intervention to be offered. So a public health approach with staging of interventions for children at risk is essential
$R_eG1jl51DiHRqXKB$	Sometimes children do "outgrow" a language impairment. It is important to assess the consistency of impairment. Also depends on the age of the child. It also has been shown that some children whose language impairment seems to have been resolved, later show problems with higher order language tasks.
$R_8bIXFrv4VBlvVyZ$	I'm in favour of avoiding diagnosis of LI too soon given the fluid trajectory of language development, particularly in younger children. But we have to allow the possibility of identifying a child, on first assessment, where they are afforded robust asseessment and where there is clear information on risk factors (history and observation) and who demonstrate impairment on clinical marker tasks
$R_8AhxnQPe8mJkUoR$	Not in the context of parental and teacher concern as well as, for example, the language impairment affecting interaction with family and peers. A whole year is quite a lot of developmental time for a four year old.

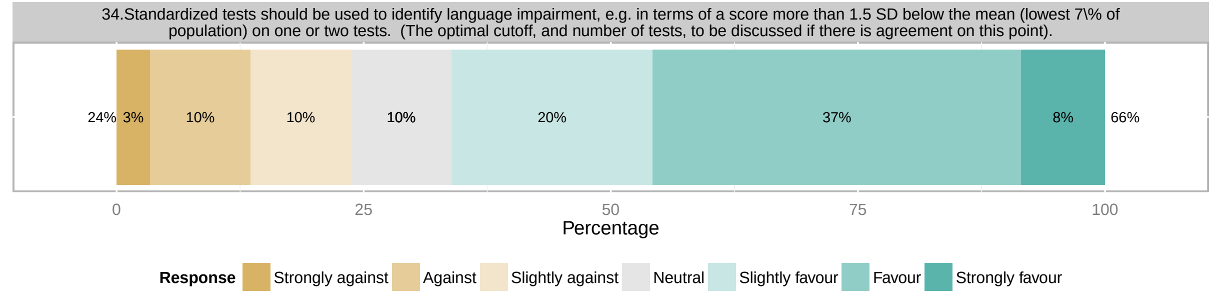

Figure 69: Percentage of panel members in each response category to statement 34. The percentages shown at each end of the scale are the cumulative percentages for the top and bottom three categories respectively.

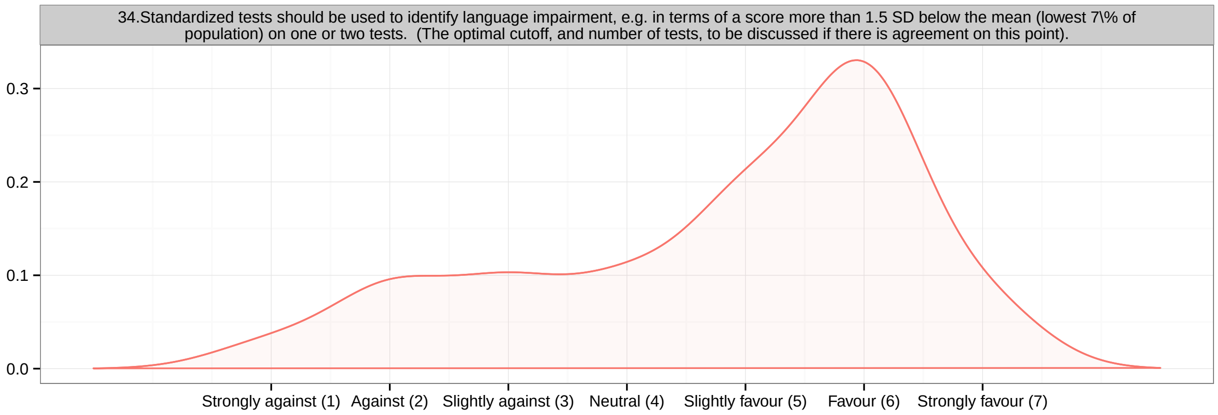

Figure 70: Distribution of responses to statement 34.

Table 34: Comments for each statement.

ResponseID	Q34B
$R_6JOosydU46ZndMF$	I stringly suspect that diagnosis will remain a clinical decision - no single score will be sufficient . Any cut-off is arbitrary and scores are test-dependent, being determined by the range and focus of test items
$R_6JZKVRyNZK6U0zX$	Yes, but they should be supplemented with other, more ecologically valid forms of assessment.
$R_bOrkJKVQ6T8FeGp$	The problem being an absence of good standardised tools for children who are largely non verbal at older ages.
$R_5cd8BDkYcGfGLKl$	The cut is arbitrary and I think it is important to discuss how various cuts map onto functional impairment. Having said that, standard measures give the profession credibility and a provide a baseline for measuring change. The move away from child assessment is a dangerous one in my opinion.
$R_e9cPjWuFpcer4B7$	Really we should use the threshold below which the natural history is most clearly defined and which will lead to persistent problems if intervention is not provided. These threshold are arbitrary and are likely to be sensitive to “service availability prevalence”. This is not the right way of doing this from an epidemiological perspective and for my money the sooner we see this as an epidemiological question the better.
$R_9uJ5LinD5e8X5Yh$	On which test/s though? A child can score AE on one test and very low on another. In general there may have to be agreement on this - it could be helpful, but all SLTs and therefore LAs would need to support the agreement. Work to be done!
$R_3pDedyU4fM1kOXj$	I do not think that you need a standardised test to identify language impairment.... in some cases they can be helpful but I do not feel that they need to be used in order to give the diagnosis.

$R_b wwc7dPFEcp1azH$	They should be included, but only as one aspect of the overall diagnosis
$R_0 ofhSCmeppIQ8kt$	This might be one, but not the only criteria...how many criteria does the child need to meet to have an LI?
$R_6 Dvhy7Alhw5wqIR$	It is one of the criteria but functional impairment also needs to be discussed as do parental/other views
$R_c CuacCYZiqQHKgl$	Standardized tests should be used where possible; however, these need to be complemented by other information.
$R_4 HGIGYFIvMxLWcJ$	Yes, but it should not be the only test - we need to consider the child's functioning and language skills at discourse level
$R_6 mrinfsu6CeSmBn$	What will happen to EAL children?
$R_2 o7JoTNgC3lqSIR$	It is not always necessary to carry out a formal assessment to identify language impairment - sometimes it's obvious. However, formal assessment is useful for getting a full picture and planning intervention. A cut-off of 1.5 SD below the mean seems high to us. Not all children at this level will require SLT, or not much at any rate, and in the UK certainly probably would not get it. Our concern is that the children with clear clinical needs - those below 2 SD below the mean - do get the specialised support they need.
$R_e 5KJQmN6txthTRX$	This score seems a little high. Why suggest this level? is it for research. It may not help children in practice if too many are drawn into the net, and if some of these speak fairly well, it minimises the impact on those with real problems
$R_7 1b9fvukXBUQ5dr$	Should know deviation scores - but cut offs may vary for different purposes and what consitutes a test is tricky - a composite like CELF is perhaps more powerful than a single-aspect measure like BPVS.
$R_1 QTm7VrpDX1OAi9$	But not on their own. Also need measure of functional effects on education and life in general. Also pragmatic skills
$R_1 z8h1XMT676UOwd$	While desirable there is a problem with the broad cultural applicability of some tests.
$R_e bTqVBlGUNh60eN$	but not in isolation, use of other means are vital too. We need to be able to assess language learning ability rather than a snap shot of language knowledge at a particular time.
$R_3 DfMsLnqK54HqcZ$	I agree - but with caveats - so if this forms part of an assessment which also looks at activity and participation limitations and response to intervention and if it is an omnibus language measure, then this is can be a useful characterisation of the impairment part of the ICF framework of the persons disability. Also children with EAL would need a different approach based on their exposure to English.
$R_e G1jl51DiHRqXKB$	I basically agree but functional language also needs to be taken into account, i.e., pragmatics
$R_8 bIXFrv4VBlvVyZ$	I'm in favour of this as a parameter but only where accompanied by other assessments (of clinical markers, history) and by parental/school/other relevant concerns

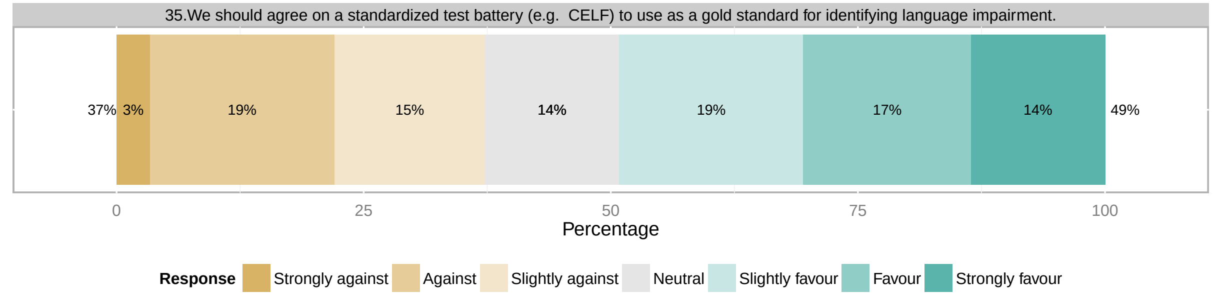

Figure 71: Percentage of panel members in each response category to statement 35. The percentages shown at each end of the scale are the cumulative percentages for the top and bottom three categories respectively.

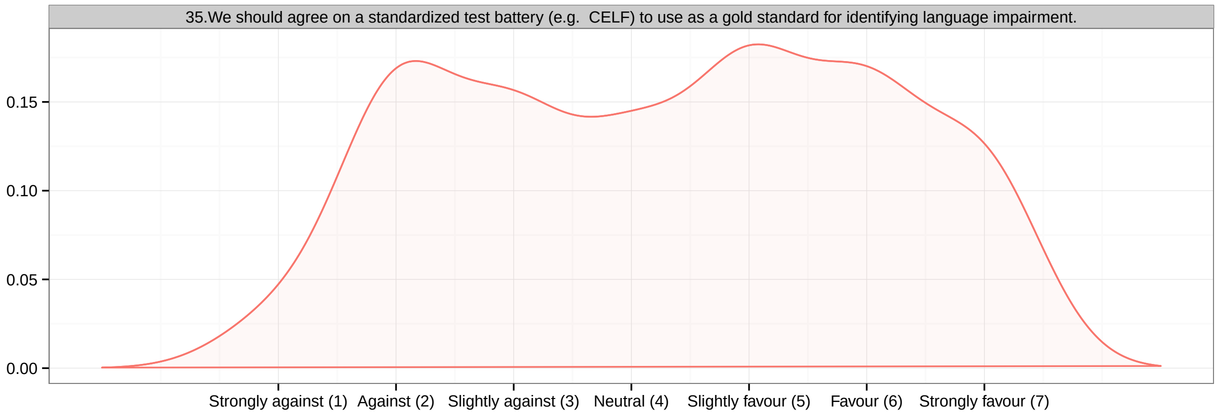

Figure 72: Distribution of responses to statement 35.

Table 35: Comments for each statement.

ResponseID	Q35B
$R_2f9ctxaHBJuJdLD$	A range would be helpful- as in ASD diagnosis plus clinicians judgement too
$R_5cKMfR48zQytYc5$	a set of criteria might be more important than a specific test
$R_6JOosydU46ZndMF$	THis would need considerable discussion - my personal opinion is that so many current language tests involve other cognitive abilities (e.g. working memory, visual-spatial abilities et), so that relying on any one commercial tool is likely to be problematic. Standardized tests are an important component of assessment but will be insufficient in themselves
$R_cIxZunCo2wnTfVj$	Would be useful to have common instruments that were agreed upon as one component of assessment/diagnosis, but standardized tests alone are insufficient.
$R_6JZKVRyNZK6U0zX$	Not possible the moment, give the dearth of such tests outside of the US and UK.
$R_5cd8BDkYcGfGLKl$	I think a core battery would be useful to researchers and clinicians alike (I doubt it will be CELF though - horrible test)!
$R_3VHaciSzwJGKIU5$	given the different presentation and the limitations of the tests it is important for practice to use a systematic approach to investigating the child's profile of needs - one single test is unlikely to be able to meet this criterion
$R_e9cPjWuFpcer4B7$	There are good reasons for doing this and it will be very helpful for researchers. I am not convinced that this will help practitioner plan interventions. So it depends what the point of this would be. We need to be mindful here that there are differences in recommending tests that are robust and those that are good at measuring change. Trials suggest that tests like the CELF, constructed as they are like IQ tests are relatively robust and are good for the ICC as a means of doing power calculation but do not measure change well. Subtests are probably more appropriate.

$R_9uJ5LinD5e8X5Yh$	Oh what a delight this would be! But all batteries of the chosen asst would need to be administered and reported. Jubilation if it could happen ....a level (ish) playing field. Would the necessary aspects be automatically gathered eg speech sound knowledge, word finding difficulties? Maybe we could make the gold standard asst tool from scratch - but stealing bits of other tests - and 'if the child has no speech production problems do not assess with Test D' etc etc. Exciting!
$R_1TXxdyLg1UFCx4V$	We will need a range of tests or testing protocol that takes into account the context of the language impairment i.e. deafness, learning disability etc. CELF will not do this
$R_dguQPTfUoDzSKB7$	often the standardized measure is not adequate especiallywhen they are english learners.
$R_0ofhSCmeppIQ8kt$	We could maybe have a 'common' approach that is used for a majority, but clinicians need the latitude to use other measures....
$R_dmR80BQCC0tAFuZ$	open to sample bias in standardisation process
$R_4HGIGYFIvMxLWcJ$	universally?
$R_6mrinfsu6CeSmBn$	What will happen to EAL children? It woudl need to be standardised on EAL populations before this could be applied.
$R_2o7JoTNgC3lqSIR$	There are strong arguments for using CELF or similar assessments for diagnosis - indeed we would normally expect this. But to identify?? / We are concerned that none of the questions so far really seem to take account of the terrain in the UK, where the majority of children with SLCN will have little contact with SLT services. Indeed, identification and support is expected to be done by teaching and early years staff - how and where does this fit in?
$R_e5KJQmN6txthTRX$	For diagnosis yes. Identification is something else. It is not generally SLTs who 'identify' language impairment. In the UK at least, this would be parents, teachers and early years staff.
$R_71b9fvukXBUQ5dr$	CELF is not a battery - but if a battery could be constructed, great. Hearing measures would be a good model.
$R_7WXquZJy8WlgXAx$	This depends on the agreed criteria for language impairment. and may limit practitioners ability to be able to identify. prefer a range or type of assessments
$R_1z8h1XMT676UOwd$	One test alone, even a very good one, may not identify all children with significant language impairment. A broader assessment approach that also addresses limitations in everyday life participation is recommended.
$R_ebTqVBlGUNh60eN$	Diagnosis and service access often ends up being reliant on a set of scores which is not appropriate.
$R_3rrKtkb2VvC3uG9$	Although we have multiple options for robust language assessment; there is no reason to restrict the choice to a single one.
$R_23qAFVuJCo6YHOd$	Wider measures may be needed, including measures at different ages.Language processing is too complex to pick up the potential severity of difficulties through one test battery.
$R_eG1jl51DiHRqXKB$	No one test should be used but obviously some tests are better than others. Some tests have subtests that are better or worse than others.
$R_8bIXFrv4VBlvVyZ$	There is a role for a robust standardised assessment, with a recommendation on parameters to be covered, but we should not favour one particular tool and that test should be accompanied by other information.
$R_8AhxnQPe8mJkUoR$	This may be helpful to the field as such an approach has worked somewhat for autism. However, what specific standardized test battery is the real issue as there are a number of problems with most.

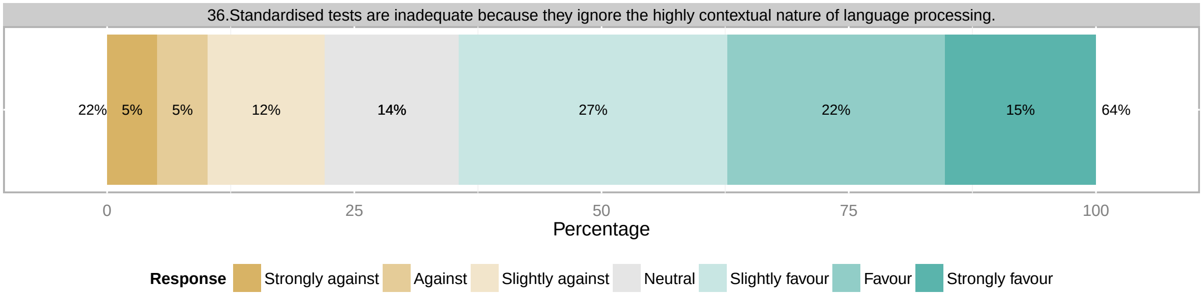

Figure 73: Percentage of panel members in each response category to statement 36. The percentages shown at each end of the scale are the cumulative percentages for the top and bottom three categories respectively.

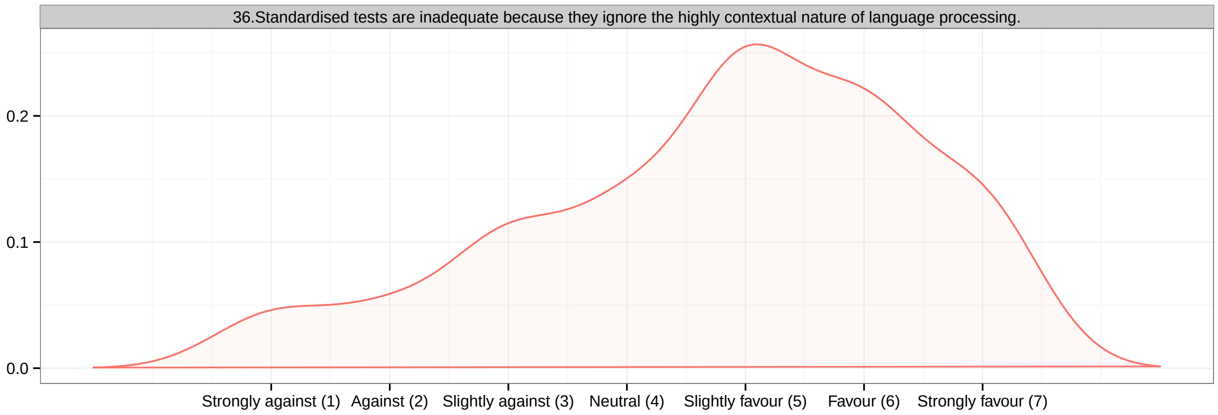

Figure 74: Distribution of responses to statement 36.

Table 36: Comments for each statement.

ResponseID	Q36B
$R_6RlkuyWJYcIIsmN$	There is always a risk that children whose "structural" language skills are within normal range will be missed if pragmatic skills are not measured/reported on as well as scores on formal language tasks (vocab, syntax, morphology etc).
$R_6JZKVRyNZK6U0zX$	They are inadequate only if used by themselves as the sole means of assessment.
$R_bOrkJKVQ6T8FeGp$	Agree with this yet we have to start somewhere.
$R_5cd8BDkYcGfGLKl$	yes, they don't get at discourse/pragmatics and that is a valid point.
$R_9U2zxMLVAPcvQUd$	They are "necessary but not sufficient"
$R_e9cPjWuFpcer4B7$	Yes this is the point made above. it would be helpful to have practitioners views on this specific point.
$R_9uJ5LinD5e8X5Yh$	You can replicate contexts if you are savvy! We now always do the CELF understanding paras / concepts and directions as we find that some children 'survive' the assts where there is visual support of any kind.
$R_1TXxdyLg1UFCx4V$	We need a protocol with decision trees to help navigate these issues. There is a need to have a standardised procedure for assessment to properly identify the level of need and to measure progress against stated aims
$R_bwwc7dPFEcp1azH$	Yes particularly for features such as pragmatic language skills
$R_0ofhSCmeppIQ8kt$	They are inadequate in some way ... but for more reasons than just that they may ignore the highly contextual nature of language processing (they can't sample all language functioning; they're just a snapshot, etc)
$R_6Dvhy7Alhw5wqIR$	they are only one tool among several

$R_cCuacCYZiqQHKgl$	Standardized tests are neither adequate or inadequate. It depends on what they are being used for. They provide the opportunity to have some reliable data on language performance. Indeed, they often are strongly associated with more naturalistic information. It is important to consider that standardized tests are intended to measure individual differences. If the diagnostic question concerns the child's relative standing, these seem to be adequate. If the diagnostic question is one of the child's ability to perform in particular ways in particular settings, they are likely to be inadequate.
$R_6mrinfsu6CeSmBn$	For children with pragmatic language difficulties, there are no tests wihich adequately identiy this.
$R_2o7JoTNgC3lqSIR$	Relying on tests alone is not enough, though in our experience test scores usually give a pretty good picture and provide essential data so should not be abandoned. Therapists however should, and the good ones do, back this up with close observation of children in real-life situations.
$R_e5KJQmN6txthTRX$	They are very useful at pinpointing the exact extent and nature of an individual child's difficulties, but do need to be put into context by the use of robust dynamic assesment
$R_71b9fvukXBUQ5dr$	They do what they say on the tin: measure within a standard context. This is not inadequate: may be inadequate for some purposes. If context matters, get further information on that.
$R_7WXquZJy8WlgXAx$	are inadequate by themselves, a range of ways of gathering information including some in context is ideal
$R_1QTm7VrpDX1OAi9$	They are inadequate on their own, but should be part of the battery
$R_1z8h1XMT676UOwd$	Standardised tests play an important role in diagnosis but should be supplemented with assessments of language use in real-life with peers, teachers and family members when planning intervention.
$R_3DfMsLnqK54HqcZ$	Agree we need other considerations - not just tests - for diagnosis but a test is a very important tool. SLTs often under estimate children's difficulties as their experiences become skewed as to what is 'typical' and educational assessments are often not linked to a child's age but rather to their educational 'stage' and so are not a substitute. Dynamic assessments are not currently sufficiently well developed either however RTI public health models may be.
$R_834xbT3yZzu1O7z$	Standardised tests ALONE are inadequate - should be seen in context of other skills
$R_23qAFVuJCo6YHOd$	Standardised test are important but not sufficient.
$R_eG1jl51DiHRqXKB$	Hard to give a good answer even though I think it is important.
$R_8bIXFrv4VBlvVyZ$	They do ignore the highly contextual nature of language processing but this is useful since it allows us to isolate specific strengths/weaknesses and gaps in a cild's language-they can also remove the possibility of coping strategies as might be used by the child in context, thus providing a more realistic baseline; however in order to determine a full picture and identify parameters, goals and contexts for intervention, we need to also know how the child processes and uses language in context.

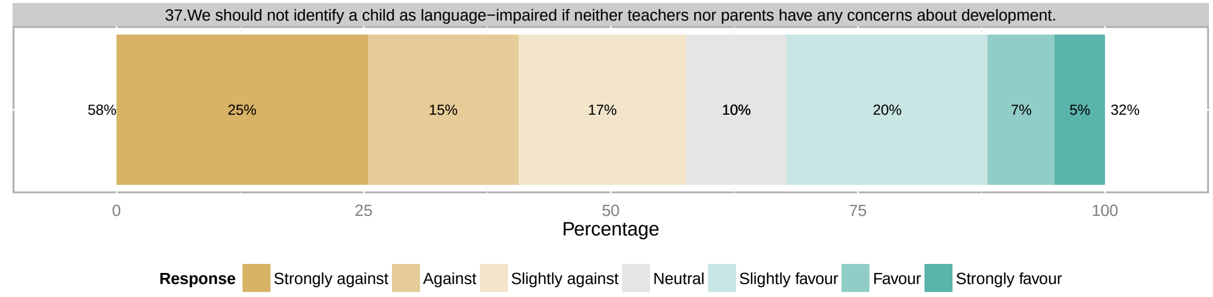

Figure 75: Percentage of panel members in each response category to statement 37. The percentages shown at each end of the scale are the cumulative percentages for the top and bottom three categories respectively.

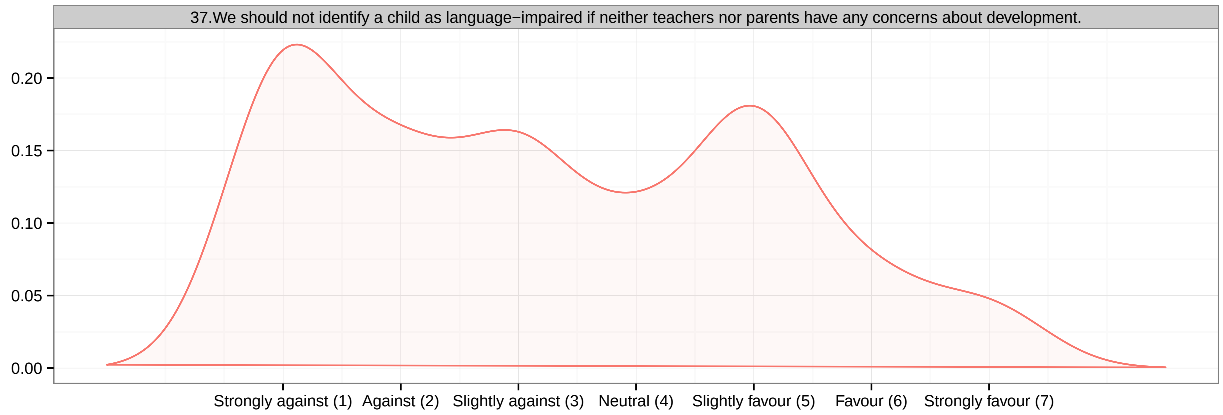

Figure 76: Distribution of responses to statement 37.

Table 37: Comments for each statement.

ResponseID	Q37B
$R_2f9ctxaHBJuJdLD$	It would be unusual not to have both concerned- but often unless parents drive a process the school is passive.
$R_6RlkuyWJYcIIsmN$	It is the role of experts to identify a problem that may be subtle and/or mistaken for another disorder (and be mismanaged as a consequence). We diagnose hearing, vision problems and other conditions in the absence of parent/teacher concern.
$R_5cKMfR48zQytYc5$	well thats an interesting one - but have to disagree as the parent may also have low language and literacy
$R_6JOosydU46ZndMF$	this is a rather ambiguous statement because the key issue is associated impairment and how to measure it - context dependent
$R_bOrkJKVQ6T8FeGp$	This gets back to the 'functional' issues discussed before.
$R_5cd8BDkYcGfGLKl$	lack of parent concern can reflect many things that may not be beneficial to the child. Educating parents/teachers seems a better way forward.
$R_3sXNbQYRlZaMb3L$	It's our responsibility to point these difficulties out. Receptive language difficulties in particular can go completely under the radar and present only as behavioural difficulties. The proportion of children with receptive langage problems in EBD schools (and, I believe, amongst the prison population) is very high. Receptive language is something I routinely look at for children with behaviour difficulties, even though it's not the concern highlighted by the family or school.
$R_e9cPjWuFpcer4B7$	This a complex question and again needs to be split to be useful. I would be very careful about making such a judgement if all those who knew the child best were expressing no concerns after discussion.

$R_9uJ5LinD5e8X5Yh$	Teachers and parents can be confused/misled by the savvy child who uses all contextual clues but actually has very poor comp. If the child is making age approp progress in the classroom after 8 years old across all areas of learning only then would I hesitate to identify as language impaired
$R_1TXxdyLg1UFCx4V$	This is a difficult statement. The individual does need to have an identifiable impairment in a real context and if this is not the case then they should not be identified as having an impairment. However there are occasions when a child's difficulties are not apparent to teachers or parents but the individual is struggling
$R_bwwc7dPFEcp1azH$	Yes- research has demonstrated that diagnostic accuracy improves if these opinions are considered, although ideally both (teachers AND parents) and not one or the other would be involved
$R_cCuacCYZiqQHKgl$	There are many children who face considerable difficulties in classroom and reading comprehension and yet neither the parents or teachers realize that the problem lies with poor language.
$R_4HGIGYFIvMxLWcJ$	it is all about expectations - especially in children from disadvantaged backgrounds.
$R_2o7JoTNgC3lqSIR$	In reality, this is unlikely to happen as, if neither parents nor teachers are concerned, it is highly unlikely that a therapist would become involved. However, should the situation arise, the first consideration should be the wellbeing of the child.
$R_e5KJQmN6txthTRX$	It's hard to imagine such a scenario happening. If it does, the interests of the child must come first
$R_71b9fvukXBUQ5dr$	Comprehension problems are routinely missed (as hearing impairment used to be).
$R_7WXquZJy8WlgXAx$	Language difficulties are often 'hidden' but may be there. if SOMEONE has raised a concern then language impairment should be idenitfied. it may not have impacts at a young age, but emerge and impact later. knowing there is an impairment is useful; this doesn't necesaarily mean there should be intervention (see earlier answer)
$R_1QTm7VrpDX1OAi9$	In general not, but there may be cases (particularly for poor comprehension) where difficulties may be masked (sometimes by behaviour), so I wouldn't want to be categorical about this
$R_1z8h1XMT676UOwd$	Teachers and parents may not have the skills to identify subtle language problems, and may not be aware of the difference between speech and language or the complexity of language.
$R_23qAFVuJCo6YHOd$	I think this should read 'carers, professionals or the young person themselves'. /
$R_8bIXFrv4VBlvVyZ$	we have to allow for a role for and a wider societal responsiblity to look after the needs of a child who difficulties may not be identified by key people in the child's environment.

2.6 Breadth of inclusion

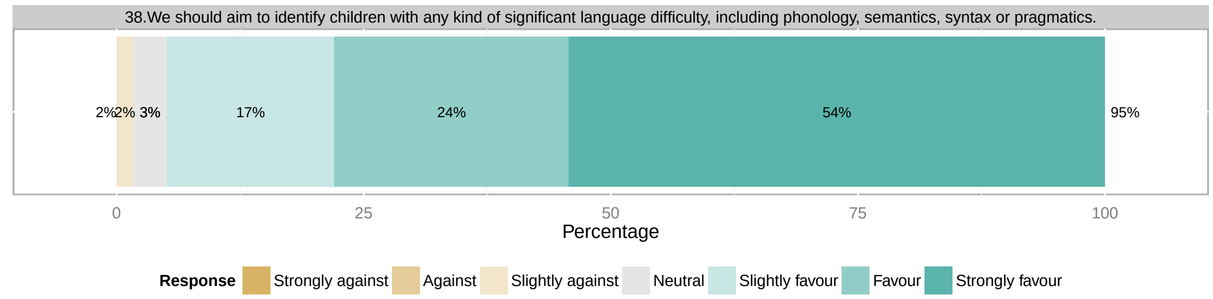

Figure 77: Percentage of panel members in each response category to statement 38. The percentages shown at each end of the scale are the cumulative percentages for the top and bottom three categories respectively.

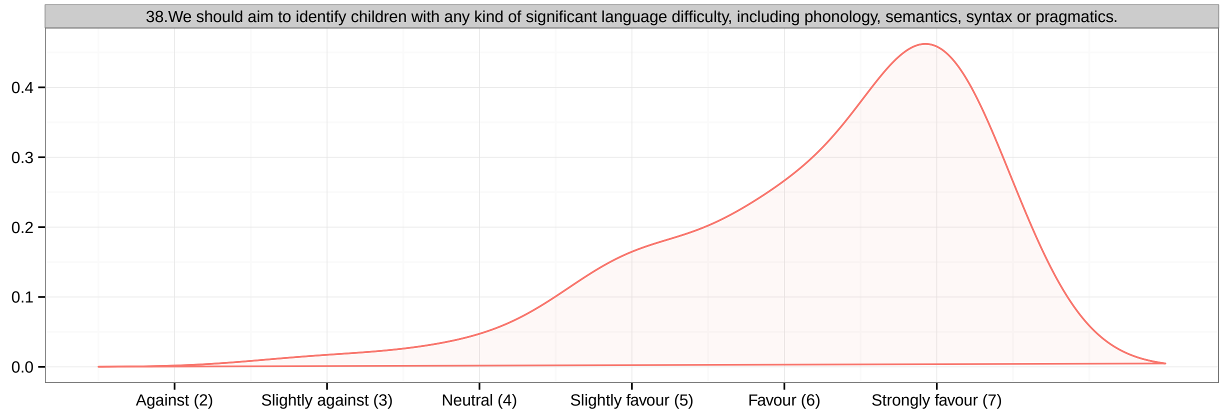

Figure 78: Distribution of responses to statement 38.

Table 38: Comments for each statement.

ResponseID	Q38B
$R_5cd8BDkYcGfGLKl$	most of these things will be highly correlated. It is worth identifying them, but also important to document how they impact on a child's academic and social well-being.
$R_6LIAgEx6sspizpX$	Only if parents or schools are concerned
$R_e9cPjWuFpcer4B7$	Identify for what? For a research study? This obviously depends on the inclusion criteria. Identification as having an SLCN? Yes absolutely. The extent to which these are language problems depends on the definition of and scope of the term language, but I would say that all these characteristics are a part of language.
$R_9uJ5LinD5e8X5Yh$	No brainer. Info to the teacher can make a massive difference to their planning and expectations
$R_0ofhSCmeppIQ8kt$	But I don't know if they all get called the same thing.
$R_cCuacCYZiqQHKgl$	For clinical identification, we need to address those areas of performance that limit the child's ability to perform. Research is needed to show how variations in abilities in these areas impacts on the child's life.
$R_2o7JoTNgC3lqSIR$	Of course, yes, but if you mean do we categorise all of these as LI or SLI, not necessarily
$R_71b9fvukXBUQ5dr$	Linguists may argue that pragmatics is not a language difficulty. It matters a lot in childhood, but non-verbal aspects should eb included.
$R_7WXquZJy8WlgXAx$	I feel this is important - not to limit to aspects of syntax, although we may consider just recpetive aspects of phonology. Unsure about pragmatics - pragmatic language impairment?
$R_1QTm7VrpDX1OAi9$	and children who have difficulties in more than one area should be higher priority for receiving intervention

$R_{8}34xbT3yZzu1O7z$	Not phonology if it is LANGUAGE impairment

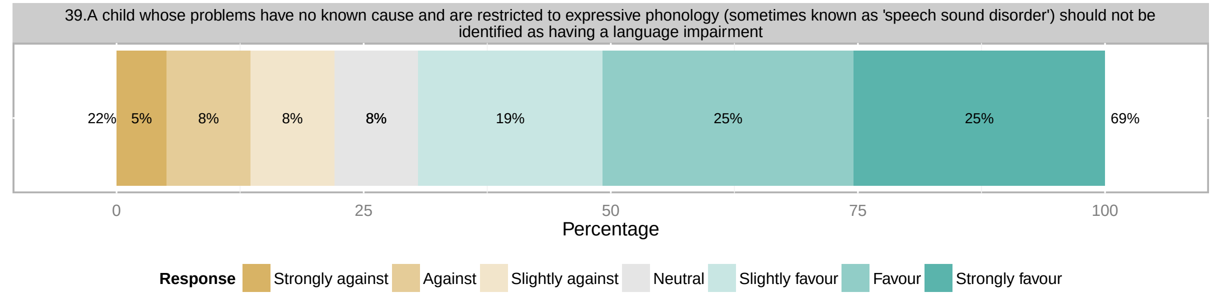

Figure 79: Percentage of panel members in each response category to statement 39. The percentages shown at each end of the scale are the cumulative percentages for the top and bottom three categories respectively.

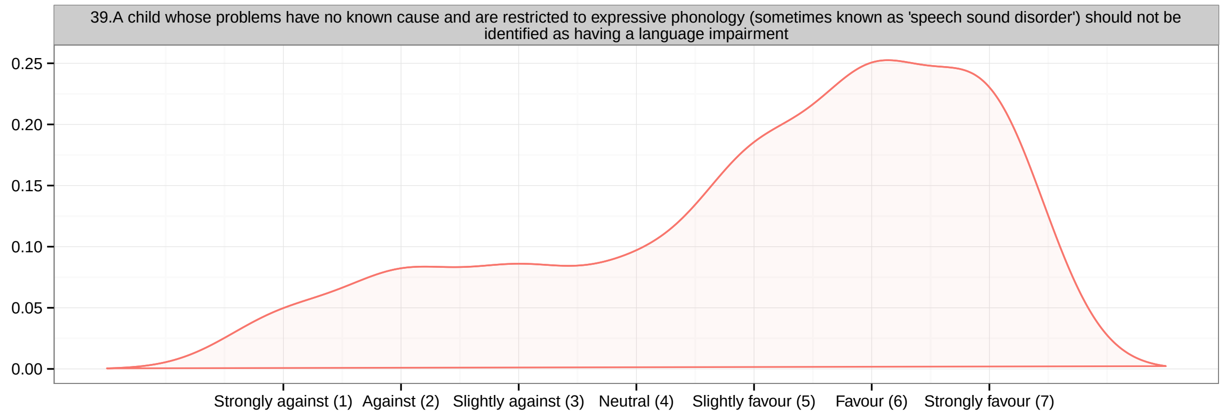

Figure 80: Distribution of responses to statement 39.

Table 39: Comments for each statement.

ResponseID	Q39B
$R_2f9ctxaHBJuJdLD$	Technically
$R_6RlkuyWJYcIIsmN$	Phonology is part of the language system. Though children with SSDs may not have difficulties in other language domains, it is still an aspect of language - albeit a very specific one. We need a diagnostic classification system that can accommodate this. / Form-Content-Use :)
$R_6JOosydU46ZndMF$	my understadning is that SSD may later be associated with or manifest as problems in reading decoding &/or comprehension as well as written expression
$R_bOrkJKVQ6T8FeGp$	This is not sometimes known as SSD - it IS known as SSD! But agree there is complexity as phonology is language.... let us know go there!
$R_6LIAgEx6sspizpX$	As long as that doesn't mean that people stop considering the child's lanaguage skills e.g some chdilren who have SSD also have difficulties with expressive language or word-finding
$R_e9cPjWuFpcer4B7$	The separation between language speech and pragmatics is supported by Bruce Tomblin's work. I would call them all language impairments and include three "types" of language impairment with different features. Speech disorders would be restricted to those where phonology was not involved.
$R_9uJ5LinD5e8X5Yh$	If language impairment just means lang formulation then maybe this is the wrong label for them? But we need to assess that it is only expressive phonology which is affected. My mantra 'if you can't say it you can't write it' is frequently true - then you have a language impairment? Very important for the classroom
$R_1TXxdyLg1UFCx4V$	It depends on the degree to which the difficulty is impairing or has an impact on the individual. Phonological disorders are language disorders
$R_bwwc7dPFEcp1azH$	It would be more 'speech and language' impairment than language alone as it involves elements from both

$R_6Dvhy7Alhw5wqIR$	I prefer the term 'speech imp' for this group
$R_cCuacCYZiqQHKgl$	There is a substantial amount of data that show that speech sound disorders seem to occupy a fairly separate dimension of development than language. They do not seem to reside on the same dimension of severity.
$R_4HGIGYFIvMxLWcJ$	this is a language-based disorder in my opinion
$R_6mrinfsu6CeSmBn$	They require a different care pathway.
$R_2o7JoTNgC3lqSIR$	Yes, if it is only a speech disorder, but it should be borne in mind that language difficulties sometimes become apparent later on
$R_e5KJQmN6txthTRX$	There is a clear distinction between speech and language
$R_71b9fvukXBUQ5dr$	Yup. Useful distinction to keep.
$R_7WXquZJy8WlgXAx$	I feel this should be spearate - the focus should be on imairments with language
$R_23qAFVuJCo6YHOd$	In practice, a child who has severe persisting phonological difficulties will show difficulties with language (e.g. morphology, vocabulary storage and retrieval), so this is very difficult to split.
$R_eG1jl51DiHRqXKB$	Yes,because some problems are motoric– but should always be discussed for other or additional explanations
$R_8bIXFrv4VBlvVyZ$	Having seen the effects of including speech sound disorder within a broader category of SLI in one context, I would now question the wisdom of including SSD-it has resulted in children being given a label which is seen to characterise a long term need, when those children's difficulties resolved by early primary school; additionally the inclusion was used to enable children to access the more intensive/speciailised sources of help as would be required by children with wider LI, when in fact the children with SSD did not need this.

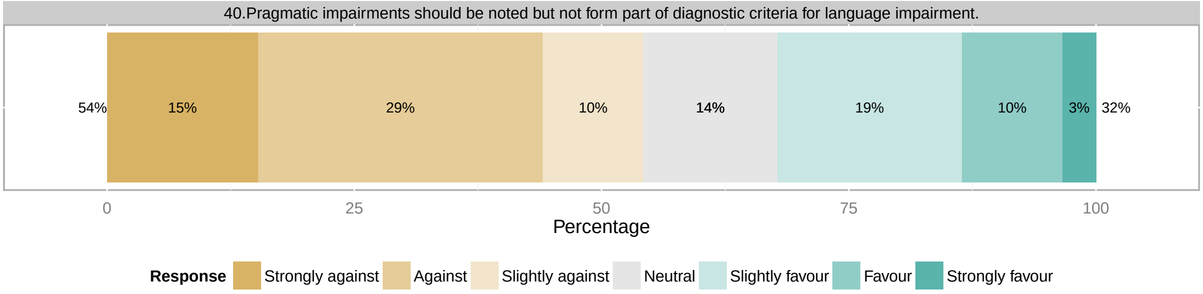

Figure 81: Percentage of panel members in each response category to statement 40. The percentages shown at each end of the scale are the cumulative percentages for the top and bottom three categories respectively.

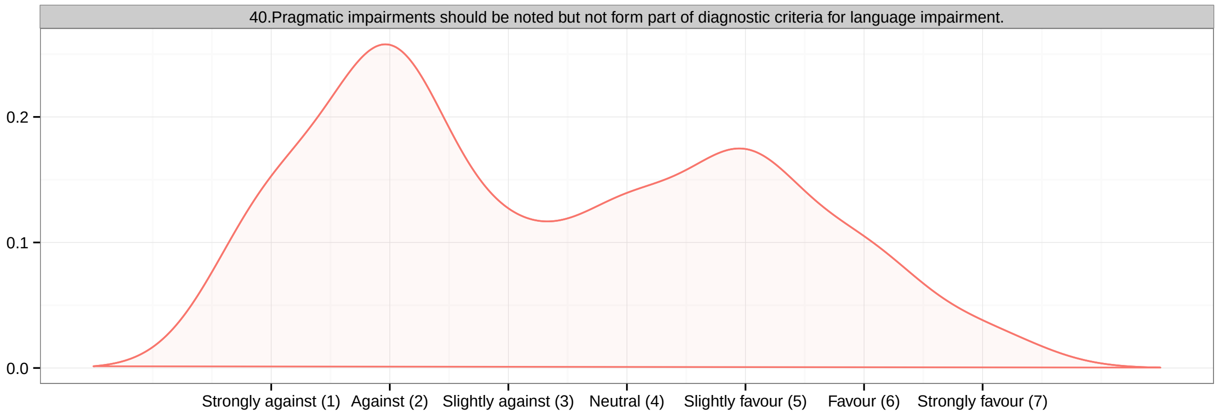

Figure 82: Distribution of responses to statement 40.

Table 40: Comments for each statement.

ResponseID	Q40B
$R_6RlkuyWJYcIIsmN$	A no-brainer! Pragmatic impairments often result in the highest degrees of psychosocial impairment, so we can't ignore them. / Form-Content-Use :)
$R_5cd8BDkYcGfGLKl$	what we mean by pragmatics probably requires clarification, but many aspects of pragmatics are linguistically based, important for academic and social success (i.e. inferencing) and may respond to SLT.
$R_3sXNbQYRlZaMb3L$	I don't think it's important. We need to describe what the need is so that we can meet the need.
$R_3VHaciSzwJGKIU5$	pragmatic language difficulties can impact on reading comprehension and narrative text and as such it would make sense to include them in language impairment
$R_e9cPjWuFpcer4B7$	Of course we have to be careful with our terms here. So a child who was socially awkward and had trouble interacting effectively with peers might look like they have a pragmatic impairment but without some element of language difficulty I would not include them under LI.
$R_9uJ5LinD5e8X5Yh$	If just pragmatic impairments then this is a language impairment but maybe stemming from ASD (book 5)? So not the full whammy of language impairment but a significant impairment all the same. I know the research says that non ASD have pragmatic impairments but I find it is always as a result of the SLI. Pragmatic impairment alone us usually stemming from a deeper cause than just lang in the ASD population (I hear the screams from the SLT researchers!! If only you would involve educationalisits as part of your research!)
$R_1TXxdyLg1UFCx4V$	This statement relates to the concept of a specific LI but if one considers the broader spectrum of language impairment including those co-occurring with deafness, autism and learning disability then pragmatic impairments are integral to the profile of the individual and their needs

$R_cCuacCYZiqQHKgl$	This then means that we are defining language as semantics, grammar and discourse. We need better data as to whether this narrow notion of language represents a cohesive different dimension of development than pragmatics. The data seem to suggest that this may be the case, but more evidence would be good.
$R_2o7JoTNgC3lqSIR$	I would tend to agree, provided that the use of an alternative term such as pragmatic language impairment is clearly understood and used. One problem we encounter a lot is that children with social communication difficulties might get reasonable scores on a CELF assessment and are then dismissed as not having a problem, or a serious problem at any rate.
$R_e5KJQmN6txthTRX$	It's not the same as a structural language impairment, but it should certainly be diagnosed and treated
$R_71b9fvukXBUQ5dr$	Yup. Useful distinction to keep. See above about non-verbal factors.
$R_7WXquZJy8WlgXAx$	Language impairment in the area of pragmatics? defintiely noted. unsure
$R_3DfMsLnqK54HqcZ$	I need this topic to be more clearly articulated is this asking whether PLI should be part of LI?
$R_23qAFVuJCo6YHOd$	Pragmatics should be part of SLI OR it should be clear that SLI and PLI features often co-occur.
$R_eG1jl51DiHRqXKB$	This is a difficult question but should be discussed.
$R_8bIXFrv4VBlvVyZ$	I would see PLI as part of LI and that PLI includes children with language processing difficulties-as such they will need and benefit from some similar supports/services but pragmatic impairments do not need to be specified in the diagnosis; I would assume each component (pragmatics, semantics, morpho-syntax, phonology) to potentially be part of the presentation of LI

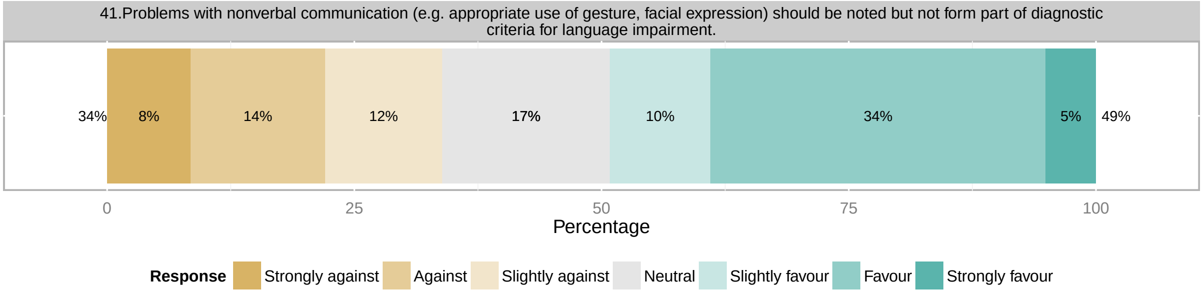

Figure 83: Percentage of panel members in each response category to statement 41. The percentages shown at each end of the scale are the cumulative percentages for the top and bottom three categories respectively.

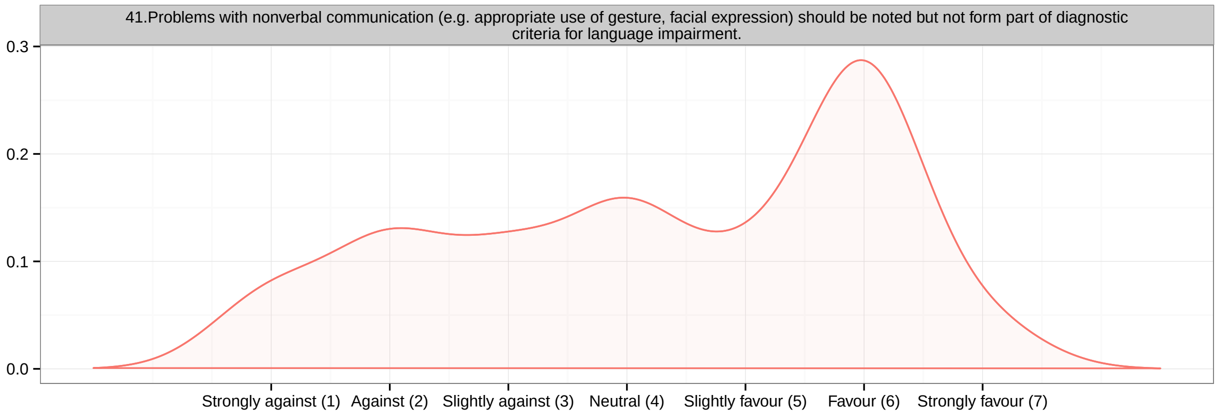

Figure 84: Distribution of responses to statement 41.

Table 41: Comments for each statement.

ResponseID	Q41B
$R_2f9ctxaHBJuJdLD$	vital to distinguish from receptive language disorders for example
$R_6RlkuyWJYcIIsmN$	See above re pragmatic language skills - nonverbal skills fall under this umbrella.
$R_6JOosydU46ZndMF$	I am not sufficently knowledgeable with the literature on this, but since non-verbal communication contributes to effective communication, my belief is that these features should be specified
$R_e9cPjWuFpcer4B7$	Yes, I agree these are part of the description of all children but are really just part of normal variation and are likely to be sensitive to the age of the child, expectations of those around them etc. The wording has drifted back from identification to "diagnostic criteria". Again I would suggest that the term diagnosis is strictly medical. It does not make much sense to educationalists who often see psychologists and speech and therapists as relying too heavily on a deficit model when assessing children.
$R_9uJ5LinD5e8X5Yh$	See above.
$R_1TXxdyLg1UFCx4V$	I agree this is not integral to a disorder of language impairment but it should be included as part of a broader evaluation of the individual's profile of strengths and difficulties and will be relevant to the diagnosis of co-existing conditions such as autism and plans for intervention that might include compensatory strategies such as signing
$R_cCuacCYZiqQHKgl$	Again if such problems are actually problems for children then we could entertain this.
$R_2o7JoTNgC3lqSIR$	Not in themselves no.
$R_71b9fvukXBUQ5dr$	As above.
$R_3rrKtkb2VvC3uG9$	this is age dependent
$R_3DfMsLnqK54HqcZ$	I need this topic to be more clearly articulated - so is this saying whether Social Communication difficulties should be part of LI?

$R_2 3qAFVuJCo6YHOd$	Query where turn taking skills fit - whether in non verbal or in pragmatic language.
$R_e G1jl51DiHRqXKB$	This seems to be part of the issue of pragmatic language (social communication skills) described above. If the focus is on communication rather than language per se, this is important. Difference is between a language and a communication disorder which may or may not overlap.
$R_8 bIXFrv4VBlvVyZ$	I think including these could result in children with wider needs or difficulties other than LI being described as LI

2.7 Co-occurring problems

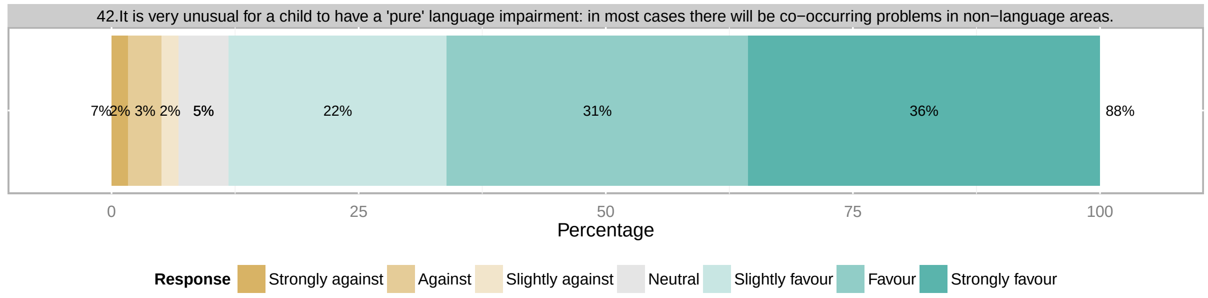

Figure 85: Percentage of panel members in each response category to statement 42. The percentages shown at each end of the scale are the cumulative percentages for the top and bottom three categories respectively.

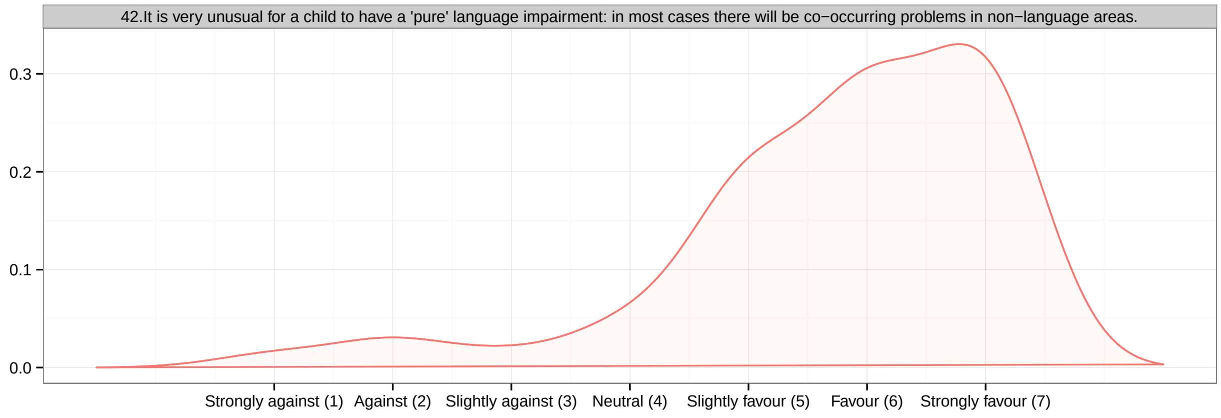

Figure 86: Distribution of responses to statement 42.

Table 42: Comments for each statement.

ResponseID	Q42B
$R_e9cPjWuFpcer4B7$	This is very rare in my experience and probably indicates positive outcomes. Comorbidity is key to predicting persistence.
$R_9uJ5LinD5e8X5Yh$	If this means motor planning areas then I strongly agree. Do you mean also literacy/number skills to be included in the pure LI - if not they co occur.
$R_2o7JoTNgC3lqSIR$	It depends what we mean by 'most' cases. It certainly is the case that some children diagnosed with SLI when young, go on to get other diagnoses later. The extent to which these other difficulties were present or could have been identified earlier varies.
$R_71b9fvukXBUQ5dr$	As a result, or concomitant factors?
$R_7WXquZJy8WlgXAx$	I am not sure about the 'very unusual' and 'most cases'. there may well be co-occuring problems
$R_1QTm7VrpDX1OAi9$	There are a few children with a 'pure' language impairment, but far fewer than the research literature would lead us to believe.
$R_1z8h1XMT676UOwd$	It depends on whether this statement applies to the general population or to people at risk for language problems that includes conditions such as autism spectrum disorder or Down syndrome. The literature suggests a significant minority of children have 'pure' language impairment, but their auditory processing or working memory, etc, was not comprehensively assessed to see whether they had 'non language' problems.
$R_eG1jl51DiHRqXKB$	I don't know about the 'very' part. Here is where the importance of looking at nonlanguage behaviours is important.

$R_8bIXFrv4VBlvVyZ$	yes in research and practice, I've seen very few children with "pure" language impairment

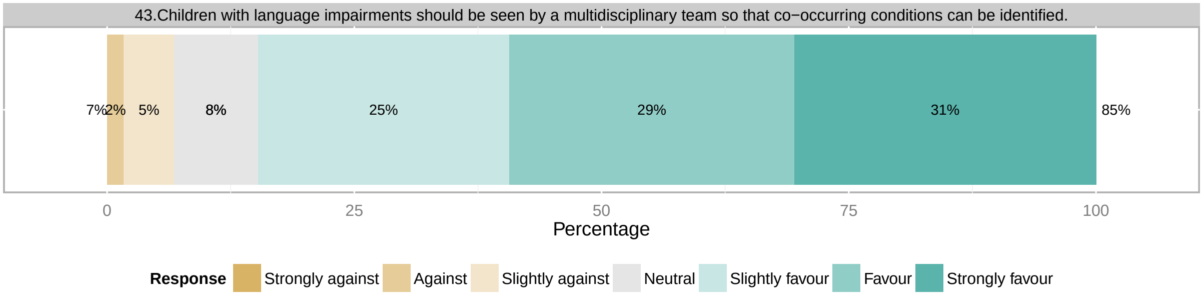

Figure 87: Percentage of panel members in each response category to statement 43. The percentages shown at each end of the scale are the cumulative percentages for the top and bottom three categories respectively.

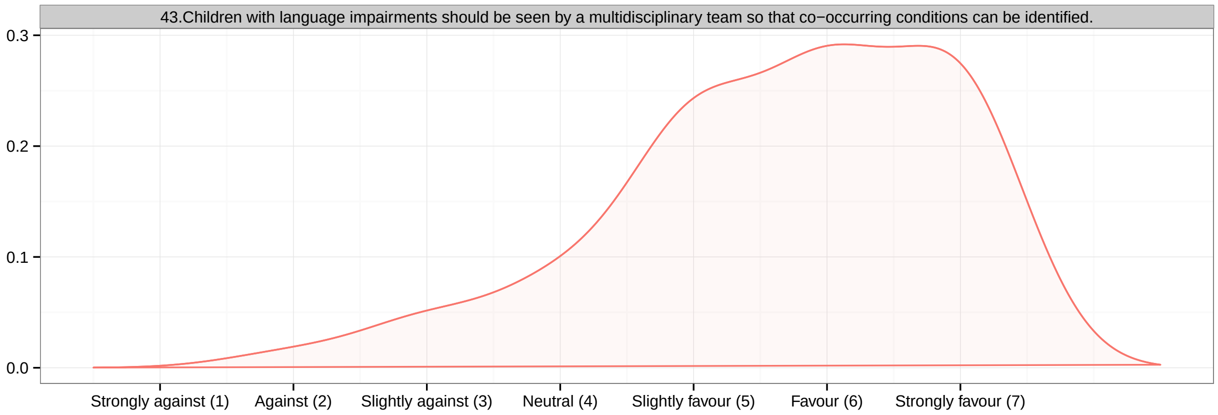

Figure 88: Distribution of responses to statement 43.

Table 43: Comments for each statement.

ResponseID	Q43
$R_6RlkuyWJYcIIsmN$	I agree with this statement in a clinical sense (ie good practice), but SLT/SLP can still diagnose a LI, irrespective of knowledge re co-occurring conditions (knowledge of which is of course critical to management).
$R_6JOosydU46ZndMF$	yes, LI co-occurs with other neurodevelopmental disorders (ADHD, ASD, DCD etc) as well as psychiatric/mental health disorders (anxiety, oppositional defiant disorder,depression etc)
$R_6JZKVRyNZK6U0zX$	In an ideal world where resources are unlimited...!
$R_6LIAgEx6sspizpX$	It depends what the presenting issues are - this should be an option not an obligation
$R_3VHaciSzwJGKIU5$	There is a practical issue here about resources. There seems little point to ask for something that cannot happen for the majority of children
$R_e9cPjWuFpcer4B7$	This an interesting discussion point but clearly quite impractical in most cases given the availability of this sort of resource. I think this could be linked to RTI and that these teams could be brought to bear for children who do not respond. brining in children for a series of one off assessments is not I think the solution to this problem.
$R_9uJ5LinD5e8X5Yh$	Unravelling a child's profile is critical - SLTs EPs and specialist teachers take a holistic view and then pathways for progress are strong. Lose any one of these perspectives and the weaknesses of diagnosis can become apparent later on -when behaviour and self esteem deficits are then co occurring.
$R_bwwc7dPFEcp1azH$	Yes including Psychology, OT, Teachers etc although agreement about the definition of the diagnosis should be agreed across disciplines which is difficult

$R_e s7hPPlfD7bdd65$	Sounds logical but in reality, how do you know they need to be "seen" by a team until you identify the problem/s? Ultimately this starts with one person identifying a problem, so really the question is how to follow up in terms of treatment, right?
$R_0 ofhSCmeppIQ8kt$	I just don't think we can build this into our system and sustain it.
$R_4 HGIGYFIvMxLWcJ$	it really depends on the clinician, experience, presenting problems.
$R_2 o7JoTNgC3lqSIR$	Absolutely. A full multidisciplinary team with medical involvement would help to give the condition status and validity - provided that is they take it seriously. At the moment, too many paediatricians seem to assess for autism and if children do not meet the criteria simply dismiss their difficulties as 'an educational issue' and not a medical condition. Parents and children/young people do themselves form part of the multidisciplinary team and their views and infomation should be taken seriously.
$R_e 5KJQmN6txthTRX$	Parents, children and young people should be part of the team, inform the discussions and decisions. They must be taken seriously. Professionals must know what questions to ask parents.
$R_7 WXquZJy8WlgXAx$	other professionals involved if there is an indication of other problems. this may not always be necessary
$R_8 bIXFrv4VBlvVyZ$	yes they should be provided access to an MDT but not to the extent that an MDT is needed to identify a significant LI in the first instance

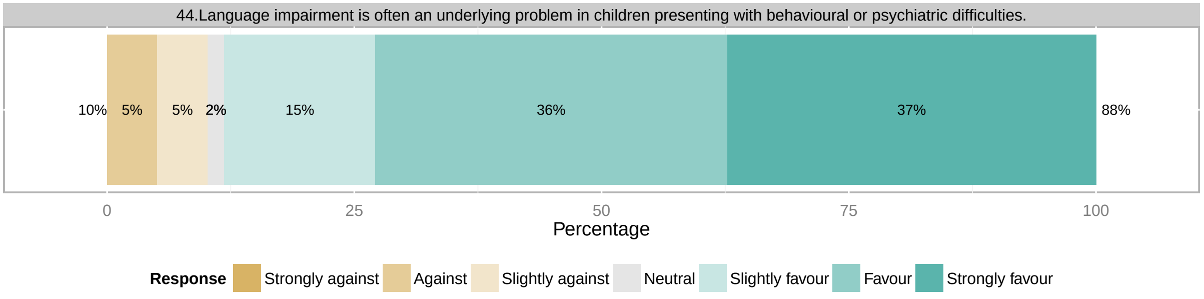

Figure 89: Percentage of panel members in each response category to statement 44. The percentages shown at each end of the scale are the cumulative percentages for the top and bottom three categories respectively.

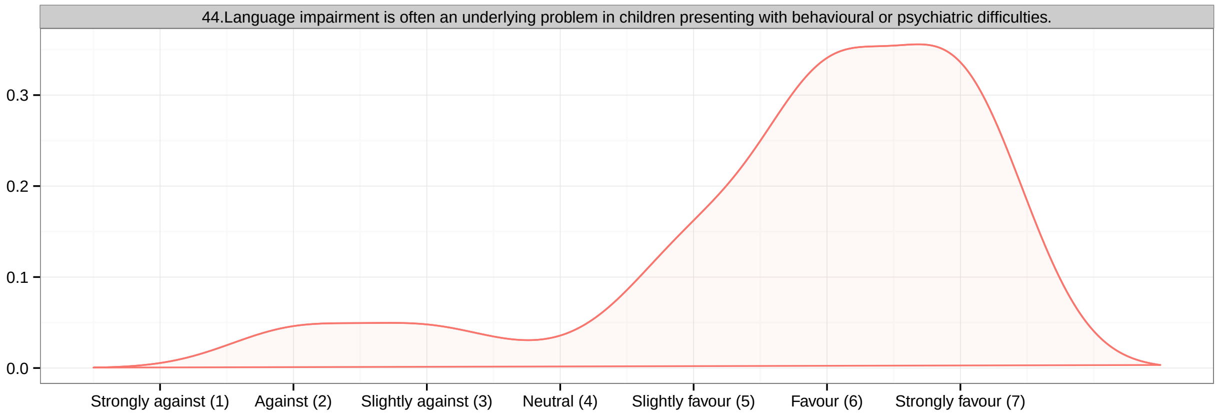

Figure 90: Distribution of responses to statement 44.

Table 44: Comments for each statement.

ResponseID	Q44B
$R_b a8iHG84IJ8cW7X$	I feel uncomfortable about the inclusion of 'psychiatric difficulties'; I do recognise that children's manifest behaviour may be an expression of difficulties with expression or comprehension of language. Which of us has not at times felt some frustration at not being able to find the right words or not understand what has been said (and feeling somewhat 'stupid' as a result)?
$R_6 RlkuyWJYcIIsmN$	This is commonly overlooked/misunderstood in school settings.
$R_5 cKMfR48zQytYc5$	not sure about often but can be present
$R_6 JOosydU46ZndMF$	and unfortunately often overlook in these children with behavioral/psychiatric disorders
$R_9 U2zxMLVAPcvQUd$	I agree that LI is common in EBDs but I disagree with the term underlying if the intent is to imply that the LI causes the EBD symptoms or EBD misdiagnosis. I see them as commonly co-occurring.
$R_e 9cPjWuFpcer4B7$	This ia very loaded question because it implies directionality which probably is not there. These aspects of a child do commonly overlap as thirty years of evidence have shown us but they much more likely to be a result of common underlying neurodevelopmental conditions as language difficulties causing behavioural difficulties, especially in the early years. I think it may become more complex as the child gets old especially if pragmatics is involved, but this is not really an issue for a questionnaire.
$R_9 uJ5LinD5e8X5Yh$	See above............a bigger issue than most people think, despite research showing re the behavioural /youth offending links.
$R_6 mrinfsu6CeSmBn$	I think lack of identificaiton of these chidlren's language difficulties is a separate issue.

$R_2o7JoTNgC3lqSIR$	This may be the case, but the reverse does not always apply. By no means all children with language impairments go on to develop behavioural or psychiatric difficulties, so it is important not to conflate the two. A difficulty with language is enough of a disability in itself, it should not have to be tied to something else to be taken seriously.
$R_e5KJQmN6txthTRX$	Yes, but the reverse isn't always true. Children with language impairments do not by any means always develop behavioural or psychiatric difficulties
$R_71b9fvukXBUQ5dr$	Don't know about 'often' - some evidence for this, but LI children do not move into behavioural difficulties categories as often as seems to be thought.
$R_7WXquZJy8WlgXAx$	I agree, but am not how useful this is to include in this discussion

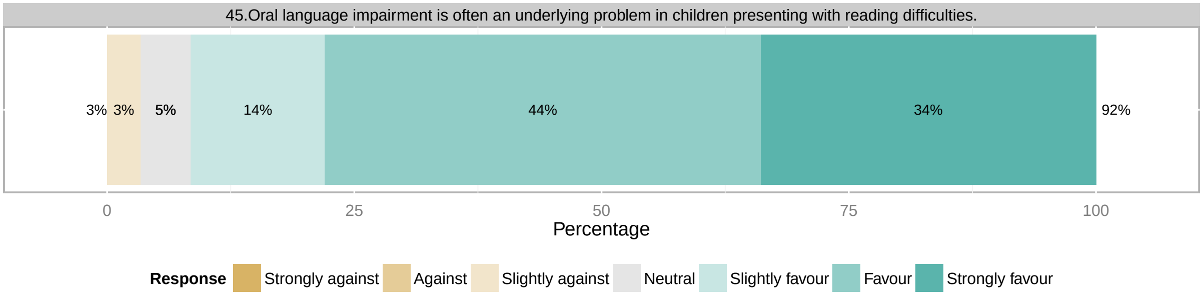

Figure 91: Percentage of panel members in each response category to statement 45. The percentages shown at each end of the scale are the cumulative percentages for the top and bottom three categories respectively.

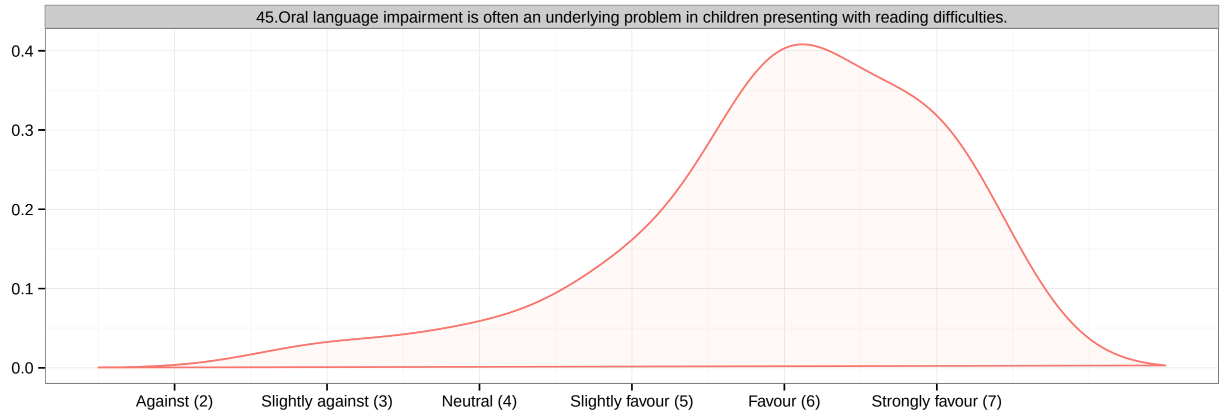

Figure 92: Distribution of responses to statement 45.

Table 45: Comments for each statement.

ResponseID	Q45B
$R_6RlkuyWJYcIIsmN$	Again, this connection is often understated / not well understood in education settings. needs emphasis for policy makers.
$R_6JOosydU46ZndMF$	in English language, yes often with decoding words because of challenges with English orthograpy; also associatioed with poor reading comprehension across languages, regardless of orthography
$R_e9cPjWuFpcer4B7$	Yes this is true for children with reading comprehension problems. It is not necessarily true for children with problems decoding.
$R_9uJ5LinD5e8X5Yh$	If you had said oral meaning a child's spoken and comprehension skills then I would have scored strongly agree. Comprehension is a key barrier to reading difficulties, spoken lang skills problems undermine all literacy skills to a pervasive and long lasting degree - especially if the focus on phonics remains. Gggrrrrrrr the phonics assts!!
$R_3pDedyU4fM1kOXj$	Not every child with oral language impairment will have difficulties with reading. If children do present with difficulties these difficulties can present differently depending on the profile of the child. Some children with language impairment can be very successful at de-coding phonemes however they struggle to understand the words that they have read. Some children with language impairment can have difficulty de-coding the phonemes and understanding the vocabulary.
$R_2o7JoTNgC3lqSIR$	There is certainly a connection, but it is by no means the case that all or even most children with reading difficulties have or had language problems, or that all children with language problems go on to have significant reading difficulties
$R_e5KJQmN6txthTRX$	Yes, but again the two are not the same and can co-exist or exist independently
$R_71b9fvukXBUQ5dr$	But - reciprocal issues.

$R_7WXquZJy8WlgXAx$	as above
$R_ebTqVBlGUNh60eN$	Not sure if this is the case. Receptive language difficulties more so.
$R_8bIXFrv4VBlvVyZ$	yes many children with reading difficulties have an underlying oral language impairment or a history of same

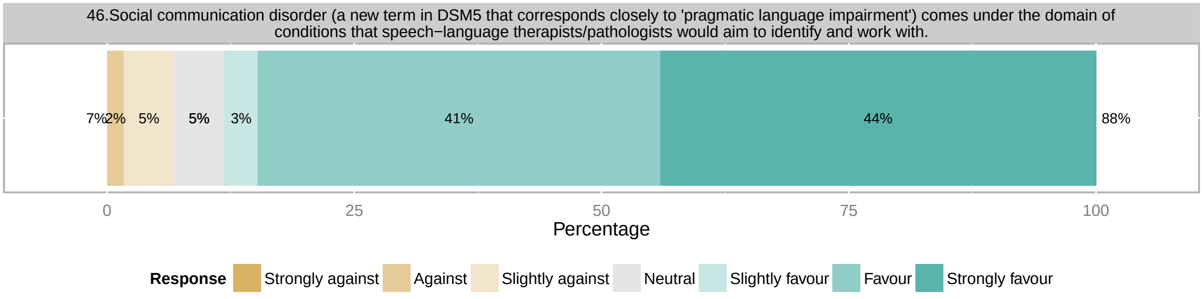

Figure 93: Percentage of panel members in each response category to statement 46. The percentages shown at each end of the scale are the cumulative percentages for the top and bottom three categories respectively.

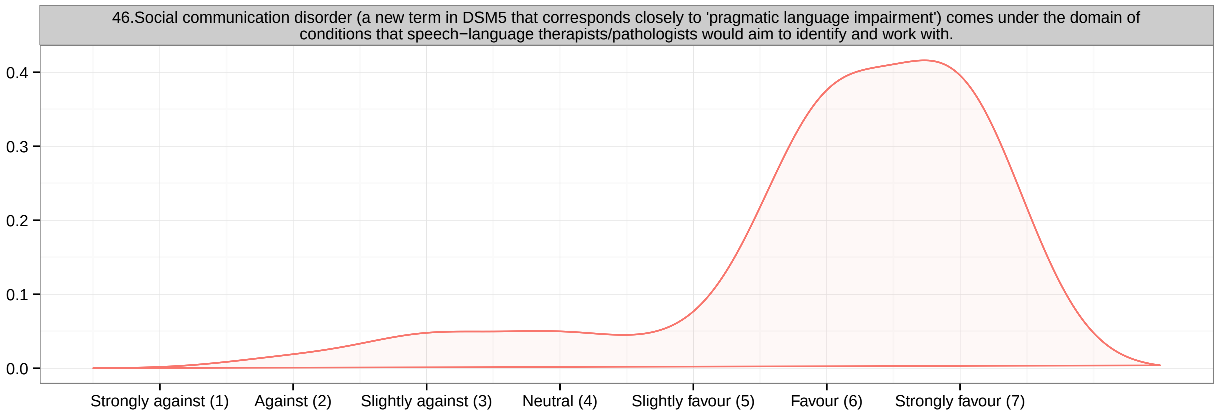

Figure 94: Distribution of responses to statement 46.

Table 46: Comments for each statement.

ResponseID	Q46B
$R_2f9ctxaHBJuJdLD$	social communication disorder also covers PDD NOS or PLI but PLI can occur within ASD as well so we are back to the subgroup of PLI who are not ASD.
$R_6JOosydU46ZndMF$	this new DSM-5 condition needs investigation since it was not possible to assess validity/reliability of this category
$R_e9cPjWuFpcer4B7$	Yes, assuming that most of these children have underlying language problems and that they are not just a feature of an anxiety disorder. I would expect psychologists and speech and language pathologists/ therapists to collaborate closely in the management of these cases.
$R_9uJ5LinD5e8X5Yh$	This is may be my final book - it will take til I die! I have never met a pure pragmatic lang disorder. I am an educationalist and there is pretty well always an ASD learning style which sits with the pragmatic lang disorder (can be subtle I will agree, but there!). Of course every SLI young person will have pragmatic ie social difficulties if their lang skills are not fit for purpose. But there is another type of pragmatic lang disorder which goes beyond lang (eg confusion over non verbal messages, inappropriacies, sause and effect difficulties). Can we be clearer about the label pragmatic lang disorder V social communication disorder (ASD in the educational world), SLCN and SLI. This study should help massively if you take account of all professional aspects (ie education specialists). / An SLT can help all groups but in different ways.
$R_1TXxdyLg1UFCx4V$	I don't agree with the premise of this statement but I do agree that pragmatic impairments can part of the SLTs focus for intervention. The need for SLT involvement should depend on degree of communication impairment and impact on the individual not the domain of impairment
$R_2o7JoTNgC3lqSIR$	Speech and language therapy can be of considerable benefit to children with SCDs, though they need to know what they're doing

$R_e5KJQmN6txthTRX$	Yes. Many of the children we represent have social communication disorders and benefit hugely from SLT, but it is a skilled area and therapists need to know what they're doing. / Some children with SLI have or go on to develop some social communication difficulties, but do not have a social communication disorder. They nevertheless need a social skills programme such as SULP
$R_71b9fvukXBUQ5dr$	But - tiny numbers in current research studies. Why use DSM5 - WHO ICD is Europe/worldwide standard.
$R_7WXquZJy8WlgXAx$	as above
$R_eG1jl51DiHRqXKB$	There are also some other professionals who work with these children, e.g., occupational therapists

2.8 Final comment

Table 47: Comments for each statement.

ResponseID	final.comment
$R_5uxk08XTwJpUk9D$	I was forced to answer Q1 (whether I work in a clinical/teaching field) –I don't work in any of the professions listed! / / I thought this was an excellent survey covering a wide range of relevant issues - the questions were very thought-provoking.
$R_2f9ctxaHBJuJdLD$	There is often an overlap with receptive language disorders and ASD and an association with particular language profiles- how do we tease out the comorbidity and do we treat them any differently because of the comoribidity? Also sli vs SLI- there is confusing terminology out there!
$R_6RlkuyWJYcIIsmN$	I'd like to see a broad-ranging diagnostic classification system that allows for different, but equally important types of language impairment. The diagnostic system should reflect the diversity and complexity of the LI territory - it should not try to trim away messy details that interfere a desire (not matter how well motivated) for with a "neat" diagnostic framework. Comorbidities are the norm and we just need to deal with this, and will need to educate policy-makers and funding bodies accordingly. / / Thanks for undertaking this research and including me in it!
$R_6JOosydU46ZndMF$	Developmental changes & impact of co-occurring conditions are critical to consider for virtually every topic identified
$R_1L0uyOsRR9gYKAB$	role of investigations and role for screening siblings
$R_e9cPjWuFpcer4B7$	It is difficult to generate a set of questions that will work across a widely variety of contexts/countries where the way that language impairment is often construed in very different ways. / / The focus seems to be on speech language pathologists/therapists. This is fine except of course that the vast majority (60% from various studies) of these children are managed in mainstream schools and rarely, if ever get to see a therapist. So I would say that the educationalist's perspective is largely missing here. / / We know that the profile of these children changes dramatically across time but there is little or nothing about the time sensitive nature of the needs of these children from earliest identification though to adulthood. It is quite possible that the age of the child would affect the answer to the questions. / / It would be helpful to rework some of these questions to tease out some of the issues.
$R_9uJ5LinD5e8X5Yh$	A very thorough survey of all the chestnuts in the SLI debate (over many years!). Did I mention the vital need for an educational perspective when we make our decisions........but my books will be finished soon so you can use those! Or me! / Thank you for taking this on........really exciting work.
$R_3pDedyU4fM1kOXj$	There are NICE guidelines regarding the amount of therapy that is recommended for a child with a diagnosis of ASD. It would be helpful to have similar guidelines for children with SLI. Perhaps we could have some discussions about the amount of therapy that is recommended for a child with SLI?
$R_bwwc7dPFEcp1azH$	I look forward to review on the terminology issues (e.g. SLI/ LI/ PLI) etc as this is relevant to this discussion, although the definitions of the condition as outlined here do need consideration. How these apply to the DSM-V and ICD-11 are problematic as they seem disconnected, particularly if we are to achieve consensus across countries and disciplines given the current disparities
$R_dguQPTfUoDzSKB7$	i think dealing with language learners and effects of bilingualism complicates assessment and intervention. so addressing all the langauges the children speak is critical. / I think many schools do not allow the diagnosis of LI when there is no signficant discrepancy; however, many of these children struggle and need specailized intervention. /
$R_6Dvhy7Alhw5wqIR$	severity as an important factor / age at assessment important

$R_2o7JoTNgC3lqSIR$	I think one issue that has not been looked at is the lack of adequate 'medical' terminology. Most of the terms used in this survey are ordinary English words that can be used in a descriptive way. At what point does a child with a language impairment stop being a child who has some difficulties with language and become a child with a genuine condition? It is not clear in the language and as a result different people use the words to mean different things. It would be really useful if we had a term for 'severe, long-term impairment' that we could use to say child X has SLCN or a language delay etc. but child Y has (whatever), which everyone would understand to mean a medically diagnosed problem with language. / This questionnaire seems to be very SLT/Researcher focused. It takes no account at all of the role of education services in, at the very least identification and support, if not diagnosis.
$R_e5KJQmN6txthTRX$	Too mucn focus on SLT/health. No account taken of education and other frontline services: children's centres etc. health visitors. / No account of the parental perspective. Very research rather than practice focused. No real account taken of real world assessments and situations. / Diagnostic terminology and consistency a huge problem for us as an organisation and our families. It is essential to seize this opportunity and clarify what terms should be used about whom when. The nature and impact of language impairment is poorly understood and as a result it's tagged onto more tangible issues such as social deprivation, literacy (though the link is not clearly understood), English as a second language (skewing the numbers, according to the BCRP findings)
$R_1QTm7VrpDX1OAi9$	Thank you for all your work on this. I look forward to seeing the results and hope we can reach some kind of consensus!
$R_3rrKtkb2VvC3uG9$	the term "language processing" is overly broad and potentially confusing, creating interpretation problems / / the term "language impairment" is overly broad and potentially confusing, creating interpretatoin problems
$R_3DfMsLnqK54HqcZ$	The starting point that this process refers to children who will get specialist input is potentially problematic. / / As previous custom and practice has been to give specialist services to children who fit SLI criteria we don't have the research or practice experience to decide who else might benefit from specialist services. there is the potential for bias towards saying it is those children who we currently see for specialist services who need specialist services. / / It also does not tackle the very real issue of transient versus persistent LI and when (i.e. at what age) might we decide LI is persistent. / / Also it constrains the debate such that other staged models of diagnosis linked to preventative interventions and change over developmental time cannot be considered. / / I can see the debate need to be constrained in some way but I fear this is an unhelpful starting point and may significantly limit the debate. / /
$R_eG1jl51DiHRqXKB$	I don't think I can add to the comprehensive set of questions asked above.
$R_8bIXFrv4VBlvVyZ$	(i) It may be useful to explore & possibly reach a consensus regarding specific assessment areas to be considered at different ages in the identification of language impairment. / (ii) we need greater consideration of the cognitive, motor and perceptual dimensions of language impairment in developing a consensus in this area / (iii) if the consensus gathering includes only clinicians/academics/researchers coming from an SLI perspective we may miss an opportunity to identify similarities and differences in descriptors/symptoms and underpinning characteristics across current diagnostic groups (e.g. SLI, ASD, HI, DS etc). Having this wider perspective might usefully inform more robust knowledge on dimensions of impairment and help us to move away from strict categorical approches. I appreciate this may have been attempted in the development of the DSMV and not succeeded but this exercise may present such an opportunity. / (iii) for children with LI, differences in the theoretical understanding of SLTs v Educators v Psychologists etc may be contributing to some of the challenges we currently experience in the planning of and access to service delivery. Again, considering those differences in perspective may move the field forward in a shared understanding of LI.

Contact Information

Oxford Study of Children's Communication Impairments (OSCCI),

Department of Experimental Psychology,

Tinbergen Building,

South Parks Road,

Oxford,

OX1 3UD,

UK.

Tel. (+44) 01865 271386

fax. (+44) 01865 281255

Email. oscci@psy.ox.ac.uk

Centre for Reading & Language @ Oxford,

Department of Experimental Psychology,

Tinbergen Building,

South Parks Road,

Oxford,

OX1 3UD,

UK.

Tel. (+44) 01865 277419

Email. CRLOxford@psy.ox.ac.uk

Health Experiences Research Group,

Nuffield Department of Primary Care Health Sciences,

Radcliffe Observatory Quarter,

Woodstock Road,

Oxford,

OX2 6GG,

UK.

Tel. (+44) 01865 289363

Email. trish.greenhalgh@phc.ox.ac.uk

Copyright Information

This copy of the CATALISE Delphi results has been supplied on the condition that anyone who consults it recognizes that its copyright rests with the authors and that no quotation from the report or information derived from it may be published without prior consent.
