# Supplementary material for: CATALISE: A Multinational and Multidisciplinary Delphi Consensus Study. Identifying Language Impairments in Children
Source: PLoS One. 2016 Jul 8;11(7):e0158753. doi: 10.1371/journal.pone.0158753 (PMC4938414; doi:10.1371/journal.pone.0158753)

Round 1 item	Wording for Round 2
33, 37	1. Reasons for referral for specialist assessment/intervention include concern about speech, language or communication expressed by caregivers or teachers, or a lack of progress despite targeted classroom assistance. [1]*
44, 45	2. Language impairments may go undetected. Referral for language assessment is recommended for children who present with unexplained behavioural or psychiatric difficulties, and for children with poor reading or listening comprehension. [2]
VB	3. Between 1 to 2 years of age, the following features are indicative of atypical development in speech, language or communication: (a) No babbling (b) Not responding to speech and/or sounds. [4]
23, 24	4. Many late-talkers (children with limited vocabulary at 18-24 months) catch up without any special help. We have only limited ability to predict which children will go on to have longer-term problems. Children at greatest risk of persisting problems are late-talkers with poor language comprehension, poor use of gesture, and/or a family history of language impairment. [3]
VB	5. Between 2 to 3 years of age, the following features are indicative of atypical development in speech, language or communication: (a) No interaction; (b) Does not display intention to communicate; (c) No words; (d) No/minimal reaction to spoken language. [5]
VB	6. Between 3 to 4 years of age, following features are indicative of atypical development in speech, language or communication: (a) No speech; (b) At most two-word utterances; (c) Not intelligible to close relatives; (d) Does not understand simple commands. [6]
VB	7. Between 4 to 5 years of age, the following features are indicators of atypical language development: (a) Inconsistent or abnormal interaction (b) At most three word utterances (c) Not intelligible to strangers (d) Parents cannot understand more than half of what child says (e) Poor understanding of spoken language. [7]
33	8. Children's language can change dramatically, especially in the preschool/early school years (aged 4 to 5 years), even if there is no intervention. However, severe language impairment involving both comprehension and expression is more likely to be persistent. [8]
	9. From 5 years of age upwards, the following features are indicators of atypical language development: (a) Difficulty in telling or re-telling a coherent story (producing narrative) (b) Difficulty in understanding what is read or listened to (c) Marked difficulty in following or remembering spoken instructions (d) Talking a lot but very poor at engaging in reciprocal conversation (e) Many instances of over-literal interpretation, missing the point of what was meant [9]

KEY
% Agree
- >90
- >80
- >79
- >60
- >50
- >40
- >30
- >20
- >10

* denotes number of corresponding final statement

Round 1 item	Wording for Round 2
25	10. A staged approach to language assessment is efficient, with an initial omnibus test that taxes both receptive and expressive language (e.g. tests involving narrative retelling and/or sentence repetition), to establish severity of impairment, followed by more specific assessments as necessary. [14]
31	11. A well-standardized test that has good reliability, validity and sensitivity can quantify severity of impairment relative to a peer group in a relatively objective manner, but other types of assessment can provide complementary information. [10]
2	12. A low score on a language test is not the same as a need for intervention; the functional impact needs to be taken into account. [11]
1, 3	13. There is no clear cut-off that distinguishes between language impairment (regardless of its cause) from the lower end of normal variation of language ability. [12]
17	14. Currently available assessments do not show clear language profile associated with social disadvantage. [15]
18	15. If a child with English as an Additional Language (EAL) learns English more slowly than their peers from the same language background, an assessment in the home language should be conducted to clarify whether additional support from a Speech and Language Therapist/Pathologist is needed. [18]
26	16. 'Markers' for language impairment which give good agreement with clinical diagnosis are nonword repetition, sentence repetition, and production of verb inflections. [16]
28, 29, 30	17. Dynamic assessment that explores how children learn seems a promising approach. In principle it might help distinguish children whose difficulties are simply due to lack of exposure from those whose learning is impaired. However, more research is needed to develop approaches to dynamic assessment that could be recommended for this purpose. [17]
34	18. For comparing rates of language impairment over time, or in different places, it would be useful to have a standard assessment process, e.g. a test battery which used a statistical definition. [13]

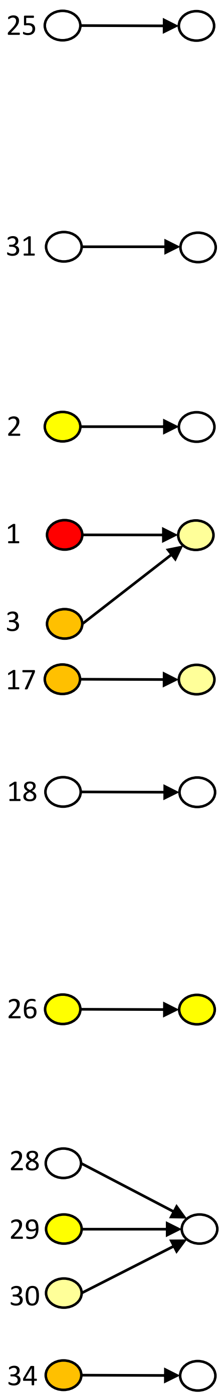

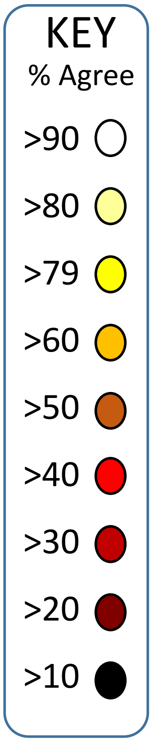

Round 1 item

Wording for Round 2

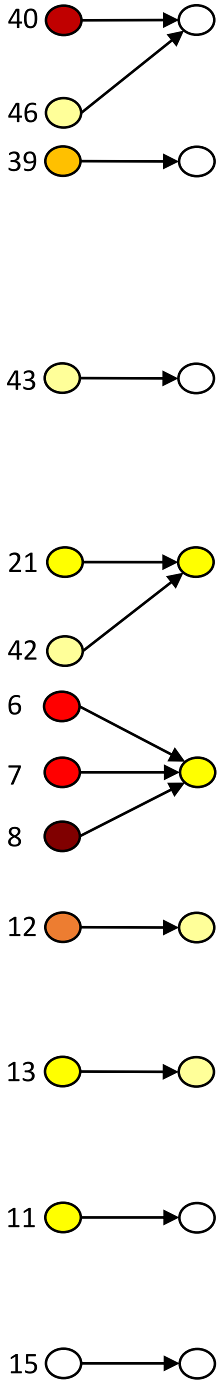

19. Speech and language therapists/pathologists have the skills to assess and plan intervention for children who have pragmatic difficulties (including those diagnosed with social communication disorder). [19]

20. Speech and language therapists/pathologists have specialist expertise in the assessment of problems with production of speech sounds, many of which are linguistic rather than motor/structural in origin. Speech difficulties can occur separately from or together with other language difficulties, and have different prognosis and intervention needs. [20]

21. Language impairment frequently co-occurs with other neurodevelopmental difficulties, including attentional problems, motor impairments, reading difficulties, social impairment and behaviour problems. [21]

22. If research is restricted to those with 'pure' language impairments, it will have little relevance for clinical practice since most language impaired children have a range of other problems. [22]

23. Where a child's nonverbal functioning is more than two standard deviations below average, the primary diagnosis should be intellectual disability. For children who function above that level, language impairment should be identified regardless of whether there is a mismatch with nonverbal ability. [23]

24. The language difficulties of children with autism spectrum disorder (ASD) normally require a different approach to intervention to those of nonautistic children. [24]

25. Children with known syndromes (e.g. Down syndrome, Klinefelter syndrome) often have accompanying language problems that resemble those seen in children with no known aetiology. [25]

26. Children with acquired language impairment (e.g. caused by traumatic brain injury) are likely to have a different prognosis from those with developmental problems with no acquired aetiology. [26]

27. Hearing impairment and language impairment can co-occur, as demonstrated by cases of children whose language abilities – in either spoken or signed language – are well below those of their hearing-impaired peer group. [27]

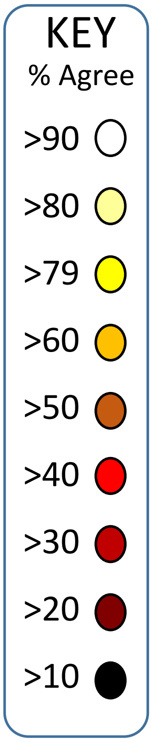

Supplement: S4 Doc — Final item number shown in square brackets. (PDF) [file pone.0158753.s004.pdf]
