# Supplementary material for: CATALISE: A Multinational and Multidisciplinary Delphi Consensus Study. Identifying Language Impairments in Children
Source: PLoS One. 2016 Jul 8;11(7):e0158753. doi: 10.1371/journal.pone.0158753 (PMC4938414; doi:10.1371/journal.pone.0158753)
Supplement: S5 Doc — (PDF) [file pone.0158753.s005.pdf]

# CATALISE ROUND 2: **Background report**

## Preamble

Many thanks to all our panel members. It has been a fascinating process looking at the pattern of data and reading your comments. We are excited about the potential this project has for moving the field forward.

Consideration of responses to round 1 led to some general insights as well as ideas for dropping/rewording/combining items. In this document, we list a revised set of 27 items which you will be asked to respond to in round 2.

In round 1, all agreed that language impairment changes with age, and we felt further specification would be useful. We were helped here by a very timely study by Visser-Bochane et al (2015) who conducted a Delphi in the Netherlands which included specification of 'red flags' appropriate for different age ranges. This study is not yet published, but the authors kindly let me have a copy of a presentation they gave in May and have me permission to adapt their items for inclusion in our Delphi. What they term 'red flags' are aspects of language development that are clearly atypical at a given age; a child would not be required to show these features to be regarded as giving concern, but presence of these features would prompt further assessment/referral. Your ratings of these items will indicate whether we can include them, and will give a preliminary indication of the extent to which there is cross-national agreement.

The current document allows you to preview items, and explains how they relate to the previous items from round 1. Also, we are providing here (but not on the online questionnaire) some additional explanation and rationale, as well as references to research papers where appropriate, outlining what we know about the evidence base. Ultimately, this additional information would be incorporated in the CATALISE report for items where there is reasonable consensus. We therefore ask panel members to **please use the comments box on the online questionnaire** to comment not just on the briefer wording of the new items, but also to add to the rationale given here, or to challenge anything that seems to misrepresent research knowledge. References are currently skewed towards our own UK work. Feel free to suggest alternatives or additions. Also, much research in this area is far from perfect and research evidence does not always agree with insights by practitioners; panel members should feel free to speak out if they feel that the research evidence cited is leading us in the wrong direction.

Just to confirm, the only person who has the link between identity of respondents and the response forms is Paul Thompson, who does not know any of you, so please don't feel constrained about giving your views.

## A note on round 1 relevance ratings

It was clear from comments that many people were uncertain about what the relevance rating encompassed. We should have given clearer instructions: essentially the aim was to find out whether the topic of the item was relevant for the exercise of finding statements we could agree on, regarding identification of children requiring additional support. The median response to these questions was 6 (agree) or 7 (strongly agree) for all but 2 items. Because of this we have dropped relevance ratings for round 2, and to keep things simple, we have not included them in your personalised report.

The two items where there was less consensus on relevance (i.e. several respondents thought they should not be included in future rounds) were:

5. Any definition of language impairment should take into account what services are available/affordable.

33. We should only consider a diagnosis of language impairment when language has been shown to be impaired at two time points 12 months apart.

Both of these items have been dropped from round 2, but please note that the issue of available resources is mentioned at the end of the document, and the issue of changes in language profile over time is now incorporated in a new item on longitudinal aspects of LI.

## New items for round 2

We had planned to simply retain items with good agreement, but we have gone beyond this on the basis of comments made by panel members, and dropped, collapsed and reworded many items. The whole process should be transparent – your personalised report gives the distribution of ratings and the comments, and this document explains the relationship between items in round 1 and round 2. However, it is complex, and so please use comments to let us know if you are concerned about any aspect of how we have developed round 2 items from round 1.

The round 2 items are grouped into three broad categories; referral for specialist assessment/intervention, assessment, and accompanying conditions

### A. When should a child be referred for specialist assessment/intervention?

#### 1. Reasons for referral for specialist assessment/intervention include concern about speech, language or communication expressed by caregivers or teachers, or a lack of progress despite targeted classroom assistance.

Based on: original items 33,  37

#### 2. Language impairments may go undetected. Referral for language assessment is recommended for children who present with unexplained behavioural or psychiatric difficulties, and for children with poor reading or listening comprehension.

Based on: original item 44-45

Reference

Cohen, N. J., Davine, M., & Kelly-Meloche, M. (1989). Prevalence of unsuspected language disorders in a child psychiatric population. Journal of the American Academy of Child and Adolescent Psychiatry, 28, 107-111.

Dawes, P., & Bishop, D. (2010). Psychometric profile of children with auditory processing disorder (APD) and children with dyslexia. Archives of Disease in Childhood, 95, 432-436.

Nation, K., Clarke, P., Marshall, C. M., & Durand, M. (2004). Hidden language impairments in children: parallels between poor reading comprehension and specific language impairment? Journal of Speech, Language, and Hearing Research, 47, 199-211.

## 3. Between 1 to 2 years of age, the following features are indicative of atypical development in speech, language or communication:  (a) No babbling (b) Not responding to speech and/or sounds

Note that this items and subsequent items from Visser-Bochane describe behaviours that most experts regarded as **definitely atypical** at this age. We are not saying that children who meet these minimum levels of language and communication have no problems! The more general aspects in items 1-2 need to be taken into account.

Item based on Delphi by Visser-Bochane et al (2015)

## 4. Many late-talkers (children with limited vocabulary at 18-24 months) catch up without any special help. We have only limited ability to predict which children will go on to have longer-term problems. Children at greatest risk of persisting problems are late-talkers with poor language comprehension, poor use of gesture, and/or a family history of language impairment.

The desirability of early intervention needs to be calibrated against the disadvantages of intervening with children who would outgrow their problems without any special help.

Based on: original item 23-24

References

Reilly, S., Wake, M., Ukoumunne, O. C., Bavin, E., Prior, M., Cini, E., . . . Bretherton, L. (2010). Predicting language outcomes at 4 years of age: Findings from Early Language in Victoria Study. [Article]. Pediatrics, 126(6), E1530-E1537. doi: 10.1542/peds.2010-0254

Zambrana, I. M., Pons, F., Eadie, P., & Ystrom, E. (2014). Trajectories of language delay from age 3 to 5: persistence, recovery and late onset. International Journal of Language and Communication Disorders, 49(3), 304-316. doi: 10.1111/1460-6984.12073

## 5. Between 2 to 3 years of age, the following features are indicative of atypical development in speech, language or communication: (a) No interaction; (b) Does not display intention to communicate; (c) No words; (d) No/minimal reaction to spoken language

Item based on Delphi by Visser-Bochane et al (2015)

## 6. Between 3 to 4 years of age, following features are indicative of atypical development in speech, language or communication: (a) No speech; (b) At most two-word utterances; (c) Not intelligible to close relatives; (d) Does not understand simple commands

Item based on Delphi by Visser-Bochane et al (2015)

### 7. Between 4 to 5 years of age, the following features are indicators of atypical language development

(a) Inconsistent or abnormal interaction (b) At most three word utterances (c) Not intelligible to strangers (d) Parents cannot understand more than half of what child says (e) Poor understanding of spoken language

Reference: Item based on Delphi by Visser-Bochane et al (2015)

### 8. Children's language can change dramatically, especially in the preschool/early school years (aged 4 to 5 years), even if there is no intervention. However, severe language impairment involving both comprehension and expression is more likely to be persistent.

The implication is that a staged approach is optimal for most children in the early school years, with specialised provision considered for those children who do not respond to targeted intervention provided by teachers, or who have severe problems.

Based on: original item 33

Reference

Bishop, D. V. M., & Edmundson, A. (1987). Language-impaired four-year-olds: distinguishing transient from persistent impairment. Journal of Speech and Hearing Disorders, 52, 156-173.

Dockrell, J., Ricketts, J., & Lindsay, G. (2012). Understanding speech, language and communication needs: profiles of need and provision Better Communication Research Programme. London: Department for Education.

### 9. From 5 years of age upwards, the following features are indicators of atypical language development: (a) Difficulty in telling or re-telling a coherent story (producing narrative) (b) Difficulty in understanding what is read or listened to (c) Marked difficulty in following or remembering spoken instructions (d) Talking a lot but very poor at engaging in reciprocal conversation (e) Many instances of over-literal interpretation, missing the point of what was meant

This item was newly created as an extension of the Visser-Bochane approach.

## Aspects of language assessment

### 10. A staged approach to language assessment is efficient, with an initial omnibus test that taxes both receptive and expressive language (e.g. tests involving narrative retelling and/or sentence repetition), to establish severity of impairment, followed by more specific assessments as necessary.

There are many components to language, but it is seldom feasible to evaluate all of them in an initial assessment, even if suitable instruments are available. Results from an omnibus test should be interpreted in relation to information about functional impairment. More specific evaluation of component skills can be conducted alongside intervention and be informed by response to intervention.

Based on: original item 25

Bishop, D. V. M., & McDonald, D. (2009). Identifying language impairment in children: combining language test scores with parental report. International Journal of Language and Communication Disorders, 44, 600-615.

## 11. A well-standardized test that has good reliability, validity and sensitivity can quantify severity of impairment relative to a peer group in a relatively objective manner, but other types of assessment can provide complementary information.

Standardized age-normed tests, criterion-referenced and observational measures have different strengths and weaknesses for assessment of language impairment. All may play a role, depending on the purpose of assessment. Standardized scores are helpful in documenting severity of a language problem, but are usually blunt instruments for measuring change over time. A criterion-referenced test can help pinpoint targets for intervention, but the significance of impairments will be age-dependent. Observational assessment gives an indication of how the child functions in a more naturalistic setting, but reliability of observation can be hard to establish, and interpretation depends heavily on the experience of the clinician.

Based on: original item 31

Reference

Paul, R., & Norbury, C. F. (2012). Language Disorders from Infancy through Adolescence: Assessment and Intervention, 4th Edition. St. Louis: Mosby-Year Book.

## 12. A low score on a language test is not the same as a need for intervention; the functional impact needs to be taken into account.

Information from caregivers and teachers is important for identifying what the main presenting issue is, which; this will inform which targets are selected for intervention.

Based on: Original item 2

Reference

World Health Organization. (2007). International classification of functioning, disability and health: children & youth version: ICF-CY. Geneva: World Health Organization.

## 13. There is no clear cut-off that distinguishes between language impairment (regardless of its cause) from the lower end of normal variation of language ability.

Language impairment can be a secondary consequence of known conditions, such as hearing loss, genetic syndromes, or epilepsy, but in many cases there is no known cause, and no clear cutoff between impairment and normal variation. Regardless of the cause, where a person's language abilities fall at the low end of the normal range of language, it can be appropriate to recommend intervention, ranging from environmental adjustments to specialised help, depending on the

severity and nature of the problems and accompanying risk factors. Obesity and high blood pressure provide useful analogies: both are conditions that can arise for a range of reasons, but there is often no obvious cause, and the cutoff between normal and abnormal is arbitrary; nevertheless, those falling in the more extreme range merit intervention.

Based on: Original items 1 and 3

References

Leonard, L. B. (1991). Specific language impairment as a clinical category. Language, Speech, and Hearing Services in Schools, 22, 66-68.

Dollaghan, C. A. (2011). Taxometric analyses of Specific Language Impairment in 6-year-old children. Journal of Speech, Language and Hearing Research, 54(5), 1361-1371. doi: 10.1044/1092-4388(2011/10-0187)

## 14. Currently available assessments do not show clear language profile associated with social disadvantage.

Low socio-economic status is associated with language impairment, but it is unclear what causal pathways are involved.  Since language difficulties may play out differently in different contexts, socio-economic status may affect response to intervention.

Based on: original item 17

Reference

Roy, P., & Chiat, S. (2013). Teasing apart disadvantage from disorder: The case of poor language. In C. R. Marshall (Ed.), Current Issues in Developmental Disorders (pp. 125-150). Hove: Psychology Press.

## 15. If a child with English as an Additional Language (EAL) learns English more slowly than their peers from the same language background, an assessment in the home language should be conducted to clarify whether additional support from a Speech and Language Therapist/Pathologist is needed.

Many children develop as skilled bilinguals. Nevertheless, research shows that EAL is a risk factor for poor academic achievement, and some children will benefit from additional language support in the classroom.

Based on: original item 18

Reference

Strand, S., Malmberg, L., & Hall, J. (2015). English as an Additional Language (EAL) and educational achievement in England: An analysis of the National Pupil Database: University of Oxford, Department of Education.

## 16. 'Markers' for language impairment which give good agreement with clinical diagnosis are nonword repetition, sentence repetition, and production of verb inflections.

It is not, however, established how these markers align with functional impairments. Research showing that a 'marker' distinguishes a language impaired group from a matched control group does not guarantee it would show sensitivity and specificity in a clinical context.

Based on: original item 26

References

Archibald, L. M. D., & Joanisse, M. F. (2009). On the Sensitivity and Specificity of Nonword Repetition and Sentence Recall to Language and Memory Impairments in Children. Journal of Speech Language and Hearing Research, 52(4), 899-914. doi: 10.1044/1092-4388(2009/08-0099)

Conti-Ramsden, G., & Hesketh, A. (2003). Risk markers for SLI: a study of young language-learning children. International Journal of Language and Communication Disorders, 38(3), 251-263. doi: 10.1080/1368282031000092339

Deevy, P., Weil, L. W., Leonard, L. B., & Goffman, L. (2010). Extending Use of the NRT to Preschool-Age Children With and Without Specific Language Impairment. Language Speech and Hearing Services in Schools, 41(3), 277-288. doi: 10.1044/0161-1461(2009/08-0096)

Redmond, S. M., Thompson, H. L., & Goldstein, S. (2011). Psycholinguistic profiling differentiates Specific Language Impairment from typical development and from Attention-Deficit/Hyperactivity Disorder. Journal of Speech Language and Hearing Research, 54(1), 99-117. doi: 10.1044/1092-4388(2010/10-0010)

Rice, M. L., & Wexler, K. (1996). Toward tense as a clinical marker of specific language impairment in English-speaking children. Journal of Speech and Hearing Research, 39, 1239-1257.

## 17. Dynamic assessment that explores how children learn seems a promising approach. In principle it might help distinguish children whose difficulties are simply due to lack of exposure from those whose learning is impaired. However, more research is needed to develop approaches to dynamic assessment that could be recommended for this purpose.

Based on: original item 28-30

Reference

Hasson, N., Camilleri, B., Jones, C., Smith, J., & Dodd, B. (2013). Discriminating disorder from difference using dynamic assessment with bilingual children. Child Language Teaching and Therapy, 29(1), 57-75. doi: 10.1177/0265659012459526

## 18. For comparing rates of language impairment over time, or in different places, it would be useful to have a standard assessment process, e.g. a test battery which used a statistical definition.

An example of this, used in an epidemiological context, is the EPSLI criterion adopted by Tomblin and colleagues. Note, however, that many children identified by this criterion had not been identified by parents or professionals as having language difficulties.

Based on: original item 34

Reference

Tomblin, J. B., Records, N., & Zhang, X. (1996). A system for the diagnosis of specific language impairment in kindergarten children. Journal of Speech and Hearing Research, 39, 1284-1294.

## 19. Speech and language therapists/pathologists have the skills to assess and plan intervention for children who have pragmatic difficulties (including those diagnosed with social communication disorder)

This item relates to the construct of pragmatic language impairment, a term used to refer to cases of non-autistic children with poor pragmatic skills. Some of these children also have structural language problems, but others do not. The term 'social communication disorder' (SCD) is very close in meaning to the term 'pragmatic language impairment', which has been adopted in the UK, but does not have any formal status. There has been concern that social communication disorder has been introduced in DSM-5 without any validation studies, and without clear diagnostic guidelines. In the absence of relevant data, we recommend that speech and language therapists/pathologists are the appropriate profession to work with children who have pragmatic difficulties (including those diagnosed with SCD).

Based on: original item 40 and 46

Reference

Bishop, D. V. M. (2000). Pragmatic language impairment: a correlate of SLI, a distinct subgroup, or part of the autistic continuum? In D. V. M. Bishop & L. B. Leonard (Eds.), Speech and Language Impairments in Children: Causes, Characteristics, Intervention and Outcome (pp. 99-113). Hove, UK: Psychology Press.

Norbury, C. F. (2014). Practitioner Review: Social (pragmatic) communication disorder conceptualization, evidence and clinical implications. Journal of Child Psychology and Psychiatry, 55(3), 204-216. doi: 10.1111/jcpp.12154

## 20. Speech and language therapists/pathologists have specialist expertise in the assessment of problems with production of speech sounds, many of which are linguistic rather than motor/structural in origin. Speech difficulties can occur separately from or together with other language difficulties, and have different prognosis and intervention needs.

Problems with expressive phonology are identified when the child collapses or substitutes phonological categories despite there being no structural or motor reason for this. Such problems have not been treated consistently in systems of terminology and classification. Because phonology is part of language, one can make a logical case that they should be categorised as part of language impairment. In practice, however, difficulties restricted to production of speech sounds often occur separately from other language difficulties, and have different prognosis and intervention needs. Therefore, if these are included as part of language impairment, they need to be recognised as a distinct subgroup.

Based on: original item 39

Reference

Shriberg, L. D., Tomblin, J. B., & McSweeny, J. L. (1999). Prevalence of speech delay in 6-year-old children and comorbidity with language impairment. Journal of Speech, Language and Hearing Research, 42(6), 1461-1481.

## Relation of language impairment to other developmental difficulties

### 21. Language impairment frequently co-occurs with other neurodevelopmental difficulties, including attentional problems, motor impairments, reading difficulties, social impairment and behaviour problems.

Such co-occurring problems are not a reason for ignoring a language impairment; they should be noted, as it is likely that the presence of these additional difficulties affect prognosis and intervention strategies. A multidisciplinary approach to assessment and intervention can be useful to give a full picture of the child's needs.

Based on: original item 43

Reference

Bishop, D., & Rutter, M. (2008). Neurodevelopmental disorders: conceptual issues. In M. Rutter, D. Bishop, D. Pine, S. Scott, J. Stevenson, E. Taylor & A. Thapar (Eds.), Rutter's Child and Adolescent Psychiatry (pp. 32-41). Oxford: Blackwell.

### 22. If research is restricted to those with 'pure' language impairments, it will have little relevance for clinical practice since most language impaired children have a range of other problems.

Criteria for language impairment will depend to some extent on the question being asked, and there will be occasions when researchers need to adopt narrow, exclusionary criteria to minimise confounding and define a homogeneous group; however, there is now ample evidence that most children with clinically significant language impairments have additional problems, and so if research is restricted to those with 'pure' language impairments, it may have little relevance for clinical practice.

Based on: original item 21 and 42

Reference

Stark, R. E., & Tallal, P. (1981). Selection of children with specific language deficits. Journal of Speech and Hearing Disorders, 46, 114-122.

## 23. Where a child's nonverbal functioning is more than two standard deviations below average, the primary diagnosis should be intellectual disability. For children who function above that level, language impairment should be identified regardless of whether there is a mismatch with nonverbal ability.

Based on: Original items 6, 7, and 8

References

Reilly, S., Tomblin, B., Law, J., McKean, C., Mensah, F., Morgan, A., . . . Wake, M. (2014). SLI: a convenient label for whom? International Journal of Language and Communication Disorders.

## 24. The language difficulties of children with autism spectrum disorder (ASD) normally require a different approach to intervention to those of nonautistic children.

Based on: original item 12

References:

Kjelgaard, M. M., & Tager-Flusberg, H. (2001). An investigation of language impairment in autism: Implications for genetic subgroups. Language and Cognitive Processes, 16, 287-308.

Williams, D., Payne, H., & Marshall, C. (2013). Non-word repetition impairment in autism and Specific Language Impairment: Evidence for distinct underlying cognitive causes. Journal of Autism and Developmental Disorders, 43(2), 404-417. doi: 10.1007/s10803-012-1579-8

## 25. Children with known syndromes (e.g. Down syndrome, Klinefelter syndrome) often have accompanying language problems that resemble those seen in children with no known aetiology.

There is little data on interventions for these groups; it seems plausible they would respond to the types of intervention used with children whose language difficulties have no known cause.

Based on: original item 13

References

Laws, G., & Bishop, D. V. M. (2003). A comparison of language abilities in adolescents with Down syndrome and children with specific language impairment. Journal of Speech, Language and Hearing Research, 46, 1324-1339.

Bishop, D. V. M., & Scerif, G. (2011). Klinefelter syndrome as a window on the etiology of language and communication impairments in children. Acta Paediatrica, 100(6), 903-907. doi: DOI: 10.1111/j.1651-2227.2011.02150.x

### 26. Children with acquired language impairment (e.g. caused by traumatic brain injury) are likely to have a different prognosis from those with developmental problems with no acquired aetiology.

Based on: original item 11

References:

Paquier, P., & van Dongen, H. R. (1996). Review of research on the clinical presentation of acquired childhood aphasia. Acta Neurol Scand, 93, 428-436.

### 27. Hearing impairment and language impairment can co-occur, as demonstrated by cases of children whose language abilities – in either spoken or signed language – are well below those of their hearing-impaired peer group.

Moderate-severe-profound hearing loss is associated with difficulties in learning oral language, but most hearing-impaired children demonstrate normal language skills if exposed to a sign language early in life. Nonetheless, it is possible to have an impairment in sign language, just as in spoken language. In a similar vein, some children make unexpectedly poor progress with spoken language after a cochlear implant. Also, a mild hearing loss which is insufficient on its own to cause language difficulties may have an adverse impact when combined with other risk factors. Language assessment and intervention with hearing-impaired children requires specialist skills.

Based on: original item 15

References

Briscoe, J., Bishop, D. V. M., & Norbury, C. F. (2001). Phonological processing, language, and literacy: A comparison of children with mild-to-moderate sensorineural hearing loss and those with specific language impairment. Journal of Child Psychology and Psychiatry, 42, 329-340.

Hawker, K., Ramirez-Inscoe, J., Bishop, D. V. M., Twomey, T., O'Donoghue, G. M., & Moore, D. R. (2008). Disproportionate language impairment in some children using cochlear implants. Ear and Hearing, 29, 467-471.

Morgan, G., Herman, R., & Woll, B. (2007). Language impairments in sign language: breakthroughs and puzzles. Int J Lang Commun Disord, 42(1), 97-105.

## Future directions

For the future, we are considering the possibility that the results from CATALISE might be used to produce a decision tree in the form of a flowchart that shows pathways from referral.

Many of the comments are helping us crystalise an agenda for future research. In some cases, we have dropped items from the Delphi for lack of a clear evidence base, but we will flag these up when describing the project in a separate section on needs for future research.

Another issue that emerged was the distinction between what is desirable and what is available. There was strong agreement that decisions about identifying problems should not be influenced by

what we know about available resources. This issue will also be flagged up when we come to write up the study.
