# Supplementary material for: CATALISE: A Multinational and Multidisciplinary Delphi Consensus Study. Identifying Language Impairments in Children
Source: PLoS One. 2016 Jul 8;11(7):e0158753. doi: 10.1371/journal.pone.0158753 (PMC4938414; doi:10.1371/journal.pone.0158753)
Supplement: S6 Doc — Personalised version of this sent to all respondents, showing overall distribution of responses and qualitative comments. (PDF) [file pone.0158753.s006.pdf]

CATALISE

(Round 2)

Criteria and Terminology Applied to Language Impairments:

Synthesising the Evidence

Dorothy Bishop*[1], Maggie Snowling[1], Trish Greenhalgh[2] and Paul Thompson[1]

[1]Department of Experimental Psychology, Tinbergen Building, South Parks Road, Oxford, OX1 3UD, UK.

[2]Nuffield Department of Primary Care Health Sciences, New Radcliffe House, 2nd floor, Walton Street, Jericho, OX2 6NW, UK.

August 2015

Individual Report:

ANONYMOUS

*oscci@psy.ox.ac.uk

Contents

1 Summary **1**

1.1 Participants . . . 1

1.2 Overview of responses . . . 2

2 Delphi analysis results:Your responses relative to rest of panel **3**

2.1 When should a child be referred for specialist assessment/intervention? . . . 3

2.2 Aspects of language assessment . . . 26

2.3 Relation of language impairment to other developmental difficulties . . . 52

2.4 Final comment . . . 69

1 Summary

1.1 Participants

A multidisciplinary group of 60 experts from English-speaking countries in Europe, North America and Australasia were recruited to the study. The group comprised eight different diciplines and some combinations of disciplines (Audiology, N=1; Charity, N=4; Educational Psychologist, N=6; Paediatrician, N=3; Psychiatrist, N=2; Psychology, N=2; Speech and Language Therapist/pathologist (SLP), N=30; Specialist teacher, N=2; SLP/Ed Psych, N=5; SLP/Psych, N=2). One member opted out from the panel at the start of round one. A further two members failed to respond in time to be included into round two analyses. Figure 1 shows the breakdown of the group by discipline and country.

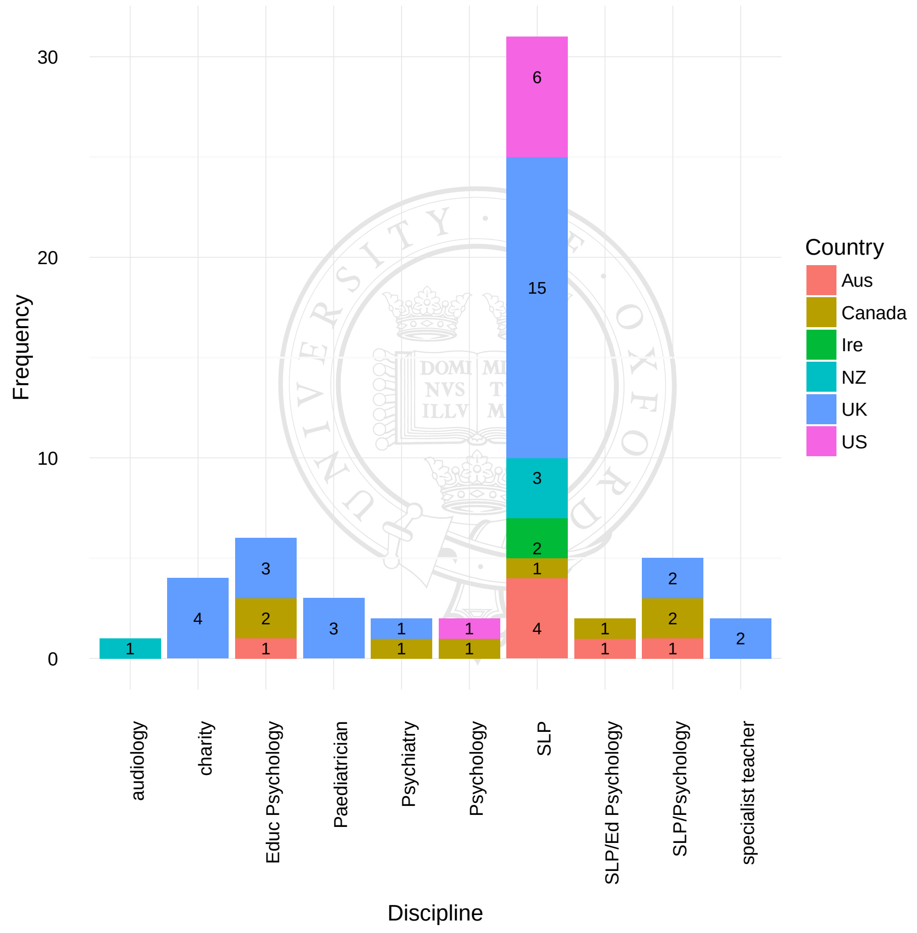

Figure 1: Number of participants summary by Discipline and Country

1.2 Overview of responses

Figure 2 shows an overview of the responses to all 27 statements according to Validity ('Do you agree with the statement?'). Each bar in the polar histogram represents a specific statements on Valdity and assigns a different colour for each response category in the Likert scale ('Strongly disagree' to 'Strongly Agree'). Within each bar, the percentage responded in each category is represented proportionally as the size of each coloured chunk.

The following section provides a more detailed investigation on an item-by-item basis. Furthermore, we include all the feedback commentary for each item from the panel.

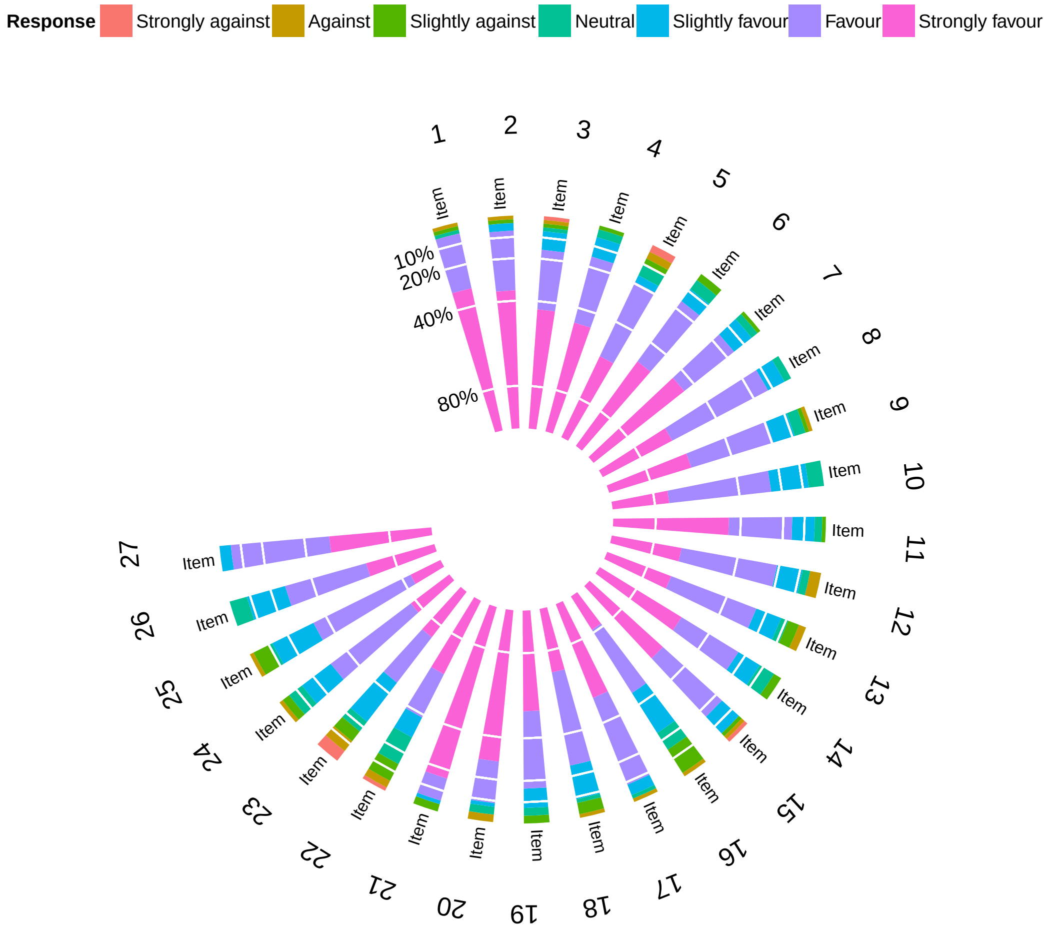

Figure 2: Overview of consensus by statement

2 Delphi analysis results:Your responses relative to rest of panel

2.1 When should a child be referred for specialist assessment/intervention?

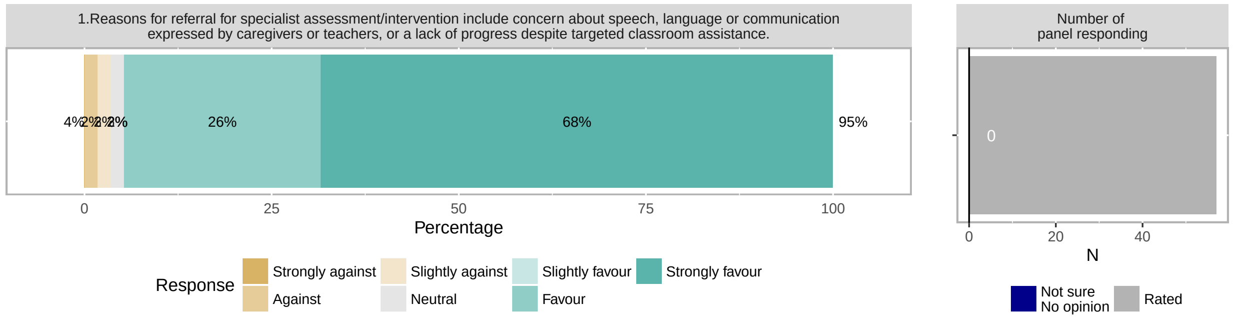

Figure 3: Percentage of panel members in each response category to statement 1. The percentages shown at each end of the scale are the cumulative percentages for the top and bottom three categories respectively.

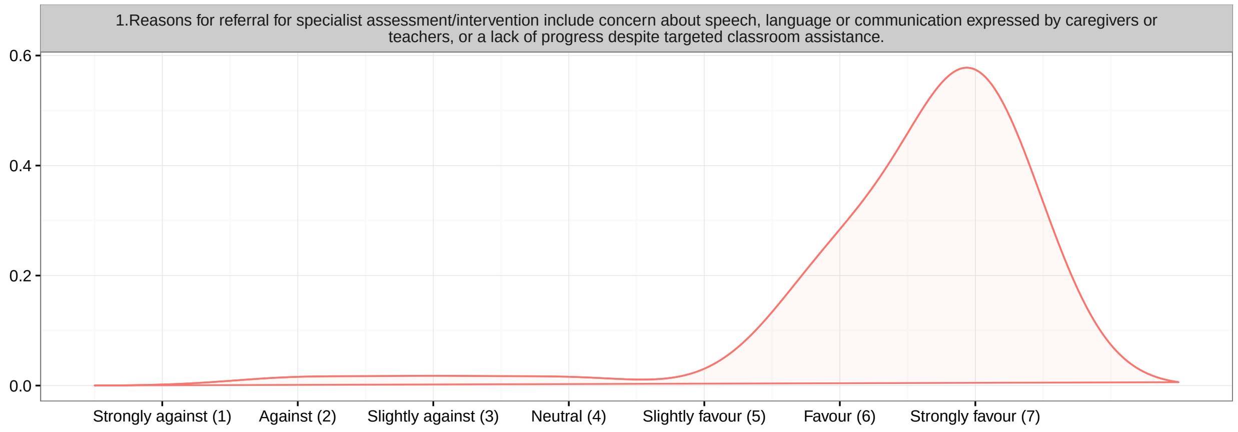

Figure 4: Distribution of responses to statement 1. The bold vertical line coloured red is Anonymous's response to this question for reference.

Table 1: Comments for each statement.

ResponseID	Q1B
$R_ba8iHG84IJ8cW7X$	It is important to include in all considerations the context and views others who have frequent contact with the child.
$R_5cd8BDkYcGfGLKl$	Your wording might want to be more specific regarding 'lack of progress' - in language development or in scholastic attainment?
$R_8AhxnQPe8mJkUoR$	Reference with supporting evidence that parental concern improves positive predictive value. / Klee, T., Pearce, K., & Carson, D. K. (2000). Improving the positive predictive value of screening for developmental language disorder. Journal of Speech, Language, and Hearing Research, 43(4), 821-833. /
$R_4HGIGYFIvMxLWcJ$	Favour - but I would like to see – across spoken and/.or written modalities included
$R_bQ13TaeUPFsxVJP$	I am happy with the rewording of this statement as it suggests referral for assessment rather than diagnosis. It also emphasises functional impairments in everyday life.
$R_bwwc7dPFEcp1azH$	It is useful to have parental and teacher views, and some information on general progress is helpful, although this should not be essential or a requirement as other practitioners (e.g. healthcare/medical) may have noticed a difficulty where parents/teachers have not

$R_2o7JoTNgC3lqSIR$	The question is poorly phrased. The term 'specialist assessment/intervention' is not defined. Do we mean speech and language therapy, teaching intervention, both or what? We assume parents are included within the 'caregivers' label, but that might not be immediately apparent to everyone. Most children with severe speech and language difficulties are identified (if not diagnosed) during the preschool but there is no reference here to people such as Health Visitors, Early Years staff (or not explicitly), or even GPs. Consequently I find it hard to give a categorical answer to this question.
$R_6LIAgEx6sspizpX$	Ideally reports from family & settings but they may see different aspects of the child so concern from either may be considered
$R_e5KJQmN6txthTRX$	inclusion of parents and others HV and EP preschool teachers this isn't at all clear who is included in caregivers? What specialist are they referring to?
$R_6JOosydU46ZndMF$	I believe that some requirement for 'persistent problems' is important to include. That is, caregivers/teachers may voice concerns about speech, language etc only to find the 'problem' resolves in a few weeks. Specifying a time frame for 'persisting problems' is challenging and should vary depending on chronological age. In fact, the last part of this item 'or a lack of progress despite targeted classroom assistance' implies duration of problems.
$R_71b9fvukXBUQ5dr$	Concerns should always be investigated.
$R_cYBwzqu4ivWh9qJ$	Some pupils become less able to manage in education as the demands become more challenging e.g higher order language and proliferation of technical terms in secondary school. It is important to be able to refer pupils who may have not been picked up in early years.
$R_cIxZunCo2wnTfVj$	Additional reasons might include failing a preschool screening test, if administered as part of public health care, even in the absence of concern from caregivers.
$R_834xbT3yZzu1O7z$	Parents/teachers will have lots of information about children's language and communication skills. However to rely on this does have its constraints - we still have lots of work to do to ensure that caregivers and teachers have the information they need about typical development and atypical development.
$R_3rrKtkb2VvC3uG9$	Other professionals should be included, such as MDs, audiologists, psychologists
$R_5C49A94jWehNBB3$	But the epidemiological and longtudinal data have consistently shown that only a very small minority of cases of language impairment are ever referred. Relying on caregiver and teacher referral to start the process is problematic when it leads to large disparities in service (e.g. boys with externalizing behavior problems unintentionally given preferential access over girls with internalizing behavior problems, comorbid cases given preference over SLI, etc.). A defining feature of language impairment should never be "must annoy their teacher".
$R_5ceQk7pgvAecMAt$	I was not certain how to interpret "include" in this item. / / I agree that these could be among the reasons for referral, and that sources of information other than standardized assessment should be considered. If the meaning of this item is that these are among multiple critieria and are not necessary for referral than I would have agreed with the item. / / However, I have concerns about relying on parents/teacher identification, some of which were raised in round 1. Parents may not have enough information about typical language development and/or may miss comprehension difficulties. Also, there are equity issues. Children's whose caregivers are least able to identify language issues may be children particularly in need of support due to the overlap between language difficulties and social disadvantage, and the compounding of challenges. Further, girls are less likely to be identified for a number of services (addressing language, attention, and problems). There are a number of potential reasons. Girls are more likely than boys to socially withdraw when they are having difficulties. Further, social withdrawal in girls is more congruent with gender norms than in boys, so may not be flagged. Similar disadvantage in parental ability to monitor their child's progress and engage with the education system would apply to newcomers and families living in poverty or with mental illness. Structured identification processes (screening) may identify disadvantaged children including girls, ethnic minority, and children living in poverty/ family chaos, etc.
$R_23qAFVuJCo6YHOd$	References on correlations between educational impact/ attainment and language difficulties would be useful justification for the lack of progress. The 'lack of progress' in the statement should read 'lack of progress with aspects of learning related to language within the curriculum'. / If this statement is about referral for any communication difficulty (rather than just language), it needs to refer to issues with social interaction and friendships.

$R_8bIXFrv4VBlvVyZ$	I agree with all bar the reference to "caregivers or teachers"; if this were to become a statement used in a decision tree or other guidelines on referral the group would need to be expanded to include for example other relevant health and social care professionals who may also observe signs (e.g. health visitors/public health nurses; or early childhood educators other than teachers who work with preschoolers); alternative would be to word it "....expressed by persons including
$R_7WXquZJy8WlgXAx$	The word 'include' is important here. there may be other reaons given the hidden nature of language difficulties - these are covered in the next question(s)
$R_cCuacCYZiqQHKgl$	I think these are certainly valid standards. I do wonder whether screening at certain points in development should be reconsidered. James Law and others have examined this to some degree and found that screening can often yield too many false positives. I believe that using metrics such as sensitivity/specificity and false positive/negative are not good accuracy metrics for quantitative traits. These assume that the condition is discrete and thus hits and misses are discrete. Most screening errors for language involve children who are clustered around the cut point and thus their risk status is not all that different from those who are correctly identified. It used to be common in the U.S. for children to be screened for speech and language during kindergarten. This is not common any more. To some degree RTI now serves this purpose, but many of our late identified poor comprehenders could have been identified in kindergarten.
$R_es7hPPlfD7bdd65$	Can't imagine disagreeing with this - "includes" leaves the door open to other reasons of course.
$R_e9cPjWuFpcer4B7$	This does depend a little on what one means by specialist. If we assume that SLT means specialist this is fine. Of course in many schools SLTs are now just part of the school staff.
$R_6tiOrhFOdV4NANf$	Reasons should include but not be limited to those highlighted in this statement. It is also pertinent to include unexplain behavioural difficutlies, literacy difficulties etc. as reasons for specialist assessment (and then intervention if indicated).
$R_3sXNbQYRlZaMb3L$	This needs to be alongside more input for the early years workforce and classroom teachers to give them more understanding of typical language development trajectories/age-related expectations, and to support them to use screening tools.
$R_3DfMsLnqK54HqcZ$	Include yes. However parents and teachers may not seek help or be concerned about speech and/or language. Educators need to be supported to consider speech and language as a possible factor in a range of children who may not be making expected levels of progress and pre-school provision and Health Visitors should be supported to specifically surveil and monitor speech and language development and seek specialist advice if there are concerns. We need a joined up educational and health system within which speech and language development is monitored over development as a key developmental and educational outcome, data collected and (crucially) shared to allow individual pathways to be tracked, children at risk/vulnerable monitored and help provided as required, those who develop (e.g.) behavioural or literacy difficulties and those with recognised co-morbid diagnoses have language assessed.

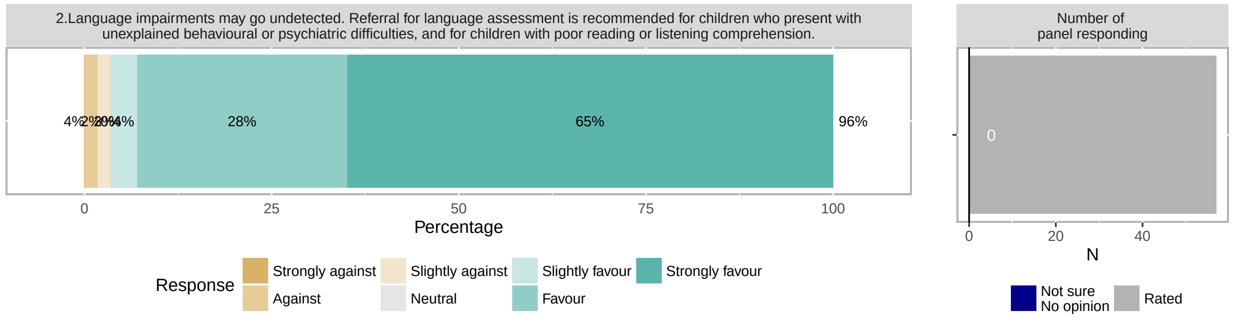

Figure 5: Percentage of panel members in each response category to statement 2. The percentages shown at each end of the scale are the cumulative percentages for the top and bottom three categories respectively.

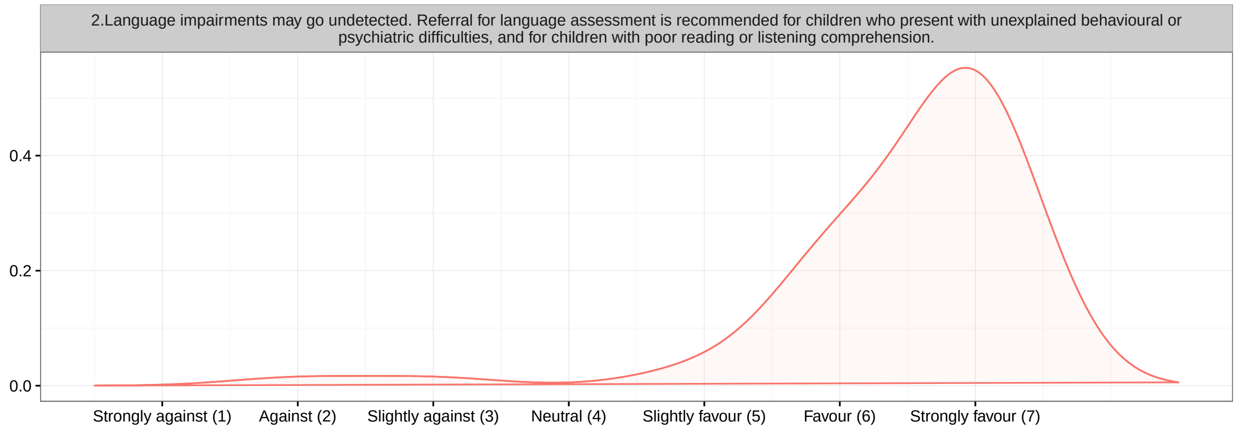

Figure 6: Distribution of responses to statement 2. The bold vertical line coloured red is Anonymous's response to this question for reference.

Table 2: Comments for each statement.

ResponseID	Q2B
$R_b a8iHG84IJ8cW7X$	As suggested these may be important avenues for investigation but care should be taken to ensure children are not uncomprehendingly subjected to several ' assessments' in very close proximity.
$R_5 uxk08XTwJpUk9D$	This may be the largest group of children with LI who have gone undetected.
$R_8 AhxnQPe8mJkUoR$	Evidence regarding relationship between language and behavioural/psychiatric difficulties evaluated in friendly manner by Beitchman and Brownlie recent book. / Language Disorders in Children and Adolescents, by Joseph H. Beitchman and Elizabeth B. / Brownlie, 2014. Series: Advances in Psychotherapy – Evidence-Based Practice /
$R_2 f9ctxaHBJuJdLD$	As a ———— I have been surprised at the associated PLI in conjunction with ADHD and conduct disorder cases; I guess what we know from Gillberg around the concept of DAMP that there is an association between ADHD dyspraxia and conduct problems with ASD and accordingly these conditions in particular need careful language assessments. If there were such a thing as primary conduct disorder I would expect high comorbidities with specific reading disorders and also ASD based language conditions. It would be useful for any tertiary CAMHS tier 4 service to have access to specialist speech therapy assessments. The CCC – 2 is enormously helpful as a screening tool for all CAMHS services but will the resources match the ensuing demand!?
$R_4 HGIGYFIvMxLWcJ$	Bu the statement is a little vague. Poor reading – should this read reading difficulties or reading accuracy, comprehension, or fluency difficulties? Listening comprehension seems to indicate/ point to language impairment.

$R_bQ13TaeUPFsxVJP$	I support this statement as the research indicates that language impairments may go undetected in these groups. However, it should be noted that two of the research studies used relatively small groups, so it would be interesting to note if this held in larger studies.
$R_bwwc7dPFEcp1azH$	Bright children in particular can be good at masking their difficulties and the associated difficulties listed above can be an indication of an underlying language difficulty- particularly shown in the case of young offenders
$R_2o7JoTNgC3lqSIR$	Yes, theoretically I would support this. Certainly CAMHS teams need to have SLTs on staff, from the start, embedded in. However, the reality on the ground in the UK is that there are very few SLTs working with the schoolage population and with specialist skills are in very short supply. So there is a risk that broadening referral criteria would overwhelm services and perhaps mean the relatively small number of children with severe, long-term SLI etc missing out on intervention they really need. Perhaps, though, we need to use to make the case for more, and more highly skilled SLTs.
$R_e5KJQmN6txthTRX$	the risk of doing this is you might put a lot of pressure on resources and without more resource services will be over stretched.
$R_2hLYvspULpn8NnL$	I would suggest beginning with SCREENING for children referred for unexplained non-language disorders. To give a full language evaluation for every child with every kind of problem invites practitioners to ignore the recommendation (as American pediatricians ignore the recommendation to screen every toddler for autism). Those who fail a well-validated screening (OK, maybe we con't have one now) would go on to a full evaluation.
$R_6JOosydU46ZndMF$	However, I disagree with the use of 'unexplained behavioral or psychiatric difficulties'. Most such problems are 'unexplained' . My strong recommendation is to refer for communication assessment any youngster who manifests behavioral or psychiatric difficulties, particularly in preschoolers and young children
$R_71b9fvukXBUQ5dr$	Likely associations - check!
$R_1TXxdyLg1UFCx4V$	It might be helpful to include children making poor or unexpected lack of progress in other subjects e.g. maths where verbal reasoning is required or history requiring narrative and writing skills. Some children manage well in the acquisition of early skills such as reading or addition etc but these subjects can reveal difficulties in older individuals.
$R_cYBwzqu4ivWh9qJ$	In a subjective reaction, yes ———— poor communication, misunderstood motives, behavioural difficulties, mental health issues (increasing) in schools. Whilst there are also societal and policy choices at work here, a language assessment would be very useful and could alter the trajectory for some young people.
$R_6RlkuyWJYcIIsmN$	This position is strongly supported by child psychiatry literature (eg work of Nancy Coehm Joe Beitchman) and also by the literature on language skills of young people in the youth justice system (most of whom were not identified with respect to language during their school years).
$R_834xbT3yZzu1O7z$	The evidence speaks for itself on this one
$R_3rrKtkb2VvC3uG9$	Also children with known high risk health conditions such as hearing loss and exposure to HIV
$R_5C49A94jWehNBB3$	However, most children with LI do not present with difficulites in these areas. The risk with this strategy is that we could unintentionally fill our finite caseloads with an overabundance of comorbid cases (that are already geting other serivces) at the expense of children with SLI who would otherwise not receive any services at all.
$R_cLU7KRGW2XvEql7$	So often the wrong intervention (or inadequate) is given because underlying language disorder has not been considered. This is also essential for children who may have seen an SLT when they were 2-3 years old and discharged with NAD and are now 8 or 9, because they may easily have a different type of language disorder, relating to later stages of language development
$R_23qAFVuJCo6YHOd$	This item is very important- for both structural language and pragmatic language. I am not sure if pragmatic language difficulties are being included in the current questions about 'language impairment'.
$R_eG1jl51DiHRqXKB$	I think that the term "unexplained behavioural or psychiatric difficulties" is vague and not useful. Even if the behavioural or psychiatric difficulties can be "explained" in some way a child may still have language difficulties. Moreover, as children move into adolescens it is especially important to examine higher order/figurative language.

$R_8bIXFrv4VBlvVyZ$	yes this an uncontroversial statement, in addition to those cited in the background document, there are several studies that would support this (e.g. Law & Stringer 2013; Hulme & Snowling 2011; Ricketts 2011)
$R_7WXquZJy8WlgXAx$	Very definitely for unexplained behavioural or psychiatric difficulties. And also for poor reading or listening comprehension on the understanding that there is not a 'one size fits all' assessment i.e. that 'assessment' can be an initial screen before a more in-depth assessment
$R_eLQkgmeJRWdZ1Vr$	Agree that langauge impairments go undetected. However I feel that we should be enskilling our partners and universal services to spot communication difficulties and base referrals on this rather than a blanket referral for all children with unexplained behavioural or psyciatric difficulties and for children with poor reading or listening comprehension.
$R_9U2zxMLVAPcvQUd$	Hollo, A., Wehby, J. H., & Oliver, R. M. (2014). Unidentified language deficits in children with emotional and behavioral disorders: A meta-analysis. Exceptional Children, 80(2), 169-186. / / i find it odd to include "listening comprehension." Behaviour problems and reading problems are issues that commonly co-occur with LI and can be the reason why LI hasn't be considered or detected. Listening comprehension, on the other hand, is one of the core features of LI, which seems to necessitate listing other core language features (e.g., poor grammar, small vocabulary, etc.). I think it distracts from this item being focused on the kinds of things that lead LI to go undetected. If it's meant to capture APD, then perhaps change "listening comprehension" to "auditory processing" or "processing sounds".
$R_cCuacCYZiqQHKgl$	Again to my point regarding screening. In our work where we do use screening as a part of ascertainment we find many children who have genuine language impairment with no reports of parental or teacher concern.
$R_3sXNbQYRlZaMb3L$	Absolutely re behavioural difficulties. Think the research into the SLCN of the prison/offending population is very important here. / / The difficulty is that poor reading comprehension or listening comprehension are themselves under-identified by school staff, so we're relying on one undetected problem as a marker for another undetected problem.
$R_3DfMsLnqK54HqcZ$	see above

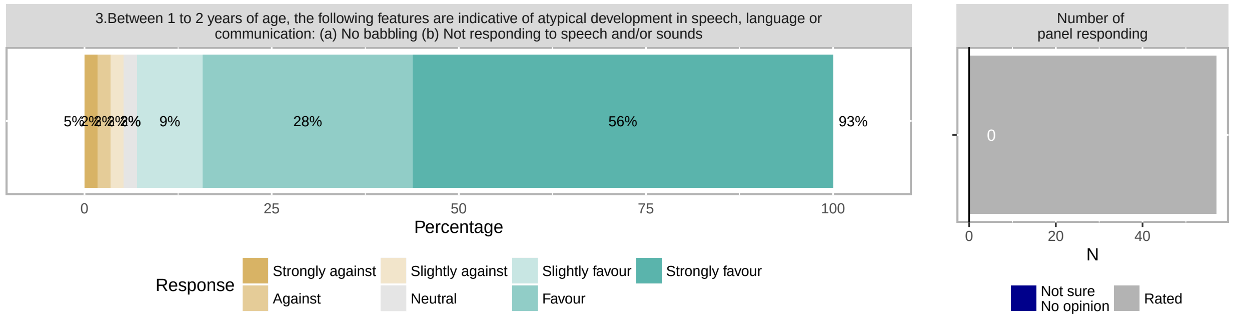

Figure 7: Percentage of panel members in each response category to statement 3. The percentages shown at each end of the scale are the cumulative percentages for the top and bottom three categories respectively.

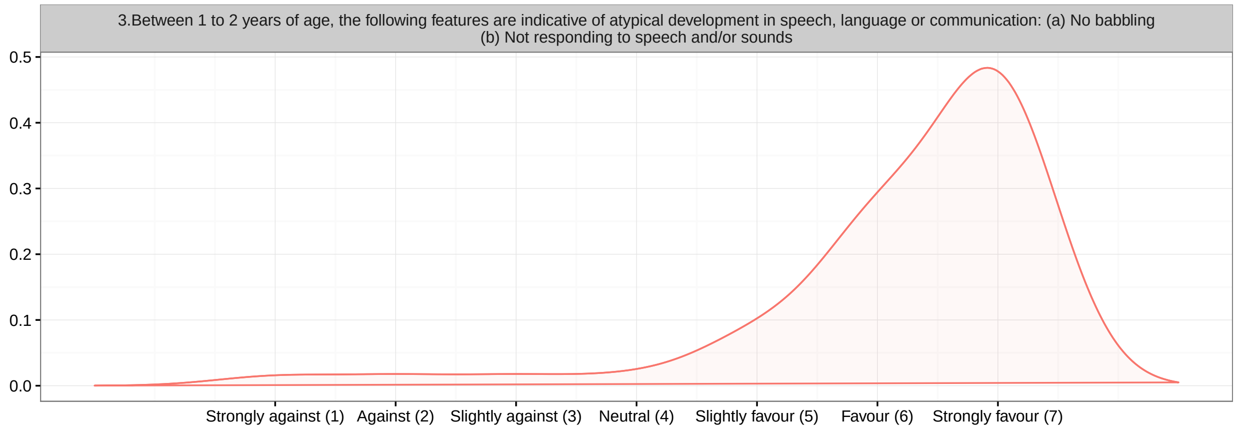

Figure 8: Distribution of responses to statement 3. The bold vertical line coloured red is Anonymous's response to this question for reference.

Table 3: Comments for each statement.

ResponseID	Q3B
$R_5uxk08XTwJpUk9D$	These are quite extreme red flags that could signal much more than LI
$R_5cd8BDkYcGfGLKl$	Yes, although the first port of call might be GP/audiologist here!
$R_8AhxnQPe8mJkUoR$	The addition of the items based on the Visser-Bochane recent article is generally welcome. / However, I have some concerns about the KEY population FOCUS of the Delphi (regardless of terminology). It seems to me that we want to improve our understanding of children with language impairments. The Visser-Bochane work seems to really focus on the spectrum of speech and communication as well as language. Thus, some of the "red flags" are in some ways quite obvious but in some developmental periods not as sensitive to language impairment (versus ASD for example) as I would like them to be. I will make suggestions throughout as I think there are quite a few identified red flags that are indeed very helpful. / For this item: / I would add "no interaction" here as a key red flag. 18 month olds who are not interacting definitely have problems. The crucial period is 1-2 years of age.I think including this item in the 2-3 year range is not as helpful a red flag as it could be earlier in development, as it is worded as "no interaction" and it would be quite unusual in my experience to have a parent or caregiver to have a walking 18 month old not interested in interaction at all. / I would also reword "not responding to speech and or/sounds", to "not responding to spoken language (speech and/or sounds)"
$R_4HGIGYFIvMxLWcJ$	Although I agree that these features are atypical, this statement needs rephrasing – this whole statement seems to refer to hearing impairment.. And if we are talking about communication in general, then it should also include - not initiating communication and so on.

$R_bwwc7dPFEcp1azH$	Other factors such as lack of eye-contact, poor joint attention and interaction, poor use of gesture, lack of pretend play (functional or symbolic) need to be added also- also by 2 you should really expect a minimum of 50 single spoken words and 2-word sentences so the two risk factors listed about really refer to younger children (0-1)
$R_2o7JoTNgC3lqSIR$	This is true. However, they do not only point towards SLI and related conditions. Often they are the first signs of ASD, developmental delay etc.
$R_6LIAgEx6sspizpX$	Would expect to see noisy, communicative toddlers even if few words are used. Would also look at progress/postive change over any given couple of months
$R_e5KJQmN6txthTRX$	We are all for red flags but most of the time you can not pick up on SLI below 2 years. There is a risk that this might mislead non specialists into believing those who don't exhibit those characteristics don't have difficulties.
$R_2hLYvspULpn8NnL$	These criteria seem too lax and will allow too many children to slip through. Very few children don't babble at all or don't respond at all. No canonical (i.e., CV) babble by 12 mo. (Oller et al., 1998) and no response to simple requests for objects or actions might be a little better.
$R_6JOosydU46ZndMF$	also, wouldn't 'limited or no engagement or interest in social interaction' be relevant here?
$R_cYBwzqu4ivWh9qJ$	However as a sole criterion for assessment/intervention we could miss some difficulties
$R_6RlkuyWJYcIIsmN$	I agree with the statement but would have concerns about these features well before 1-2 yrs of age.
$R_cIxZunCo2wnTfVj$	I agree, but think there should also be an indicator that reflects difficulties in play or interaction. If child is not engaging in reciprocal interations between the ages of 1-2, I think it's appropriate to refer - and I'd say it was indicative of some abnormaility in the development of communication.
$R_834xbT3yZzu1O7z$	While I agree that this is indicative of atypical development and I am aware that you state that children who meet these minimal levels do not necessarily have no problems, I am not clear about what the inclusion of these statements will mean - I feel we should exercise caution about setting out these as minimum requirements, given what we know about under identification of language impairment. This comment applies to all the statements below re: features of atypical development
$R_3rrKtkb2VvC3uG9$	this is good as far as it goes, but overly focused on speech. should include few words in vocabulary by 2 years of age; See Ages & Stages questionnaire and others of this sort for language-related items. this is just too speech-defined.
$R_5C49A94jWehNBB3$	A very low bar but I would agree that these represent atypical development.
$R_23qAFVuJCo6YHOd$	I am concerned that these may be indicators of ASD type social communication difficulties - where the child has difficulties with the purpose of communication. They do not include lack understanding or use of words.
$R_8bIXFrv4VBlvVyZ$	If I can safely assume as per background document that other features could also be considered (per items 1 & 2). The above list is not comprehensive and thinking of inclusion in a decision tree, a child having a very "off" day with severe otitis media might neither babble nor respond to speech/other sounds; as long as referral agents & decision trees include other features then this is fine and I concur these are atypical.
$R_7WXquZJy8WlgXAx$	They may also, of course, be indicative of other atypical developments - but certainly of speech, language or communication
$R_6mrinfsu6CeSmBn$	These features do not seem to indicate 'SLI' as we currently identify it. In my experience, children who later present with disordered language learning skills will often be babbling, vocalising and responding in some way to others' attempts to communicate with them. These features seem more indicative of other groups of children with atypical development such as those with a HI or ASD. Children with 'SLI' may be missed in early intervention if these were the criteria for referral for assessment.
$R_cCuacCYZiqQHKgl$	Certainly, absence of response to speech is worrisome as it could be a sign of hearing loss or ASD. My clinical experience has indicated that late or no babbling was often in the history of children with speech and language problems. I'm not sure how common this is in typically developing children. I suspect is uncommon.
$R_es7hPPlfD7bdd65$	potentially; but I'm not sure we know yet what proportion of LI cases initially present with this early on; lots of talk about whether not responding to speech is also a precursor to ASD, so some potential confusion there.
$R_e9cPjWuFpcer4B7$	I agree that criteria would be helpful but they need to be quite extreme to be useful. A low level of Vocabulary on its own is not enough.

$R_3DfMsLnqK54HqcZ$	I would add detail about pointing here too.

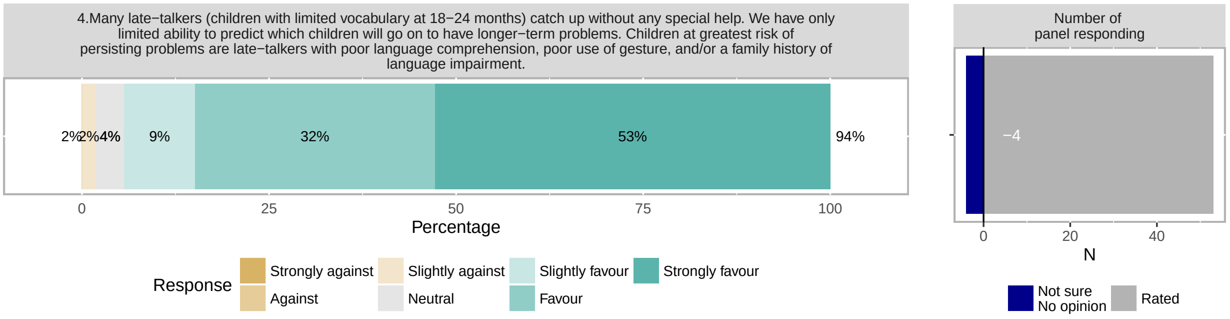

Figure 9: Percentage of panel members in each response category to statement 4. The percentages shown at each end of the scale are the cumulative percentages for the top and bottom three categories respectively.

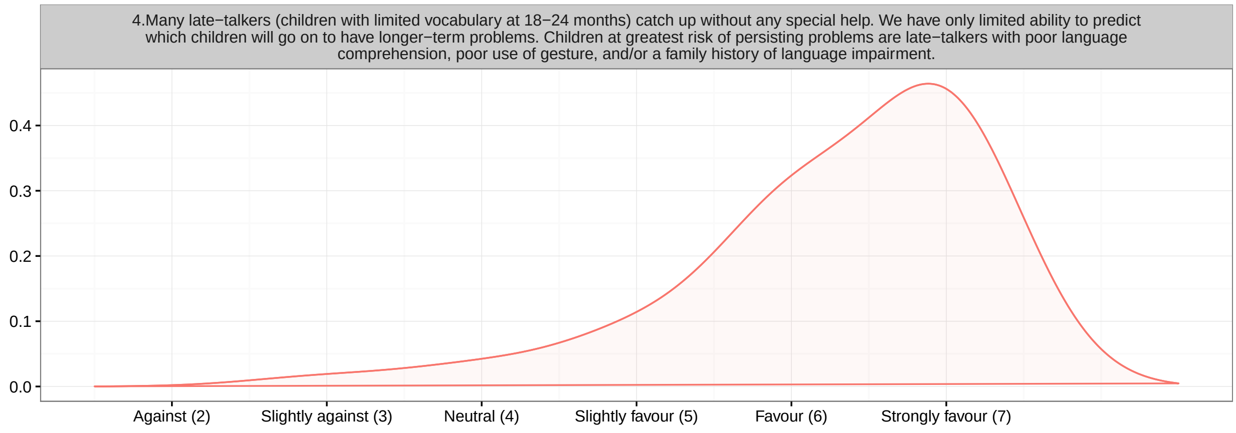

Figure 10: Distribution of responses to statement 4. The bold vertical line coloured red is Anonymous's response to this question for reference.

Table 4: Comments for each statement.

ResponseID	Q4B
$R_bDBwfKBpPTJqjjf$	Quite a few statements in here - agree with some, but not others.
$R_5uxk08XTwJpUk9D$	My own review of the evidence supports this strongly. Of course being male is an additional risk factor but given the challenges of bias in referral etc. I would not want to see this added to the list.
$R_2f9ctxaHBJuJdLD$	Does this not reflect the generally reported poor prognosis for receptive language disorders overall as well as those with ASD overlap and high genetic contribution and therefore impaired parental support for remediation in some cases?
$R_4HGIGYFIvMxLWcJ$	Agree
$R_0ofhSCmeppIQ8kt$	Also consider: / Dollaghan, C. (2013). Late Talker as a clinical category. In L. A. Rescorla and P. S. Dale (eds.), Late Talkers: Language development, intervention, and outcomes, pp. 91 – 112. Baltimore: Brookes.
$R_bQ13TaeUPFsxVJP$	This statement is difficult to reconcile with statement 3 & 5. A recent editorial (Norbury, 2015) argues that early intervention is not always best. An important question for research is when language measures become reliable, and whether in certain at – risk groups, these measures become stable earlier in time? And in the meantime, is a 'watchful waiting' approach appropriate?
$R_6Dvhy7Alhw5wqIR$	my response to thsi rather depends on the severity of the delay at 1 – 2 eg Q3 indicates a severe problem and likely to be more serious but mild delays at 18 months can catch up
$R_bwwc7dPFEcp1azH$	Nonetheless, for children who do not have these risk factors but have limited vocabulary, some general help and watchful waiting should be offered

$R_2o7JoTNgC3lqSIR$	Problems at birth or a preterm delivery also seem, anecdotally, to be common factors. If this has not been investigated, perhaps it is worth looking into.
$R_6LIAgEx6sspizpX$	These 'red flags' would concern me; also children who are not making progress
$R_e5KJQmN6txthTRX$	this is true but the priority is to identify these children.
$R_6JOosydU46ZndMF$	Also, what about family psychiatric history? Maternal depression, bipolar disorder, substance abuse, violent abusive behavior etc are likely to have a negative impact on children's communication abilities
$R_1TXxdyLg1UFCx4V$	Clinicians need to feel confident in explaining a decision, for example to 'watch and wait' to parents who may be concerned about a very young child with language or speech delay. Few currently utilize research to support this approach but it is common in other areas of NHS practice
$R_cYBwzqu4ivWh9qJ$	I would also like to see included a family history of other specific and non-specific learning difficulties. SpLD/dyslexia springs to mind as an obvious consideration given the phonological loop connection.
$R_3rrKtkb2VvC3uG9$	So what are we asked to focus on, the way we can define risk and use it as an indicator or the lack of prediction? Once we have these 3 indicators we have a highly respectable index of risk. I don't know why the ASD scientists are pleased about the new finding that 20% of young sibs show signs of ASD whereas in our field the same proportion is regarded as dubious indication of risk..
$R_5C49A94jWehNBB3$	In this case, another "red-flag" would be female sex. The male/female ratio in what we can refer to as the "late bloomers" group decidedly favors males whereas the sex ratio for unresolved LI cases has been more balanced.
$R_cLU7KRGW2XvEql7$	So this is why it is important to have monitoring in those early years, proper information for parents and well trained/informed health visitors
$R_23qAFVuJCo6YHOd$	I have rated neutral- as I am concerned about the 'poor use of gesture'. Does this indicate language difficulties or lack of communicative intent and therefore a wider group of children?
$R_8bIXFrv4VBlvVyZ$	agree though we might remain open to the possibility that with further data from longitudinal studies we may add to the list (thinking of Zubrick et al's 2007 finding that neurobiological factors, e.g. lbw, were also predictive, which wasn't found in Reilly et al 2009; there are large studies pointing up risks arising from prematurity and low birth weight for development generally)
$R_7WXquZJy8WlgXAx$	I have changed my response to this slightly this time as I was so out of line with everyone else! I know that many late talkers do catch up without specilaist help - but this presupposes good, universal practice is in place in homes and in early years settings to facilitate this early development - i.e. not specialist help, but good practice. I think it is a risky statement to run with outside of this exercise. it risks the general opinion being that late talking is not a potential problem, and that there shouldn't be on-going monitoring. I would be happier if there was an addendum to this that indicated the need for on-going, regular monitoring of development.
$R_6mrinfsu6CeSmBn$	I agree that a family history of language impairment and demonstration of poor comprehension alongside poor expression would increase my level of concern about a specific child, however poor use of gesture is not in my experience indicative of later diagnosis of 'SLI'. In my experience, many children who later receive a diagnosis of disordered language learning skills can have good non-verbal communication skills including use of gesture. Hence I am not sure how to respond to this statement... These descroptors would not necessarily pick up children with primary speech sounds disorders.
$R_9U2zxMLVAPcvQUd$	Paul, R., & Roth, F. P. (2011). Characterizing and predicting outcomes of communication delays in infants and toddlers: Implications for clinical practice. Language, Speech, and Hearing Services in Schools, 42(3), 331-340.
$R_cCuacCYZiqQHKgl$	This is an accurate summary of the late-talker data. I am increasingly convinced that we should be able to predict much better than we can and that much of our problems are due to measurement problems. Recently Bornstein and Putnick (2012) reported in Developmental Psychology that individual differences in language between 2 years and 4 years was very stable (r=.84). This study used better methods of measurement and analysis and used continuous measures throughout. There is always a danger in cutting continuous scores as this can lead to regression to the mean. Other than measurement issues, it could be argued that Late-Talkers are different from other language learners, but then how do we account for the fact that they move into the average to low average range?
$R_es7hPPlfD7bdd65$	I just don't see kids this young in the research domain. So I'll let the real experts chime in on this.

$R_e9cPjWuFpcer4B7$	As above. Vocabulary is not enough. These other criteria are helpful although from what I recall of Whitehurst's work the gesture point is wrong. Children with persistent gesture rather than expressive language ARE at risk. So this specific behaviour is time sensitive.
$R_6tiOrhFOdV4NANf$	Intent to communicate is also important at this age
$R_1FT913eWSaeKlhP$	My response to the first and second sentence is "strongly favour". My response to the third statement is "Strongly against". The 3 risk factors listed here may be amongst the best of weak predictors but they are still weak predictors. This statement ignores longitudinal research that shows that risk factors change over time.
$R_3DfMsLnqK54HqcZ$	Agree that these risks are important however the use of late talker as a risk factor is problematic as in population samples approx 50% of 4 year olds with low language abilities WERE NOT LATE TALKERS. (Reilly S, McKean C, Levickis P. Late talking: can it predict later language difficulties?. Centre for Research Excellence in Child Language, 2014. Research Snapshot 2).It also ignores the risks associated with parental interactive behaviours and family 'resources' which are also vitally important. I would add in social risks and remove/modify 'late talker' to a more dimensional rather than categorical term (low language - and define more widely than < 10th centile). Also Note that late talker status is defined using CDI type instruments which peak in their reliability at 14 months - thereafter they are pretty unreliable(Robinson, B. F., & Mervis, C. B. (1999). Comparing productive vocabulary measures from the CDI and a systematic diary study. Journal of Child Language, 26, 177-185) .and importantly are skewed with respect to social disadvantage such that socially disadvantaged parent over-estimate abilities. (Law & Roy (2000)nParental report of infant language skills: a review of development and application of the Communicative Development Inventories. Child Adolescent Mental Health. 11, pp. 198-206).

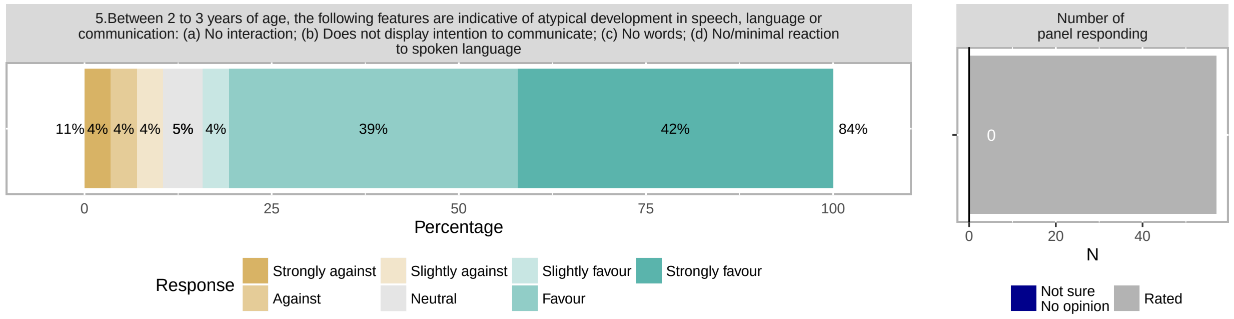

Figure 11: Percentage of panel members in each response category to statement 5. The percentages shown at each end of the scale are the cumulative percentages for the top and bottom three categories respectively.

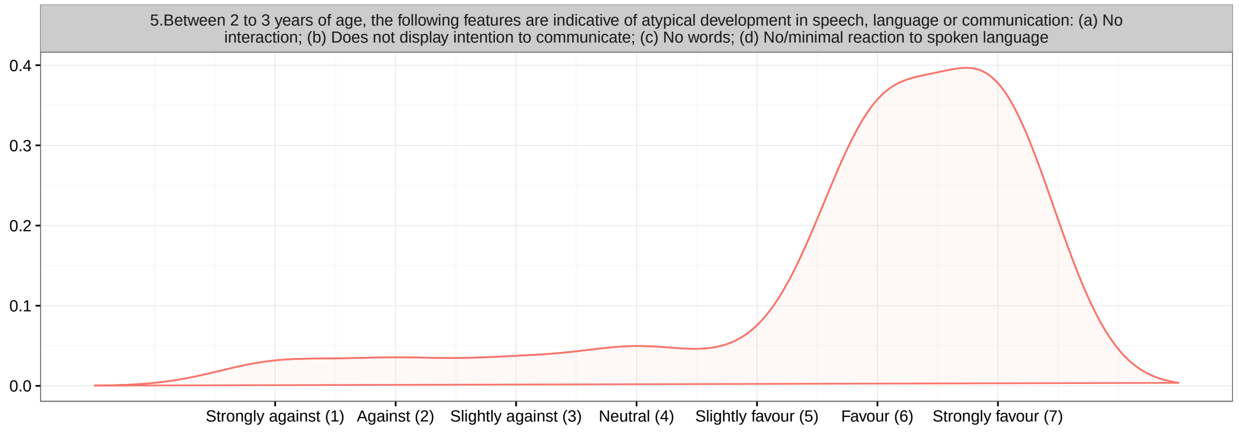

Figure 12: Distribution of responses to statement 5. The bold vertical line coloured red is Anonymous's response to this question for reference.

Table 5: Comments for each statement.

ResponseID	Q5B
$R_bDBwfKBpPTJqjjf$	I'm assuming these are 'or' lists, rather than 'and' lists? /
$R_5uxk08XTwJpUk9D$	Yes - this is true but it is far more likely that these features are associated with a diagnosis of ASD rather than LI and therefore the referrals need to be more directed toward clinicians who can evaluate for more than language/communication problems
$R_5cd8BDkYcGfGLKl$	I would say 'minimal interaction' - most children are likely to interact on some level.

$R_{8}AhxnQPe8mJkUoR$	I have reservations about this item. Yes, these are “red flags” but do they raise alarm bells for language impairment or ASD/learning disabilities of quite severe kind? / In a way, my view is that any red flags at earlier developmental periods that apply to children who are older, then are still red flags. So a kid who comes with no interaction at 2 and a half is of course of concern because we know that no interaction is a red flag for 1-2 years already. Hope this comment is clear. There is some repetition of red flags as the items stand and I would like to see more specificity of red flags which are developmentally sensitive. / My suggestions are as follows: / Drop “no interaction”...I have suggested to move it to the earlier developmental period of 1-2 years. / I would also drop “no/minimal reaction to spoken language”...again it appears in the earlier developmental period (1-2 years), slightly differently worded, but it is far too extreme for this developmental period. / The item “no words” should stay. / I am ambivalent about “does not display intention to communicate” simply because it again brings quite an extreme picture for me of a two and half year old not displaying intention to communicate in any way (gesturally, nonverbally, physically pulling you to where they need something, pointing) I would say this needs nuancing. Perhaps, something like “does not point and does not often display intention to communicate”. / Reference re: pointing / Behne, T., Liszkowski, U., Carpenter, M., & Tomasello, M. (2012). Twelve month olds’ comprehension and production of pointing. British Journal of Developmental Psychology, 30(3), 359-375. / / I would also add something like “little gain in spoken language between 2-3 years” or in 6 month period. The evidence from longitudinal studies including those of Reilly and colleagues in the preschool period suggest that rate of change is important as key steps (for example, okay at words but then problems with word combination, which appropriately is flagged by the item 6 below, when the focus is between 3-4 years of age.
$R_{2}f9ctxaHBJuJdLD$	Suggests hearing test is mandatory.
$R_{4}HGIGYFIvMxLWcJ$	As per statement 3, this needs rephrasing – what about the child who shows delayed or incorrect reactions to spoken language – is that not atypical?
$R_{6}Dvhy7Alhw5wqIR$	again, these are extreme examples
$R_{b}wwc7dPFEcp1azH$	Again by 3 we would expect 2-3 word sentences and a minimum of 200 spoken words (see studies based on CDI data). The previous criteria of lack of gestures, family history and poor comprehension should continue to apply. At these young ages (0-3)we should also ideally have different normative expectations of boys and girls with early impairments so that girls do not slip through the net and boys are not over-diagnosed
$R_{2}o7JoTNgC3lqSIR$	These factors certainly indicate probable communication difficulties. However, most of them seem more likely to point towards ASD or at any rate Social Communication Disorders/Pragmatic Language Impairment rather than SLI/Speech sound disorders as we normally understand them. Some children with SLI will have no words at 2 years but quite a lot of them have a few single words and often, in our experience, are wanting to communicate and try to talk or gesture, but can’t quite get it out or not clearly enough.
$R_{6}LIAgEx6sspizpX$	Would be thinking about ASD
$R_{e}5KJQmN6txthTRX$	this seems to relate to ASD rather than SLI as we understand them.
$R_{2}hLYvspULpn8NnL$	Again, these seem too lax. Few children–even those with autism–show NO interaction or ANY intention to communicate (those with ASD make requests, although they use unusual means). I would say no spoken words, communication limited to concrete requests, and inability to follow simple requests for objects or actions.
$R_{6}JOosydU46ZndMF$	what about youngsters with severe developmental apraxia who show intent to and attempts to communicate, respond to spoken language & appear to understand spoken language, but produce no intelligible words whatsoever - neither caregiver nor close relative or preschool teacher can understand youngster’s communicative attempts? I guess the key issue is how many of these features are required to be manifest to trigger a referral. In the case of severe developmental apraxia, another sign at this age concerns the poor muscle tone around mouth tongue etc, so child is unable to lick lips clean, close lips around spoon or fork etc
$R_{3}pDedyU4fM1kOXj$	..... What do you mean by no interaction?
$R_{c}YBwzqu4ivWh9qJ$	This shows a severe end of the spectrum but does not include all young people who will go on to have speech and language difficulties
$R_{6}RlkuyWJYcIIsmN$	I agree with the statement but would have concerns about these features well before 2-3 yrs of age.
$R_{c}IxZunCo2wnTfVj$	I’d move these indicators into the 1-2 year range, rather than 2-3.

$R_8 34xbT3yZzu1O7z$	see above
$R_3 rrKtkb2VvC3uG9$	This is too conservative; children with this profile at 3 would be at the very low end. We can add language indicators such as word combinations that would be sensitive to language impairments in children without speech impairments.
$R_5 C49A94jWehNBB3$	A very low bar but I would agree that these represent atypical development.
$R_c LU7KRGW2XvEql7$	This is a very limited list indeed. There is a danger that these become the only criteria for identifying 2-3 year olds at risk for speech & language impairment and of course we would miss a lot of children who actually do need support
$R_2 3qAFVuJCo6YHOd$	I am concerned that these may be indicators of ASD type social communication difficulties - where the child has difficulties with the purpose of communication, rather than language difficulties.
$R_e G1jl51DiHRqXKB$	It would also be important to look at psychological factors such as attachment security.
$R_8 bIXFrv4VBlvVyZ$	agree but same comment as for number 3 above.
$R_7 WXquZJy8WlgXAx$	Similar proviso to question 3. these may also be indicators of other difficulties, but certainly speech, language and communication - i.e. not specificity
$R_6 mrinfsu6CeSmBn$	As with point 3 above, these features do not seem to indicate 'SLI' as we currently identify it. In my experience, children who later present with disordered language learning skills demonstarte desire to communciate with others, and will often be attempting to interact with others, even if their ability to use verbal communciation to do this may be very limited. They are unlikely to have 'no' words, although this is possible, but they would usually repsond to attempts to communciate with them. Again, these features seem more indicative of other groups of children with atypical development such as those with ASD.
$R_c CuacCYZiqQHKgl$	Little doubt here.
$R_e s7hPPlfD7bdd65$	I tend to think this is more closely aligned with ASD or pragmatic disorder than a LI as I typically envision it.
$R_e 9cPjWuFpcer4B7$	Although the reality is that strict interpretation of these criteria would mean a minutely small number of children being identified would be very small. Even the most severely autistic children rarely have NO intentional communication.
$R_3 DfMsLnqK54HqcZ$	Yes these factors identify children with very very severe problems with language and communication development - between 2 and 3 years these would identify children with severe disabilities usually associated with other diagnoses (ASD; SLD; Profound HL). These children absolutely should be seen for specialist assessment. However they are unhelpful for the broad range of abilities/risk factors which are present in 2 year olds but who go on to have language difficulties. Should be reworded as "children should be seen for specialist assessment if".rather than "features indicative of atypical development". These problems only relate to children with very severe problems - children can by atypical with much milder levels of delay. This is where considering only specialist assessment or not makes things difficult. I think we could talk about children at risk between 2 and 3 years and give some more detailed indicators and we could provide enhanced pre-school educational provision/parent programmes for these "at risk" groups in this age range without necessarily having specialist assessment by an SLT. Given our current predictive abilities this is a very problematic age range if our only model is one of specialist services. Preventative interventions to children at risk would seem to be the answer given our current knowledge. Please also see Reilly S, McKean C, Morgan A, Wake M. Clinical Review: Identifying and managing common childhood language and speech impairments. British Medical Journal 2015, 350, h2318.

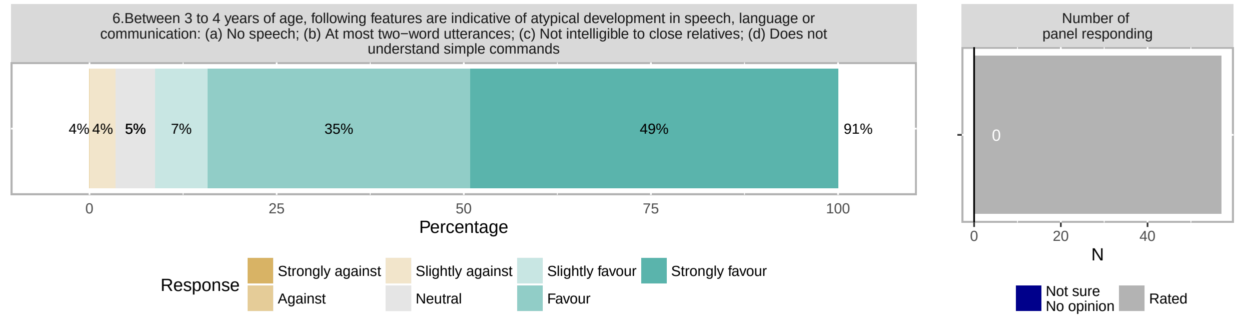

Figure 13: Percentage of panel members in each response category to statement 6. The percentages shown at each end of the scale are the cumulative percentages for the top and bottom three categories respectively.

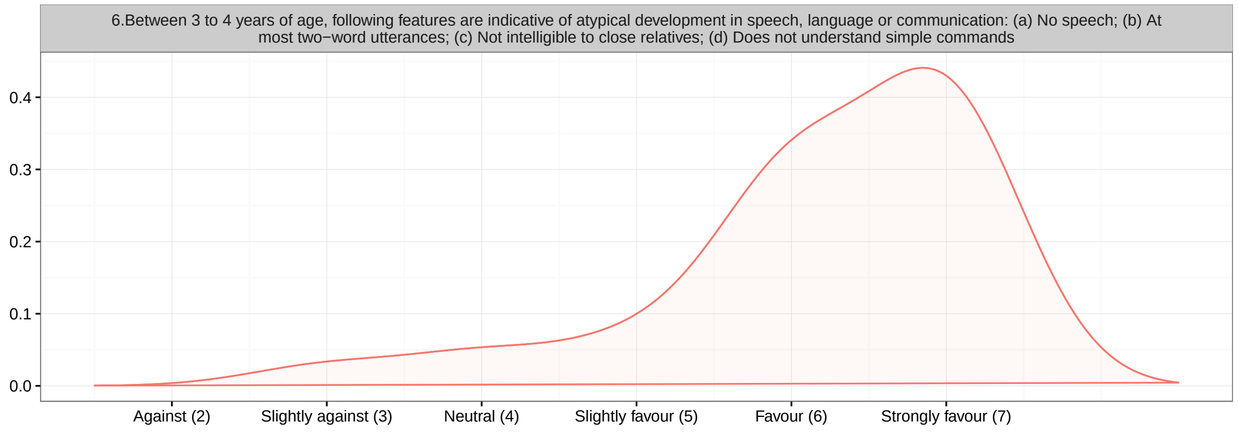

Figure 14: Distribution of responses to statement 6. The bold vertical line coloured red is Anonymous's response to this question for reference.

Table 6: Comments for each statement.

ResponseID	Q6B
$R_5uxk08XTwJpUk9D$	Again, these are good indicators but quite strong red flags for a problems that may go well beyond LI
$R_8AhxnQPe8mJkUoR$	Again, the "no speech" (a) by 3-4 years is far too obvious. If someone has not referred a child who is nonverbal even at the beginning of this period (3 years), then I am not sure what we are trying to achieve. / I also want to know: what is the difference between "no speech" in this item (item 6) and "no words" in item 5 (between 2-3 years of age? Consistency of descriptors/red flags is crucial in what we need to achieve I believe, and once statements are agreed on it is very important that we re-visit this. / Once again, if the red flag appears in an earlier stage of development, then if observed in an older child it still is a red flag. /
$R_4HGIGYFIvMxLWcJ$	what about the child who demonstrates atypical phonological speech patterns and is intelligible to close relatives. I would call this atypical for a child between 3 and 4.
$R_6Dvhy7Alhw5wqIR$	again extreme – cannot imagine anyone disagreeing
$R_bwwc7dPFEcp1azH$	These factors seem to apply to children at the more severe end of the spectrum. By 4 in TD, we expect children to be intelligible to those outside of the family too so if not that would warrant a referral. In addition, 3-4 year olds should be using aspects of morphosyntax as is relevant to the language(s) they are speaking so even difficulties in this area in a child using 4-5 word sentences could be cause for concern.
$R_2o7JoTNgC3lqSIR$	I would agree with this. Having more language than this does not, however, mean that the child does not have a problem. Some children with SLI do manage to produce utterances of more than 2 words at this stage.

$R_e5KJQmN6txthTRX$	I am not sure what this list is trying to achieve. Triggers are at a high level. There will be children with language disorders that do not meet this threshold and could therefore be missed. Advice along these lines could be misleading.
$R_2hLYvspULpn8NnL$	No need to say BOTH 'no speech' and 'at most two-word utterances.' Second is sufficient.
$R_6JOosydU46ZndMF$	here i would add not intelligible to caregiver as well - caregiver cannot understand most of or none of what child 'says'
$R_cYBwzqu4ivWh9qJ$	As before I agree these features are indicative but they are too narrow to include all children with speech and language difficulties
$R_6RlkuyWJYcIIsmN$	I agree with the statement but would have concerns about these features well before 3-4 yrs of age.
$R_cIxZunCo2wnTfVj$	I'd move the age limits down here, as well. Consider atypical as 2-3 years.
$R_834xbT3yZzu1O7z$	see above
$R_3rrKtkb2VvC3uG9$	again, this is far too conservative and would pick up only the most limited children.
$R_5C49A94jWehNBB3$	A very low bar but I would agree that these represent atypical development.
$R_cLU7KRGW2XvEql7$	as above, This is a very limited list indeed. There is a danger that these become the only criteria for identifying 3-4 year olds at risk for speech & language impairment and of course we would miss a lot of children who actually do need support
$R_23qAFVuJCo6YHOd$	These and following questions seem to refer to all types of communication rather than just language/ pragmatic language. If the statements are meant to refer to language/pragmatic language only, I am unsure about a): 'No speech' as this might indicate lack of social intent. Also c) will depend on whether we are including phonological difficulties. / I wonder if a potential indicator is frustration in relation to not understanding or being able to convey needs. I appreciate there may not be research evidence, but perhaps an area for future research.
$R_eG1jl51DiHRqXKB$	Ruliing out hearing problem of course
$R_8bIXFrv4VBlvVyZ$	this one may need a statement added; at this age range would consider adding inconsistent or abnormal interaction (as indicated for 4 to 5 year age group); harder to be definitive about before 3, but could be more easily observed after 3-particularly since children are more likely now to be in organised child care and interacting with other children.
$R_7WXquZJy8WlgXAx$	as above - yes, not not specificity
$R_6mrinfsu6CeSmBn$	I agree these features woudl be seen in children with disordered speech or language difficulties, although in my opinion they in no way indicate a primary language learning difficulty, as they would also be seen in children with a wide range of other diagnoses and conditions which affect their development more generally. There would be no reason to assume children with language learning difficulties would necessarily have difficulties with speech sounds production (although they can).
$R_9U2zxMLVAPcvQUd$	Depending on the audience, "intelligible" could be jargon. Another option would be "speech not understandable to . . . "
$R_cCuacCYZiqQHKgl$	Again, these are pretty good "red flags."
$R_e9cPjWuFpcer4B7$	Ok but these would again be quite extreme. A mother is unlikely to say that a child is completely unintelligible.
$R_3DfMsLnqK54HqcZ$	Should be reworded as "children should be seen for specialist assessment if".rather than "features indicative of atypical development". These problems only relate to children with very severe problems - children can by atypical with much milder levels of delay. These descriptors are slightly more helpful than the "red flags" for 2 to 3 years but do need more specification for non-specialists to make sensible decisions about referral (e.g. what is a simple command). (Again see Reilly S, McKean C, Morgan A, Wake M. Clinical Review: Identifying and managing common childhood language and speech impairments. British Medical Journal 2015, 350, h2318.) /

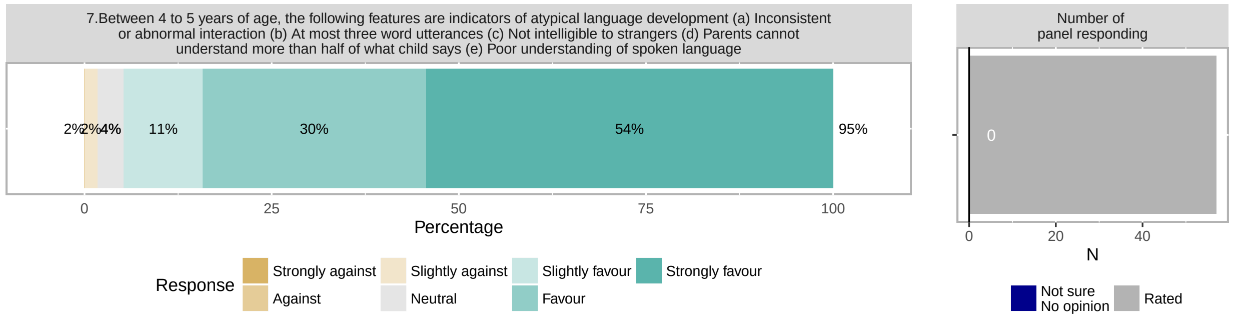

Figure 15: Percentage of panel members in each response category to statement 7. The percentages shown at each end of the scale are the cumulative percentages for the top and bottom three categories respectively.

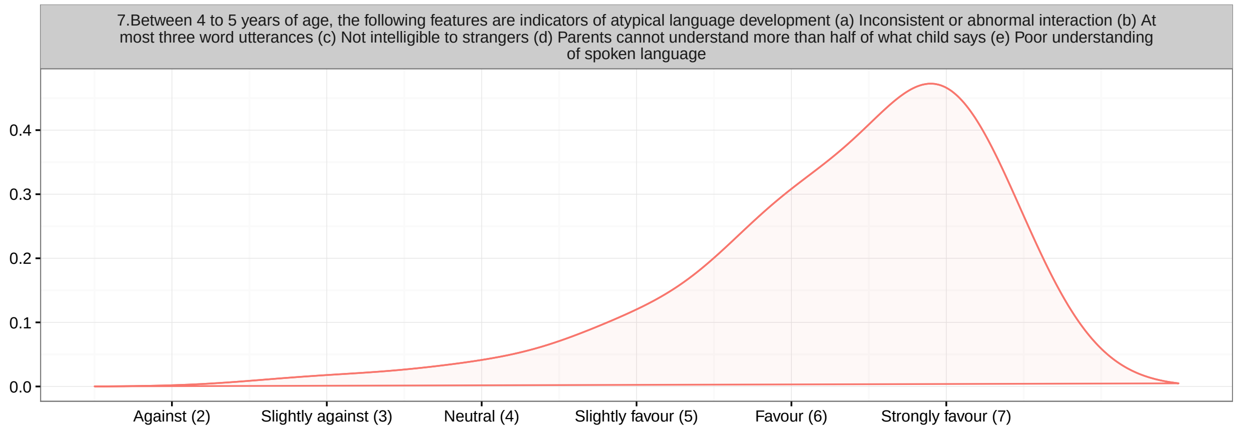

Figure 16: Distribution of responses to statement 7. The bold vertical line coloured red is Anonymous's response to this question for reference.

Table 7: Comments for each statement.

ResponseID	Q7B
$R_5uxk08XTwJpUk9D$	Same comments as before
$R_4HGIGYFIvMxLWcJ$	Similar comment to the statements above. it seems to exclude many children who would, in clinical practice, be considered to be developing in an atypical manner – in this instance a child with atypical phonological speech errors.
$R_6Dvhy7Alhw5wqIR$	same comment
$R_bwwc7dPFEcp1azH$	See above- these features again seem to apply to children with severe speech and language difficulties, and those with less severe impairments, including difficulty with complex sentences, narrative, phonological awareness and reading, or mild speech difficulties such as with multisyllabic words or the 'late 8' developing phonemes could also be candidates for intervention by 5.
$R_2o7JoTNgC3lqSIR$	Yes, but as with the previous questions, in reality some children with very real difficulties might not meet these very tight criteria.
$R_e5KJQmN6txthTRX$	Again the threshold is rather high.
$R_6JOosydU46ZndMF$	again need to clarify whether any single feature suffices to trigger referral...the severity level & risk associated with each feature seem to differ widely. Wht do you refer to parents rather than caregivers here?
$R_cYBwzqu4ivWh9qJ$	As before I agree these features are indicative but they are too narrow to catch all children with speech and language difficulties
$R_6RlkuyWJYcIIsmN$	I would include lack of narrative skills here too (and as per above, these features would be of concern well before 4-5 yrs)

$R_8 34xbT3yZzu1O7z$	see above
$R_3 rrKtkb2VvC3uG9$	This is too conservative and overlooks a large literature documenting the linguistic indicators of language significantly below age expectations. I don't know what the source is for 3 word utterances for this age range. See Rice et al 2011 for age norms for MLU showing means for language impaired children of 3.3-3.9 in this age range and typically developing means of 4.1-4.5. too much emphasis on speech
$R_5 C49A94jWehNBB3$	A very low bar but I would agree that these represent atypical development.
$R_c LU7KRGW2XvEql7$	as above, This is a very limited list indeed. There is a danger that these become the only criteria for identifying 4-5 year olds at risk for speech & language impairment and of course we would miss a lot of children who actually do need support. I think there is also an issue here about only identifying children with very severe difficulties (e.g. those who go to specialist schools and require a high level of specialist support to access the curriculum) , not the full range of children who have language impairment requiring specialist support.
$R_2 3qAFVuJCo6YHOd$	If this statement is meant to be focusing on language - again, I am not sure about a)- whether this is picking up severe difficulties with social communication, b) and c) if we are including phonological difficulties. / In addition, all expressive difficulties may not be covered under b)
$R_8 bIXFrv4VBlvVyZ$	yes this is uncontroversial; what will need to be defined are the exemplars for each item included in a decision tree for referral agents (e.g. examples of spoken language not understood) and the tools used by SLTs carrying out assessments-thinking of poor sensitivity and specificity of standardised language assessment batteries (Eadie et al 2014 for example) and the over/under-identification that can result (per comments by Roy & Chiat on the Nuffield VEPS study results where they propose that some comprehension subtests favour children from more advantaged backgrounds who have had access to consistent early education opportunities)
$R_7 WXquZJy8WlgXAx$	as above - yes, but no specificity. some indication of the amount of spoken language not understood - based on number of instructions or information carrying words
$R_6 mrinfsu6CeSmBn$	Again, as above, children demonstrating these difficulties clearly have impairments in their development of communcation skills, but applying these descriptors would result in many children with ongoing difficulties learning language not being referred for assessment or picked up.
$R_9 U2zxMLVAPcvQUd$	Depending on the audience, "intelligible" could be jargon. Another option would be "speech not understandable to . . . ", which is more parallel to the wording in the (d) item about parents.
$R_c CuacCYZiqQHKgl$	Item "a" may be hard to define, but noticeable abnormal or ineffective social communication does belong.
$R_e 9cPjWuFpcer4B7$	The two/three word criteria is not very helpful because it depends what sort of words were being combined and whether the words were being used spontaneously or learned/echoed. How would people judge 'more than half'?
$R_3 DfMsLnqK54HqcZ$	These are slightly more helpful than the "red flags" for 2 to 3 years but does need more specification for non-specialists. (Again see Reilly S, McKean C, Morgan A, Wake M. Clinical Review: Identifying and managing common childhood language and speech impairments. British Medical Journal 2015, 350, h2318.)

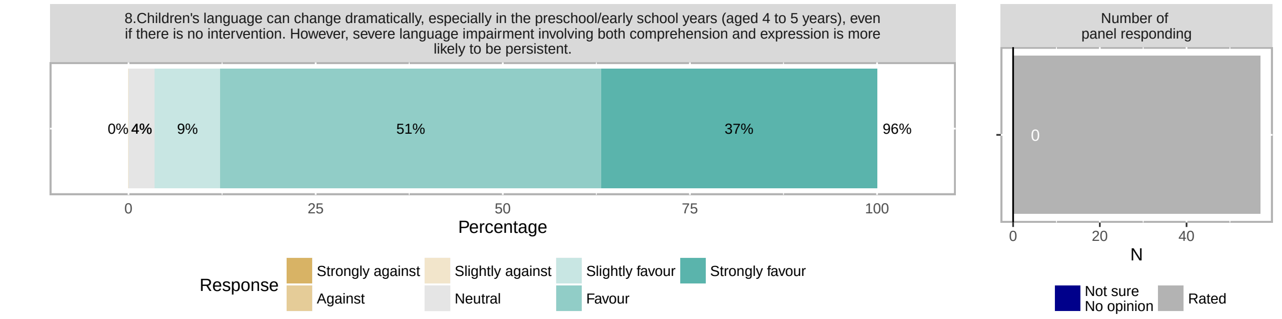

Figure 17: Percentage of panel members in each response category to statement 8. The percentages shown at each end of the scale are the cumulative percentages for the top and bottom three categories respectively.

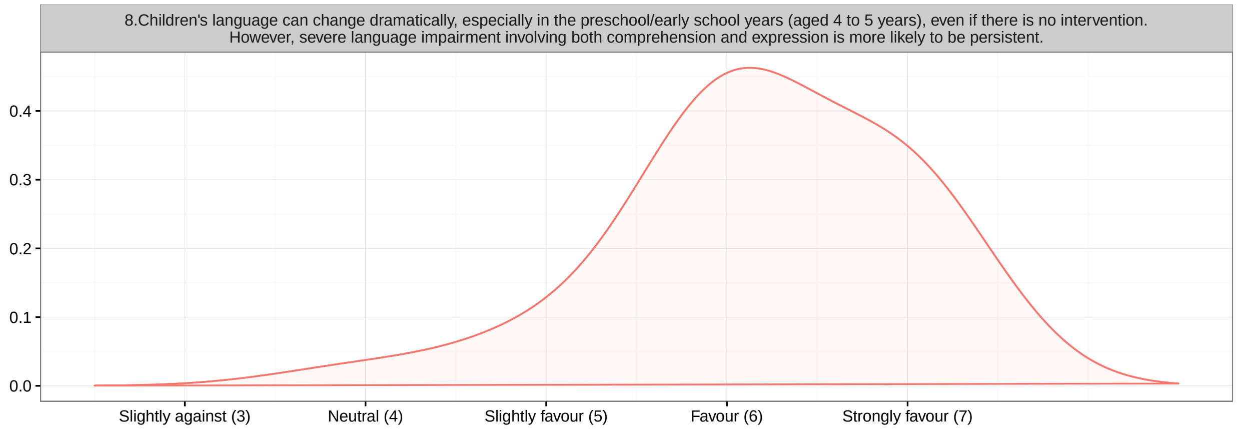

Figure 18: Distribution of responses to statement 8. The bold vertical line coloured red is Anonymous's response to this question for reference.

Table 8: Comments for each statement.

ResponseID	Q8B
$R_4HGIGYFIvMxLWcJ$	Although I agree with this statement, the implication that a staged approach fwould be optimal for children in the early school years would be country specific.
$R_0ofhSCmeppIQ8kt$	Of course, if there is a severe impairment, it's harder to move 'enough' so as to not appear impaired even if improvement does occur.
$R_bwwc7dPFEcp1azH$	Having said that, a 4-year old who cannot communicate well is open to isolation at school and at risk for academic failure. Providing no intervention to such as child between 4-5 could therefore have detrimental long term effects.
$R_2o7JoTNgC3lqSIR$	Yes, in general terms this is probably true. But there are many individual cases where this has not applied. So it is important in practice to treat each child as an individual.
$R_e5KJQmN6txthTRX$	Response to intervention can indicate the need for specialist intervention and on going assessment. This requires a quick response.
$R_6JOosydU46ZndMF$	so is the requirement for evidence of BOTH comprehension and expressive problems ? If so, I have some concerns since some children manifest major problems with expressive language and seemingly mild comprehension problems (eg with developmental apraxia
$R_3rrKtkb2VvC3uG9$	The consistency of the receptive/expressive distinction is not robust across ages. some areas of language competencies are not equally addressable/measureable in comprehension and expression tasks. Comprehension is notoriously vulnerable to confounding in naive judgements. Again, this seems a step background, to overly simplistic guidelines.

$R_cLU7KRGW2XvEql7$	Close monitoring is therefore very important. t is very important that SLTs take on a public health role with the preschool population to support non-specialist staff in making decisions about the at risk children, as it is not a straightforward or formulaic task
$R_23qAFVuJCo6YHOd$	Might this be clearer if worded 'severe difficulties with both comprehension and expression identified at age 4-5 yrs are not likely to resolve spontaneously' ?
$R_8bIXFrv4VBlvVyZ$	This is a point where the "cut-off" may be relevant-how do you define severe? thinking of children identified in the epi-sli study (Tomblin et al) with scores of -1.25 below who at follow up at 16 yrs were still behind peers (Tomblin, Nippold et al)
$R_7WXquZJy8WlgXAx$	and therefore there is a need for on-going monitoring by practitioners who are skilled with knowledge of early development, and in idenitfying early langauge difficulties
$R_9U2zxMLVAPcvQUd$	I would remove the specific bracketed mention of 4 – 5 years. / / In reviewing the rationale for this item provided in the background report, I don't find that this item leads to the implication about a staged approach. Instead, the statement seems an expanded supplement to item #4 re outcomes
$R_cCuacCYZiqQHKgl$	By 5 years and certainly by 6 our data show very high levels of stability.
$R_e9cPjWuFpcer4B7$	This is often true but regression to the mean is also likely to mean that these children will change most especially if they are more disadvantaged (see Jerrim and Vignoles).

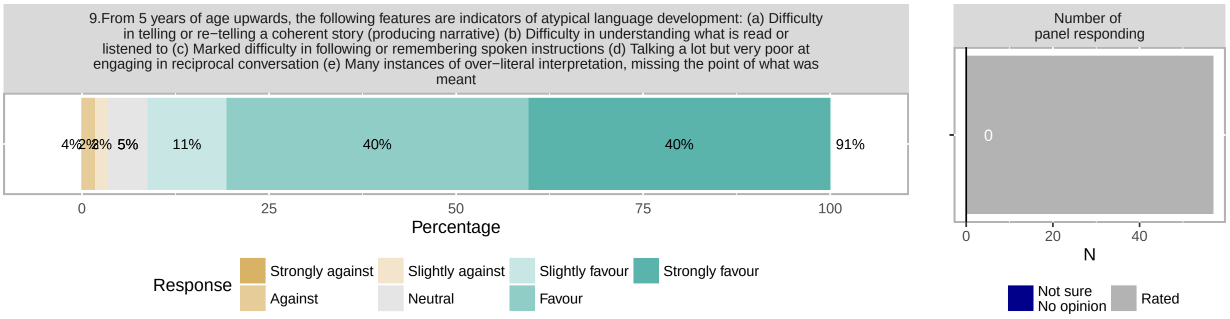

Figure 19: Percentage of panel members in each response category to statement 9. The percentages shown at each end of the scale are the cumulative percentages for the top and bottom three categories respectively.

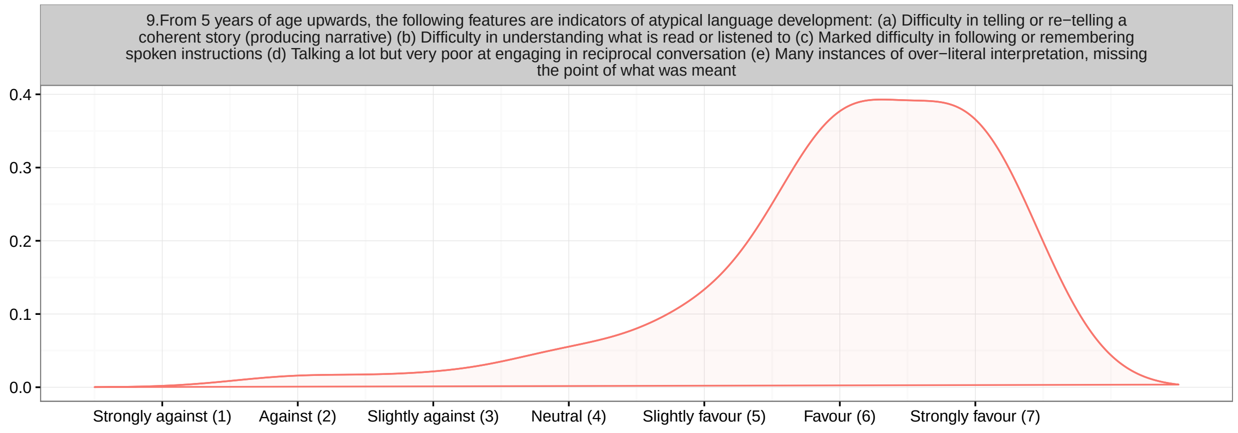

Figure 20: Distribution of responses to statement 9. The bold vertical line coloured red is Anonymous's response to this question for reference.

Table 9: Comments for each statement.

ResponseID	Q9B
$R_5uxk08XTwJpUk9D$	Same comments as before - these definitely pick out children with language problems in school settings but are likely to signal other problems - these may be hidden symptoms of ASD in girls for example.
$R_4HGIGYFIvMxLWcJ$	Again, this statement excludes children who would demonstrate atypical development, for example children who show difficulties in producing more advanced discourse, including expository and persuasive discourse.
$R_0ofhSCmeppIQ8kt$	These milestones are interesting. They're a little difficult to pin down, but they are much like a developmental checklist. Is this the direction we need to take though? Does it move us away from how professionals in speech and language need to identify children?
$R_bQ13TaeUPFsxVJP$	Especially in older groups, it is important to flag more subtle issues in communication. This could be easily achieved by using a phrase such as 'strong indicators'.
$R_0Gj2hZlxlaPtHbT$	All of the children described above will require simultaneous referrals to Community Peadiatricians for assessment & early recognition of co-morbid neurodevelopmental difficulties/disorders. This is essential when formulating the final assessment pathway
$R_bwwc7dPFEcp1azH$	And poor intelligibility (see above)
$R_2o7JoTNgC3lqSIR$	Yes, though of course not all children will have all features. Apart from (a), there are not many markers here for expressive difficulties specifically.
$R_e5KJQmN6txthTRX$	what about expressive skills? This seems rather focused on receptive skills. It maybe that is hard to produce a clear marker over 5 years of age. What are these indicators for?

$R_2hLYvspULpn8NnL$	d) seems to refer primarily to ASD; however, children with SLI may also talk a lot and use basic reciprocal conversation but show rambling, discursive, disorganized styles of speech (Hart et al., 2004) that are equally handicapping, especially in academic situations, and fail to lay a strong basis for writing development.
$R_6JOosydU46ZndMF$	These features are all rather subjective. a) Many 5+ yr-olds have difficulty telling/retelling a coherent narrative because of cognitive demands associated with keeping listener's perspective in mind, while organizing narrative events etc, so depends on social and cognitive demands as well as communicative demand of the context; b) contextually dependent - depends of how the 'reader' reads to the youngster as well as on the material that is selected.; c) again contextually dependent & is often a problem with the interlocutor- many teachers and caregivers string spoken instructions together in rapid-fire way - but when shown how to chunk and repeat exactly, without adding more language, the child understands well.d) very challenging - many very very bright children talk +++++ and might have difficulty engaging in or interested in reciprocal communication if the interlocutor doesn't understand (especially peer interlocutors)
$R_71b9fvukXBUQ5dr$	Speech is creeping in, which has different issues (non-developmental processes for example). If it is to be included, need such additional descriptions. Could also include surface grammar errors.
$R_834xbT3yZzu1O7z$	see above
$R_3rrKtkb2VvC3uG9$	Several of these indicators are likely to be vulnerable to contextual effects, and seem very school-centric which is, in turn, very dependent on teaching/observer skills. It overlooks more specific language vulnerabilities that can be informative. Exactly how do we know if children are good at engaging in reciprocal conversation–with whom, under what circumstances, on what topic?
$R_5C49A94jWehNBB3$	Shouldn't we also be including "low levels of peer initiation/conversational passivity" in to this list?
$R_cLU7KRGW2XvEql7$	of course, these difficulties may manifest in many different ways
$R_eOEFfbvY55KRtRP$	Great to have emphasis on post-5 discourse level development, but persistence of grammatical errors alone could be seen as atypical even with relatively sound discourse level development (although both aspects are likely to be affected)
$R_23qAFVuJCo6YHOd$	Again, this seems to be asking about all communication. If our focus is 'language' I would agree - only assuming we are including pragmatic language in the construct of language difficulty.
$R_8bIXFrv4VBlvVyZ$	Item (a) on the list needs better specification-observed difficulties producing a narrative might relate to story order/sequence and/or inclusion of main event and characters and/or difficulties with local structure-sentence length, use of simple and complex structures and expected grammatical features. An observed concern with any one of these is relevant. So we need to ensure that people deciding whether a child met criteria or not, would be prepared to refer where a child's narrative contained key story features but had structural/grammatical weaknesses.
$R_7WXquZJy8WlgXAx$	these are indications, but as above re specifcity. And they are not exclusive
$R_9U2zxMLVAPcvQUd$	"difficulty understanding what is read" can be interpreted that the child should not only be reading by 5 but also comprehending what she is reading. It needs some adjustment to be clear that we are talking about what is read TO not BY the child. / / The word "reciprocal" could be jargon; could consider changing to "back and forth conversation"
$R_cCuacCYZiqQHKgl$	I think talking little and very limited conversational initiation and unelicited conversational continuation is probably more likely than talking a lot. Item "d" sounds like cocktail party speech that I have seen in spina bifida cases. But I don't see it very much in the run of the mill language impaired child.
$R_e9cPjWuFpcer4B7$	I agree with these overall but they do automatically include many low SES children.
$R_3DfMsLnqK54HqcZ$	Should be reworded as "children should be seen for specialist assessment if".rather than "features indicative of atypical development" - see above comments. Would need greater specification for non-specialists (what is a marked difficulty). Also would need an upper age limit (e.g. adolescents with LI may be able to master narrative but not expository discourse.

2.2 Aspects of language assessment

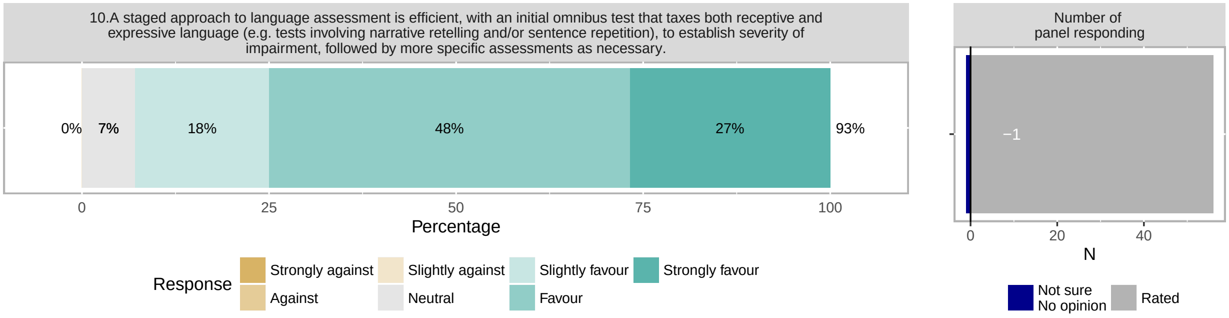

Figure 21: Percentage of panel members in each response category to statement 10. The percentages shown at each end of the scale are the cumulative percentages for the top and bottom three categories respectively.

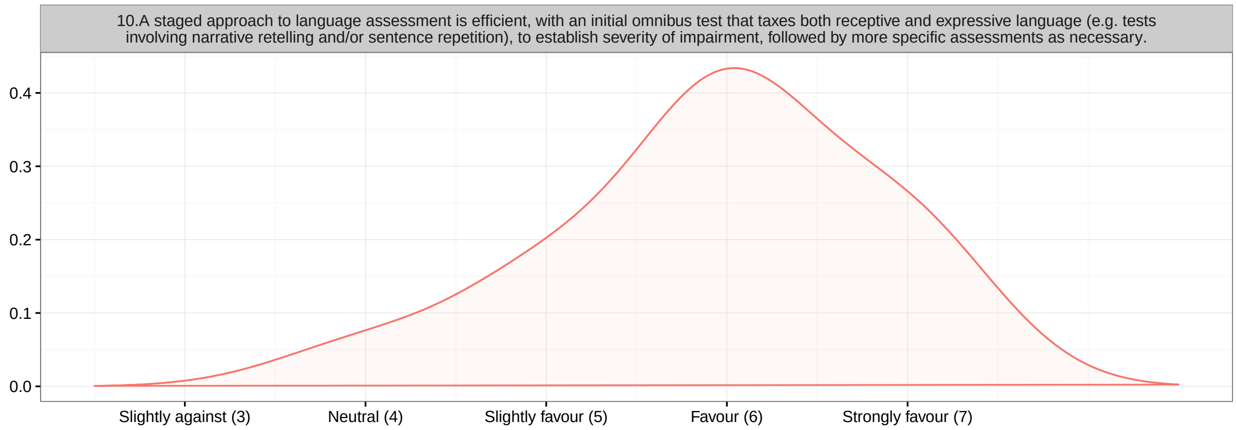

Figure 22: Distribution of responses to statement 10. The bold vertical line coloured red is Anonymous's response to this question for reference.

Table 10: Comments for each statement.

ResponseID	Q10B
$R_1L0uyOsRR9gYKAB$	I have not come across 'omnibus' as a term in this way? Is it similar to the concept of' universal' which is used in child health surveillance?
$R_ba8iHG84IJ8cW7X$	A staged approach to assessment reciprocally linked to intervention and feedback from parents/carers/teachers /others.
$R_5uxk08XTwJpUk9D$	The omnibus test can lead the clinician to more fine-tuned assessments
$R_5cd8BDkYcGfGLKl$	The problem is the most commonly used omnibus test is the CELF, which is not a great test and takes a huge amount of time!! I think an initial test with narrative, e.g. the Bus Story, provides a huge amount of information about comprehension and production and can inform further testing. Also a great prognostic indicator of persistent LI and response to reading comprehension interventions.
$R_8AhxnQPe8mJkUoR$	Evaluation of language in the preschool period that addresses assessment issues. / Conti - Ramsden, G., & Durkin, K. (2012). Language development and assessment in the preschool period. Neuropsychology review, 22(4), 384-401. /
$R_2f9ctxaHBJuJdLD$	The ACE is particularly useful and a cognitive assessment probably needs to be undertaken well before choosing the next more specific test

$R_4HGIGYFIvMxLWcJ$	I wonder if this is a deliberate move away from using, for example, CELF core subtests for testing children's expressive and receptive language at word – and sentence level? the idea of an initial omnibus test is appealing, but I am not convinced that a test involving narrative retelling combined with a sentence repetition test would do the job.
$R_0ofhSCmeppIQ8kt$	Yes, this is one good approach for a comprehensive assessment. It might be the case that not every child needs a full comprehensive assessment.
$R_6Dvhy7Alhw5wqIR$	at the right age the Bus story is very helpful
$R_bwwc7dPFEcp1azH$	Yes although the assessment will need to consider informal methods too as some aspects such as pragmatic language are not always picked up by test batteries.
$R_2o7JoTNgC3lqSIR$	Yes, provided that this is allied with an effective functional assessment.
$R_6LIAgEx6sspizpX$	Narrative is an effective way to observe the child's difficulties with all aspects of language. Good observation, followed by a considered approach to other assessments needed should refine the assessment process. This supports better descriptions of children's language profiles and intervention planning and reduces the need for blanket use of assessments which can be difficult for the child.
$R_e5KJQmN6txthTRX$	this must be backed up with functional assessment by SLTs.
$R_6JOosydU46ZndMF$	In the abstract, yes this sounds sensible. But it depends what approach has been taken prior to this omnibus test. For instance, a strong clinical interview is essential to ascertain nature, severity & duration of problem,,developmental, medical, family history etc. While the clinical interview (especially semi-structured interview) is the cornerstone of meantl health assessment, — unclear whether this is standard for SLPs. Are there well-developed semi-structured interviews for communication problems? I know of one developed for selective mutism (Martinez Yj, et al Canadian J School Psychology 2015 ...
$R_71b9fvukXBUQ5dr$	Needs to be appropriately standardised.
$R_1TXxdyLg1UFCx4V$	This doesn't stipulate if the assessment will be normed/standardised. If so it will allow accurate evaluation against peers which is of value but this may make meaningful evaluation for children with learning disability or other conditions more problematic
$R_dguQPTfUoDzSKB7$	this is unclear. The omnibus test can be a major problem depending of the validity. Screening may be more appropriate although still dependent on the validity for better or worse results.
$R_cYBwzqu4ivWh9qJ$	This very much depends on what the consensus is on 'severity' and which and when assessments are deemed necessary. This will need further definition.
$R_cIxZunCo2wnTfVj$	In general, I'm ok with the statement, but would change a few words to change the emphasis. I think a battery of measures will always be necessary in order to get a full assessment, and a comprehensive assessment should include a spontaneous language sample. The statement that provides the example of an omnibus test, as in one that involves narratives retelling, etc., needs to indicate that the type of test that taxes both receptive and expressive language will vary according to child's age. So, narrative retelling would just not be appropriate for young children. Finally, I do agree that a staged approach is efficient, but am not sure it's effective. But, if one uses the results of the initial test to help structure and guide subsequent assessment, that's one way efficiency can be increased.
$R_834xbT3yZzu1O7z$	I agree that testing is important, however I feel that in order to establish severity functional assessment (informal observation for example) must be included as well as standardised testing
$R_3rrKtkb2VvC3uG9$	How does one assess severity from an omnibus test? by the standard score, requiring both recep and exp language subtests? is a language impairment more or less severe if the low test score is accompanied by good sentence repetition skills? / this seems like clinical practice guidelines now many decades old.
$R_cLU7KRGW2XvEql7$	what do you mean by an 'omnibus test'?
$R_eOEFfbvY55KRtRP$	In practice, many SLTs will favour an observational and informal approach to gain insight into what to test. They wouldn't therefore necessarily take exactly the same staged approach as described here, although there is merit init. So one might start with say a play based language sample or story telling task and then drill down into specific areas such as word-finding and sentence comprehension. Many SLTs will only do a omnibus test if required for the purposes of formal reporting back or access to a specific service.

$R_23qAFVuJCo6YHOd$	I agree a staged approach is needed. However I think: / 1) the first stage should be gaining information through questionnaire/interview about concerns on different areas of language. This can then lead to assessing in the right areas. I am unconvinced that a single assessment or two can pick up on ANY language difficulties. Alternatively a screen which involves challenging tasks tapping all aspects of language, not giving a standardised score but identifying areas of concern to then assess fully. / 2) I am also concerned even if a single assessment can cover all language processes, if difficulties are specific (e.g. with semantics) then they may only show small effects on such a test and therefore get lost. However, the difficulties could have a major impact on learning and communication. We need to be able to identify all types of language difficulties in specific areas, and not just the children who have the most severe difficulties and difficulties affecting all areas of language.
$R_8bIXFrv4VBlvVyZ$	Yes but the omnibus test and its components are important but not all tests are equal in terms of construct validity (e.g. where assessment subtests are too weighted toward general knowledge rather than underlying linguistic representations) or subtests conducted at different ages may have reduced sensitivity; some omnibus assessments are very limited in scope for example to capture connected langauge or specific grammatical markers
$R_6JZKVRyNZK6U0zX$	While I favour a staged approach, the question only allows for an omnibus standardised test in the first instance. I would add language sampling at this stage, even if only informal (ie without accompanying transcription and analysis), since one would be able to judge conversational and pragmatic ability – two features not easily assessed with standardised tests.
$R_7WXquZJy8WlgXAx$	Yes. I have indicated this is a previous answer - an initial 'screening' for want of a better word, and then more indepth investiugation as required
$R_cCuacCYZiqQHKgl$	I think the standardized assessments are fine for determining the child's language status relative to other children and thus for making case determination decisions. Often these tests are not intended or designed for making decisions regarding treatment goal. The criteria for treatment goals may be driven much more on the grounds of functional need and we don't have many good tools for this.
$R_e9cPjWuFpcer4B7$	Depends on the age of the child and the test in question.
$R_3DfMsLnqK54HqcZ$	Yes I think these are a good starting point - with further more in depth assessment following indications of impairment.

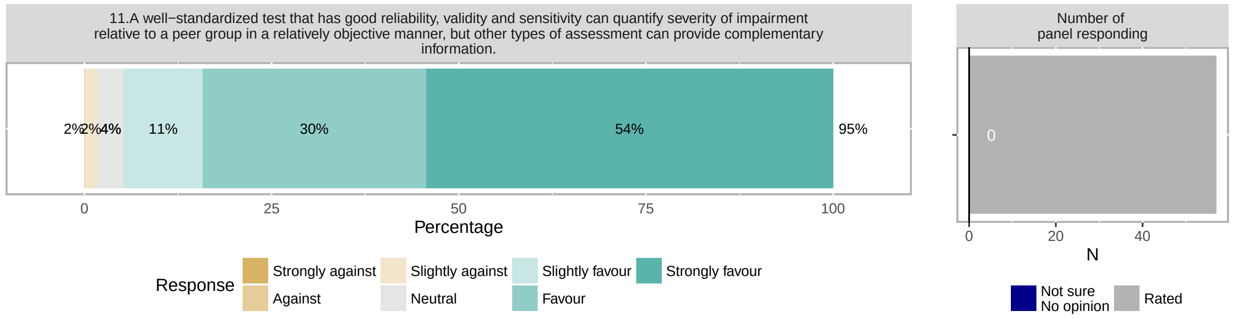

Figure 23: Percentage of panel members in each response category to statement 11. The percentages shown at each end of the scale are the cumulative percentages for the top and bottom three categories respectively.

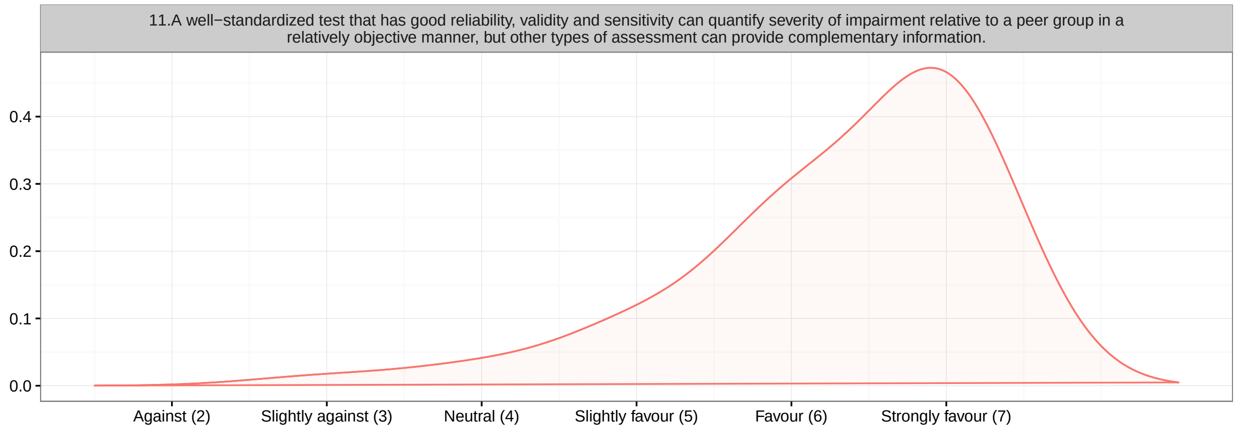

Figure 24: Distribution of responses to statement 11. The bold vertical line coloured red is Anonymous's response to this question for reference.

Table 11: Comments for each statement.

ResponseID	Q11B
$R_1L0uyOsRR9gYKAB$	Should specificity be mentioned too as a test characteristic as a measure of true negatives is important. Sometimes measures of langaue structure appear OK but there are difficulties in functional use and I could envisage that some children who appear to be unaffected on a standardised test could be in this category if their families or educators are concerned
$R_ba8iHG84IJ8cW7X$	But we need to be aware of subtle / inherent cultural biases within tests.
$R_5cd8BDkYcGfGLKl$	Tests do different things - if you want to get at severity, you need a standardised test. If you want to look at funcitional impact, you will need parent report. If you want to plan therapy, more detailed assessment of particular language features is warranted. And pragmatics is difficult to assess in standard way.
$R_4HGIGYFIvMxLWcJ$	Yes, I am pleased to see a change from 'can provide' to 'are needed' to provide complementary information.
$R_bwwc7dPFEcp1azH$	Many standardised tests are not available outside of the UK/US and are not appropriate for multilingual/multicultural clients or from with SES. Also see point re: pragmatics. Clinicians need to be more confident in using their clinical skills and experience in diagnoses and not hide behind the scores provided in a standardised test. Educational policies need to also take note of clinical opinions and not have scores on a standardised test as the only means by which additional help can be accessed
$R_2o7JoTNgC3lqSIR$	Yes, particularly functional assessments of real-life situations and classroom/playground observations etc.

$R_6LIAgEx6sspizpX$	Standardized tests are important but don't give a complete picture of the child's strengths and difficulties
$R_2hLYvspULpn8NnL$	In addition to criterion-referenced testing and observation, analysis of spontaneous speech should be included in the follow-up assessment (Paul & Norbury, 2012).
$R_6JOosydU46ZndMF$	Absolutely - great danger in relying solely on scores from standardized tests irrespective of reliability and validity: they are necessary but not sufficient for diagnosis; need supporting evidence from other sources of information
$R_71b9fvukXBUQ5dr$	Depends if you are asking the first question only (relating the child to a peer group) or needing to answer some other question? The stamens is trivially true, but what 'complimentary' information is needed that cannot be gained by a standardised measure should be specified.
$R_1TXxdyLg1UFCx4V$	As per my comments above in addition to those in the notes re: naturalistic observation etc accompanying this point
$R_cYBwzqu4ivWh9qJ$	Peer interaction, the ability to concentrate, listen and understand in a group, crowded setting or classroom also affect the child or young person. 1:1 assessments can miss the implications of other settings.
$R_834xbT3yZzu1O7z$	As stated above, true severity can be demonstrated through assessment of functional skills, and potential via dynamic assessment approaches
$R_3rrKtkb2VvC3uG9$	the devil in this statement is in the last clause...it is so vague as to be meaningless
$R_cLU7KRGW2XvEql7$	it is essential to use information from a range of sources and to continue to inform our view by adding to that information
$R_8bIXFrv4VBlvVyZ$	my concerns relate to whether we have many tests that have good reliability, validity and sensitivity, important the word "relatively" is retained and that the limitations of existing standardised assessment tools really are emphasised
$R_6JZKVRyNZK6U0zX$	Other measures of a test's diagnostic accuracy could be added to the 3 mentioned in the question, such as positive and negative likelihood ratios, etc.
$R_7WXquZJy8WlgXAx$	Yes, I feel strongly about this. a standardised assessment plays a role, but does not paint the whole picture on 2 counts. firstly, it does not necessarily idenitfy functional performance as in your background paper but also, it may not pick up some of the aspects of language impairment such as pragmatic elements. as you say in your paper, there are laregly useful for identification not necessarily for measures of progress
$R_1QTm7VrpDX1OAi9$	Especially for planning intervention.
$R_6mrinfsu6CeSmBn$	Working with a predominately bilingual / EAL population, scores cannot be used to quantify severity of impairment relaitve to their peers group as we do not have the standardisation data available. other types of assessment are very important.
$R_9uJ5LinD5e8X5Yh$	Some tests do not give the holistic picture eg BPVS - 1 word only, TROG - high scoring, and some give a very good indication of functional classroom skills eg non picture supported - understanding spoken paras, concepts and directions (CELF).
$R_cCuacCYZiqQHKgl$	Standardized tests are often berated for their lack of ecological validity, but they are often more reliable than unstructured measures. Without reliability we can't have validity of any sort. The use of unstructured or naturalistic observations are useful in concert with standardized evidence.
$R_es7hPPlfD7bdd65$	I tend to worry about getting lost in the reeds of "spiky" profiles, where much of what looks like individual variation is in fact the vagaries of standard scores. For instance the difference between the 10th and 25th percentile on some standard tests is only 1 or 2 items when we're dealing with 5 year olds. Yet I've read clinical reports that have made a big deal of a 10-15 percentile difference between two tasks.
$R_e9cPjWuFpcer4B7$	It depends on the test. Well standardised tests do not necessarily agree because even the omnibus tests tap into different constructs.
$R_6tiOrhFOdV4NANf$	While I agree that a well standardized test has the ability to quantify severity of impairment, such tools are often not available for all populations/communities (e.g., culturally or linguistically diverse groups, English speakers outside the UK/USA). /

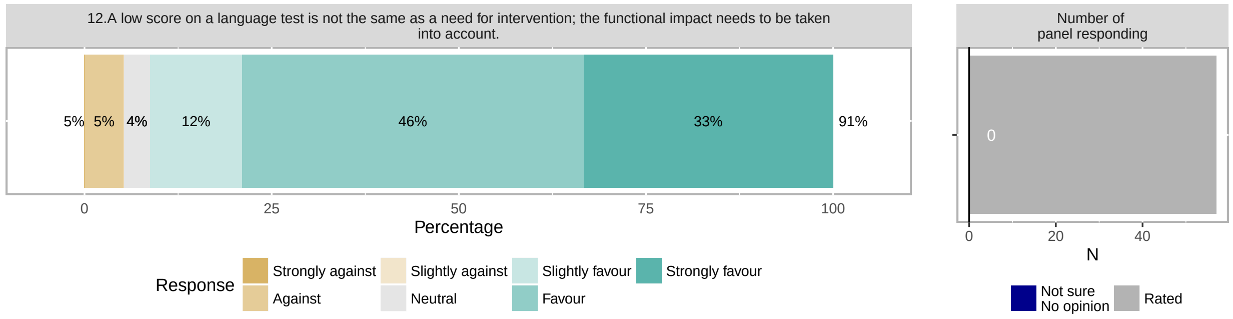

Figure 25: Percentage of panel members in each response category to statement 12. The percentages shown at each end of the scale are the cumulative percentages for the top and bottom three categories respectively.

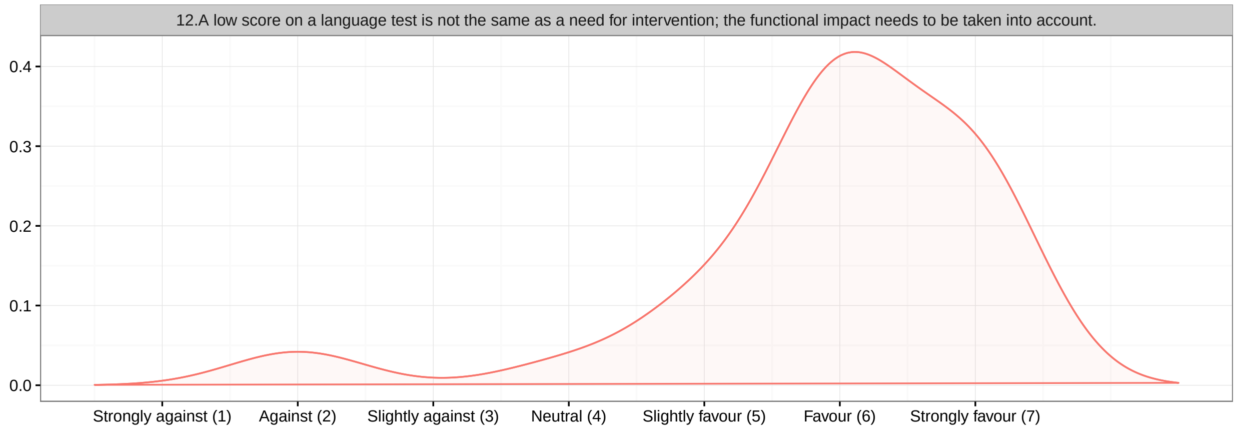

Figure 26: Distribution of responses to statement 12. The bold vertical line coloured red is Anonymous's response to this question for reference.

Table 12: Comments for each statement.

ResponseID	Q12B
$R_1L0uyOsRR9gYKAB$	Whilst I agree with this staetment I am also a bit concerned about how one can operationalise the functional impact assessment particularly for young children. With the contributions of visual cues and context at home and the 'child centred' approach of nurseries it is possible that a child may circumvent their language difficlties in such a facilitating enviroment but then struggle as soon as they enter school
$R_5uxk08XTwJpUk9D$	I am concerned that many parents and teachers lack the sensitivity to LI once a child is able to speak - relying on them to offer information about functional impairment especially in preschoolers may lead the clinician astray. Functional impairment may show up later - for example once the child reaches school and begins learning to read.
$R_2f9ctxaHBJuJdLD$	This applies to all tests and the tests of course should attempt to assess impairment and impact on daily functioning so it is somewhat circular and points towards future research.
$R_6Dvhy7Alhw5wqIR$	lots of reasons–that i sthe job of the assessor
$R_bwwc7dPFEcp1azH$	Particularly for multilingual/multicultural children and with low SES
$R_2o7JoTNgC3lqSIR$	This might be true, although it would depend how low the score is. If there is a very low score (at least 2 SD below the norm), this should be looked at very closely to check whether or not intervention is required. There is a risk that if you ignore low scores you ignore something that might not be causing a significant difficulty at the moment, but might not go on to do so if not addressed.

$R_e5KJQmN6txthTRX$	I would be very wary about not responding to a very low test score.- low is not actually defined here so its not clear what in meant. / Anyone taking the decision not to respond to a low sore needs to have the necessary skills and experience.
$R_6JOosydU46ZndMF$	a single low score on language test should never be the sole reason for intervention; need several sources of information as well as functional impact. How do SLPs assess functional impact? Is there a widely used interview, rating scale etc?
$R_71b9fvukXBUQ5dr$	But even harder to assess than language level!
$R_1TXxdyLg1UFCx4V$	This is particularly the case for one off assessments in a different context e.g. clinic or where adjustments to individual needs such as problems with compliance have not been taken into account
$R_cYBwzqu4ivWh9qJ$	if the test is deemed necessary to carry out then it should follow that intervention is also necessary, an adjustment for the child in this area of need may have wider reaching consequences and also prevent other difficulties arising.
$R_6RlkuyWJYcIIsmN$	A low score suggests that further assessment is needed in order to ascertain its basis.
$R_834xbT3yZzu1O7z$	I think this depends on what you mean by intervention - I assume you are meaning by a specialist? Communication supportive practice and targeted support would not I feel classify as an intervention, but may be implemented for a child with a low score on a language test.
$R_3rrKtkb2VvC3uG9$	the problem with this approach is that it is a large opening for denial of services because people are not aware of how linguistic impairments can have persistent and pervasive academic and social effects even when a child seems to be "getting by alright." further it can be dreadfully contaminated by misconceptions about SES and a child's assumed levels of intelligence. It is very difficult to empirically define "functional impact".
$R_5C49A94jWehNBB3$	Functional impact should include future risks.
$R_5ceQk7pgvAecMAt$	(Current) functional impact is important, but outcomes associated with particular types of difficulties are also important to consider as their impact may be delayed (e.g., until expectations for reading level / independent learning increase).
$R_cLU7KRGW2XvEql7$	We need to consider confidence intervals and the robustness of the test. Also, some children who score low on tests have strategies so that the functional impact is low, whereas some who apparently do better on a standardised test may cope badly when communicating. I don't see this item as about goal setting, but about prioritisation and recognising risk factors
$R_23qAFVuJCo6YHOd$	Yes, very important, as the assessments have limitations. Each child's profile of other skills, awareness of their difficulties, ability to use strategies to overcome their difficulties and the demands of their environment will contribute to the impact. These factors won't be picked up with a language test, yet I believe, can contribute significantly impacts on a child's self image and well being.
$R_8bIXFrv4VBlvVyZ$	In general yes, but may need to further qualified with guidance on how to define "low" ? It would be good to see "functional impact" foregrounded more in diagnostic criteria and clinical decision making-in my work I've encountered decision making based only on the results of test scores.
$R_9uJ5LinD5e8X5Yh$	All testing reveals something but prioritisation of intervention is the key to success.........what is the foremost barrier.
$R_cCuacCYZiqQHKgl$	This seems reasonable. However, we don't have many measures that really get at functional impact. To do this we need to define functions and the developmental profile of functional development.
$R_es7hPPlfD7bdd65$	If the language test is at all meaningful, a poor score has functional implications. Otherwise, it's not a useful language test, or else I don't understand the meaning of the word "functional".
$R_e9cPjWuFpcer4B7$	The point is that a low score at one time point is not useful as a criterion.
$R_3DfMsLnqK54HqcZ$	Yes - in line with the ICF model of functioning and disability - however we MUST also consider within this A) risk of later difficulties and B) interaction with the environment - - A) a child may look as though they are doing OK in early schooling but in adolescence problems with (for example) peers or higher level language abilities required for success in exams may emerge with significant consequences for the child's life chances. B) a child may look as though they are functioning OK with low language in one context because of the skilled modifications , strategies and scaffolding in place but then when the demands change (e.g. transition to secondary school) the child no longer functions - so children would need long term surveillance with levels of support increasing and decresing as child needs and environments needs change over time.

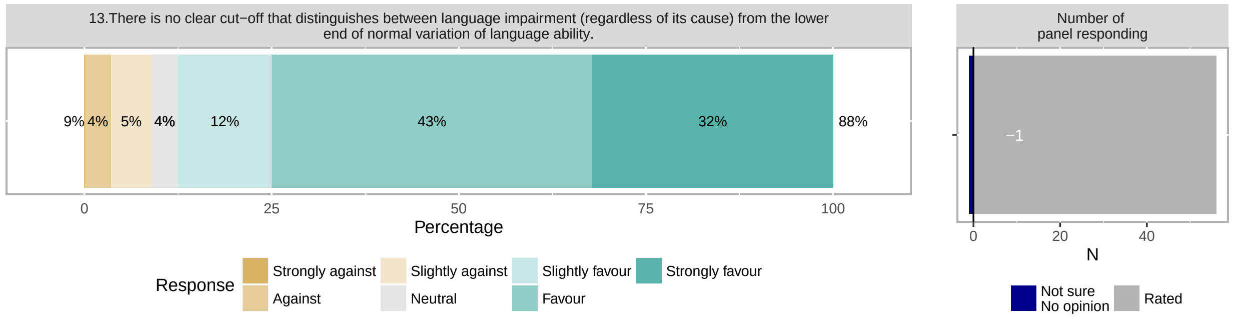

Figure 27: Percentage of panel members in each response category to statement 13. The percentages shown at each end of the scale are the cumulative percentages for the top and bottom three categories respectively.

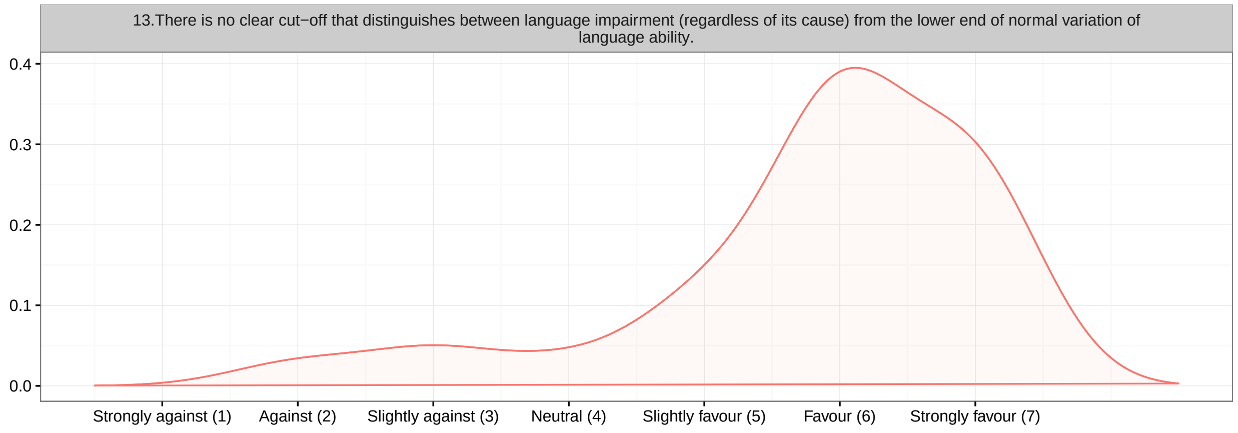

Figure 28: Distribution of responses to statement 13. The bold vertical line coloured red is Anonymous's response to this question for reference.

Table 13: Comments for each statement.

ResponseID	Q13B
$R_1L0uyOsRR9gYKAB$	Again I feel this is true but a cut off needs to be applied somehow to define 'caseness' or participant status
$R_78kR8ERViieBuyF$	This statement is more true of SLI children, so the "regardless of its cause" does not fit with my favoring the statement.
$R_5uxk08XTwJpUk9D$	Behaviorally this is true though in the future biological markers that have a clearer cut-off could emerge. For now, this is a good statement to include.
$R_2f9ctxaHBJuJdLD$	The context and functional impairment point is very helpful and to determine normal variation means a good understanding of context culture family and genes.
$R_0ofhSCmeppIQ8kt$	Yes, and this is what makes our task so difficult. Cultural differences are important here...cultures placing high values on verbal skills may identify impairment at different rates from others.
$R_bQ13TaeUPFsxVJP$	The background report drew a parallel between intervention for obesity and language impairment. A tool commonly used to classify obesity is the body – mass index. Perhaps diagnostic classification for language impairment could include a similar classification in terms of severity, which would mark out the children with the greatest need for intervention?
$R_6Dvhy7Alhw5wqIR$	no clear cut off but some features of the speech/language may help as does past history

$R_2o7JoTNgC3lqSIR$	I think it depends what this question is asking. If you are saying that there is no clear dividing line when you look at the graph of distribution of language skills, then this is true, However, if you are meaning that you cannot draw a line at a certain point and say children below it definitely have a problem, I would disagree. I would think you can. However, where you draw the line would be another question. A higher cut-off might indicate a level of difficulty but not necessarily a need for specialist intervention.
$R_e5KJQmN6txthTRX$	This is true when you look at the graph it is an arbitrary cut off. I think you can indicate points where children will probably need intervention.
$R_2hLYvspULpn8NnL$	This is true, but not helpful to clinicians. I think part of the purpose of this panel should be to establish guidelines for interpreting test and other scores, such as the criteria for ID, which include BOTH scores below 70 on an adaptive assessment AND a score below 70-75 on an IQ test.
$R_6JOosydU46ZndMF$	yes, so any cut-off is somewhat arbitrary - but 'there's the rub' ! Should the cut-off be more or less stringent? Using this logic of norrnal distribution, would an individual in the top end of the distribution also be earmarked as "extraordinary, bizarre, atypical"???
$R_71b9fvukXBUQ5dr$	Some causes - e.g. hearing impairment affect aspects of language such as intonation, stress and vowel production in rather typical ways however, which differ from other causes.
$R_1TXxdyLg1UFCx4V$	I agree with the points made in the notes and would add that the demands of the context should also be considered i.e. different demands on language and literacy in school and home contexts.This is very much the focus of education as an intervention
$R_834xbT3yZzu1O7z$	We have milestones for language development and standardised tests that give information about performance against the norm.
$R_3rrKtkb2VvC3uG9$	This is a bit of a straw man. We have all kinds of measurement systems that provide generally agreed upon definitions of "normal range" vs "range of concern." this twists on a definition of "normal" variation that triggers other assumptions. As with other possible identification approaches, it allows for denial of services to many as we debate what is "normal" and at the same time the literature is filling with evidence of relatively high risk of sustained persistence of low levels of performance over much of childhood and on into adulthood as we find better ways to measure the areas of language weaknesses.
$R_5C49A94jWehNBB3$	This is a loaded statement. What are we measuring here? Some areas of language at some ages do display bi-modal distributions (e.g. morphosyntax).
$R_7WXquZJy8WlgXAx$	As long as this is kept distinct from a decision about intervention, as discussed in the background paper
$R_6mrinfsu6CeSmBn$	Again, cut -offs are difficult to implement in a blingual population anyway. We need to look at types of errors, difficulties, profiles etc. to determine language impairment, not just a score. However, I beleive language impairment is not just about severity but the quality of language learnign and use.
$R_9U2zxMLVAPcvQUd$	Spaulding, T. J., Plante, E., & Farinella, K. A. (2006). Eligibility criteria for language impairment. Language Speech and Hearing Services in Schools, 37(1), 61.
$R_9uJ5LinD5e8X5Yh$	Some LAs use the 1%le score as the indicator - but this is far too crude. Overall scores profile needs to be analysed; there are patterns!
$R_cCuacCYZiqQHKgl$	The evidence seems quite strong that there are not discontinuities in language ability even within the low end, so determining where to put a cut off is arbitrary. There may be some discontinuity in pragmatics, although as the ASD spectrum is presented, there seems to be plenty of grey areas there as well. We need to learn to think about these traits as dimensional and emergent.
$R_es7hPPlfD7bdd65$	Not unless we really believe that LI represents a distinct, natural category. I don't. Everything is just shades of gray.
$R_e9cPjWuFpcer4B7$	These differences are largely socially constructed. We would need threshold beyond which all children are likely to have persistent problems but as Bishop and Edmundson and various Conti - Ramsden papers have shown such a threshold has proved elusive.

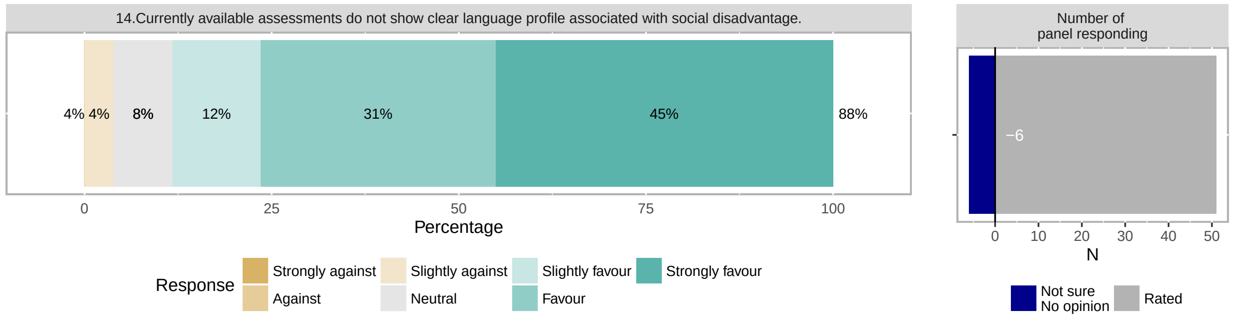

Figure 29: Percentage of panel members in each response category to statement 14. The percentages shown at each end of the scale are the cumulative percentages for the top and bottom three categories respectively.

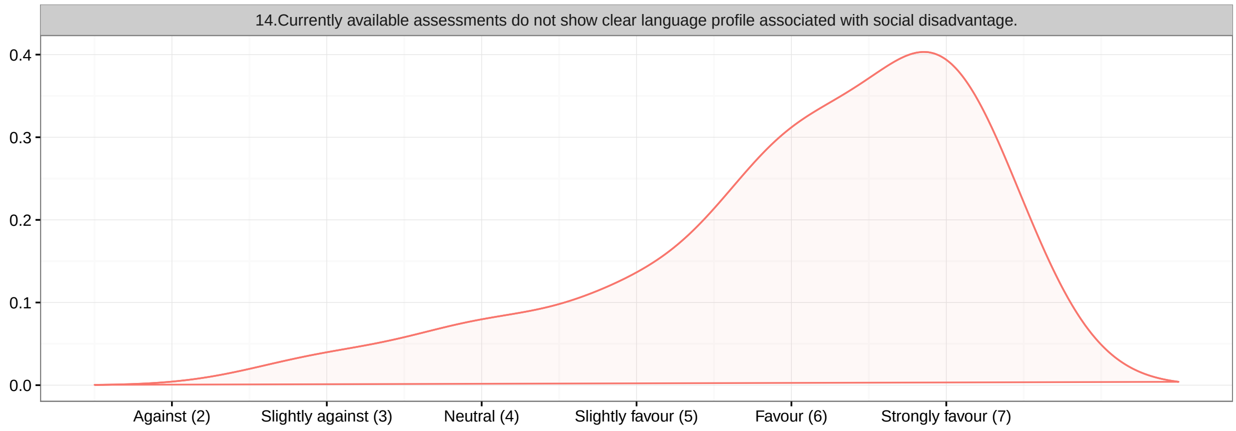

Figure 30: Distribution of responses to statement 14.The bold vertical line coloured red is Anonymous's response to this question for reference.

Table 14: Comments for each statement.

ResponseID	Q14B
$R_1L0uyOsRR9gYKAB$	This is very difficult to answer and is probably unrealistic to think one can identify social disadvantage through the medium of the langaue profile. Rather it should be identified as a co-existing factor. There are so many multi factorial influences eg foetal alcohol exposure, maternal addiction and poverty that I don't think this is an attainable goal.
$R_bQ13TaeUPFsxVJP$	Does this comment mean that some aspect of SES should be assessed by speech – language therapists?
$R_bwwc7dPFEcp1azH$	We have found that children from socially disadvantaged areas are both over and under-diagnosed when certain core language scores from a well-known standardised test are used. further assessment, including dynamic assessment is always warranted in these cases
$R_2o7JoTNgC3lqSIR$	An interesting question is whether it would make a difference if we could distinguish 'genuine' language disorders from those arising from social disadvantage. Would they need different intervention? Do we know how many language impairments are largely the result of social disadvantage? Given the interplay between genes and environment, can we ever really separate them out, other than in the most obvious cases perhaps?
$R_e5KJQmN6txthTRX$	This would seem to be the case at the moment, meaning that we do not know whether there is genuine difference between the two. Establishing whether there is a distinction and, if so, how to identify it, needs to be a priority for research. The association with social disadvantage is one reason why speech and language impairments are not taken as seriously as they should be.

$R_2hLYvspULpn8NnL$	I think this is not entirely true. Chlldren with language disadvantage will NOT show the diagnostic markers of LI (on NWR, rapid naming, etc.), or in pragmatic areas. Although there may not be a clear pattern, there are some differences in the profiles, with children from disadvantaged backgrounds showing marked deficits in vocabulary and complex language and those with LI in morphology.
$R_6JOosydU46ZndMF$	I am unsure what the purpose is of this statement. Implies that of there was such an instrument, then one would differentiate 'atypical' communication development associated with low SES from those with higher SES???
$R_1TXxdyLg1UFCx4V$	I note the point made in the accompanying document re SES and response to treatment and would want to ensure that this should not be used in a way that might disadvantage those from poorer SES contexts
$R_6RlkuyWJYcIIsmN$	My read of the literature on this is that while aetiological factors are many and debatable in low-SES children, it is common to see language scores that are 1-1.5 SDs below published norms. Roy and Chiat's work shows difficulties on both "core" and non-core language abilities. Other work that needs to be considered includes that of Spencer, Clegg & Stackhouse (University of Sheffield).
$R_834xbT3yZzu1O7z$	We are still finding out about the profile of children with language difficulties associated with social deprivation.
$R_3rrKtkb2VvC3uG9$	I thought I understood the item until I read the commentary and now don't know if it is about response to intervention (with the hint that low SES might intefere with intervention outcomes). Sees to have multiple assumptions in play. My reading of the literature is that in well executed and well reported the SES effects turn out to be relatively modest, if at all. The causal pathways are likely to be affected by some but clearly not all covariates of low SES. A real complication is that persons with low language abilities are likely to grow up to be persons without advanced educational degrees which in turn affect their SES. If a statement about social advantage is to be included it needs to have more careful wording to avoid these complications in interpretation.
$R_5C49A94jWehNBB3$	Vocabulary has been shown (in some reports) to be more strongly affected by social disadvantage than grammar and verbal memory.
$R_5ceQk7pgvAecMAt$	I find the wording unclear. What does it mean to show a profile? Does this mean that empirically no such profile emerges in samples defined by degree of social disadvantage, or that this kind of research has not yet been done?
$R_1z8h1XMT676UOwd$	This is generally true but something like Dynamic Assessment might distinguish a language learning problem from lack of exposure/disadvantage.
$R_cLU7KRGW2XvEql7$	Well, of course they don't and really, how could they? We should not even be considering this. This is not really about 'social disadvantage' as a blanket label, but we should be looking at maternal and paternal education and the home language environment. oh, and birth order and gender (god help the third born son of a woman who has no further education after her GCSEs)
$R_8bIXFrv4VBlvVyZ$	At least not based on any research with which I am familiar; but the comments in the background document regarding implications for intervention must be taken into account when making decisions about individual children
$R_9uJ5LinD5e8X5Yh$	Here is my Orange box back into play - you will not get a full picture of social disadvantage aka ASD spectrum if you just look at language scores. The testing is not subtle enough for many. You must look at the whole learning style of the child eg a literal reader, poor comp from text /pix of character motivation, cause and effect difficulties, facts galore but no linkage/transfer/networking. Talk with the teachers!
$R_cCuacCYZiqQHKgl$	This is likely, but social disadvantage has so many dimensions (economic, educational, language differences) that I'm not sure that they all have similar effects on language development.
$R_es7hPPlfD7bdd65$	They might, but we don't have the information we need to assess that to be the case. Either way it's a critical question and I look forward to the field getting a handle on the issue.
$R_e9cPjWuFpcer4B7$	This is true although very few papers have explicitly sought to discriminate between low SES and higher SES language difficulties. Language alone is unlikely to be the sole criterion. The same is true of executive function, literacy etc.
$R_3sXNbQYRlZaMb3L$	This is interesting; I had thought that research shows a vocabulary 'gap' associated with SES, and that at school entry this then becomes predictive of other key outcomes.

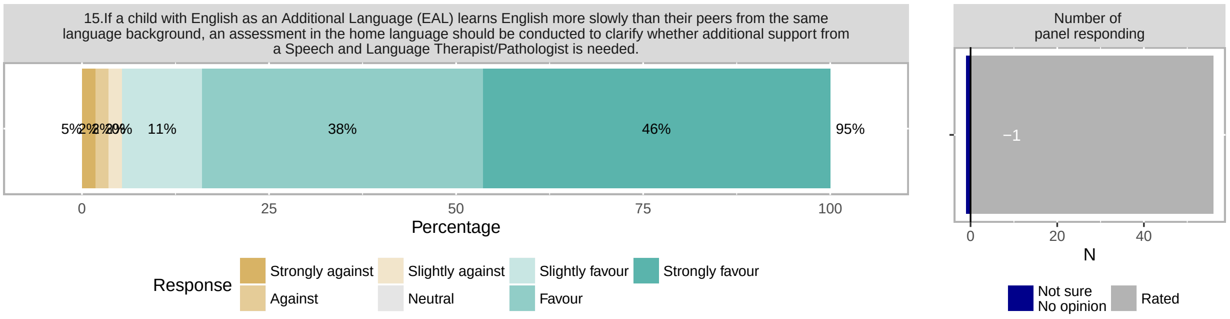

Figure 31: Percentage of panel members in each response category to statement 15. The percentages shown at each end of the scale are the cumulative percentages for the top and bottom three categories respectively.

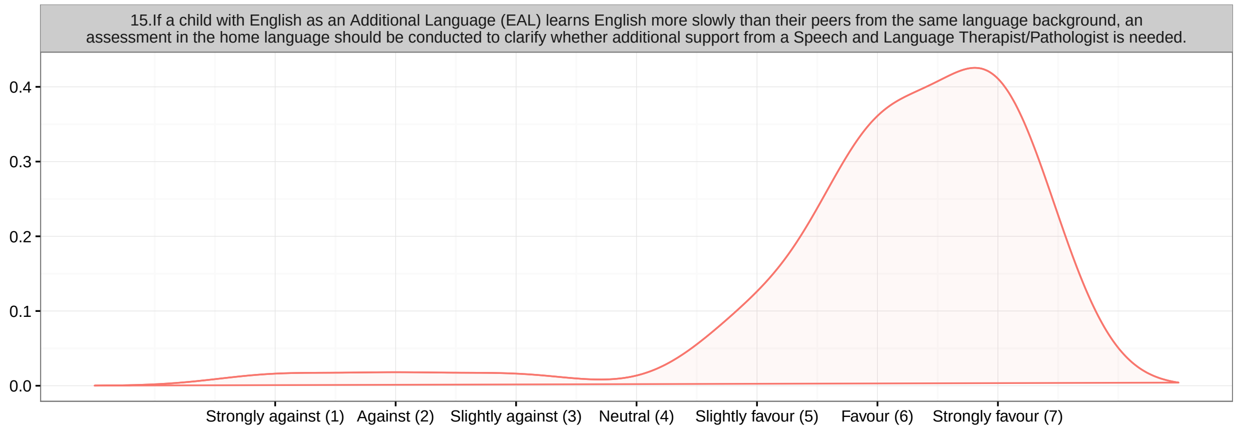

Figure 32: Distribution of responses to statement 15. The bold vertical line coloured red is Anonymous's response to this question for reference.

Table 15: Comments for each statement.

ResponseID	Q15B
$R_1L0uyOsRR9gYKAB$	This requires considerable expertise to make this judgement. 10% of children in ——- local authority area have additional support needs educationally arising from english as a second language so this represents a huge volume of work. Very important but would require a great deal of thought as to how one would assess or operationalise this. There are so many other factors such as older siblings using english, mother perhaps not learning and using english etc so just the fact that another child in nursery from the same language background makes better progress is not sufficient.
$R_5cd8BDkYcGfGLKl$	This is a laudable goal, but very impractical. ———- East London - 120 different language communities. Even in less diverse areas, there are more than 60 different languages in one school district. If we require assessment in the child's home language, many children with EAL will continue to fall through the net. I would suggest that slow progress in English, affecting school progress, should justify additional support and consultation with an SLT. Discussion with family, yes, requirement for formal assessment in home language I think would not be possible.
$R_2f9ctxaHBJuJdLD$	It is worth noting the research on increased executive function skills for those who are bilingual and also the impact of too rapid an acculturation in terms of undermining mental health for immigrants. ————— Multilingualism and SEN and emotional and mental health Routledge Eds Peer and Gordon ———
$R_4HGIGYFIvMxLWcJ$	I strongly agree with this statement. I was not in complete agreement with the rationale provided in the background document that EAL is a risk factor for poor academic achievement as this seems to ignore research showing clear advantages of growing up learning more than one language.

$R_0Gj2hZlxlaPtHbT$	Generally if differentiated performance between same language peers noted, there are often additional developmental/learning needs, hence the need for concurrent multidisciplinary assessment for co – morbidities. Additional assessment in home language may specifically quantify these additional areas of language needs but operational strategies are in English & home language recommendations cannot be effectively delivered. More importantly the neruodevelopmental tests available do reliably identify the profile & pattern of need in EAL & English speaking cohorts. The only adjustment we will need is more frequent administration of assessments in EALs to detect change & adjust provision.
$R_6Dvhy7Alhw5wqIR$	not always easy!
$R_bwwc7dPFEcp1azH$	Children with EAL can benefit from general intervention delivered ideally through school (i.e. not specialist SLT intervention, unless true language impairment is identified though assessment in their home language). The availability of assessments in the home language is problematic. Dynamic assessment shows promise
$R_6LIAgEx6sspizpX$	Essential- factors such as length of exposure to a second language should also be considered
$R_6JOosydU46ZndMF$	Presumably this would depend on age and length of time living in english speaking country/environments. Also this is a long and rather complex statement to process - the criticalcomponent (apart from EAL) is 'learning English more slowly than their peers from the same language background' which is 'unmarked grammatically' and buried in the middle of this statement that is > 40 words long!!!
$R_834xbT3yZzu1O7z$	I think this would depend on if there are concerns about the way the child is learning their home language. I don't think a blanket statement would be appropriate.
$R_3rrKtkb2VvC3uG9$	It is vital to differentiate between simultanous bilingual acquisition and sequential language learning. It is the latter children who must be carefully assessed to avoid confounding language impairments with second language differences. Assessment in the home language is needed but for many languages the proper methods do not yet exist.
$R_cLU7KRGW2XvEql7$	this may not be possible, so some form of questions for parents about how the child performs in their home language
$R_23qAFVuJCo6YHOd$	Does 'more slowly' cover all language difficulties? This may need to be reworded. / Regarding assessment, this could read 'an assessment to clarify whether the same language difficulties exist in the home language'
$R_eG1jl51DiHRqXKB$	Favour but obviously there are relatively few standardized tests in alternate languages that are well standardized.
$R_1QTm7VrpDX1OAi9$	I think we have to recognise that while this is the ideal, in many cases, we do not have the assessments available to do this. We certainly need to try our best to establish the child's language ability in the home language, but saying that an assessment in the home language should be conducted, may be too strong.
$R_9U2zxMLVAPcvQUd$	I'm not a fan of using "their" as a singular pronoun in a scientific document coming from language experts; considering rewording to avoid this. / / Kohnert, K. (2010). Bilingual children with primary language impairment: Issues, evidence and implications for clinical actions. Journal of Communication Disorders, 43(6), 456-473.
$R_9uJ5LinD5e8X5Yh$	We don't serve EAL children well overall I think. Knowledge and assessment techniques let them down. Big area for devt - some team needs to sort us out!
$R_e9cPjWuFpcer4B7$	Yes although in practice this would probably mean most such children NOT being assessed as having LI because there would not be enough people to make such detailed assessments. The alternative of course is within the education system where SLCN is commonly used to describe EAL children in the first few years of schooling.
$R_eLIdYhExxkQtUZn$	I agree with the main thrust of this argument, assuming that the Delphi exercise only relates to countries where the language of the mainstream community and education setting is English. However, I don't think that the issue is restricted to only English. The wording of this statement could refer to 'any language being learnt as an additional language to a child's home language'.
$R_4ORQ8jYm1JwWwN$[illegible]	This is a worthy statment, but the problem is that we lack even rudimentary assessments and knowledge about development in many languages. I don't see the current research effort being able to produce the evidence to support this practise universally.

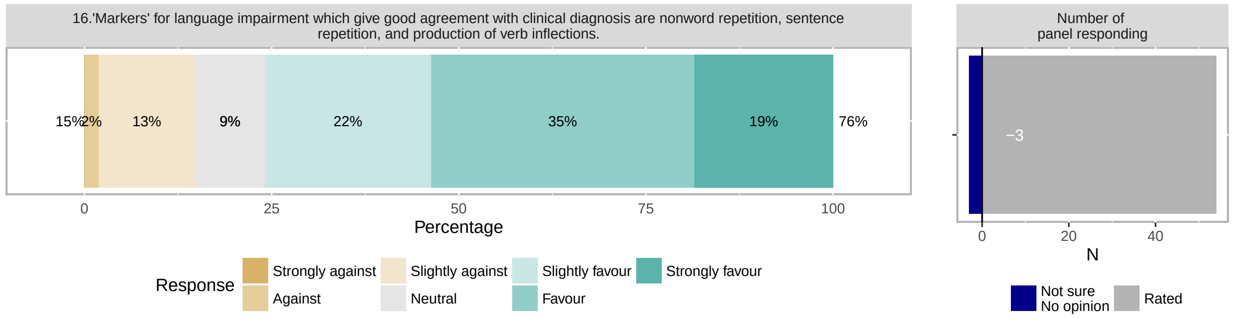

Figure 33: Percentage of panel members in each response category to statement 16. The percentages shown at each end of the scale are the cumulative percentages for the top and bottom three categories respectively.

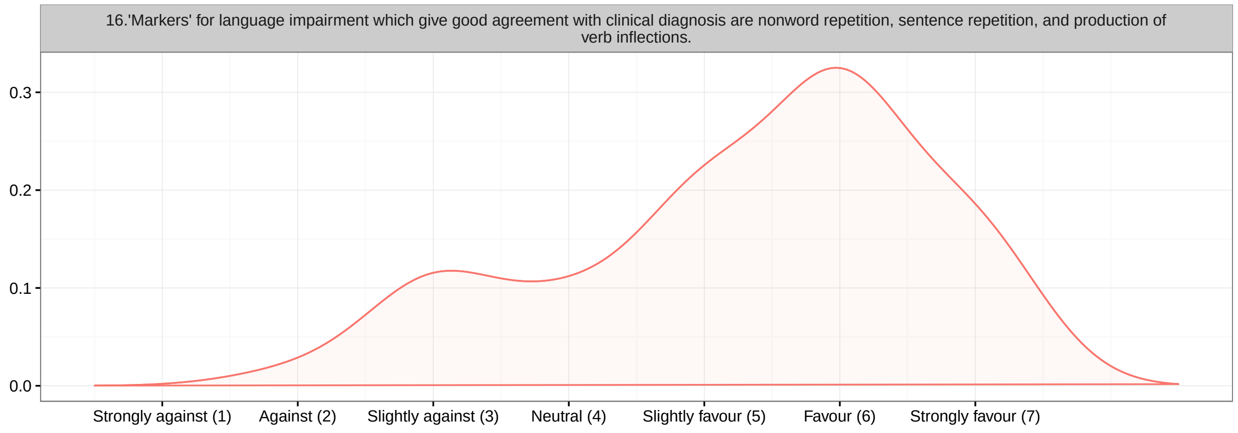

Figure 34: Distribution of responses to statement 16. The bold vertical line coloured red is Anonymous's response to this question for reference.

Table 16: Comments for each statement.

ResponseID	Q16B
$R_bDBwfKBpPTJqjjf$	None of these are consistent enough across people with LI to be used as a diagnostic 'marker'. Asessment of the actual behavioiur (i.e., langiage ability) will always be better.
$R_5uxk08XTwJpUk9D$	The evidence is very strong.
$R_5cd8BDkYcGfGLKl$	I don't think we have sufficient evidence to demonstrate these have better sensitivity/specificity than more traditional measures and the link with intervention targets is even more remote.
$R_8AhxnQPe8mJkUoR$	Markers: reference where the three markers suggested are directly evaluated / Conti Ramsden, G., Botting, N., & Faragher, B. (2001). Psycholinguistic markers for specific language impairment (SLI). Journal of Child Psychology and Psychiatry, 42(6), 741-748. /
$R_bQ13TaeUPFsxVJP$	While it is unclear how these markers line up in functional assessment, they should help screen for language difficulties?
$R_bwwc7dPFEcp1azH$	Yes but these often only apply to English. Also they should perhaps just be an indication that further assessment is needed given their low sensitivity/specificity
$R_2o7JoTNgC3lqSIR$	These appear to be good markers for SLI.
$R_6LIAgEx6sspizpX$	i don't how specific these are to this population
$R_e5KJQmN6txthTRX$	But with the current state of knowledge, we should not rely solely on the presence or absence of these. This might be useful for screening purposes but diagnosis still depends on a full assessment.
$R_2hLYvspULpn8NnL$	may want to add rapid naming (Cody, 2013;Ebert et al., 2014)
$R_6JOosydU46ZndMF$	But performance may be test-dependent. Also if scores on such tests do not agree with clinical diagnosis, what decision is the assessor advised to make?

$R_{7}1b9fvukXBUQ5dr$	Don't know if they do in practice - research suggests they distinguish, but and rather circular if so?
$R_{1}TXxdyLg1UFCx4V$	Whilst I think this is true I am not certain about the functional value of these 'markers' since other factors e.g. vocabulary seem to be important predictors of outcome in individuals. The important issue would be whether these identifying factors have value in informing decisions about intervention and expected outcome; both factors are integral to the establishment of distinct diagnostic categories.
$R_{d}guQPTfUoDzSKB7$	non word repetition is not necessarily sensitive. Current evidence that it is more related to dyslexia. Verb production only applies to English. In Spanish we would focus on other inflections in the noun phrase or mood in the verb.
$R_{c}YBwzqu4ivWh9qJ$	This is not an area in which I am able to give an opinion
$R_{3}rrKtkb2VvC3uG9$	Again, I thought I knew how to respond until I read the commentary. First, it is not "verb inflections" in general, because there are strengths as well as weaknesses. Participals, such as "broken" are quite robust even in youngsters with language impairments. The weaknesses are in some morphosyntactic forms, and equally syntactic as morphological. the issue of how to interpret sensitivity and specificity in a clinical context makes one wonder what this means. That problems of these kinds are not amenable to intervention, or should not receive attention, or should be approached as adjunct skills to other language skills? It is important to keep in mind that nonword repetition has a strong speech component, as well.
$R_{c}LU7KRGW2XvEql7$	and? surely this is just for a narrow range of children? more evidence is essential for this approach to have credibility
$R_{e}OEFfbvY55KRtRP$	My view is that this may work for a proportion of children where the impairment is mainly based around grammatical and phonological memory type problems but not necessarily for others with higher level difficulties
$R_{2}3qAFVuJCo6YHOd$	This would miss semantic- vocabulary type difficulties, inferencing and other pragmatic language difficulties (e.g. giving enough information etc). It may be true for a narrow 'pure' research group with grammatical/ phonological type profiles.
$R_{8}bIXFrv4VBlvVyZ$	all three for children acquiring English as their first language; but production of verb inflections is not a robust marker across languages (as per Leonard's 2014 synthesis; also Armon-Lotem & de Jong 2015) so perhaps change production of verb inflections to mother tongue relevant grammatical markers
$R_{7}WXquZJy8WlgXAx$	I have put neutral here as although I agree from an evidence perspective, I agree with the outline in the background paper - what they tell us in terms of functional impairment is also important and thereforeshould be used with cuation by practitioners, especially if the practitioners carrying out the assessment is a non specialist
$R_{1}QTm7VrpDX1OAi9$	I certainly agree with this statement for sentence repetition and production of verb inflections. However, at an individual level, a significant number of children with language impairments do not have difficulties with non-word repetition (see Ebbels, Dockrell & van der Lely, 2012, Non-word repetition in adolescents with specific language impairment (SLI), International Journal of Language & Communication Disorders, 47, (3), 257–273). Indeed studies have found that NWR only seems to be impaired in children with both language and reading difficulties, not in those with language impairments but good reading accuracy (Baird, G., Slonims, V., Simonoff, E. and Dworzynski, K., 2011, Impairment in non-word repetition: a marker for language impairment or reading impairment? Developmental Medicine and Child Neurology, 53, 711–716; Rispens, J. and Parigger, E., 2010, Non-word repetition in Dutch-speaking children with specific language impairment with and without reading problems. British Journal of Developmental Psychology, 28, 177–188.)
$R_{9}U2zxMLVAPcvQUd$	I thought the Archibald & Joanisse (2009) paper referenced in the background report suggested that NWR had good specificity but low sensitivity, and only had better sensitivity when combined with sentence repetition (which was good enough on its own).
$R_{9}uJ5LinD5e8X5Yh$	I used to think non word repetition was the key to understanding, but usage of this with severe and complex proved me wrong. Many words are non words to our population (even when they are words) so the findings are not secure. ASD spectrum can parrot very nicely. Sentence repetition is memory only maybe....not totally indicative of SLI

$R_cCuacCYZiqQHKgl$	The term marker could be interpreted that there is a latent category and these are sensitive symptoms that are indicators of this category. I would disagree with this. My view is that these different measures are likely to generate more variance in a group of children than other measures and therefore are good indicators language ability in general. If this is true, they are also good markers of language facility. It is also likely that these measures will not be effective at all ages. Sentence repetition and nonword rep. as tasks may be good across ages, but the particular words or sentences that are informative will differ at different points in development.
$R_es7hPPlfD7bdd65$	Yes, as long as our definition of LI is narrowed to what most of the literature envisions it as being. Clearly dyslexia and pragmatic language disorders are not going to follow this same pattern however. So it's important to be clear that we're talking about LI, not ASD, dyslexia and so on.
$R_e9cPjWuFpcer4B7$	We do not have the age here and the wording suggests all three. These may be generally true but may not work at the level of the individual level. So there are children with low non word rep who are generally ok. Sentence rep does work. I am less sure about inflections. Much has been made of the EOI in the U.S. But I am not sure is very meaningful even in adolescence.
$R_6tiOrhFOdV4NANf$	While such markers may give good argeement for children with 'pure' language impairment, they are of limited use for children with comorbid speech and language difficulties.
$R_4ORQ8jYm1JwWwND$	These have not been sufficiently examined in the context of e.g. bilingualsim and low SES. In my view, a useful clinical marker should be an improvement over current standardized language tests and be able to identify a language disorder that is due to neural-developmental factors.
$R_3DfMsLnqK54HqcZ$	These have not been adequately tested in population studies - only in matched groups which artificially inflate their sensitivity - if used they should not be considered 'markers' - this over-states their usefulness- suggesting they are inclusion criteria for specific diagnoses. Sentence repetition and non-word repetition can be used as useful clinical tools to triage whether children might require further language and literacy assessment - they can indicate risk for poor language abilities which may have many potential underlying causes - but not as 'markers' of any diagnoses. I have a big problem with verb inflections - this is English specific - and is only relevant in an age range when we can tell anyway if a child has poor language development without this test - and some children with LI have more vocabulary and semantic based impairments with this does not help to identify.

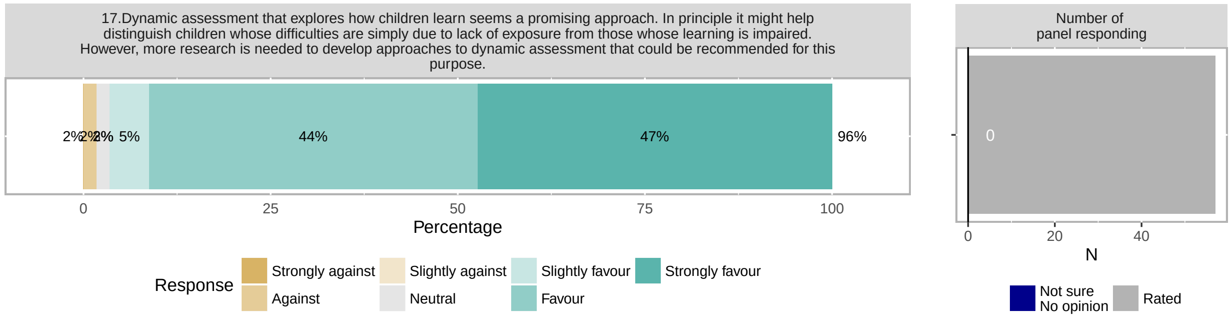

Figure 35: Percentage of panel members in each response category to statement 17. The percentages shown at each end of the scale are the cumulative percentages for the top and bottom three categories respectively.

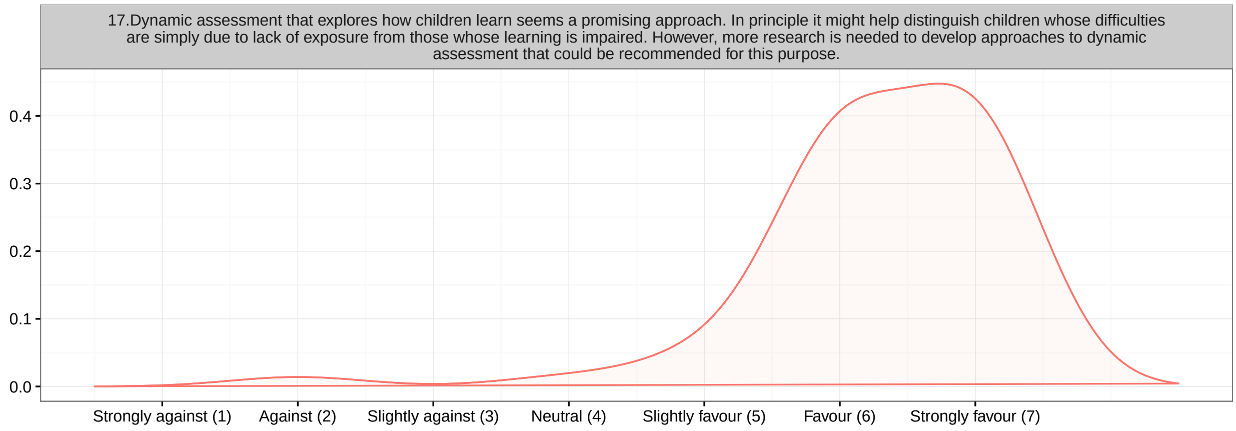

Figure 36: Distribution of responses to statement 17. The bold vertical line coloured red is Anonymous's response to this question for reference.

Table 17: Comments for each statement.

ResponseID	Q17B
$R_1L0uyOsRR9gYKAB$	It is amazing how long dynamic assessment has been the favoured model in educational psychology and yet measurement has not been developed
$R_ba8iHG84IJ8cW7X$	As noted above means of guaging Response to Intervention and ongoing 'dynamic' assessment that provides reliable information about learning and areas for development should be developed.
$R_5cd8BDkYcGfGLKl$	Agree - we need more research on this before we can recommend this approach.
$R_8AhxnQPe8mJkUoR$	I agree this is particularly useful approach for EAL children where it appears that they have not had the opportunity for enough English language exposure. /
$R_0ofhSCmeppIQ8kt$	The key here is that more research is needed.
$R_bQ13TaeUPFsxVJP$	I think this would be a really interesting development and could help differentiate assessment for children with language impairment.
$R_bwwc7dPFEcp1azH$	See above
$R_2o7JoTNgC3lqSIR$	Yes, practitioners carrying out dynamic assessment need to be highly skilled. But yes, the emphasis on SLI being a language learning disability and not just 'being behind' is a very important one and one that should be stressed.
$R_6LIAgEx6sspizpX$	It seems like a good idea in principle but do we know enough about how responses from those two groups of children will differ?
$R_6JOosydU46ZndMF$	So basically, you are arguing against the use of dynamic assessment at this time. There must be a simpler more straight forward way of stating this! Is dynamic assessment intended only to distinguish communication problems from lack of exposure from those with 'impaired learning'?

$R_71b9fvukXBUQ5dr$	Yes - dynamic assessment needs further development.
$R_dguQPTfUoDzSKB7$	agree, certainly it needs to go throught the same scruitiny than standardized assessments for validation
$R_cYBwzqu4ivWh9qJ$	Dynamic assessment is very useful in the context of education but - and a strong but- dynamic assessment is not useful on its own. We also need standardised tests to show where we are starting from when we put interventions in place. I have seen dynamic assessment used by some EPs in place of, instead of alongside, cognitive assessment and the consequent reports are woolly to the point of managing to avoid giving any opportunity for access to intervention resources.
$R_6RlkuyWJYcIIsmN$	My major caveat on this point is that I don't think there is anything "simple" about lack of exposure. Lack of exposure equates to impairment in a functional sense, and significantly impedes both academic and social success.
$R_834xbT3yZzu1O7z$	A dynamic approach to assessment, an approach which looks at potential for learning rather than a static level of achievement, has been shown to be useful in diagnosing language impairment as well as informing future interventions (Pena, Resendiz and Gillam (2007) the Role of clinical judgements of modifiability in the diagnosis of language impairment Advances in Speech-Language Pathology)
$R_3rrKtkb2VvC3uG9$	This is another statement that is agreeable if it gets a rather vacuous reading. Of course, there are great challenges in actually implementing and defining criteria of outcomes for dynamic assessment, arguably more daunting than those evident in static assessments.
$R_cLU7KRGW2XvEql7$	this is a misconception that DA is a 'thing' like a CELF or TROG, there are some 'tests' that are constructed as DA, but actually, skilled practitioners should be able to construct a DA with mediated learning to exactly suit the child with whom they are working.
$R_8bIXFrv4VBlvVyZ$	while it is necessary, I don't know that the need for further research is what is truly precluding greater uptake and use of dynamic assessments in clinical practice; expediency and time constraints may be hindering uptake-so static standardised Ax is chosen for convenience and familiarity; we need more use in practice of dynamic approaches (test-teach-retest; Mediated learning environment; graduated prompting); wider use of DA tools that have been developed and CPD for qualified clinicians in this area
$R_7WXquZJy8WlgXAx$	This would greatly enhance the tools we have to assess and plan intervention - therefore enhancing support for children
$R_1QTm7VrpDX1OAi9$	I think there are some other relevant references from Elizabeth Pena
$R_9uJ5LinD5e8X5Yh$	Dynamic asst is a very useful tool and refinement would be gratefully received. Usually staff use speed of learning as an indicator of lack of exposure V lang impairment - can work but Learning Difficulties usually assumed.. /
$R_cCuacCYZiqQHKgl$	It has been promising for a long time, but continues to be pretty ad hoc.
$R_es7hPPlfD7bdd65$	Yes. But also I think we need to be clear that an impairment is an impairment and we shouldn't assume that kids who respond quickly to intervention were somehow not impaired in the first place.
$R_e9cPjWuFpcer4B7$	——- special issue of IJSLP which was not picked up in your review. These issues have been much discussed and many are already doing this and assessments are available. Of course there is more could be done.
$R_4ORQ8jYm1JwWwND$	I would not single out dynamic assessment as the promising approch for future research. I think there are other similar approaches that focus on evaluating learning rate, but are more in line with current research on the nature of language development.
$R_3sXNbQYRlZaMb3L$	Interesting. I wonder whether DA affiliates might see this description as a misuse of DA; its purpose is to work out each child's zone of proximal development, and how to best scaffold their learning, rather than to sort between children who can and cannot learn...

$R_3DfMsLnqK54HqcZ$	I think we are a long way away from this and we need a much more nuanced debate and discussion about gene environment interactions - I doubt that there are many children (other than those with EAL) for whom language problems “simply due to lack of exposure” could be identified using dynamic assessment approaches. My understanding is that the child’s language learning mechanism is tested in dynamic assessment. If one accepts that language knowledge and learning mechanism are separable then one could identify children whose language delay is “simply due to lack of exposure”. However learning is influenced by the nature of long term knowledge and exposure would affect that long term knowledge and hence learning.......so I think things are a bit more complicated than this.....but I would welcome it as an area for future research.

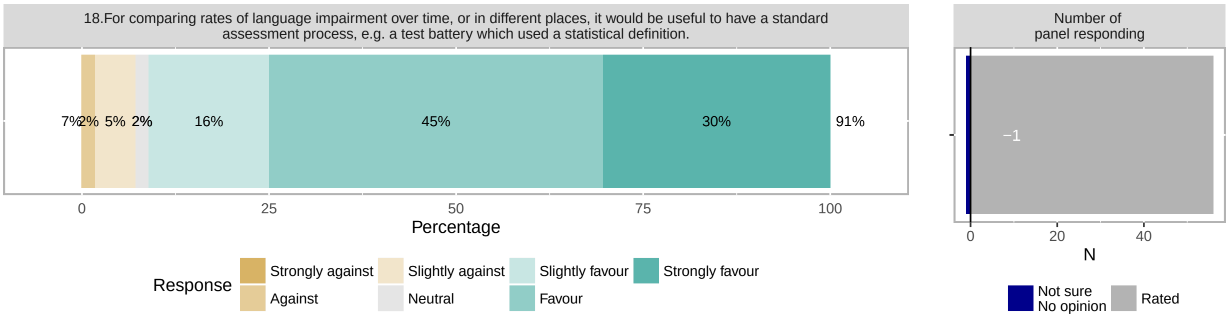

Figure 37: Percentage of panel members in each response category to statement 18. The percentages shown at each end of the scale are the cumulative percentages for the top and bottom three categories respectively.

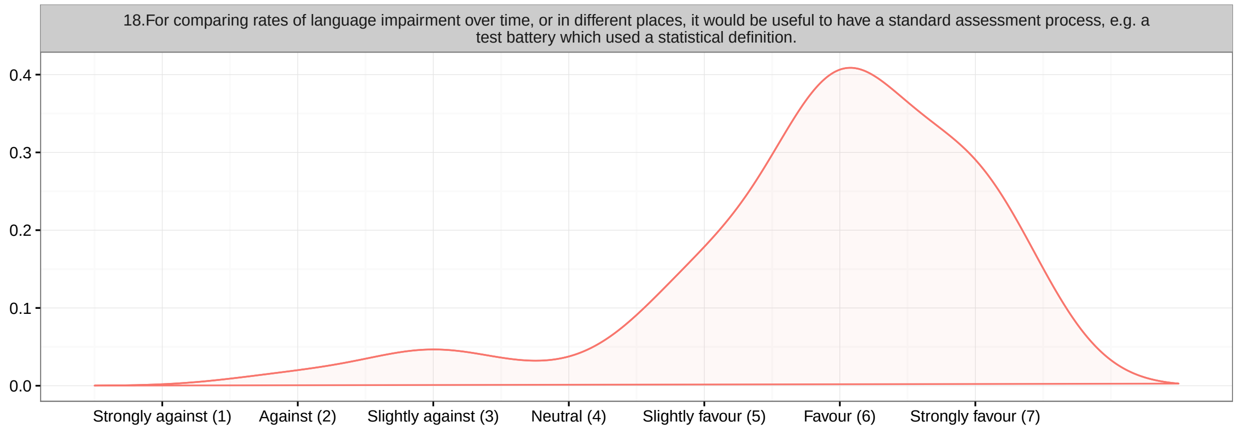

Figure 38: Distribution of responses to statement 18. The bold vertical line coloured red is Anonymous's response to this question for reference.

Table 18: Comments for each statement.

ResponseID	Q18B
$R_5cd8BDkYcGfGLKl$	In theory, yes, but you still have a problem about which norms you would use! But a more consistent approach to diagnosis would be welcome.
$R_2f9ctxaHBJuJdLD$	Percentile functioning compared with matched normal controls would be essential.
$R_4HGIGYFIvMxLWcJ$	this seems very useful – in theory – but is this achievable or even useful in practice, considering the evidence?
$R_0ofhSCmeppIQ8kt$	I wonder if this is attainable.
$R_6Dvhy7Alhw5wqIR$	to some extent this is what existing tests provide–those that are normed
$R_bwwc7dPFEcp1azH$	Standardising this definition can be problematic- in research a cut off of -1.5 or -1.25 SD below the mean is used, presumably to capture more children to include in studies, but in practice a cut off of -2SD below the mean is used- presumable to capture fewer needing services!
$R_2o7JoTNgC3lqSIR$	Though, of course, SLI does not necessarily present in the same way over time. Assessments would need to be sensitive to these changes.
$R_6LIAgEx6sspizpX$	However, children may progress functionally, emotionally, socially over time and this may be more important to them in the real world than statistical definition. Self-reporting alongside stats would give a better picture of the long term imapcts of language impairment.
$R_e5KJQmN6txthTRX$	However, language impairment can change over time, so any assessment would need to take this into account.
$R_2hLYvspULpn8NnL$	It would be; but a lot more research would be necessary to get there. And it would, of course, be culture-specific.

$R_6JOosydU46ZndMF$	HMMM... any mention of test batteries with statistical definition is doomed to failure for international epidemiological prevalence studies - issue sof traslation/back-translation, cultural sensitivities, availability of locally-normed tests etc...
$R_71b9fvukXBUQ5dr$	No other way. /
$R_1TXxdyLg1UFCx4V$	This depends on the purpose of the monitoring. If comparing a cohort of children against peers to establish a trajectory for a specific condition this might be helpful and indeed has revealed trends for children in the SLI literature. It should be made clear that this is not the same as measuring response to intervention. Still need to take into account my earlier point about adaptations for populations with additional developmental disorders
$R_dguQPTfUoDzSKB7$	depends what you mean by places. the cultural and linguistic varation may be too much to just have one test
$R_cYBwzqu4ivWh9qJ$	Alongside observations in settings and dynamic assessment. I know I am asking for the earth.
$R_6RlkuyWJYcIIsmN$	Agree but other clinical observations/rating tools are important too.
$R_cIxZunCo2wnTfVj$	Any such battery needs to include tests that are appropriate across age spans.
$R_834xbT3yZzu1O7z$	With careful consideration of what would be included in that battery.
$R_3rrKtkb2VvC3uG9$	this is a fundamentally fair way to identify children in need of services regardless of other mitigating circumstances. Note that if it were more widely used in clinical practice it could identify a common pitfall. Children can be identified for clinical services,enrolled on the basis of a standard score benchmarked to age expectations, put into a treatment plan with goals, and then dismissed from services because the goals were met, i.e., the child made change, but still be at the same position relative to age peers as at the beginning of services because the observed change did not keep up with the pace of age peers' change. the concern in the comments seems to be children with low scores might be be identified by others. it seems to be the scope of practice for professionals to identify the children and then advocate for services, given all that is known. this way girls and children with low SES would be more likely to receive services.
$R_5C49A94jWehNBB3$	This could combine several methods (standardized tests, caregiver ratings). .
$R_cLU7KRGW2XvEql7$	What would be the purpose of this? How would it benefit the child?
$R_23qAFVuJCo6YHOd$	I'm not sure about the statement and the purpose.
$R_8bIXFrv4VBlvVyZ$	but even for this purpose, other information is needed; Tomblin et al's work utilised standardised assessment but also reports from parents/others to confirm
$R_7WXquZJy8WlgXAx$	This would yield a fanastic ongoing indiccator of rates of LI which could be used to stimulate a case of support - but would have to be used with caution. it may not pick up some types of language impairment and there is a risk of under-reporting as well as over-reporting e.g. pragmatic difficulties. if the infromation yielded is used for planning services, this could be both helpful and unhelpful!
$R_6mrinfsu6CeSmBn$	Again, no use on the population—— which is predominately bilingual or EAL.
$R_9uJ5LinD5e8X5Yh$	We struggle to prove outcomes in an evidenced way eg robust data, from starting points. Trying to find our own assts which can cover 5 - 11 years, but a bit piecemeal and not comparable to children in other settings. If we had a test as you suggest and it was used for all SLI children we could then prove progress, outcomes and compare settings. Bring it on!
$R_cCuacCYZiqQHKgl$	It seems necessary that if we are going to count something, we need to count in the same way. This becomes really challenged by the fact that language isn't the same thing at different points in development. So our current solution is to measure a latent trait that has developmental continuity even though it is measured with different tasks and content at different times. The really big question concerns what it is that we mean when we say a child has a language disorder. Is it some inherent characteristic of the child that is stable or can resolve? Or is does it have to do with the person's ability to meet communication demands placed on the person. In the latter case, the disorder could emerge not because of changes in the person's ability but changes in the communication expectations. This might lead to a change in what we count and how we measure.
$R_e9cPjWuFpcer4B7$	Yes this would be useful but I suspect it would be highly time sensitive. Of course makers of standardised test batteries would probably suggest that this is precisely what they have done already. This key issue to to enhance item conscience across time.
$R_eLIdYhExxkQtUZn$	Although a standard assessment process or test battery would be useful in many contexts, issues relating to multilingualism and culture mean great caution is required when interpreting assessment data from some children.

$R_3DfMsLnqK54HqcZ$	Yes I think epidemiological work is essential and if we can create ways to look across cohorts this would be incredibly helpful for looking at prevalence, longitudinal stability and longitudinal risks

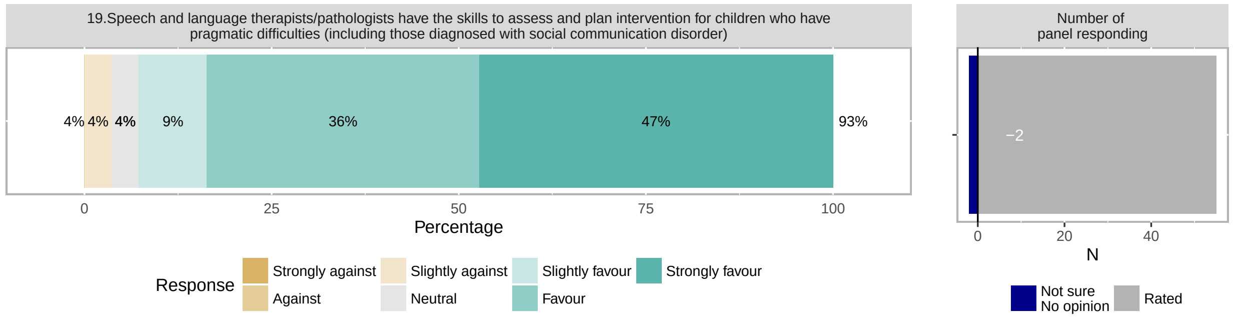

Figure 39: Percentage of panel members in each response category to statement 19. The percentages shown at each end of the scale are the cumulative percentages for the top and bottom three categories respectively.

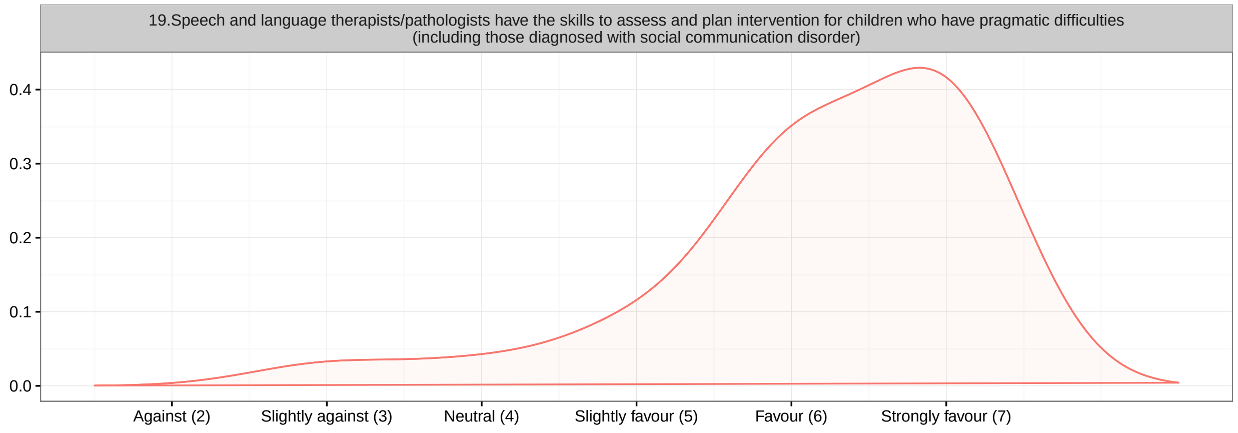

Figure 40: Distribution of responses to statement 19. The bold vertical line coloured red is Anonymous's response to this question for reference.

Table 19: Comments for each statement.

ResponseID	Q19B
$R_5uxk08XTwJpUk9D$	It is true for some SLPs but not for others!
$R_5cd8BDkYcGfGLKl$	They may not have the skills to make the differential diagnosis though, which involves assessment of restricted interests and repetitive behaviours and ruling out autism. Multidisciplinary team diagnosis would be very useful if pragmatic language difficulties are suspected.
$R_8AhxnQPe8mJkUoR$	There needs to be more training of course, as this aspect of SLT work is “younger” in years, so to speak, than speech, vocabulary or grammar for that matter (particularly inflections, although complex grammar not so sure). But yes, it is important to have an understanding of pragmatic difficulties to have a profile of the whole child.
$R_2f9ctxaHBJuJdLD$	Essential in a bronze standard ASD diagnostic pathway as well
$R_0Gj2hZlxlaPtHbT$	Best outcomes are i conjunction with clinical & educational psychologists
$R_6Dvhy7Alhw5wqIR$	not a strength of all however!
$R_bwwc7dPFEcp1azH$	Consideration of ASD should be given in all of these cases however, which may warrant referral to educational psychologist/ASD team. this could be considered following a period of intervention however.
$R_2o7JoTNgC3lqSIR$	They should have, but do they all? We have heard of therapists failing to recognise pragmatic impairments and services insisting it is not their job to provide intervention for children with these difficulties.
$R_6LIAgEx6sspizpX$	Yes in assessment (although not all seem confident that they can do this). However, the ability to plan and deliver effective intervention is in a fairly shocking mess in some instances.

$R_e5KJQmN6txthTRX$	Yes, but this needs to be acknowledged by services and commissioners, and therapists need the necessary skills. It is not unusual for children with significant pragmatic difficulties to be diagnosed with SLI (and not always given the most appropriate support) while children with structural language impairments are dismissed as having a 'language delay'.
$R_6JOosydU46ZndMF$	In theory perhaps, but unless UK training is far in advance of training in other countries, this aspect of language is still inadequately understood by SLPs. I believe that additional training will be necessary, particularly for experienced SLPs who have been in the field for a long time
$R_1TXxdyLg1UFCx4V$	This is an interesting statement and one that makes me uneasy. I agree we have the skills but the sentence may be taken to imply that others do not have them and that would be incorrect. If such a thing as pragmatic language impairment existed as an isolated disorder (and I do not believe this to be true) then others such as teachers, drama therapists, psychologists etc etc would also be equally able to assess and plan interventions to address the problem. This is not a trivial point. Often SLTs are asked to provide the social and pragmatic intervention for children with ASD, even those in specialist educational settings. I think this is poor practice since the development of a child's social competence is the remit of every adult working with children, especially those working with children with autism and it leads to two problems 1) the child may have a 'social skills session' with an SLT and the school think the job is done and 2) SLTs feel under huge pressure to be the person who is resolving any social difficulties.
$R_cYBwzqu4ivWh9qJ$	Especially if they have a special interest in this area. joint work with e.g. education staff would be extremely useful in this area e.g. specialist teachers, support staff, counsellors, behaviour and inclusion teams and social services
$R_cIxZunCo2wnTfVj$	SLTs generally should have assessment skills, but areoften less than well prepared re planning and / implementing intervention for pragmatics.
$R_3rrKtkb2VvC3uG9$	Again, there are mixed messages in the commentary. I support that speech/language pathologists are the professionals with the skill set to provide intervention for children with pragmatic language impairment, and also with the implication that SCD is poorly defined. I am not sure that any profession will know how to identify this new category with acceptable reliability and validity.
$R_5C49A94jWehNBB3$	But are presently poorly equipped to differentiate "the generic S(P)CD cluster" from overlapping symptoms with other psychiatric problems, cases of abuse, neglect, etc.
$R_23qAFVuJCo6YHOd$	Yes! There should be no doubt with this statement. ———-. SLTs have the expertise to assess, analyse, and provide intervention specific to the child.
$R_8bIXFrv4VBlvVyZ$	Yes they do, but it is a less developed area of practice-recent initiatives like production of SCIP manual (Adams et al) will help this but upskilling needed: I'm aware of services where this practice area is deemed to be the responsibility of therapists employed in CAMHS teams and not SLTs employed in wider primary care/community settings.
$R_7WXquZJy8WlgXAx$	Absolutely!
$R_9uJ5LinD5e8X5Yh$	They can look at and plan for language issues but need to work hand in glove with the specialist teacher to understand interventions needed for curric access.
$R_cCuacCYZiqQHKgl$	Perhaps I would say that SLT/Ps should have the ability to do these things and in many cases do.
$R_es7hPPlfD7bdd65$	Too broad to make a clear response to this. Maybe some of them do? My experience is many of them don't really agree on what language impairment looks like.
$R_e9cPjWuFpcer4B7$	Lots of different groups do this. Psychologists SLTs and educationist all need to be involved. They are all interested in these children although they often characterise them in different ways.

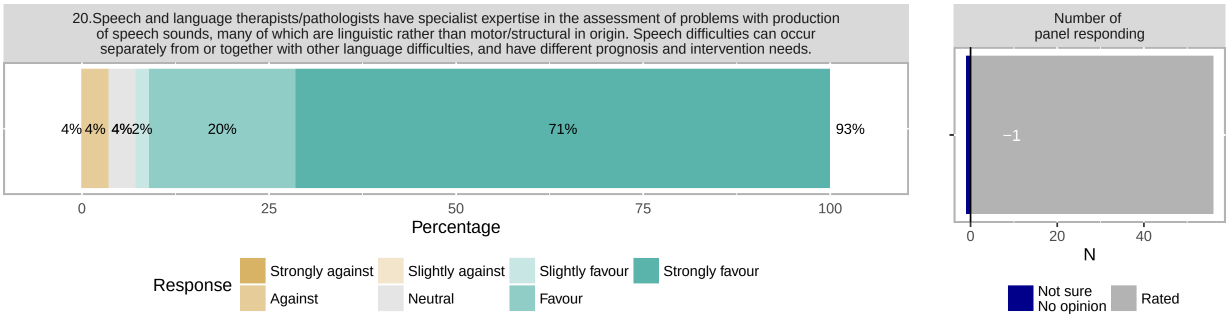

Figure 41: Percentage of panel members in each response category to statement 20. The percentages shown at each end of the scale are the cumulative percentages for the top and bottom three categories respectively.

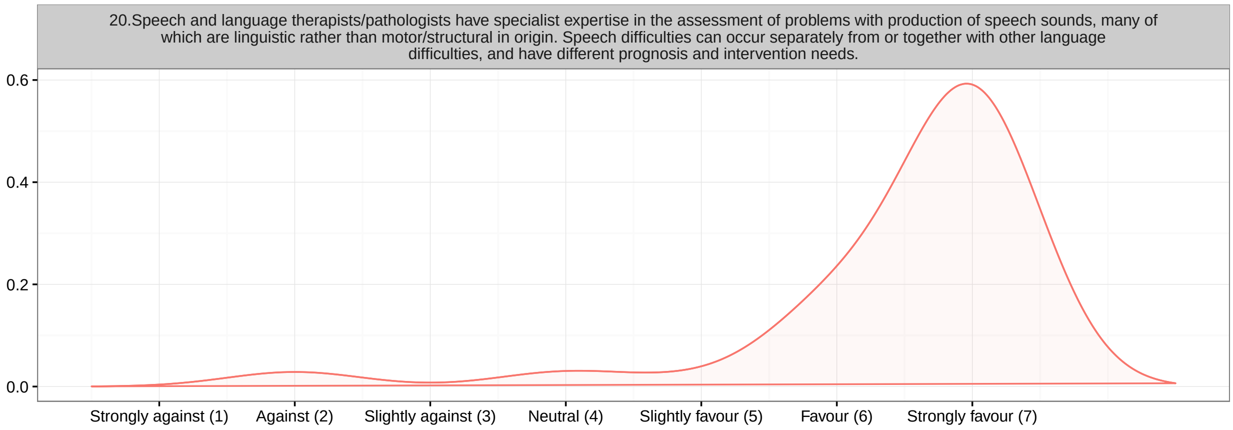

Figure 42: Distribution of responses to statement 20. The bold vertical line coloured red is Anonymous's response to this question for reference.

Table 20: Comments for each statement.

ResponseID	Q20B
$R_8AhxnQPe8mJkUoR$	Speech Sound disorders reference / Dodd, B. (2014). Differential Diagnosis of Pediatric Speech Sound Disorder. Current Developmental Disorders Reports, 1(3), 189 –196. /
$R_2o7JoTNgC3lqSIR$	Yes, or they should do. Again, individual therapists' skills will vary. In some cases, intervention for speech sound disorders requires highly specialist skills, and not all therapists will have them.
$R_6LIAgEx6sspizpX$	Yes! However, practice varies considerably across the UK with some areas using consultancy models, some don't see children for therapy until they go to school and others are delivering intervention from two or three
$R_e5KJQmN6txthTRX$	Again, therapists need the requisite specialist skills.
$R_6JOosydU46ZndMF$	don't know enough about this area to make a judgement
$R_71b9fvukXBUQ5dr$	whole other thing.
$R_6RlkuyWJYcIIsmN$	Agree but this is actually four propositions! Some respondents might have preferred these to be separated out :-)
$R_bOrkJKVQ6T8FeGp$	I note the literature was sparse for the rationale for this section and suggest this paper which is one of the few detailed community cohort based studies in the area and a far more contemporaneous reference to the current reference cited. / / Dev Med Child Neurol. 2015 Jun;57(6):578-84. doi: 10.1111/dmcn.12635. Epub 2014 Nov 18. / Speech sound disorder at 4 years: prevalence, comorbidities, and predictors in a community cohort of children. / Eadie P1, Morgan A1,2, Ukoumunne OC3, Ttofari Eecen K4, Wake M1,2,5, Reilly S1,2.

$R_3rrKtkb2VvC3uG9$	Absolutely, and it is good to see this item in the list. I suggest that the first questions about early development and early detection of language impairments be reconsidered given the considerable independence of speech impairment and language impairment in children and the over-reliance on early speech development as indicators of language impairments in young children.
$R_cLU7KRGW2XvEql7$	Of course
$R_23qAFVuJCo6YHOd$	I think it's important to note that phonological processing difficulties are a possible underlying factor in language difficulties (e.g. word storage/ morphology). Therefore it is not possible to separate where phonological difficulties persist. I think there are, however some phonological difficulties which are v specific and can be separated.
$R_8bIXFrv4VBlvVyZ$	Yes. This is important and future clinical decision aids/diagnostic criteria tools should reflect this. We have had the unfortunate scenario of children with speech sound disorders only, being inappropriately referred for specialist SLT and educational supports on the basis of the child's profile satisfying cut-off points on a standardised test for speech only, with no other language involvement or clinical markers. Based on discussion with personnel involved, it seems to be happening in order to gain access to more intensive forms of intervention than might be afforded in community services but has resulted in children being inappropriately placed in language classes or resolving soon after referral. so it's important to recognise children with isolated speech sound disorders, with no other language involvement, as a separate group who may be inappropriately placed in settings for specialist language intervention
$R_7WXquZJy8WlgXAx$	Very much so. I think this continues to e=be an area of confuision for non specialists where the visiable difficulty is a 'speech' problem, masking the underlying 'language difficulty'. SLTs role in idenitfying and supporting other to understand thisis crucial
$R_9uJ5LinD5e8X5Yh$	New SLTs don't have this body of knowledge in sufficient depth. Even where they can assess they often have no interventions appropriate to the child's profile..........think this is as a result of the more consultative role they are trained for and have?
$R_e9cPjWuFpcer4B7$	This I would say is an area of specialisation covered by SLTs but no other groups of professional.
$R_4ORQ8jYm1JwWwND$	Shriberg, L. D., Tomblin, J. B., & McSweeny, J. L. (1999) is certainly not sufficient evidence to support this claim.

2.3 Relation of language impairment to other developmental difficulties

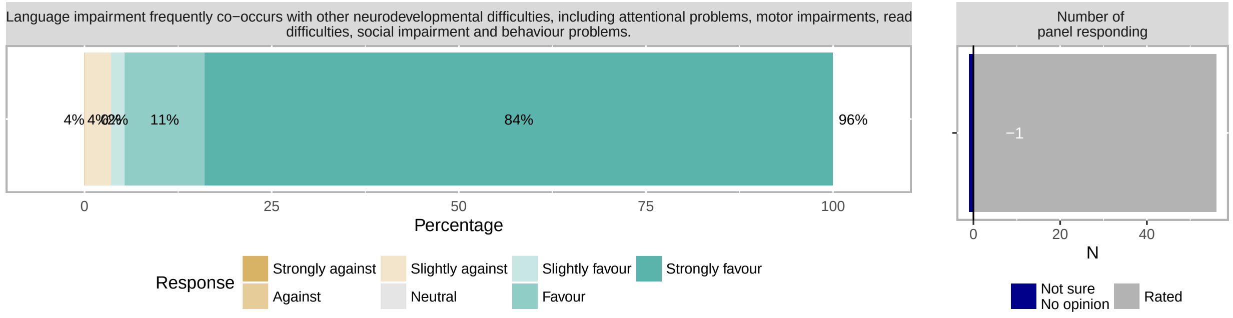

Figure 43: Percentage of panel members in each response category to statement 21. The percentages shown at each end of the scale are the cumulative percentages for the top and bottom three categories respectively.

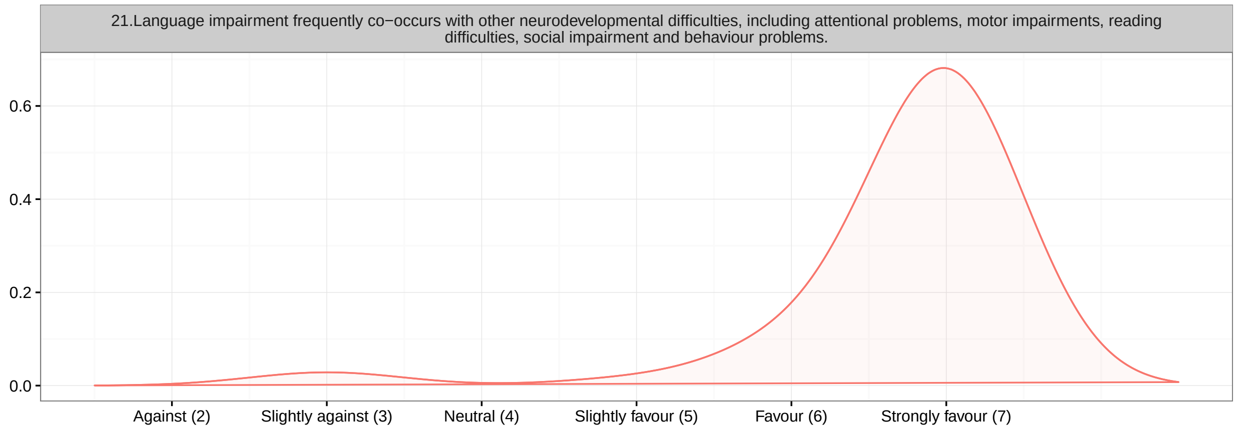

Figure 44: Distribution of responses to statement 21. The bold vertical line coloured red is Anonymous's response to this question for reference.

Table 21: Comments for each statement.

ResponseID	Q21B
$R_ba8iHG84IJ8cW7X$	I'm wary to compounding 'behaviour problems' and social 'impairment' with neuro- conceptualisations.
$R_5uxk08XTwJpUk9D$	We now know that this statement is true for many/most neurodevelopmental disorders.
$R_8AhxnQPe8mJkUoR$	Overlap LI and other difficulties / Mok, P. L., Pickles, A., Durkin, K., & Conti – Ramsden, G. (2014). Longitudinal trajectories of peer relations in children with specific language impairment. Journal of Child Psychology and Psychiatry, 55(5), 516-527. / St. Clair MC, Pickles A, Durkin K et al. (2011) A longitudinal study of behavioural, emotional and social difficulties in individuals with a history of specific language impairment (SLI). Journal of Communication Disorders, 44, 186 – 199. /
$R_2f9ctxaHBJuJdLD$	This has indeed been my experience over the last 20 years as a child psychiatrist.
$R_4HGIGYFIvMxLWcJ$	In reality these 'other difficulties' may sometimes be more noticeable than the underlying or co –occurring language impairment.
$R_bwwc7dPFEcp1azH$	Yes, I am in favour of approaches that use labels such as Language Impairment secondary to, or with concomitant motor/ reading/ behavioural difficulties et.
$R_2o7JoTNgC3lqSIR$	This is true, unfortunately in some ways, as it makes it much more difficult for us to advocate for recognition of SLI and appropriate intervention for affected children.
$R_e5KJQmN6txthTRX$	Unfortunately, this again militates against language impairments being taken seriously. All too often it is seen as part and parcel of being from socially disadvantaged backgrounds.

$R_6JOosydU46ZndMF$	not only co-occurs but often precedes, especially reading difficulties. Not only co-occurs with reading difficulties but also with arithmetic difficulties, especially word-problems, math reasoning etc in which it is often the specific arithmetic linguistic terms that give rise to problems
$R_5C49A94jWehNBB3$	The word "frequently" is problematic. It connotes "usually" or "ineveitably" which I think is an exaggeration of the evidence. From an epidemiological perspective, most children with LI do not present with clinically signficant levels of reading deficit or socioemotional behavioral disorder. However, I do think that children with co-occurring difficulties tend to get more services from SLPs and that children with problems in these areas that do not have LIs regularly appear on SLP caseloads.
$R_23qAFVuJCo6YHOd$	Also to note the potential influence of language difficulties on presenting attention, reading, social and behaviour difficulties.
$R_8bIXFrv4VBlvVyZ$	Yes based on my clinical experience and knowledge of the current research base, I concus that "comorbidy" is now more the rule than exception but the percentage who have language difficulties only are still relevant and still have needs
$R_1FT913eWSaeKlhP$	The prevalence of these co-occurring conditions varies therefore the extent to which I agree with the statement varies across these co-occurring difficulties.

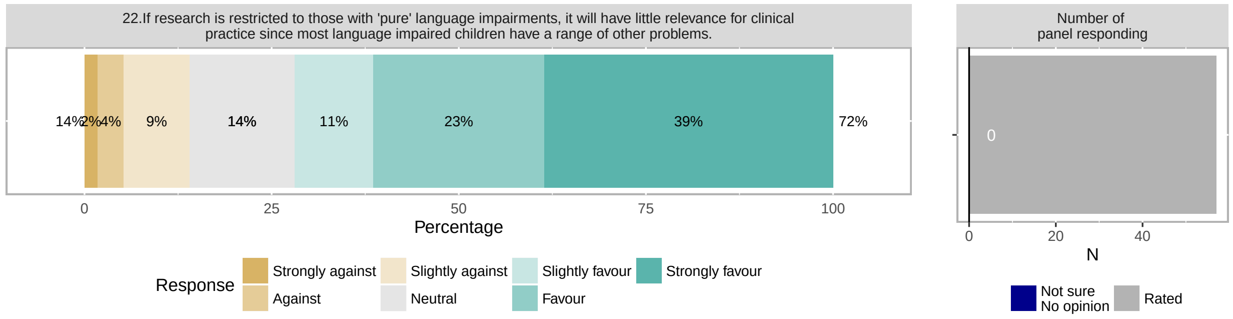

Figure 45: Percentage of panel members in each response category to statement 22. The percentages shown at each end of the scale are the cumulative percentages for the top and bottom three categories respectively.

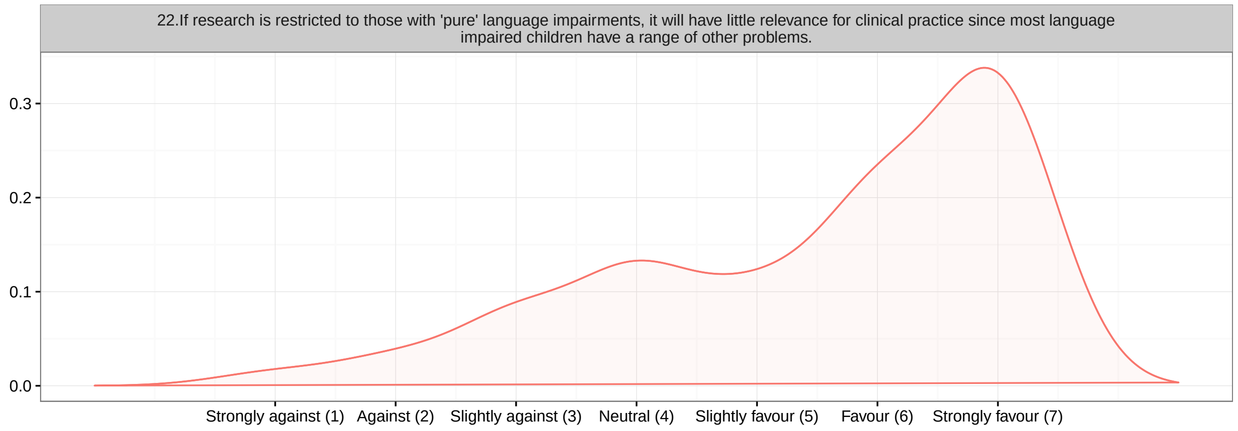

Figure 46: Distribution of responses to statement 22. The bold vertical line coloured red is Anonymous's response to this question for reference.

Table 22: Comments for each statement.

ResponseID	Q22B
$R_{7}8kR8ERViieBuyF$	"Other problems" do not usually reach the threshold of "other impairments" unless language impairment is being defined so broadly that it is useless.
$R_{5}uxk08XTwJpUk9D$	Few studies actually explicitly evaluate co-occurring conditions in children with LI; unless they do so the studies aren't ever just about the 'pure' condition. Certainly studies that are relevant to intervention should include (and evaluate) co-occurring conditions.
$R_{5}cd8BDkYcGfGLKl$	Such children would be extremely rare anyway!
$R_{2}f9ctxaHBJuJdLD$	Research has to include the whole range but in terms of simplification of study design it makes sense to try and isolate specific factors and interventions which may be otherwise confounding results.
$R_{4}HGIGYFIvMxLWcJ$	Agree, although research into pure impairments may be needed to advance our theoretical knowledge.
$R_{0}ofhSCmeppIQ8kt$	Both sides of the argument here are relevant – and so there is no clear solution. The restricted research focus will provide information for evidence –informed practice, one hopes.
$R_{6}Dvhy7Alhw5wqIR$	almost impossible to do since comorbidity so prevalent but researching profiles of purer conditions can speak to other situationsg
$R_{b}wwc7dPFEcp1azH$	Yes- researchers should clearly describe the children included in studies, and the range of other difficulties present or otherwise so that more realistic and clinically applicable conclusions can be made
$R_{2}o7JoTNgC3lqSIR$	I should think it would depend on the nature and purpose of the individual study. Some research might yield very helpful and useful insights; others might not.

$R_e5KJQmN6txthTRX$	It depends on the research. This might be true in some situations, but not in others. We do need more research relating to real life situations and good practice.
$R_2hLYvspULpn8NnL$	We need a range of studies, on both 'pure' and mixed disorders. We shouldn't be ruling out any kind of research, but should be increasing its diversity. One outcome of this exercise should be to alert journal editors and reviewers to the need to be a bit more lenient in including studies with somewhat 'messy' sets of participants, PROVIDED there is clear, strong characterization of participants presented in the the Methods.
$R_6JOosydU46ZndMF$	Yes, but the challenge is how to ascertain which aspects of communication problems are attributable to SLI versus the co-existing conditions; requires complex designs and/or very large samples
$R_71b9fvukXBUQ5dr$	But - may not be the aim of research, much of which is about language processing/modularity, not clinical practice.
$R_1TXxdyLg1UFCx4V$	I agree with the comments in the notes. The definition and specificity of groups will depend on the purpose of the research. Certainly we need to push for broader inclusion in intervention studies'
$R_cIxZunCo2wnTfVj$	Even so, this type of research is crucial to identify core deficits, and at least some aspects of intervention can be gleaned from this work.
$R_834xbT3yZzu1O7z$	It is essential that research reflects the cohort of children and young people that practitioners work with in everyday settings
$R_3rrKtkb2VvC3uG9$	The sequencing of items, and the commentary, implies that language impairments are essentially co-existent with other developmental disabilities. This is a very complex issue, that is somewhat obscured in the items here. It is arguable if reading, social, behavioral, attention problems are co-morbid or in a complex set of conditions that ride on the consequences of language impairment. Motor impairments have yet to be identified that consistently, and over time, serve as clinical markers of language impairment. The term "pure" language impairment seems to be a straw man, an idealized condition that could always be disconfirmed, in preinciple, by any one child presenting with a concomitant individual difference of another sort, maybe too tall? there is a history of debates about "pure" language impairment that somehow set the entire field off on a tangent.
$R_5C49A94jWehNBB3$	This is another loaded statement based on several objectionable presuppositions. I'm not sure what "little relevance" is intended to mean in this context but it sounds very dismissive of decades of productive scientific work. We seemed to have learned a lot of clinically relevant information by studying SLI (e.g. language impairment is heritable). Also, I don't think anyone has ever adovcated for restricting research to only cases of "pure" LI. I agree that a lot of clinically useful information could be learned from cross-etiology comparisons as well as examining the profiles of cases of comorbidity. But this cuts both ways. If we want to open up inquiry of LI to include other problem areas (e.g. attention deficits) then we are obligated to expand our comparison groups in a symmetrical fashion. For example, we should include comparison groups of cases of ADHD that do not have language impairments. Otherwise, associations based on sampling errors will be misintepreted as potentially causal. As a result, precious resources are wasted chasing down false leads. Most of these reported "soft signs" of sub-clinical weaknesses have been based on comparisons of affected groups to groups of "typically developing" children that are in fact not very typical at all but rather tend to perform in the above-average/gifted range and come from advantaged backgrounds.
$R_5ceQk7pgvAecMAt$	As stated in the background, there may be particular studies where a "pure" approach is a worthwhile step in a larger research plan, but generally excluding children with co-morbidity excludes the majority of the clinical population.
$R_cLU7KRGW2XvEql7$	what is this question about? who would do the restricting of research? Surely if research proposals and funding etc arise from the children we work with (as opposed to based on theory) then this will never happen, so the question is irrelevant?
$R_eOEFfbvY55KRtRP$	I agree but having experienced language impairments from both a research and a clinical angle, there is no real difference between 'pure' impairment and others. Research protocols tend to 'tidy' up experimental groups or under report additional difficulty, as well as often excluding children with very severe problems and this needs to be more clearly reported and recognised.
$R_23qAFVuJCo6YHOd$	However, it will be necessary to distinguish between language impairment and those children with delayed language due to HI for example.

$R_8bIXFrv4VBlvVyZ$	I believe there is always a place for research that compares groups that have specific characteristics and we have learned from studies of children with purer language impairments; this is not a question of either/or, we need both types of research
$R_3VHaciSzwJGKIU5$	this also assumes one can identify children with 'pure language impairments'. Rarely do researchers have access to information to rule out ALL other associated difficulties.
$R_6JZKVRyNZK6U0zX$	The answer to this depends on the reason the study is being conducted. Research on children with SLI and research on those with language impairments 'secondary' to other developmental/neurological impairments will both continue to be needed. It is clear, though, that more research is needed on the latter since that heterogeneous group represents a large portion of children seen by clinicians.
$R_6mrinfsu6CeSmBn$	I woudl suggest that research needs to start with a group for which additional needs are not present, then if results are significant we should rolol out the research to children with additional needs.
$R_9uJ5LinD5e8X5Yh$	Often researchers exclude the population of more complex profiles - the very ones where we need to isolate the language impairment to see it in its purity and then consider the impact of associated/additional difficulties. They deal with the icing but we have the cake as well to support
$R_cCuacCYZiqQHKgl$	I don't think the research is irrelevant and certainly a case can be made for the value of insights coming from "pure cases." This kind of research may need to rationalized, just as it has been necessary to justify doing research with complex cases.
$R_es7hPPlfD7bdd65$	I don't think researchers are very good at finding "pure" LI kids - at best they'll run a quick NVIQ task. Like, when's the last time you read an SLI study where they even looked for comorbid reading impairments?
$R_6tiOrhFOdV4NANf$	We need research that examines children with 'pure' language impairments and research focused on clinical populations more generally. It depends on the purpose/research questions that are being examined.
$R_4ORQ8jYm1JwWwND$	Even when research exludes children with other frank neurodevelopmental disorders, the partiicpants often exhibit subtle difficulties in other aresas like attention and motor ablities.
$R_3sXNbQYRlZaMb3L$	Same applies to development of services and criteria for support (speech and language therapy; SEN support; S&L bases and resources).
$R_3DfMsLnqK54HqcZ$	As I mentioned in previous stage of this process I think in certain experimental studies researchers may want to add additional inclusion/exclusion criteria to those used clinically and or subgrouping analyses but for intervention, identification, prevalence research then broader but clear definitions and descriptions of populations would be very helpful to make research more relevant. Also it would force researchers to acknowledge and be explicit about the nature of their samples.

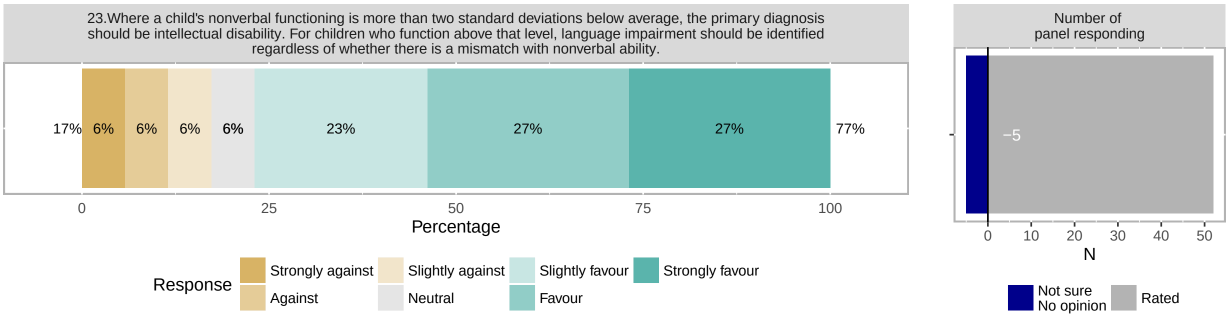

Figure 47: Percentage of panel members in each response category to statement 23. The percentages shown at each end of the scale are the cumulative percentages for the top and bottom three categories respectively.

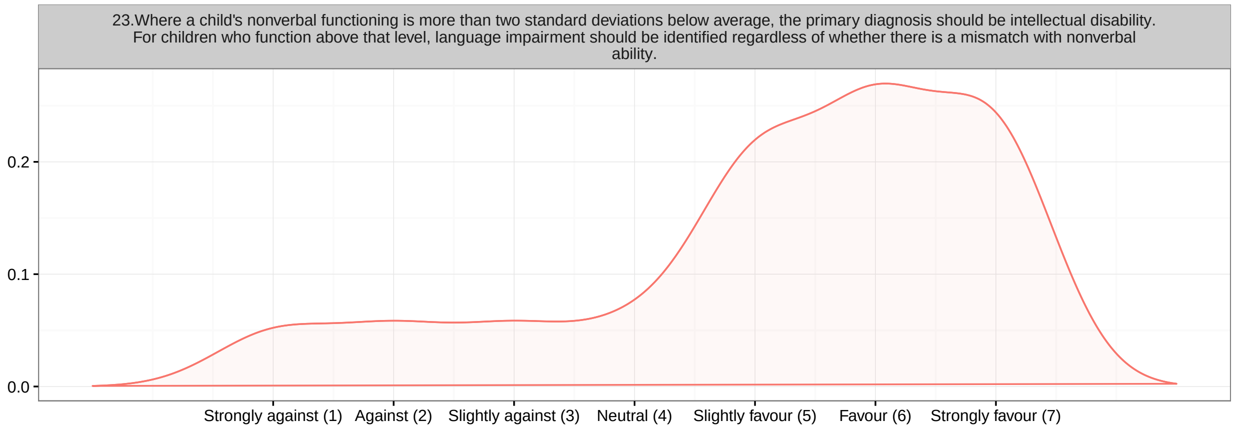

Figure 48: Distribution of responses to statement 23. The bold vertical line coloured red is Anonymous's response to this question for reference.

Table 23: Comments for each statement.

ResponseID	Q23B
$R_b a8iHG84IJ8cW7X$	It seems to me that attention to a child's communicational competence should be given irrespective of other aspects of functioning.
$R_5 uxk08XTwJpUk9D$	Even in the context of frank ID a child should also be considered as LI from the perspective of assessing their language problems and offering specific interventions to address their clinical needs.
$R_5 cd8BDkYcGfGLKl$	—removed comment as too identifying—'
$R_5 cKMfR48zQytYc5$	i think this is very complex and needs a little more unpacking - I'm not certain about this - i don't think cut point here are helpful.
$R_2 f9ctxaHBJuJdLD$	What about NVLD profiles and ID is supposed to be a global issue so this does not make sense clinically.
$R_4 HGIGYFIvMxLWcJ$	Agree, although I am not completely comfortable with the 2SD cutoff.
$R_0 ofhSCmeppIQ8kt$	I recognize the need for differential diagnosis but the use of cutoffs is uncomfortable.
$R_0 Gj2hZlxlaPtHbT$	Even with amongst children who function above 2SD there should be a sub – dimensional or categorical demarcation between those with normal & abnormal non-verbal (who tend to have more neurodevelopmental & social pragmatic features)
$R_6 Dvhy7Alhw5wqIR$	but the language/communication needs of the child with ID do need to be assessed
$R_b wwc7dPFEcp1azH$	Again diagnosis based purely on a response to a standardised test is not favourable to me

$R_2o7JoTNgC3lqSIR$	This question is poorly worded. I presume the first part refers to a child with nonverbal and verbal scores below 2 SD below the average, with the verbal scores considerably below the non-verbal? If so, I would agree that intellectual disability should be the primary diagnosis (provided that we are satisfied that this reading of the situation is accurate - poor verbal abilities can depress non-verbal scores). The second scenario is very confusing. Read one way it could suggest that anyone and everyone could be diagnosed with a language impairment, but presumably this is not what you're meaning. I agree that relying on scores alone is rather an arbitrary judgment. It would be better if we could say a child with certain characteristics definitely has a language impairment, but I'm not sure we can do this at the moment, or not always. So in effect, the discrepancy is basically all we can rely on. The problem is that if we ignore discrepancies, is there any difference at all between children with language impairments and those with mild/moderate learning difficulties. If not, we fear that there would be an increased reluctance to spend money on high levels of SLT for affected children.
$R_6LIAgEx6sspizpX$	Still unsure about this...
$R_e5KJQmN6txthTRX$	Conflating three items has made this question really quite confusing. The discrepancy, and exclusionary, model has always been important in terms of making the case for specific intervention and provision. If we lose that, there is a risk that children with significant language impairments will get no more help than children with learning difficulties, meaning that the condition might disappear entirely.
$R_2hLYvspULpn8NnL$	Yes, but this should not preclude a diagnosis of comorbid LI in those with NVIQ<70.
$R_6JOosydU46ZndMF$	Intellectual disability (ID) must not be based solely on non-verbal functioning (aka performance IQ etc); requires evidence of poor adaptive behavior as well as low IQ (albeit non-verbal IQ). SLPs must have a solid understanding of current conceptualization of ID. see DSM-5, Intellectual disability as a disorder of reasoning and judgement: the gradual move away from intelligence quotient-ceilings. Greenspan S, Woods GW. Curr Opin Psychiatry. 2014 Mar;27(2):110-6.
$R_71b9fvukXBUQ5dr$	And what 'match' means given test error needs to be further understood in practice.
$R_1TXxdyLg1UFCx4V$	This implies that language impairment cannot exist in individuals with IQ below 70 which is borderline to mild LD and I do not think this is the case. Language impairment should be identified in any individual independent of NV ability
$R_dguQPTfUoDzSKB7$	cognitive referencing is so out of date and unproven to lead to any good practical outcome.
$R_cYBwzqu4ivWh9qJ$	I can't agree with a two tier system especially if it alters access to services and resources. In this instance what are you diagnosing for?
$R_834xbT3yZzu1O7z$	I think this is a really tricky one to answer - i don't agree that there is a cut off, as a child will have a language impairment if they are above or below the 2SD cut off - we will still need to identify and describe that language impairment. But when you talk about PRIMARY diagnosis, non verbal ability may be taken into account.
$R_3rrKtkb2VvC3uG9$	Why do we have to throw the intellectually limited kids off the bus? Is this a fuss about "primary" vs "secondary" or an echo of the DSM-5 and other such nosologies driven by medical practice? Again, this is another item that points in different directions across the components. I strongly favor a position that says that all children with language impairments, regardless of mismatch with nonverbal ability, should be so diagnosed, i.e., as having language impairments. I realize this would be inconsistent with the exclusionary conditions invoked by DMS-5. I also note that under this exclusion many of the children unjustly confined in hopeless residential facilities for people with intellectual disabilities for decades and decades would never have gotten out and received valuable services to meet their communication needs. This item threatens to take us back to the bad old days.
$R_5C49A94jWehNBB3$	Notice, this would include the segment of the population that used to be referred to by school officials as "mild/borderline mentally retarded" that then swelled the ranks of the "learning disabled" when the MR/ID eligibility criteria was pulled down to -2.0 SDs... not a trivial number.
$R_eOEFfbvY55KRtRP$	The first statement I agree with - but it does not preclude the possibility of a co-occurring language or speech impairment, even at a very low level of functioning. The important factor would be what the child's potential is to respond to intervention and facilitation of language/speech. I agree with the second statement. /

$R_eG1jl51DiHRqXKB$	This is hard to answer because a child could have a nonverbal learning disability. The statement above does not allow for the possibility of very low nonverba functioning is the context of adequate language functioning.
$R_8bIXFrv4VBlvVyZ$	yes but we need more research that includes children across levels of non-verbal ability, and carefully describes different cognitive characteristics
$R_6JZKVRyNZK6U0zX$	Difficult to answer, since there is not a strong evidence base yet for the most appropriate cut-off.
$R_7WXquZJy8WlgXAx$	I find cut off points really difficult! My approach has always been to profile a child's language skills, idenitfying areas for in-depth investigation. these isolated scores may provide some indication of level of functioning, but without additional information it is difficult to make absolute decisions/diagnosis
$R_1QTm7VrpDX1OAi9$	However, I do think that a child with a primary diagnosis of intellectual disability should also be able to have a secondary diagnosis of language impairment
$R_9uJ5LinD5e8X5Yh$	We assess for an unexplained gap between learning age and language age. Therein lies the hub of the SLI. If the learning age (on non verbal cog skills assts eg Kaufmann) is the same level as the lang age then this is a 'flat liner' ie SLD or MLD. If the gap is significant then there is cause to investigate for SLI
$R_cCuacCYZiqQHKgl$	Unfortunately, there is no bright line between these categories either. I have a sense that research in ID is going to move toward distinguishing between ID resulting from major genes and chromosomal abnormalities and those that are idiopathic (perhaps multifactorial). Probably, these idiopathic cases may be the low end of the language disorder continuum.
$R_es7hPPlfD7bdd65$	OK I agree. But the presence of language impairment should not get buried in the intellectual disability diagnosis.
$R_e9cPjWuFpcer4B7$	These criteria are useful but of course many EPs do not use such tests and we often don't really know whether a child falls above or below a specific criteria. The minus 2 SD does not have any educational relevance nowadays. We don't know whether intervention is sensitive to this sort of criterion. In fact studies such as Boyle et al would suggest that it may not be. That said the principal is probably sound.

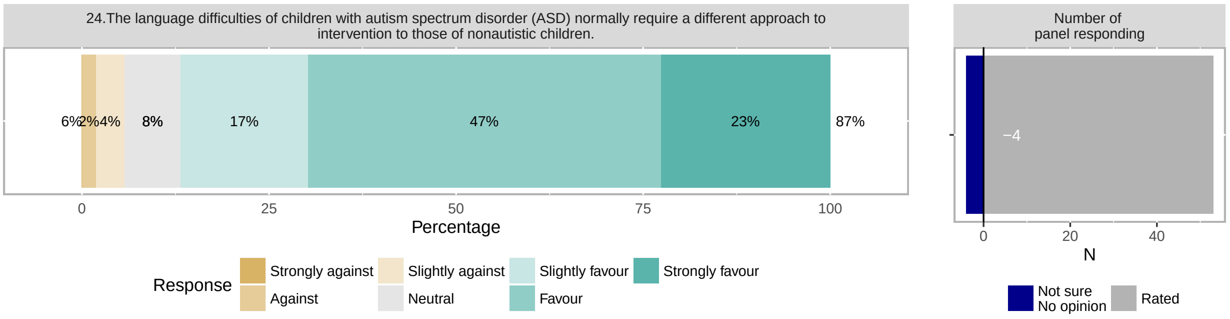

Figure 49: Percentage of panel members in each response category to statement 24. The percentages shown at each end of the scale are the cumulative percentages for the top and bottom three categories respectively.

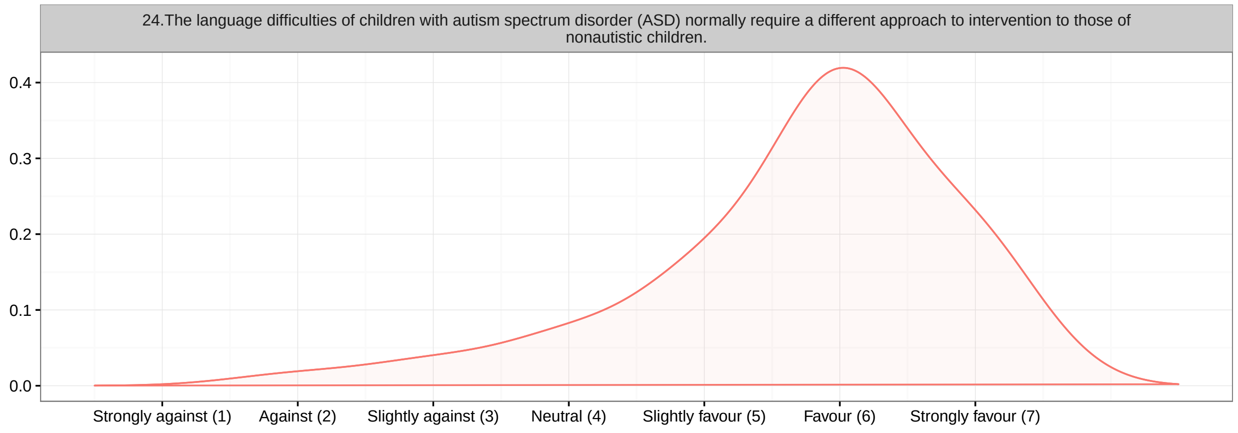

Figure 50: Distribution of responses to statement 24. The bold vertical line coloured red is Anonymous's response to this question for reference.

Table 24: Comments for each statement.

ResponseID	Q24B
$R_5uxk08XTwJpUk9D$	I disagree with the source of this statement - I don't think we know whether the language deficits in LI and ASD are the same or different (the evidence is more mixed than presented in the background document). Either way, the problems in language show up in the same areas of impairment. Nevertheless, I voted in 'favour' because of the term "approach" - most children with ASD will do better with speech therapy that is delivered in a more highly structured 'ABA' format, which would not be needed by children with LI.
$R_5cd8BDkYcGfGLKl$	There is no evidence that this is the case. The concern is that all children with ASD diagnosis are prioritised for 'social skills' training and their language needs are not addressed. It is clear that some children with ASD have language impairments that are not directly caused or associated with their social difficulties. They may need additional support for ASD, which of course may impact learning, but we have no idea whether improving core language skills requires a fundamentally different approach.
$R_8AhxnQPe8mJkUoR$	I would like to note though that does not mean that approaches used in autism cannot be used with children with LI. / / The key difference I see is the rate at which change is observed. Developmentally rate of progress in targeted areas is protracted in ASD as compared with LI.
$R_2f9ctxaHBJuJdLD$	Most interventions arising in conjunction with ASD require modification due to the ASD clinically speaking- eg modified CBT – intelligent differentiation as well as different aetiologies.
$R_0ofhSCmeppIQ8kt$	This might just as much because of the other characteristics that go along with ASD than the language impairment itself.

$R_bQ13TaeUPFsxVJP$	While the background literature suggests that the causes of language difficulty may be different in the two groups, in future research, it is important to understand whether different intervention strategies are necessary for these groups.
$R_6Dvhy7Alhw5wqIR$	they may do and usually do do but not exclusively / this question is oversimplified, individuals are much more nuanced–depends on the severity of ASD I see continua of asd and language impairment as well as the impact of ASD on language
$R_bwwc7dPFEcp1azH$	I'm not sure if we have sufficient evidence yes as to how successful interventions targeted at children with ASD are yet- they often benefit from similar interventions to other groups such as parent-training, visually mediated interventions through symbols etc. and the principles underlying behavioural interventions apply to others (although not the same intensity as applied to children with ASD). I think we need to see more of the evidence base emerging
$R_2o7JoTNgC3lqSIR$	Yes, to some extent, but there is little practical difference between many children with ASD and those with pragmatic language impairments, for example. And children with ASD may need intervention for additional speech/language impairments, and some children with language impairments (especially older ones) may need support to develop their pragmatic skills.
$R_6LIAgEx6sspizpX$	Often - but we do see some children with ASD who present with language needs requiring work e.g. on syntax
$R_e5KJQmN6txthTRX$	Up to a point yes. There is a clear difference between the typical language profiles of children with SLI and ASD, but there can also be clear overlaps. Some ASD children have additional structural language difficulties, and some SLI children have, or develop social interaction difficulties.
$R_2hLYvspULpn8NnL$	Yes, some of the techniques devised for children with LI may be less effective for those with ASD, but some of the techniques developed for ASD may be useful for those with LI, particularly at the severe end of the continuum or for children with LI AND deficits in attention, behavior and executive function
$R_6JOosydU46ZndMF$	Superficially this statement seems OK (if this is the case) but it is uninformative - would be better to explain why or add example of type of approach that is needed
$R_3pDedyU4fM1kOXj$	There will be overlap of interventions which may be helpful to both but generally speaking children with ASD require a different approach.
$R_71b9fvukXBUQ5dr$	If you include pragmatic factors within language.
$R_1TXxdyLg1UFCx4V$	I don't think this statement is well framed. The 'language difficulties' may not need a different approach, however the child may need a different sort of approach which might be a modification such as using more pictures or providing additional steps in the progression of tasks.
$R_cYBwzqu4ivWh9qJ$	With the stress on the word 'normally', I have certainly taught exceptions to this rule.
$R_834xbT3yZzu1O7z$	some aspects may be the same, but there will be elements that are different
$R_3rrKtkb2VvC3uG9$	Again, the term "language difficulties" masks the dimensionality of language, which is an issue underlying the item. If the "language difficulties" are pragmatic in nature, then the answer is more obviously in favor. If the "language difficulties" are "more structural" (by the way, is there some ban on the term "grammar" in this inventory? –I assume "structural" in various places in the commentary means "grammar") then the answer is more equivocal.
$R_23qAFVuJCo6YHOd$	I can't answer this- are we talking about the same type of language issues? And what do we mean by approach?
$R_8bIXFrv4VBlvVyZ$	the key word here is "normally", so there are some children with ASD who will have structural difficulties that are very similar to those of a child with language impairment who do not have ASD
$R_7WXquZJy8WlgXAx$	I think there is a hige overlap. Longstanding personal clinical experience shows that many of the approaches traditionally used with children with ASD are helpful for those with LI. There is no doubt, however, that for some children with ASD their language difficulties respond to specific approachs which take into account the nature of their cognitve structure and strengths/weaknesses. knowledge of these strengths and weaknesses, as with all children, inform intervention
$R_6mrinfsu6CeSmBn$	There does seem to be a group of children with ASD (high functioning) who have additional difficulties learning the structure and content of language and who need explicit teaching in these area before developing age appropriate skills. I woudl consider thse chidlren to have some degree of Language Impairment on top of their ASD.

$R_9U2zxMLVAPcvQUd$	I believe there are two forms of communication impairment in ASD. First, some but not all children with ASD have a co-occurring LI. This view is consistent with the removal of specific problems with language development from the core diagnostic symptoms and inclusion of with and without LI specifiers in the DSM-V. Second, all children with ASD have, by definition, some form of social communication impairment. With respect to those with ASD+LI, I am not convinced that we have compelling evidence that the language difficulties associated with the +LI piece "require a different approach to intervention" and the references provided in the background document do not speak to intervention efficacy. Certainly the core social communication impairments are unique and may benefit from different intervention approaches (but it could be argued that this part of their problems isn't best captured by the term "LI" anyway as opposed to manifestations of ASD in the communication realm). I think this item needs a more nuanced approach.
$R_9uJ5LinD5e8X5Yh$	Some overlap but some differences - seen particularly in the teaching arena (transference of knowledge, problem solving, literal interpretations, huge vocab knowledge but poor comp. I could wax on here for ages but won't - sighs of relief!
$R_cCuacCYZiqQHKgl$	Children with ASD are likely to have different needs and therefore different treatment objectives. Whether the basic principles of intervention need to be different is not clear to me. It is possible that these children do need a different kind of pedagogy such as ABA or a strong focus on social cognition
$R_e9cPjWuFpcer4B7$	If we assume that delivery would be different this would be true. Similarly one would probably adopt a functional rather than developmental approach. That said the targets might well be the same.
$R_eLIdYhExxkQtUZn$	Some of the intervention approaches used for children with ASD are useful for those who have language impairment without ASD and vice versa, particularly when considering children who are close to the margins of ASD but do not fulfill the criteria for a diagnosis.
$R_3sXNbQYRlZaMb3L$	Would like more clarity over whether this means completely different, or some overlap with additional approaches needed.

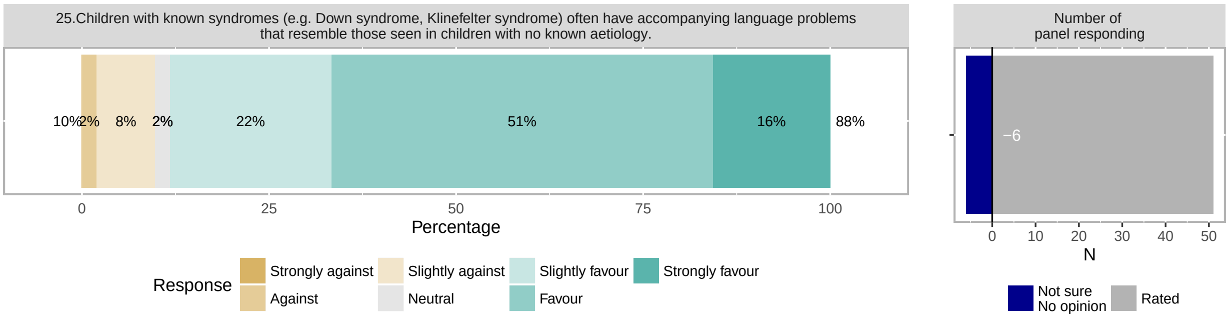

Figure 51: Percentage of panel members in each response category to statement 25. The percentages shown at each end of the scale are the cumulative percentages for the top and bottom three categories respectively.

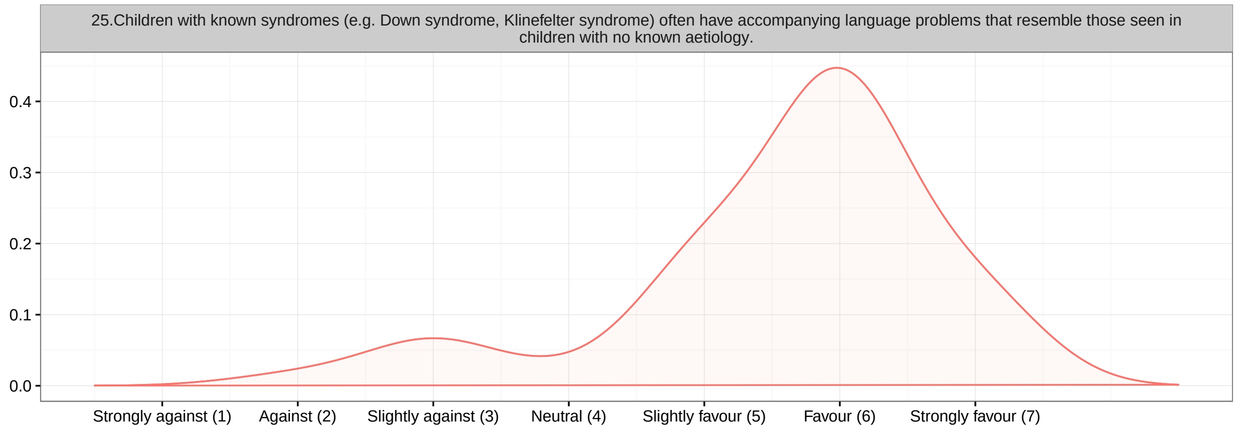

Figure 52: Distribution of responses to statement 25. The bold vertical line coloured red is Anonymous's response to this question for reference.

Table 25: Comments for each statement.

ResponseID	Q25B
$R_5cKMfR48zQytYc5$	phenotypes can however differ
$R_bQ13TaeUPFsxVJP$	However, these children already have clear diagnoses. To allow them to access the appropriate services, their language status could be appended to the existing diagnosis. It could muddle the public perception of language impairment of unknown aetiology if these groups were given diagnoses of language impairment alone.
$R_0Gj2hZlxlaPtHbT$	However a number of these children do present with a higher rate of ASD fxs, but generally the 'typical child' with an additional syndrome mirrors the language pattern of those of unknown with etiology at a significantly higher level of need
$R_bwwc7dPFEcp1azH$	Yes the language feature of SLI and DS have been shown to overlap
$R_6LIAgEx6sspizpX$	Not always- much of the advice/intervetion can be the same
$R_2hLYvspULpn8NnL$	They resemble LI, but may have somewhat different profiles, which are important for clinicians to know and look for (Paul & Norbury, 2012)
$R_6JOosydU46ZndMF$	Hmmm, in my experience with preschoolers with DOwn syndrome, the major difference appeared to be in gaze development, referential communication - but here the statement refers only to language, which i think is lisleading. Even if the langusge problems of these kids are somewhat similar to those with no known etiology, differences in other aspects of communication and in cognitive functioing, motoric tone etc may necessitate inclusion of different approaches
$R_3pDedyU4fM1kOXj$	The children with known syndromes may show different rates of process in comparison to children with no known aetiology.

$R_{7}1b9fvukXBUQ5dr$	And some that don't - non-fluency in Down syndrome for example.
$R_{1}TXxdyLg1UFCx4V$	I think this is essentially the same as the point made in no 24
$R_{c}YBwzqu4ivWh9qJ$	I have no background in this
$R_{8}34xbT3yZzu1O7z$	I have little experience in this field, but based on what I do have there may be some similarities, but i don;t think i would say 'often'
$R_{3}rrKtkb2VvC3uG9$	All children with language impairments should be eligible for language intervention to improve their communication skills. Without evidence to the contrary, the assumption would be that in general the intervention methods would not differ although the pace might be adjusted. Prognosis would assume positive change under competent professional guidance. Individual differences can be extreme in these clinical syndromes, which should be noted.
$R_{5}C49A94jWehNBB3$	.... but not necessarily.
$R_{8}bIXFrv4VBlvVyZ$	yes for example structural features of language in children with DS; but we need further and more in-depth studies of language problems across groups
$R_{1}QTm7VrpDX1OAi9$	This is my understanding, but I do not know the literature well.
$R_{c}CuacCYZiqQHKgl$	I think it depends on how strongly the resemblance needs to be. There seems to be good evidence that Down Syndrome, Williams Syndrome and perhaps Fragile X have somewhat different profiles in comparison with each other and typically developing children. These profile differences are most evident between speech, language and pragmatics. Within language itself, there seems to be more similarity than differences.
$R_{e}9cPjWuFpcer4B7$	There may be specific aspects of their profile which may differ but this is broadly true.

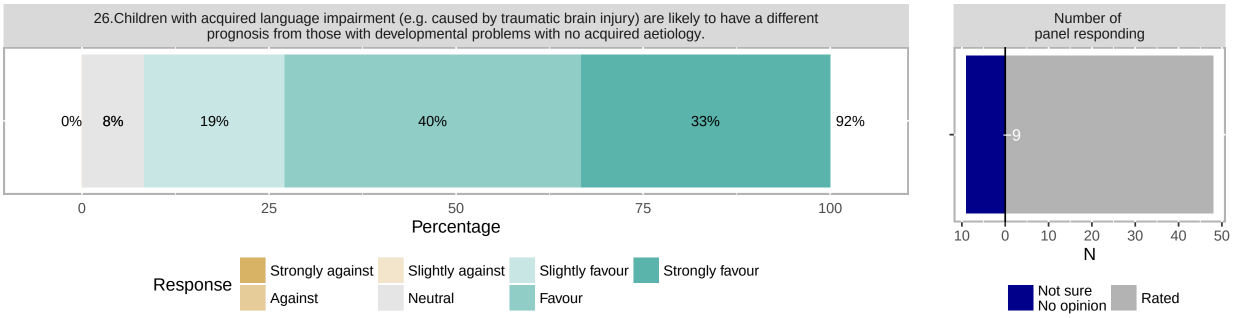

Figure 53: Percentage of panel members in each response category to statement 26. The percentages shown at each end of the scale are the cumulative percentages for the top and bottom three categories respectively.

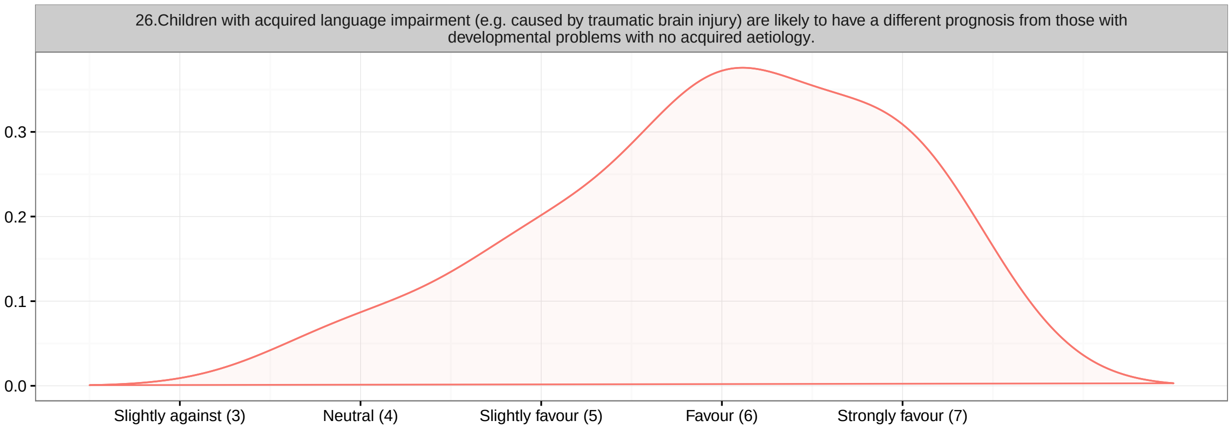

Figure 54: Distribution of responses to statement 26. The bold vertical line coloured red is Anonymous's response to this question for reference.

Table 26: Comments for each statement.

ResponseID	Q26B
$R_5cKMfR48zQytYc5$	again I am not totally sure exactly what this statement is getting at nor how important it is.
$R_2f9ctxaHBJuJdLD$	The whole ABI literature indicates that for example ADHD is much harder to manage and treat but the longitidinal trajectories of brain maturation can lead to significant improvements later in adulthood.
$R_4HGIGYFIvMxLWcJ$	Has there been any recent research in this area?
$R_0Gj2hZlxlaPtHbT$	highly variable and dependent on degree and areas of injury
$R_bwwc7dPFEcp1azH$	generally these children are younger, with no original underlying neurological condition and so plasticity can come into play for these children making their prognosis difference, especially in the early stages after their TBI
$R_6LIAgEx6sspizpX$	Wider range of cognitive/ behavioural /learning needs
$R_6JOosydU46ZndMF$	depends on pre-injury status and age-at-injury
$R_71b9fvukXBUQ5dr$	Different 'recovery'.
$R_1TXxdyLg1UFCx4V$	It is interesting that this point refers specifically to prognosis and not a different teaching approach. In my view this point and the two above relate to the correct identification of a language problem in a child with a comorbid condition or different aetiology and the necessary adaptations that need to be made in both treatment and expected outcome, do we want to specify each and every possible situation e.g epilepsy, physical illness etc?

$R_3rrKtkb2VvC3uG9$	Yes, the prognosis is better for children with acquired language impairment than for the classic SLI form of language impairment, according to work by Bates, Thal, Wolfeck, and others in that group. This does not mean that languae intervention should not be provided for children with acquired language impairments/
$R_1z8h1XMT676UOwd$	Difficult to generalise due to the wide variation in TBI effects.
$R_23qAFVuJCo6YHOd$	I don't know the evidence on this nor do I have much experience. However, I think this may depend on the age of the acquired impairment.
$R_8bIXFrv4VBlvVyZ$	yes, might need to be qualified depending on age when brain injury occurred and allow for possibility of pre-morbid language impairment
$R_1QTm7VrpDX1OAi9$	This is my understanding, but I do not know the literature well.
$R_9U2zxMLVAPcvQUd$	Vu, J. A., Babikian, T., & Asarnow, R. F. (2011). Academic and language outcomes in children after traumatic brain injury: A meta-analysis. Exceptional Children, 77(3), 263-281.
$R_e9cPjWuFpcer4B7$	It depends where the lesions following the brain injury were. Frontal lobe damage could well lead to more pragmatic difficulties lability etc which would be likely to lead to pronounced long term effects. It also depends on the age of the child. The younger the child when they they have they trauma the more likely they are to recover to some extent. So we need to be much more specific in this thought experiment. If we take two notionally identical children at say eight years their long term profiles are likely to be similar when it comes to their language skills.

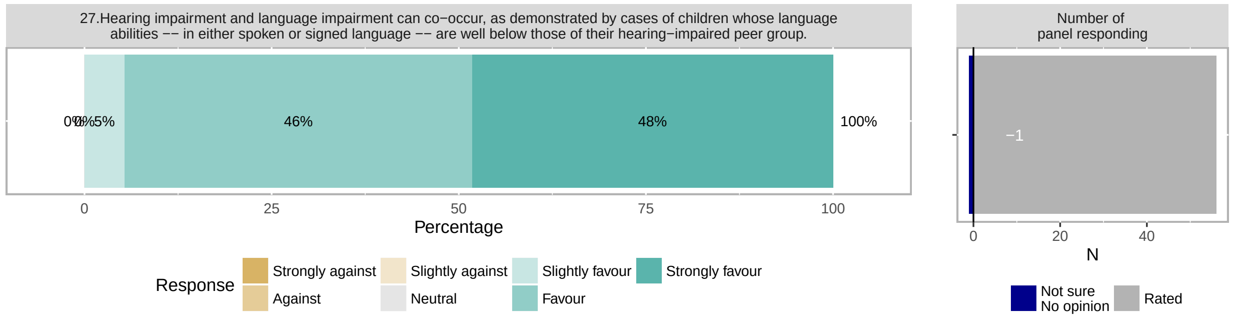

Figure 55: Percentage of panel members in each response category to statement 27. The percentages shown at each end of the scale are the cumulative percentages for the top and bottom three categories respectively.

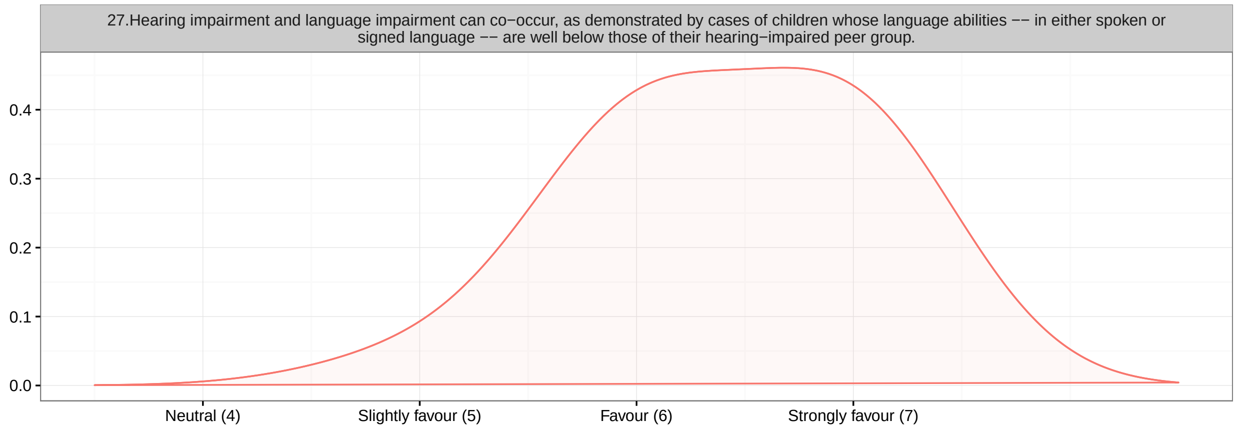

Figure 56: Distribution of responses to statement 27. The bold vertical line coloured red is Anonymous's response to this question for reference.

Table 27: Comments for each statement.

ResponseID	Q27B
$R_5uxk08XTwJpUk9D$	It would be surprising if this was not the case!
$R_6Dvhy7Alhw5wqIR$	we have lost communication in this question–that too can co-exist
$R_71b9fvukXBUQ5dr$	But hard to tell: child's input and experience hard to assess.
$R_3rrKtkb2VvC3uG9$	Another instance of multiple directions in the same item. The issues of sign language vs spoken language, receipt of CI , untreated mild hearing losses, and use hearing aids all have different dimensions that affect the judgement of agreement. The issue of whether interventionists need specialist skills plays out differently across these issues.
$R_1z8h1XMT676UOwd$	Adequate access to speech sounds appears to be critical for the development of language in children with even mild hearing loss (e.g see recent work by Bruce Tomblin and Mary Pat Moeller)
$R_6mrinfsu6CeSmBn$	Needs skilled assessment to differentiate these things.
$R_9uJ5LinD5e8X5Yh$	No brainer really but not universally understood. If you can have SLI + ASD/PD/EAL then you can have HI + SLI!
$R_es7hPPlfD7bdd65$	"can co-occur" is pretty vague though. Let's say a kid born with the key risk factors for LI also happens to have SNH due to some other factor (say, drug-induced auditory nerve damage . Then yes we would expect that child to have poor language for reasons that are at least partially independent of the peripheral auditory impairment.

$R_e9cPjWuFpcer4B7$	Same sort of problem since HI are not really a homogeneous group. This would be true of a children with marked sensor I neuronal loss. It would not be true of a child with OME. Deaf children are a different group and it depends on whether they have been raised signing by deaf parents. Cochlear Implanted children are another group again.

2.4 Final comment

Table 28: Comments for each statement.

ResponseID	S8
$R_1L0uyOsRR9gYKAB$	A very helpful clinical tool would be trajectories rather like the ones derived for mobility in cerebral palsy (Peter Rosenbaum's work in CanChild) for language and speech
$R_5cd8BDkYcGfGLKl$	Fantastic work in distilling this information. / / the only thing I'd like to see more clearly laid out is the issue of delay versus disorder, i.e. children with uneven profiles of language impairment are prioritised for SLT services over those with 'flat' profiles of impairment.
$R_8AhxnQPe8mJkUoR$	Thank you for synthesizing the large amount of information provided by Delphi 1 and establishing the Delphi 2 platform for further evaluation and discussion. / / As already mentioned in the report: fascinating information. An importantly, evidence of the potential that we can move the field forward. / /
$R_2f9ctxaHBJuJdLD$	It has been very interesting and educative- I still would welcome advice on terminology and nosology around sli and SLI though for the jobbing clinician these terms all seem opaque- perhaps we could all contribute to a shared terminology in all specialities that makes sense – watch out for PDA and whether SCDisorder will make it into ICD11.
$R_4HGIGYFIvMxLWcJ$	I liked the three broad categories of referral, assessment/intervention, and accompanying conditions.
$R_0ofhSCmeppIQ8kt$	I was less clear of the aims of this questionnaire...though the questions were more focused. Are we narrowing in on the profile of these 'unexplained' kids before we consider what to call them?
$R_6Dvhy7Alhw5wqIR$	I support a systematic battery of tests perhaps based on the areas currently included in the ICD11 language disorder defn to establish better profiles of strengths/weaknesses rather than the global lumping currently done with CELF / also sorting out what is included in communication versus pragmatics versus pragmatic language imp eg NVC or not–CN and I currently writing something about this
$R_bwwc7dPFEcp1azH$	I look forward to dealing with the issues of terminology- having more broad based terms to include children with co-morbid and concomitant conditions will be welcomed
$R_2o7JoTNgC3lqSIR$	The preamble to this questionnaire refers to 'identification' but many of the questions relate more to diagnosis. There is an important difference between the two terms. / This discussion would benefit from wider input, reflecting the experience of teachers and others who work with children with language impairments on the ground.
$R_e5KJQmN6txthTRX$	We welcome this attempt to clarify the terminology. The current usage continues to be confused, and the lack of clarity means too many children get lost. We urgently need better and easier identification tools to enable non-specialists such as teachers and parents to get to grips with the condition(s).
$R_2hLYvspULpn8NnL$	My understanding of the original task was to identify a common nomenclature for LI. I don't see much in this survey that applies to nomenclature.
$R_6JOosydU46ZndMF$	throughout this stage 2 , the emphasis appeared to be on 'language' in that most items referred to language rather than the broader term communication. Was this intentional? My concern was that I was not sure whether the term 'language' was being used specifically or generically. I believe that greater attention to 'communication' is necessary. Also, I had several concerns about seemingly limited understanding about mental health/neurodevelopmental disorders in children: psychiatric and SLP perspectives seem very different and there need to be a breakdown of these silos and better communication betwen the world of SLP/Communication Disorders and Psychiatry/neurodevelopmental disorders.....
$R_1TXxdyLg1UFCx4V$	I think this round is significantly better than the last and shows the real value of this approach
$R_dguQPTfUoDzSKB7$	I think dealing with second language issues, validity of measures, and which statisitics to use for those measures is important.

$R_6RlkuyWJYcIIsmN$	Please don't lose a focus on social disadvantage. Its effects on language and literacy are pervasive and longlasting. Irrespective of aeitological factors, this is a high-priority group that stands to benefit from rigorous application of evidence in early years' classroom settings. I know there are pros and cons in labelling such children, but the use of a label may be the only means by which they receive the skilled expertise they require. They are also at greater risk of associated difficulties in the attention and behaviour realms. At a population level, low-SES poses a signficant threat to the deveopment of language competence across the lifespan. Academic and vocational success will be more, rather than less, reliant on language competence in the future, due to the erosion of low-skilled jobs.
$R_cIxZunCo2wnTfVj$	In general, I feel that the questionnaire is directed as older, school-aged children rather than targetting some of the questions to the younger preschool ppulations.
$R_3rrKtkb2VvC3uG9$	Overall, my impression is that the items carriy assumptions that take us back decades in how to think about identification of children with language impairments, eligibility of services, and intervention commitments. Have we really lost this much ground in the last 10-15 years?
$R_5C49A94jWehNBB3$	Some consideration should also be given to which difficulties should not be incoprorated in to the language and communication impairment constructs. Otherwise, we run the risk of equating any educational or interpersonal challenge with LI.
$R_23qAFVuJCo6YHOd$	Understanding and use of language in real life is what we ultimately are trying to measure. I think the main impact of language impairments in children can be difficulties accessing the curriculum in relation to language and literacy. In addition, impacts relate to social interaction and independence. Therefore, perhaps future priorities for assessment of severity and need (rather than being abstract tasks which try to unpick aspects of language) should try to mirror i) the overall language demands of the curriculum ii) overall language demands of literacy tasks in the curriculum, iii) language demands of social situations, iv) language demands for independence. Although I think it is important and useful to have tasks to try to assess aspects of language when planning intervention, when trying to gauge severity (and impact), then how about a package of assessments that is specifically designed to mirror classroom language demands? The best fit so far is possibly a USP (CELF) type test and a narrative type test. This could go some way towards resolving the problems of classroom observations being important - but difficult to quantify. There could also be different types of tasks for different year groups as this is the reality in the classroom. Pragmatic language demands increase dramatically as the child goes through the key stages (giving key information, organising responses), as do the vocabulary learning demands. I think nearly all the kids I have worked with who have shown early language difficulties, struggle particularly with pragmatic language of the curriculum later on. Clearly different tasks would mean it wouldn't be possible to evaluate direct progress, but they could specifically be used to gauge how well a child can access and engage with the environment they are in. / / Also, I think we need to be careful to be clear about what 'markers' may tell us, and how they should not be used to evaluate the severity or impact. /

$R_8bIXFrv4VBlvVyZ$	I am very pleased for children with language impairments, their families and for clinicians and researchers that this study is underway, I expect it will make a huge contribution to the field. Just a couple of comments: / (1) At the point where the consensus statement is developed, consideration needs to be given to how the statements and criteria will be interpreted in practice and by different groups of practitioners, e.g. SLTs or psychologists. So the detail and wording are very important (use of examples, words such as "normally" "generally" "includes/including" etc). One thing that we have observed is creative use of assessments, interpretation of criteria in order to access resources/supports, but this may not always be in the best interests of the child. / (2) In terms of further statements or areas that might be considered-I think insufficient attention is paid in practice to the evidence for the the language "learning" impairment and how the child progresses/responds over time. We are aware that identification criteria and assessment approaches have over-relied traditionally on cognitive referencing based on NVIQ and cut-off points, with overuse of static omnibus standardised assessments (to quote De Villiers "of questionable linguistic value"); additionally previous knowledge and practice around the identification of language impairment, wasn't taking account of emergentist accounts of language learning and impairment or that for example, working memory and procedural learning, consolidation etc are relevant. I don't know how well these can be captured in a future checklist or agreed definition and there are challenges in terms of how some of these could be "operationalised" in clinical practice (thinking of the earlier statement about dynamic assessment for example) but I would like to see consideration given to these areas-as it bears not only on robust identification but also in directing more effective intervention. We have had a number of studies point up the learning mechanisms in language impairment and the limitations of static approaches to identification. Since the CATALISE study might be one of the most significant in this area for the foreseeable future, I think it's important that the statements and guidelines which arise from it are as comprehensive and future focussed as possible. / (3) In relation to the proposals around authorship, if it were possible to have a role I would be very interested in making a contribution if it is deemed useful to the CATALISE group or at least to be listed a member of the Delphi panel who isn't contributing significantly to the study design, analysis and write-up. / Also for information, at a point where publications are being prepared if it is deemed useful, I may be in a position to access outcomes of a national survey of SLTs which included questions around identification criteria currently in use. /
$R_3VHaciSzwJGKIU5$	I have concerns about the suggestion that we have tests with strong reliability and validity. Even the 'best' language measures are problematic and often not predictive of future levels of performance. This is especially true for the under 5s.
$R_7WXquZJy8WlgXAx$	I continue to find this process fascinating and am gald to be part of it. it has been even more difficult this time to respond using a scale and I have been grateful for the comment boxes. the interface between idenitfication, diagnosis, decision about intervention etc means that virtually all of the questions has a 'yes but.....' clause. the background paper was hugely useful. many thanks!
$R_1QTm7VrpDX1OAi9$	Much clearer and easier to rater this time.
$R_9uJ5LinD5e8X5Yh$	Thank you for doing this work. Hopefully we get some cohesion as an outcome. / Please do take on board the imbalance in professionals you have in this survey. Lots of SLTs' responses would outweigh the educationalists..........but children's education is what it's all about! Can you split the responses and see differences/similarities? That would give us food for thought! /
$R_cCuacCYZiqQHKgl$	I would be interested in a discussion about whether we diagnose language impairment on the basis of the functional impact of the child's language status or on the basis of a presumed impaired system supporting language development. The later case emphasizes factors within the child that consist of etiologies whereas the latter emphasizes the relationship of language ability and current or subsequent function. It is conceivable that one could argue for both.
$R_es7hPPlfD7bdd65$	I found this round a lot easier, maybe because I really understand the process better now. The questions were a lot easier to agree with but because the "some" and "can" statements it was hard not to endorse a lot of these. / / Either way: Thank you for including me in this process. I've learned a lot and my own views of the issues have evolved considerably as a result of the process.

$R_e 9cPjWuFpcer4B7$	I would say that the link with psychopathology needs to be explored more fully. We need to be careful in simply associating behaviour and language in too simplistic a fashion. I would also say that we need to be careful about a binary low SES/non low SES needs to be avoided because there is some pretty clear evidence not touched upon in the review that there is a pronounced social gradient not simply a flat line for all social groups and then a drop off for some putative “bottom” group. Finally, although I appreciate that this would have been difficult in a questionnarire which is already long it would he a been helpful to have a better understanding about intervention and service delivery and how people interpret the child’s needs. This is important because it often drives the way people look at these children - at least more than specific tests which mostly do not directly inform intervention. Dynamic assessment is mentioned in one question and a single reference but we also need to consider the response to intervention system in the U.S. which is in many ways similar but more systematic.
$R_e LIdYhExxkQtUZn$	Additional topics - culture and multilingualism. For clinicians and researchers these issues are highly relevant to consider when diagnosing and planning intervention, yet are complex to tease out.
$R_3 sXNbQYRlZaMb3L$	Difficult ——- to judge what SALTs have expertise to do. If the questions really mean that SALTs are best place of the available professions, then I endorse those items.
$R_3 DfMsLnqK54HqcZ$	We should consider the broader context with in which this “specialist assessment/treatment” would sit - what should happen in the “grey areas” of e.g. - children ’at risk’ pre-school but not severe enough for a diagnosis/specialist referral - children who appear to be functioning well in primary school but who may be at risk of later underachievement - children with fluctuating levels of need. I would be very happy to be a part of a group considering these issues. / / Thanks for your patience regarding my late response.

Contact Information

Oxford Study of Children's Communication Impairments (OSCCI),
Department of Experimental Psychology,
Tinbergen Building,
South Parks Road,
Oxford,
OX1 3UD,
UK.
Tel. (+44) 01865 271386
fax. (+44) 01865 281255
Email. oscci@psy.ox.ac.uk

Centre for Reading & Language @ Oxford,
Department of Experimental Psychology,
Tinbergen Building,
South Parks Road,
Oxford,
OX1 3UD,
UK.
Tel. (+44) 01865 277419
Email. CRLOxford@psy.ox.ac.uk

Health Experiences Research Group,
Nuffield Department of Primary Care Health Sciences,
Radcliffe Observatory Quarter,
Woodstock Road,
Oxford,
OX2 6GG,
UK.
Tel. (+44) 01865 289363
Email. trish.greenhalgh@phc.ox.ac.uk

Copyright Information

This copy of the CATALISE Delphi results has been supplied on the condition that anyone who consults it recognizes that its copyright rests with the authors and that no quotation from the report or information derived from it may be published without prior consent.
